# Supplementary material for: Gasdermin E is Dispensable for H1N1 Influenza Virus Pathogenesis in Mice
Source: bioRxiv. 2025 Jul 29:2025.07.29.667514. Preprint. [Version 1] doi: 10.1101/2025.07.29.667514 (PMC12324273; doi:10.1101/2025.07.29.667514)
Supplement: Supplement 1 [file media-1.pdf]

gene	cluster	WT_InfvsMoc	WT_InfvsMoc	KO_InfvsMoc	KO_InfvsMock_padj
Cdc45	orange	1.92884334	2.09E-19	1.88534913	8.60E-18
H19	skyblue	-1.6424284	7.03E-06	-0.851421	0.02878107
Cav2	red	-0.9335846	1.34E-05	-0.8042964	0.00027357
Klf6	orange	0.8728701	1.30E-12	0.55555351	1.43E-05
Tbx2	red	-0.733841	7.83E-07	-0.5594865	0.0002772
Wnt9a	red	-0.8498308	0.00240453	-0.2438622	0.44456332
Xpo6	orange	0.82317906	2.51E-10	0.77908927	3.67E-09
Axin2	red	-0.7959924	5.56E-05	-0.7731261	0.00013123
Slc22a18	red	-1.0469053	2.56E-06	-0.6448993	0.00573982
Pih1d2	red	-1.0308542	0.00050357	-0.0922554	0.79537579
Fgf23	yellow	3.82814158	0.00040528	3.5248553	3.58E-05
Nalcn	skyblue	-2.150931	0.00332943	-0.756844	0.32653805
Slfn4	black	8.52797878	3.06E-111	7.48233918	7.16E-97
Tspan32	orange	0.73564681	0.00030458	1.25944637	1.24E-09
Lhx2	black	4.68599788	1.61E-09	5.97836216	9.32E-09
Gmpr	skyblue	-1.5000948	6.54E-09	-1.3323724	5.43E-07
Mid2	red	-0.9436883	4.96E-06	-0.6942044	0.00121025
Trim25	orange	1.43192406	4.44E-25	1.21474386	3.78E-18
Scpep1	orange	0.71017073	0.00116841	0.58002007	0.01075027
Itgb2	yellow	2.59852941	2.42E-15	2.36860263	9.69E-13
Hddc2	orange	0.55529533	0.01094454	0.63921507	0.0040822
Tpd52l1	red	-1.5054475	0.00012265	-0.7632342	0.07868237
Cdh4	red	-1.0897592	0.00775348	-0.8696099	0.04073213
Bcl6b	orange	1.18826353	0.00089035	1.15240166	0.00168806
Clec10a	orange	1.03561928	0.00023132	1.32690684	3.22E-06
Arvcf	red	-1.502843	1.33E-07	-1.02996	0.00049638
Rtca	orange	0.63360097	4.52E-06	0.7487309	9.40E-08
Rem1	red	-1.0481287	8.64E-05	-0.9056515	0.00099752
Tbrg4	orange	0.93475539	4.64E-09	0.82561347	4.15E-07
Tmprss2	red	-1.4942138	1.06E-13	-1.0025063	1.41E-06
Mx1	black	6.21935933	4.11E-79	6.35017095	4.89E-81
Egfl6	red	-1.0093457	0.00066741	-0.4634148	0.15191645
Lck	yellow	2.64914809	3.81E-07	3.59907634	4.99E-12
Cttnbp2	red	-1.4415748	1.88E-07	-0.7076606	0.01556756
Galnt1	orange	0.72093665	5.66E-06	0.63875725	9.29E-05
Sept1	yellow	2.39398302	1.13E-33	2.59264637	8.11E-39
Acvrl1	red	-0.9657486	4.83E-11	-0.9096154	1.03E-09
Tom1l2	red	-0.7830779	2.09E-08	-0.6748216	2.47E-06
Zfp385a	orange	0.88091893	0.00128741	0.6676618	0.01971043
Itga5	orange	1.39715245	5.31E-06	1.35115856	1.70E-05
Adora3	yellow	2.52034982	2.59E-09	2.75479694	1.20E-09
Hnrnpd	orange	0.85005339	1.85E-13	0.79117525	1.50E-11

Gm2a	orange	0.58957536	0.00141192	0.27320184	0.17546667
Sema4f	red	-1.3794762	3.59E-09	-0.9922996	4.33E-05
Haao	orange	1.22233445	2.15E-05	1.03567733	0.00050859
Cd52	yellow	3.44558914	1.12E-35	3.08200614	1.67E-28
Loxl3	orange	0.74709353	8.44E-06	0.64303449	0.00019674
Rab5b	red	-0.607886	4.49E-07	-0.5249424	2.26E-05
Serpinf1	skyblue	-1.8240437	4.06E-13	-0.9641605	0.00024156
Tcf7	orange	-0.0959541	0.84627805	1.12034345	0.00887383
Il12rb1	black	5.40474755	4.77E-42	5.4378242	1.91E-41
Slc5a5	skyblue	-0.7727786	0.30827757	-2.1009179	0.0071946
Kcnn3	red	-1.0553365	0.0010009	-0.5070087	0.14083378
Car4	red	-0.7576541	0.00108077	-0.6995335	0.00336138
Txnrd3	red	-0.9406323	1.52E-05	-0.8264709	0.00023557
Fasl	yellow	3.77810147	3.80E-11	3.43639052	2.32E-08
Il4	skyblue	-1.566781	0.00327445	-1.3620086	0.01234153
Pxmp4	red	-0.7450768	7.52E-06	-0.654841	0.00013237
Dlg3	red	-0.6819356	1.06E-06	-0.5668882	7.73E-05
Mmp11	red	-1.2277788	8.08E-09	-0.8591474	0.00010278
Vpreb3	skyblue	-3.6825086	3.15E-15	-2.5155342	9.88E-08
Hoxa4	red	-0.6466697	0.00486645	-0.3899176	0.12040627
Mmp14	orange	1.93987109	2.32E-10	1.70249385	5.19E-08
Ccl3	black	5.76593325	6.07E-65	5.11484331	6.60E-57
S100a4	yellow	1.91923593	0.00743203	2.56887421	0.00783685
S100a6	orange	1.33819662	5.35E-08	1.27028576	4.16E-07
Scn4a	red	-0.9571357	0.00032045	-0.762562	0.00641423
Epn2	red	-0.7852815	1.69E-06	-0.6338744	0.00018271
N4bp3	red	-0.6537136	2.65E-09	-0.6480793	5.92E-09
Nhp2	orange	1.05138993	5.49E-07	1.02570167	1.85E-06
Mfsd10	orange	0.71755246	5.01E-05	0.57258917	0.00179679
Lgals9	orange	1.88460139	5.31E-24	1.85540893	3.16E-23
Cfp	orange	0.70706001	0.00027417	0.64638754	0.00130395
Timp1	black	6.15335123	6.03E-35	6.01825166	2.76E-33
Uxt	orange	0.73976994	0.00148705	0.37250742	0.14075643
Cnnm3	red	-0.6698148	0.00086621	-0.5692173	0.00620659
Mxd1	orange	1.72628126	1.27E-18	1.53686454	7.98E-15
Oas1c	orange	1.17037135	7.80E-08	1.04374748	4.06E-06
Oas1h	black	6.16179678	6.32E-06	4.87204506	4.38E-06
Sema6b	orange	1.57327672	7.49E-08	1.07433844	0.00041434
Uhrf1	yellow	2.64224952	1.01E-23	2.92551609	1.99E-28
Ramp2	red	-0.7079532	0.00217021	-0.6750532	0.00445001
Gramd1a	orange	1.01362849	2.72E-29	0.73175161	1.64E-15
Hpn	red	-0.6247772	6.90E-05	-0.1933128	0.26862157
Itgb7	yellow	2.30773289	5.04E-27	2.12325202	7.12E-23

Rarg	red	-0.9489458	2.85E-12	-0.7143145	3.29E-07
Efnb2	skyblue	-1.8416586	2.19E-15	-1.4259874	1.95E-09
Rrp15	orange	0.66166073	0.00922179	0.84578863	0.00105077
Rnd2	red	-0.6653604	0.00219818	-0.6435987	0.00395558
Srr	red	-0.6658932	1.88E-06	-0.4772369	0.00106378
Sync	skyblue	-1.687352	2.42E-11	-0.9097688	0.00060339
Fndc5	skyblue	-1.7476784	0.01064364	-2.7448222	6.25E-05
Acp5	orange	1.64557654	1.43E-08	1.25558707	2.87E-05
Cnn1	skyblue	-1.6464721	1.15E-11	-0.6985339	0.00669709
Ube2c	yellow	3.11661452	2.02E-24	3.33605824	1.14E-26
Cct3	orange	0.70616329	2.41E-05	0.56914937	0.00101282
Col18a1	orange	0.64096284	0.01268739	0.7711614	0.00305708
Kpnb1	orange	0.59676614	1.77E-06	0.59244166	3.36E-06
Tbx21	yellow	3.54029342	2.76E-68	3.77578234	5.17E-71
Tubb6	orange	1.76094442	1.56E-06	1.91832281	2.49E-07
Meox1	red	-1.3013653	1.73E-08	-0.8322526	0.00052788
Sost	skyblue	-1.856129	2.74E-06	-1.577481	0.00015844
Nkx2-1	red	-1.2622317	3.87E-08	-0.8027626	0.00081491
Irx2	red	-0.8042901	0.00028152	-0.4562103	0.05352411
Col1a1	orange	0.72398668	0.0280677	0.92436992	0.00542937
Itga3	orange	0.50194573	0.00365738	0.73659005	2.12E-05
Sgca	red	-0.8429488	0.00066005	-1.1524014	6.98E-06
Foxm1	yellow	2.46961986	5.50E-34	2.62130763	2.02E-37
Tubb5	orange	1.15071771	1.58E-09	1.01018544	2.17E-07
Ell2	orange	0.78350654	4.69E-05	0.500292	0.01332754
Jup	red	-0.9852849	6.94E-15	-0.8414938	6.33E-11
Acap1	yellow	1.81769927	1.49E-25	2.04351233	1.48E-31
Tcea3	red	-1.4005625	8.60E-11	-1.0354566	3.53E-06
Ifrd1	orange	0.801628	4.73E-12	0.52808808	1.14E-05
Stk38l	orange	0.41206265	0.00479216	0.70919302	1.01E-06
Brpf1	orange	0.81213617	2.56E-10	0.57386701	1.52E-05
Gstt1	red	-1.2535379	3.14E-06	-0.9772404	0.00044831
Gstt3	red	-0.8828657	1.39E-05	-0.6514478	0.00216128
Tat	orange	1.28316248	0.00311832	-1.1154195	0.01455459
Gramd3	orange	0.83978592	2.03E-10	0.85787475	1.68E-10
Eef1e1	orange	0.99229352	0.00081065	0.92806951	0.00239615
Cldn15	red	-1.0307108	0.0052167	-0.6655131	0.09015648
Il16	orange	0.50492568	0.00139825	0.63342781	7.74E-05
Tcirg1	orange	1.89659502	4.32E-19	1.83803358	7.26E-18
Smo	red	-0.8643607	7.41E-13	-0.5935533	1.87E-06
Tspan33	orange	0.93319028	6.18E-06	0.39771765	0.0827103
Folh1	orange	2.32582638	0.00189309	1.36974162	0.06721875
Lrp3	skyblue	-1.4781644	1.43E-14	-1.6114027	5.01E-17

Cpa3	skyblue	-2.3283458	3.62E-08	-1.4657094	0.00056798
Nfix	red	-0.7569254	9.10E-13	-0.5521654	4.37E-07
Uba1	orange	0.65162936	6.77E-06	0.61505265	3.37E-05
Siae	red	-0.3003766	0.15205863	-0.5966088	0.00367343
Spa17	red	-1.2350424	3.63E-09	-1.1703319	5.87E-08
Grik3	skyblue	-1.9863301	0.00022724	-0.8584816	0.18679042
Sipa1l2	red	-0.8206547	3.97E-08	-0.6782076	1.01E-05
Blvra	orange	1.10772303	5.11E-07	0.88132782	0.00010904
Pdzd4	red	-0.6863953	0.00028428	-0.5287061	0.00753812
Srpk3	red	-1.1719466	0.00021365	-1.1296324	0.00060667
Pnck	skyblue	-1.4729154	4.32E-09	-1.1707972	6.17E-06
Ssr4	orange	0.65529103	0.00038707	0.69910083	0.00020895
Ltbp2	red	-0.704733	0.00300127	-0.6454668	0.00837543
Ift46	red	-0.7159186	1.88E-05	-0.4463794	0.01104735
Tmem25	red	-1.4282462	4.50E-06	-0.8000323	0.01714846
Cd3g	yellow	3.56368443	2.97E-25	3.43154567	5.30E-23
Spag5	yellow	2.99686472	1.62E-34	3.5274723	5.87E-43
Unc119	orange	0.59094572	0.00615248	0.53015259	0.0178705
Ccne1	yellow	3.29856608	1.11E-25	2.83914099	2.34E-19
Psmc3	orange	0.67857414	5.41E-05	0.58050881	0.00082652
Acp2	orange	0.7722992	0.00056369	0.50293389	0.03349515
Rapsn	red	-0.928292	0.00457567	-0.6608537	0.05925388
Celf2	orange	0.67924549	4.65E-06	0.30142218	0.06008948
Spi1	yellow	2.90859969	1.41E-23	2.67283472	6.70E-20
Smg9	orange	0.82386455	1.52E-09	0.80772869	5.56E-09
Rmnd5a	red	-0.8881111	0.00072556	-0.4484255	0.11418127
Mov10	orange	1.50543222	2.66E-28	1.20035152	3.03E-18
Ppm1j	orange	1.11875321	1.06E-05	1.38595455	8.22E-08
Rhoc	orange	1.49398877	1.42E-07	1.41656305	1.02E-06
Def6	orange	1.86452293	3.17E-33	1.88353237	1.29E-33
Zim1	red	-0.4287768	0.40496438	-1.5054131	0.00332419
Angptl4	orange	1.39108031	8.17E-17	1.10665261	6.99E-11
Dbf4	orange	2.05035705	3.77E-16	1.65824375	7.46E-11
Daxx	yellow	2.46876625	7.02E-35	2.26865601	1.51E-29
Ipo4	orange	0.46981074	0.02415679	0.63160045	0.00259339
Rec8	red	-1.3100133	2.45E-05	-0.5741791	0.08529432
Irf9	orange	1.21852812	1.22E-23	1.21109055	2.71E-23
Slc25a42	red	-1.157098	2.38E-05	-1.1069896	8.20E-05
Dyrk1b	red	-0.8427883	1.81E-05	-0.675155	0.00090016
Ndufb2	red	-0.8352005	0.00109045	-0.7731059	0.00364483
Rgs19	orange	1.18723118	2.55E-07	1.09356304	3.63E-06
Abhd3	skyblue	-2.6216739	1.94E-12	-1.5510852	3.59E-05
Snrpd1	orange	1.14990523	2.39E-09	0.96677301	1.16E-06

Tsc2	red	-0.792668	3.95E-07	-0.6177061	0.00013121
Rpl3l	skyblue	-1.6153837	8.03E-06	-1.3263579	0.00042005
Slc9a3r2	red	-1.0904485	1.40E-08	-0.9704483	7.96E-07
Ikzf4	orange	1.66790866	7.42E-10	1.51139606	4.61E-08
Pon1	skyblue	-2.6564591	0.00013546	-1.2908381	0.0844935
Tgfb1	orange	0.58581467	0.00011203	0.48981696	0.00178158
Shh	skyblue	-1.6468214	3.64E-08	-1.1956509	0.00012152
Gtf2f1	orange	1.21265107	7.46E-11	1.11695019	3.86E-09
Dennd1c	orange	1.86546049	1.12E-25	1.94943437	1.34E-27
Prkd1	red	-0.8390857	2.16E-09	-0.7501332	1.60E-07
Lcp2	yellow	3.25944095	7.23E-38	3.28626334	2.87E-38
Naa20	orange	0.92228253	1.03E-07	0.687372	0.00013063
Pex6	red	-0.8818601	3.19E-10	-0.8411738	3.71E-09
Tmem143	red	-1.0112801	9.54E-09	-0.6991277	0.000149
Ggct	orange	1.79041787	1.88E-19	1.67363451	6.22E-17
Jag2	red	-0.5735144	1.27E-05	-0.6072663	5.54E-06
Epdr1	skyblue	-1.9665204	9.72E-40	-1.2128496	5.20E-16
Top3a	orange	0.61518489	0.0001032	0.46214495	0.00492803
Qtrt1	orange	0.52857175	0.00833918	0.61232402	0.00271527
Chaf1a	orange	1.86517562	2.82E-13	1.82592047	1.73E-12
Pla1a	yellow	3.92611013	2.01E-25	3.91562714	6.03E-25
Mcm2	orange	1.55727907	3.22E-14	1.47990645	9.98E-13
Tpra1	red	-0.7738334	1.52E-07	-0.5932909	1.00E-04
Adgre5	skyblue	-1.7184481	7.91E-12	-1.3569631	1.40E-07
Il17ra	yellow	1.96390946	6.08E-19	2.04864795	2.05E-20
Kcnn1	red	-1.0858328	0.00340955	-1.1212048	0.00571437
Arrdc2	orange	0.66185281	0.00011469	-0.0875434	0.67342967
Cd36	red	-1.2181073	2.11E-09	-1.0567978	3.78E-07
Bcam	red	-0.622308	7.20E-05	-0.4464289	0.00635042
Relb	orange	1.5688286	2.38E-11	1.5217	1.59E-10
Tomm40	orange	0.7990841	4.28E-08	0.62216767	3.71E-05
Hbp1	red	-0.7161276	0.00032405	-0.5603927	0.00670953
Prkar2b	skyblue	-1.7138995	4.59E-09	-1.4515505	1.30E-06
Cdkn1b	red	-0.622768	0.00063239	-0.5522104	0.0033082
Rab8a	orange	0.94531292	5.92E-10	0.74784562	1.95E-06
Hmgn2	orange	0.74023807	3.66E-08	0.57438705	3.50E-05
Cnot11	orange	0.59281099	4.64E-06	0.43393269	0.00125592
Slc2a3	orange	1.04849294	2.08E-14	0.500045	0.0005753
Ebi3	yellow	1.76095866	9.24E-05	2.06487827	2.27E-05
Edar	red	-1.1660795	0.00584514	-0.0038965	0.99385519
Car11	skyblue	-2.1609064	2.62E-14	-1.9430145	3.45E-12
Hck	yellow	2.12558027	1.77E-12	1.93139016	2.86E-10
Ap1m2	red	-0.6158221	0.00099318	-0.2764812	0.17526073

Mob3a	orange	1.15943103	4.89E-06	1.05951798	4.61E-05
Cacnb3	orange	0.77620198	4.08E-05	0.71955597	0.00021896
Ccdc65	skyblue	-1.6275461	4.38E-09	-1.1696736	3.14E-05
Pld3	orange	0.68326351	2.46E-05	0.48410832	0.00417317
St8sia6	skyblue	-1.6640615	2.63E-05	-0.7094797	0.09181042
Fcgrt	red	-0.8466156	4.23E-07	-0.7657637	8.15E-06
Rps11	orange	0.67812667	0.00047424	0.4927472	0.01519356
Phyhip	skyblue	-1.2263351	0.00012632	-1.2709809	0.00012635
Crhr2	skyblue	-1.4637578	1.05E-05	-1.2964534	0.0001901
Inmt	skyblue	-2.2984706	1.03E-05	-1.8909459	0.00044595
Cyp4f18	yellow	4.02440329	4.07E-21	3.40740941	2.91E-15
Impdh1	red	-0.6748478	9.31E-09	-0.6475494	6.41E-08
Dusp3	red	-0.7849959	1.74E-06	-0.6568598	0.00010374
Prodh	red	-1.0738231	0.00276502	-0.760746	0.04316857
Slc25a1	red	-0.3546491	0.12890946	-0.62025	0.00697151
Homer3	red	-0.7244573	9.33E-14	-0.6207599	4.53E-10
Rnf215	red	-0.7826216	3.62E-07	-0.5356003	0.00081505
Sec14l2	red	-1.2779483	6.91E-16	-0.9830983	7.04E-10
Cp	orange	0.51636982	0.00048944	0.61231196	4.56E-05
Rps6ka1	orange	0.67490046	1.25E-11	0.27665	0.00931167
Has1	orange	1.3886948	0.00435064	2.48411867	4.74E-07
Itpkc	orange	0.77237287	0.00019048	0.6277805	0.00342738
Kif20a	yellow	2.74955221	8.86E-25	2.71693615	1.24E-23
Farsa	orange	0.80461085	1.65E-10	0.6988219	5.73E-08
Dnase2a	orange	1.1481101	1.10E-07	0.97196433	1.40E-05
Syce2	orange	1.36403542	1.49E-07	0.81149792	0.00290512
Nqo1	red	-1.060672	1.29E-05	-0.4887044	0.0600515
Gys1	red	-0.912748	1.78E-08	-0.5425176	0.00142347
Bax	orange	0.8784917	1.49E-05	0.67253089	0.00140521
Il7r	yellow	2.24201949	3.01E-14	1.61902291	8.56E-08
Zfp81	red	-0.4010569	0.0651282	-0.5830466	0.00717665
Efnb3	red	-0.2446738	0.59777036	-1.1441982	0.00799838
Mmd	red	-0.3597896	0.03586152	-0.6540355	0.00011999
Hlf	skyblue	-2.3617798	5.09E-13	-2.1068349	2.16E-10
Fam162a	red	-0.6446793	7.73E-07	-0.5204247	0.00013172
Ssbp2	red	-0.5805315	0.0060504	-0.3023456	0.1874343
Gstm5	red	-0.5478984	0.03212475	-0.9299228	0.00029859
Gstm7	red	-1.3581931	0.00049254	-0.9304428	0.02373254
Stat3	orange	0.93703135	3.62E-14	0.96129243	1.00E-14
Stat5a	orange	0.6398536	3.29E-07	0.76192547	1.73E-09
Ptrf	red	-0.8918728	1.30E-11	-0.716475	1.15E-07
Zak	red	-0.9246649	9.95E-08	-0.6860956	0.00013724
Col5a3	red	-0.9161141	3.22E-05	-0.7685358	0.00081829

Dnmt1	orange	1.09903747	3.41E-14	0.95405828	9.46E-11
Ppan	orange	0.8471781	0.000139	0.87316	0.00012733
Angptl2	skyblue	-2.0078688	6.30E-21	-1.6607001	1.78E-14
Etv1	skyblue	-1.2352793	0.00015864	-1.5244295	4.13E-06
Kifc2	skyblue	-1.5436899	4.01E-06	-1.2304582	0.00036749
Psap	orange	0.73838035	0.00032475	0.53446498	0.01276852
Phb2	orange	0.59789203	2.32E-05	0.45201668	0.0021007
Ptpn6	yellow	2.28417502	6.87E-28	2.20273981	7.57E-26
Il12b	yellow	2.59677255	0.000228	1.31686307	0.07055532
Clcn5	orange	0.9175304	7.55E-06	1.09968566	1.19E-07
Hif3a	orange	1.92741888	8.25E-24	0.5873196	0.00335275
Pde1c	skyblue	-0.7831226	0.25009345	-2.6270314	0.00011257
Spic	yellow	1.84308523	0.00545182	2.19102391	0.00098898
9330159F19I	skyblue	-1.8753224	2.25E-06	-1.4762713	0.00037741
Il11	orange	1.46403802	0.00064088	1.47824737	0.00077926
Bid	orange	1.87895197	1.89E-18	1.94544358	7.92E-20
Dnajb11	orange	0.83365619	1.15E-06	0.61461759	0.00056086
Gab2	orange	0.61166882	4.36E-08	0.44990851	9.54E-05
Ctse	yellow	2.45888567	1.48E-09	2.53291721	5.53E-10
Ndrg2	red	-1.1525215	0.00011305	-1.0636781	0.00053438
Arhgef40	red	-0.590778	4.61E-07	-0.4219159	0.0005295
Cd33	orange	1.32635508	0.00011303	1.01516142	0.00444018
Nkg7	black	4.59832707	5.79E-72	4.33235126	1.98E-62
Stxbp2	orange	0.72343289	1.23E-06	0.66003194	1.62E-05
Sgce	red	-0.8639552	6.74E-09	-0.750252	9.10E-07
Slbp	orange	0.9752449	7.36E-13	0.71526745	3.60E-07
Aqp1	red	-1.2413124	1.76E-16	-1.0213003	2.84E-11
Myo9b	orange	0.71944532	2.45E-06	0.62191876	7.57E-05
Ly9	yellow	2.71556393	1.94E-23	2.42410262	1.53E-18
Cd244	yellow	2.96674007	1.39E-13	2.805945	5.06E-12
Adgre1	orange	1.77178329	0.00210365	1.464855	0.01443111
Mtfp1	skyblue	-1.3636188	3.15E-07	-1.5375534	1.72E-08
Pgf	orange	1.63017753	2.15E-14	1.80461	4.44E-17
Ulk2	red	-0.9419357	6.20E-06	-0.7372769	0.00064537
Ccl24	yellow	2.8468525	1.79E-09	2.45850137	2.12E-07
Plod3	orange	0.71101203	7.66E-07	0.62108409	2.66E-05
Ap1s1	orange	0.72276504	0.00062755	0.55126025	0.01246687
Lbr	orange	0.60803959	7.56E-08	0.61980575	6.74E-08
Bcan	skyblue	-2.4552676	1.81E-14	-1.2863466	8.45E-05
Matk	yellow	2.38607302	4.56E-14	2.09591249	7.03E-11
Nmrk2	skyblue	-1.6621408	0.00152962	-0.8705517	0.15470178
Rasa4	orange	1.77786201	2.73E-22	1.46898603	2.45E-15
Syt5	skyblue	-1.5205772	3.97E-10	-1.218616	1.17E-06

Sgsh	red	-0.8450335	1.73E-08	-0.8122331	9.59E-08
Chd5	red	-1.3711889	0.00042856	-0.9783777	0.01670933
Cstb	orange	1.67320644	4.19E-11	1.55130251	1.80E-09
Sh2b2	orange	1.17682853	0.00180716	0.7697428	0.05316345
Cd44	orange	1.06189785	1.08E-06	1.0515958	2.21E-06
Wdr1	orange	0.92048328	1.67E-07	0.89985465	5.21E-07
Slc2a9	orange	0.62817989	3.34E-05	0.4687786	0.00295515
4930550C14	red	-1.3542168	0.00082246	-1.1024744	0.00871465
Man2b1	orange	0.867267	4.26E-05	0.52918598	0.01789837
Spc25	orange	1.70561522	1.02E-13	1.59805675	8.72E-12
Dnah2	red	-1.1158472	2.87E-10	-0.7469533	4.46E-05
Ripk4	red	-0.7308372	9.64E-07	-0.3428302	0.03060396
Fgfr4	skyblue	-2.0907495	3.96E-15	-1.6996996	3.68E-10
Cadm3	red	-1.4653268	1.37E-13	-0.3280849	0.11981253
Slc1a3	red	-1.135299	1.39E-05	-0.938647	0.00050291
Mlxipl	skyblue	-2.3399896	5.92E-08	-1.7278693	0.00010496
Mcm5	yellow	2.46325542	2.46E-26	2.28998628	1.17E-22
Hmox1	orange	1.51821007	3.86E-08	1.77447803	1.64E-10
Il27ra	orange	1.57040112	7.43E-19	1.56332358	2.94E-18
Asf1b	yellow	2.97210021	1.50E-32	2.48102502	1.06E-22
Myl10	skyblue	-2.8772678	0.0014569	-0.9703596	0.2970046
Ddx39	orange	1.15376848	3.45E-19	0.74574895	2.20E-08
Por	red	-0.9181607	3.50E-08	-0.7276221	2.24E-05
Igf1r	red	-0.7537563	6.25E-07	-0.6078426	9.91E-05
Fcer2a	red	-0.9014767	0.00466815	-0.2597894	0.48094338
Cyp2a5	skyblue	-2.6857159	2.67E-07	-2.8131827	1.09E-07
Ube2m	orange	0.59522965	0.00011523	0.53891188	0.00070956
Adcy9	red	-0.9958386	0.00098155	-0.6767427	0.03300864
Mef2c	red	-1.0813746	3.17E-08	-0.74272	0.00027832
Mrvi1	red	-1.0150802	1.72E-08	-0.8867756	1.20E-06
Psmd4	orange	0.81478838	1.27E-08	0.59410018	6.27E-05
Tmod4	red	-1.1892694	0.000345	-0.2912889	0.45388119
Insrr	yellow	3.19658242	3.25E-13	2.45435058	1.80E-09
Mthfd2	yellow	2.99490762	1.48E-28	3.10162473	4.70E-30
Kit	red	-1.3499063	3.21E-09	-1.1289714	1.39E-06
Ampd3	orange	0.92261454	1.59E-06	0.67326194	0.00073068
Sh2d1a	yellow	2.97259694	8.23E-07	4.02164079	3.18E-10
Pvalb	skyblue	-2.0922892	3.32E-05	-1.3579152	0.01061247
Ranbp1	orange	1.10076226	2.88E-06	0.85089741	0.00050977
Cd247	yellow	2.20108056	7.58E-21	2.70906667	4.73E-30
Psmb4	orange	1.03756162	1.22E-08	0.86719129	3.60E-06
Mmp8	black	4.84208227	2.21E-09	3.60315342	1.28E-05
Tnfsf14	yellow	2.9141841	6.16E-17	2.69931135	4.12E-15

Gata6	red	-0.7860224	1.37E-08	-0.5740818	6.10E-05
Rsl1d1	orange	0.6494215	2.70E-05	0.43501096	0.00737904
Spo11	yellow	-0.0248985	0.98753802	5.72613793	5.23E-05
Nr2c1	red	-0.5966702	3.32E-06	-0.3430085	0.0111214
Otx1	red	-1.2114869	0.00937356	-0.8129464	0.08921787
Itgae	orange	1.32784524	7.50E-07	0.70053404	0.01493997
P2rx5	red	-1.3380665	1.69E-06	-0.7789193	0.00836291
Ephb3	red	-0.8977713	8.22E-10	-0.4440478	0.00400605
Calcoco2	skyblue	-3.9296267	0.00041713	-4.6636082	0.00035256
Snf8	orange	0.60121219	6.44E-05	0.37265532	0.01947137
Tbcb	orange	0.79018077	9.26E-08	0.49154727	0.0015166
Inpp5k	red	-0.5646891	0.00298822	-0.6037068	0.0018673
Eps8l1	red	-1.1007334	0.00042959	-0.7501095	0.0222085
Htra1	red	-1.1645399	7.32E-05	-0.57749	0.0671227
Epor	skyblue	-2.60582	3.07E-10	-1.6777218	7.79E-05
Ccdc159	red	-1.1669313	0.00193254	-0.8463609	0.02824091
Atp6v1b1	skyblue	-4.4687611	5.06E-12	-2.3459606	0.00017431
Tep1	orange	0.98838693	6.47E-08	0.81591099	1.49E-05
Arpc2	orange	1.07367644	6.45E-08	0.89816434	1.10E-05
Zbtb32	yellow	2.70124508	8.53E-28	2.65519293	8.55E-27
Susd2	red	-1.2948322	6.23E-14	-0.8021664	6.53E-06
Ggt5	orange	0.20000756	0.25384648	0.5884657	0.00042144
Ggt1	orange	1.83070369	3.53E-09	1.7918809	8.31E-09
Crip2	red	-0.910323	3.82E-06	-0.7554208	0.00020317
Cbfa2t3	red	-1.2409841	5.64E-11	-1.0789503	2.32E-08
Fbln1	skyblue	-1.4827617	2.67E-10	-1.1342239	2.75E-06
Pgrmc1	red	-0.9177827	8.08E-13	-0.7767864	2.92E-09
Tek	red	-1.2052115	1.77E-15	-1.0964068	8.34E-13
Cdc20	yellow	2.52189527	4.43E-36	2.45734524	1.21E-33
Adamts4	black	5.07188798	5.84E-26	5.6621652	1.88E-30
Rnf114	orange	1.36990814	1.28E-34	1.27195396	8.14E-30
Neurl1a	red	-1.3470689	1.37E-06	-0.961757	0.00097534
Srm	orange	1.05635421	0.0001125	1.01606733	0.00030572
Epha2	orange	1.16399931	5.11E-07	1.24769309	1.09E-07
Bbs1	red	-1.0790212	1.14E-08	-0.8259575	1.70E-05
Ptbp1	orange	0.58529366	1.26E-05	0.61924677	5.62E-06
Mvd	orange	0.88845664	0.00023248	0.96852611	8.38E-05
Cyba	yellow	2.15677498	4.58E-14	1.66436689	1.39E-08
Prkag3	red	-1.0437997	0.0048907	-0.2676541	0.53428635
Atp7b	skyblue	-1.7824392	0.00171949	-1.2366565	0.03860406
Slc4a3	red	-1.004569	0.00012369	-0.7753388	0.00427972
Cdt1	yellow	2.50215479	1.98E-24	2.00040469	7.92E-16
Runx1t1	red	-1.017632	3.05E-05	-0.8231256	0.00119246

Aprt	orange	1.44917985	1.70E-10	1.17770245	4.45E-07
Slc5a6	red	-0.9970808	3.23E-05	-0.9663198	8.12E-05
Tcf23	orange	1.75393994	2.31E-25	0.8229949	2.09E-06
P4htm	red	-0.8938257	2.68E-05	-0.9009045	3.10E-05
Pola1	orange	0.89781891	6.00E-06	0.96264735	2.66E-06
D130043K22	skyblue	-1.7321631	1.70E-07	-0.9655028	0.00159845
Gmnn	orange	1.35365302	4.82E-06	0.92632817	0.00284447
Acot13	red	-0.5959074	0.00010192	-0.8127127	2.20E-07
Zfp184	red	-1.3803182	0.00591516	-0.9199383	0.05645312
B4galnt1	orange	1.51737645	2.17E-11	1.2499184	7.37E-08
Krt23	red	-0.7986004	0.00353942	-0.5206853	0.07359249
Cnp	orange	0.76557252	2.36E-07	0.76121369	4.63E-07
Ttc25	skyblue	-1.6739376	1.20E-06	-0.9081658	0.01130202
Ezh1	red	-0.6554894	3.64E-07	-0.576293	1.34E-05
Hap1	orange	1.49315021	1.38E-25	1.668376	1.35E-31
P3h4	red	-0.7123161	4.38E-07	-0.5269142	0.00033319
Ctnnb1	red	-0.6905331	8.46E-10	-0.4549955	0.0001016
Psmd2	orange	0.58151303	1.06E-05	0.56293802	3.14E-05
Vars	orange	1.14215818	1.24E-14	1.0547309	1.93E-12
Vwa7	red	-0.7602383	0.00056823	-0.4078063	0.09360576
Slc44a4	red	-1.2796068	4.02E-08	-0.6644165	0.0065168
Ddah2	red	-0.7725031	3.57E-05	-0.6205622	0.0013622
Clic1	orange	1.51825831	7.99E-13	1.30998255	1.33E-09
Lsm2	orange	0.83723247	0.0002264	0.94585268	4.77E-05
Pole	yellow	2.79877326	6.25E-24	2.75945935	5.79E-23
Atp1a2	red	-1.0811511	8.78E-07	-0.9804665	1.39E-05
Casq1	skyblue	-1.9220242	3.70E-08	-1.1782183	0.00129991
Zfp775	red	-0.509405	0.00337113	-0.5839711	0.00098223
Scube2	skyblue	-2.5750721	2.22E-06	-1.8108521	0.00136111
M6pr	orange	0.60188858	3.95E-05	0.47479524	0.0017526
Tinf2	orange	0.58451867	3.59E-10	0.61650698	7.05E-11
Cav1	red	-1.0890916	7.42E-06	-1.0019667	5.89E-05
Bcl2l1	orange	1.455173	1.73E-18	0.93269567	5.51E-08
Dio2	orange	1.2319428	0.00988051	2.52731956	1.18E-07
Hnrnpa0	orange	0.47694278	0.00199301	0.64068205	3.85E-05
Ift43	red	-0.5983049	1.41E-08	-0.4665886	2.11E-05
Tcap	skyblue	-1.7428325	8.00E-06	-1.4496852	0.00032394
Crlf1	red	-1.4227223	1.25E-09	-0.6818943	0.00571612
Hmgcll1	red	-1.1165634	1.17E-10	-0.8641596	1.08E-06
Fzd3	skyblue	-1.358928	1.15E-06	-1.2594392	1.02E-05
Mid1ip1	red	-0.8358137	2.44E-09	-0.7407091	2.38E-07
Ap2s1	orange	1.00088566	2.04E-14	0.68022074	5.42E-07
Fgfrl1	red	-0.9147558	0.00030532	-0.7778949	0.00297336

Fhl2	red	-0.3946902	0.27020049	-0.9209074	0.00756585
Clstn3	skyblue	-3.4486704	7.73E-11	-2.7063528	7.83E-07
Fnbp4	orange	1.00111597	2.23E-16	0.91904902	9.10E-14
Cers4	red	-0.9438649	2.87E-10	-0.8010113	1.72E-07
Scrn3	skyblue	-1.4215574	3.08E-09	-1.1729368	1.83E-06
1700109H08	orange	0.91749124	0.00530109	0.9813464	0.0037562
Relt	yellow	1.92751497	2.04E-17	2.20133602	8.45E-22
Snrpb2	orange	0.92938826	1.62E-06	0.70273393	0.0005131
Ubc	orange	0.60177448	1.33E-06	0.40689006	0.00171004
Prpf31	orange	0.90844616	4.59E-08	0.79738607	3.01E-06
Sertad1	orange	1.17338263	1.52E-10	0.90134311	1.80E-06
Arpc5	orange	1.03582862	2.38E-08	0.82563648	1.60E-05
Pou2f2	yellow	2.21101839	2.41E-12	2.07558204	1.00E-10
Mgst1	red	-1.0280003	1.90E-06	-0.697274	0.00194532
Nfib	red	-0.6465503	1.89E-05	-0.7116881	3.68E-06
Rab25	red	-0.6821699	0.00062742	-0.4336972	0.04043511
Rbfox1	skyblue	-1.3682055	0.00104775	-1.4405306	0.00089762
Rps18	orange	0.69062166	2.61E-08	0.6273019	7.68E-07
Rps15a	orange	0.69230017	3.55E-08	0.638788	6.51E-07
Acyp1	red	-0.7894536	0.00122065	-0.7890825	0.00173272
Hdac5	red	-0.6858916	5.36E-08	-0.6056216	2.75E-06
Bmp7	red	-0.8796454	0.00298908	-0.0193217	0.96091109
Rsph14	skyblue	-1.6761077	8.69E-06	-1.0352976	0.00667913
Tbx1	red	-1.0196139	3.89E-05	-0.7149712	0.00575599
2610028H24	skyblue	-1.5800232	3.91E-09	-0.7361707	0.00924512
Dqx1	red	-0.8388125	0.00073289	-0.7370762	0.00376711
Ccl8	black	5.57912962	2.08E-62	5.90746372	6.68E-58
Tmem8c	yellow	2.92294863	0.00391944	3.34885549	0.00075327
Trpm2	yellow	4.40778166	1.33E-23	3.66920615	8.58E-17
Met	red	-1.0588691	5.84E-09	-0.5958704	0.00179416
Slc16a12	skyblue	-1.6875234	6.37E-08	-1.1023941	0.00052038
Syn2	red	-0.7433081	0.0050472	-0.7714755	0.00491328
Myod1	skyblue	-1.6050856	0.00910399	-1.1634216	0.08677743
Kcnq1	skyblue	-1.3942052	2.15E-11	-1.1278739	1.15E-07
Tor2a	orange	0.82401164	1.28E-07	0.6174729	0.00013137
Apobec3	yellow	2.18573663	1.14E-108	1.92112062	6.16E-84
Tex15	red	-1.3745431	3.38E-05	-0.6147954	0.08939315
G0s2	red	-1.2119558	3.08E-11	-0.802239	2.10E-05
Tex11	skyblue	-2.6067096	7.73E-06	-1.8980245	0.0011926
Fxyd5	yellow	2.46144638	5.38E-27	2.11831271	4.34E-20
Tfcp2	red	-0.6629079	3.35E-05	-0.4856234	0.00343852
Nuak2	yellow	2.37505134	2.81E-36	2.02603266	1.19E-26
Cox4i2	red	-0.7929839	9.45E-06	-0.8551324	2.71E-06

Snap47	red	-0.6229169	2.31E-06	-0.731541	4.04E-08
Wnt3a	skyblue	-2.2469952	4.12E-09	-1.5634205	7.65E-05
Kif19a	red	-1.4346221	2.38E-13	-0.6371774	0.00159781
Zmynd10	skyblue	-1.6765708	2.92E-10	-0.7617189	0.00599553
Slc38a3	skyblue	-1.8649718	7.88E-09	-1.567314	4.24E-06
Rassf1	orange	0.86091077	1.21E-06	0.80852615	8.21E-06
Rnf112	skyblue	-3.2195234	0.00077501	-1.6242879	0.09546606
Slc47a1	skyblue	-1.7470846	0.00044315	-1.4957422	0.00353992
Pifo	skyblue	-1.9568023	1.07E-14	-1.5343636	1.80E-09
Tnfrsf13b	orange	1.20963595	2.66E-06	1.03418169	0.00010311
AI597479	red	-0.6226528	4.07E-08	-0.4407698	0.00019387
Tmem86a	orange	1.00635426	0.00382504	0.60299044	0.10559857
Optc	skyblue	-3.010936	1.08E-07	-2.0867077	0.00019153
Ifi35	orange	1.62680867	6.43E-33	1.45832843	2.37E-26
Rdm1	skyblue	-1.2738675	1.64E-07	-1.2664578	3.33E-07
Mrpl52	orange	0.86551021	4.26E-06	0.88404836	4.55E-06
Ebf3	skyblue	-1.7009655	0.00361302	-0.8099884	0.20013377
Uckl1os	red	-1.4054416	2.45E-06	-0.9218415	0.00235985
Apol7a	yellow	2.91116898	1.37E-16	3.14641989	1.47E-19
Psen2	orange	1.22817786	4.80E-08	0.69343901	0.00335341
Acaa1b	skyblue	-1.8789505	5.01E-07	-1.5657628	4.18E-05
Fads1	red	-0.6837731	6.28E-12	-0.4835319	2.56E-06
Lmbr1	red	-0.745343	1.35E-06	-0.4233818	0.00874184
Tnfrsf22	red	-0.7921418	4.05E-05	-0.5990878	0.00279067
Cars	orange	0.59284075	4.82E-09	0.64201922	3.74E-10
Wnt2	red	-1.3462486	4.26E-09	-1.1069121	2.76E-06
Grid2ip	skyblue	-1.3823918	0.00993075	-1.2412934	0.02417584
Vac14	orange	0.6747288	1.18E-05	0.56565731	0.00038508
Mcoln2	orange	1.98769458	5.81E-07	1.94115935	1.39E-06
Tbrg1	orange	0.75187742	9.59E-10	0.71611154	9.73E-09
1700026D08	skyblue	-2.0387589	1.16E-11	-1.4696129	1.50E-06
Odc1	orange	0.89902955	3.65E-07	0.91558294	3.57E-07
Thg1l	orange	0.69014637	0.00163922	0.61243359	0.00711832
Adam19	orange	1.1094896	5.14E-06	0.99659052	6.82E-05
Exoc3l2	orange	1.15239809	0.00289482	0.88590964	0.02957005
Zfp296	orange	0.84876723	0.00321835	0.46641026	0.14455211
Plin5	skyblue	-1.1577322	0.00013314	-1.3713162	1.14E-05
Dhdh	red	-1.1788401	2.08E-13	-1.0772609	3.85E-11
Zfp790	red	-0.6571795	6.49E-06	-0.5879203	8.46E-05
Cpb1	skyblue	-2.1871554	0.00313996	0.13258702	0.88354444
Pinlyp	red	-1.0155093	0.00708528	-0.5889527	0.14820569
Fuz	red	-0.5631705	0.00043705	-0.5999398	0.00025281
Evi5	red	-0.6539109	3.37E-06	-0.6089497	2.44E-05

Git1	orange	0.67436995	7.80E-05	0.57790079	0.00103415
Scarf2	red	-0.6794961	0.00081956	-0.3967077	0.06711942
Med15	orange	0.70371577	1.45E-05	0.57038199	0.00067517
Aim1l	red	-0.8873727	8.08E-05	-0.8896597	0.0001121
Ehf	red	-0.7088015	0.00343133	-0.4408224	0.08756236
Tmem167	orange	0.81702937	8.93E-08	0.69013805	1.20E-05
Kif11	yellow	3.07724319	1.89E-18	3.60431246	1.45E-23
Mlkl	yellow	3.29323542	2.92E-31	3.2343938	4.30E-30
Ttll5	red	-0.7520154	4.01E-05	-0.5533626	0.00344698
Retn	skyblue	-3.4333667	5.44E-08	-1.4602609	0.02603587
Cdh23	orange	1.12963872	0.00610173	1.35556708	0.00157139
Podnl1	yellow	3.00353596	2.88E-18	2.49671608	6.06E-13
Adgrl1	red	-0.7937719	2.39E-13	-0.7402085	1.47E-11
Etv5	red	-1.3809244	4.59E-09	-0.6965656	0.00512895
Ptprs	red	-0.7177196	1.71E-06	-0.663625	1.58E-05
Fer1l4	red	-1.3818716	0.00139231	-0.7670784	0.04975381
Zfp651	red	-0.900173	2.59E-07	-0.6827726	0.00016175
Bcas1	skyblue	-1.5208064	2.39E-08	-1.2429834	6.47E-06
Snx31	skyblue	-2.3460407	0.00983461	-1.0230194	0.22021588
Cad	orange	0.65458009	0.00081643	0.53642669	0.00823969
Lypd8	black	6.00009854	4.04E-05	3.82666514	1.71E-05
Timm23	orange	0.65091634	0.00280186	0.5605344	0.01316017
Tnfaip8l2	yellow	2.50548451	1.75E-20	2.27549733	6.84E-17
Ly6g6e	red	-1.1362436	0.00866942	-0.8277164	0.07324777
St3gal1	orange	0.62483161	6.32E-05	0.41439059	0.01137191
Clip3	red	-0.933386	2.40E-14	-0.8681761	1.35E-12
Tctex1d2	orange	0.66660623	0.00167705	0.45284891	0.04538179
Zfp385c	skyblue	-1.631575	2.49E-05	-1.3237126	0.00138941
Glis2	red	-1.0092706	1.45E-19	-0.5729069	8.01E-07
Bicc1	red	-0.7766252	7.88E-10	-0.6439402	7.18E-07
Ankrd28	orange	0.58169017	0.00039129	0.66852835	5.94E-05
Klra17	black	5.66150287	0.00018179	1.44371556	0.16566181
Csf1	orange	1.05617862	1.54E-12	1.16291223	7.07E-15
Chrne	orange	1.97958135	0.00343671	1.39585045	0.04878127
Chfr	orange	0.71424315	9.97E-16	0.70526734	4.83E-15
Ceacam16	black	5.42255002	7.51E-13	3.98731917	3.29E-09
Adam28	skyblue	-1.6953806	1.16E-07	-0.7335222	0.0302225
Psmb1	orange	0.90969863	3.44E-13	0.63600105	9.14E-07
Nol3	red	-0.6266252	0.00139319	-0.2895356	0.17928083
Plekhg4	yellow	2.77415633	8.40E-09	1.80743483	0.00013507
Tppp3	skyblue	-2.3665172	2.82E-21	-1.7903636	2.12E-12
E2f4	orange	0.72337979	2.01E-09	0.55388898	8.41E-06
Surf4	orange	0.67488739	3.18E-05	0.5810194	0.00052044

Yes1	red	-0.8736163	3.32E-07	-0.7165477	5.08E-05
Galns	orange	1.31623846	1.43E-07	1.23505714	1.38E-06
Entpd2	red	-1.0364407	5.76E-07	-0.8389177	9.51E-05
Aldh1a3	yellow	1.98817791	4.19E-11	2.2259749	1.00E-13
Actn1	orange	0.73192261	0.00034571	0.82366724	7.54E-05
Nolc1	orange	0.69384526	1.97E-05	0.53294573	0.00156425
Casd1	red	-0.7656731	6.05E-06	-0.7271784	2.86E-05
Cyp2j9	red	-1.0030785	0.00178843	-0.7680002	0.02299474
Nipsnap3b	orange	0.76108367	2.48E-05	0.51877025	0.00621304
Gadd45b	orange	0.6111845	4.71E-08	0.50460979	1.20E-05
Slamf6	yellow	2.5560734	5.11E-19	3.07067519	9.10E-26
Slamf1	yellow	2.24052497	7.12E-13	1.99414988	4.24E-10
Zdhhc12	orange	0.62964939	8.13E-05	0.53929789	0.00107709
Endog	red	-1.0674517	1.12E-07	-0.8464583	4.77E-05
Cybb	yellow	2.18943997	9.60E-10	1.72777606	2.74E-06
Pcolce2	skyblue	-2.272204	8.66E-10	-1.756073	4.18E-06
Cd48	yellow	2.52281542	1.15E-24	2.60540486	1.73E-25
Dennd6b	orange	0.81294959	0.00014683	0.70352364	0.00142347
Ace2	red	-1.4183695	0.00022229	-1.0017102	0.0126004
Gzmb	black	8.11729481	2.29E-174	7.34978131	1.38E-145
C4a	orange	1.32007407	2.77E-06	1.01043117	0.00052583
Notch4	red	-0.6228404	0.00116589	-0.6095978	0.00194153
Lpl	red	-0.394915	0.09548551	-0.7420938	0.00140272
Atp6v0e	orange	0.5949828	8.30E-06	0.35995666	0.01059648
Gata5	red	-0.7852369	0.0014092	-0.5123834	0.05135471
Lama5	orange	0.47036508	0.01407994	0.61015625	0.00157427
Steap1	orange	1.63454995	0.00056993	1.37105311	0.00509373
Steap2	orange	1.08977561	1.22E-09	0.76022861	4.28E-05
Psma2	orange	1.03241341	8.55E-12	0.86672441	2.17E-08
Capza2	orange	0.9107194	2.36E-06	0.75770691	0.00014253
Plekho1	orange	0.80375069	3.46E-05	0.45267179	0.02804612
Rxrg	skyblue	-1.7039428	0.00033694	-0.9464511	0.06418404
Rxra	red	-0.7002944	3.37E-07	-0.5947996	2.49E-05
Fcrls	skyblue	-3.5507154	1.09E-46	-2.3969244	1.98E-22
Ncapg	yellow	3.84103152	3.10E-33	4.13989861	3.12E-32
H2afy	orange	0.94864351	2.35E-09	0.87339114	7.28E-08
Gatsl2	red	-0.6135401	2.96E-07	-0.3162066	0.01293568
Fcgr1	black	5.99585467	9.66E-27	5.76012717	1.38E-24
Ncf1	orange	2.00683103	1.49E-11	1.63090328	9.06E-08
Wnt11	red	-0.9935327	2.71E-12	-0.6010059	4.07E-05
Chdh	red	-1.6615729	0.00044376	-0.2949519	0.59493584
Stk32c	orange	1.74405811	4.08E-08	1.43955363	1.25E-05
Fli1	orange	0.58220772	0.00054146	0.6189358	0.00033184

Stard13	red	-0.7599813	3.21E-06	-0.5001705	0.00335334
H2-M3	orange	1.88747941	8.66E-30	1.69865888	4.74E-24
Ctsz	orange	1.79505659	1.46E-10	1.50398396	1.57E-07
Slmo2	orange	0.92532686	6.26E-09	0.79955711	1.05E-06
Sertad4	red	-1.3089826	1.65E-06	-1.1392521	5.92E-05
Slc25a5	orange	0.92815871	1.55E-11	0.76219622	6.41E-08
Eef1a2	red	-1.2095276	4.81E-06	-1.1317175	3.07E-05
Col20a1	red	-1.0371445	0.0002145	-0.5798842	0.05086006
Mpped2	red	-1.3011258	0.00023068	-1.0539618	0.00421747
E2f3	orange	0.75872246	2.28E-06	0.74121752	8.62E-06
Cd274	black	4.5924278	5.06E-210	4.60492984	7.21E-214
Pdcd1lg2	black	4.37182205	5.30E-24	5.3584594	7.95E-21
Il10	black	6.19473926	6.66E-22	5.74410153	1.23E-22
Foxred2	orange	1.63429004	3.59E-10	0.79581767	0.0027981
Eif3d	orange	0.78491334	6.98E-11	0.6519232	1.36E-07
H3f3b	orange	0.76095866	6.39E-09	0.69285622	2.23E-07
Nup50	orange	0.72028173	2.92E-06	0.46052328	0.00431352
Cmah	orange	0.29564328	0.06533692	0.76129971	1.36E-06
Scube1	red	-0.6436864	0.00025862	-1.0014463	1.24E-08
Sulf1	red	-1.2031751	7.53E-12	-0.8414156	3.53E-06
Matn4	skyblue	-1.7845442	4.28E-08	-1.1493315	0.0007174
Rbpjl	skyblue	-1.682425	8.35E-08	-1.0152345	0.00198396
Sdc4	orange	0.9363847	0.00012456	0.93841403	0.00017376
Il13ra1	orange	0.90710589	0.0006858	0.64296591	0.02172892
Nbr1	red	-0.6329527	8.60E-05	-0.50471	0.00251416
Cyth1	orange	0.73065663	1.65E-07	0.48863011	0.00078501
Rnd3	orange	0.96476764	0.0004105	0.95474303	0.00065789
Brca1	yellow	3.1407759	1.22E-24	3.00243148	7.30E-22
Cntnap1	skyblue	-1.7213185	2.57E-08	-2.0579009	5.29E-11
Psmd3	orange	0.87281782	2.50E-10	0.75104061	1.02E-07
Cd300lg	skyblue	-2.0675294	0.00082409	-1.9105273	0.00267105
Mpp2	red	-0.5691309	0.00627263	-0.7260569	0.00056954
Vtn	skyblue	-2.2350371	3.41E-09	-1.5370072	8.61E-05
Rpl19	orange	0.60573746	1.10E-06	0.46661771	0.00028922
Timp2	red	-0.9083791	1.77E-14	-0.6789521	2.46E-08
Rarb	skyblue	-1.6286961	5.09E-09	-1.0371174	0.00022212
Cdc6	yellow	2.3717837	6.67E-21	1.95526486	2.03E-14
Atad5	orange	1.70391527	1.88E-14	1.40554764	6.08E-10
Abr	orange	0.66684284	8.03E-06	0.55025494	0.00035979
Rab11fip4	orange	1.20496166	8.50E-15	1.10731524	1.94E-12
Cd40	orange	1.5454023	2.65E-19	1.48387885	1.11E-17
Slc35c2	orange	0.65446023	0.00212733	0.58204061	0.00835184
Zfp334	red	-0.4424973	0.01435791	-0.6038454	0.00086404

Wsb1	orange	0.90145649	2.39E-06	1.00670806	2.04E-07
Ttpal	orange	0.70886676	4.88E-15	0.23604657	0.01527019
Serinc3	orange	0.80908392	4.32E-13	0.42183415	0.00033098
Pgs1	orange	1.02416659	2.27E-08	0.9920343	1.05E-07
Birc5	yellow	3.1203825	3.58E-15	3.3580936	9.33E-17
Afmid	orange	1.14387655	0.00142236	0.53324341	0.17098543
Wfdc2	red	-0.936165	2.63E-08	-0.5288274	0.00277669
Etv4	orange	1.48081524	0.00070093	1.8980032	2.58E-05
Mmp9	orange	1.02502219	0.0178753	1.23692519	0.00451485
Slc12a5	skyblue	-1.4009239	3.28E-07	-1.4050658	5.23E-07
Pltp	skyblue	-1.7036657	1.04E-15	-1.3379377	7.18E-10
Ctsa	orange	0.65590005	1.05E-05	0.4252861	0.00639483
Pitpna	orange	0.58598573	0.00071498	0.54658181	0.00216128
Jph2	red	-1.4012783	7.18E-12	-0.9483671	7.80E-06
Dhx58	yellow	3.80595916	8.23E-49	3.75034241	2.41E-47
Hspb9	yellow	2.59668487	1.82E-07	2.67763788	5.46E-07
Mybl2	yellow	2.44576143	8.66E-17	2.36374708	1.37E-15
B4galt5	orange	1.51061778	1.44E-12	1.28140098	3.89E-09
Cadps2	red	-1.3044553	2.25E-23	-0.8504178	2.11E-10
Ddx27	orange	0.66239318	9.13E-06	0.58736165	0.00013184
Cyth3	red	-0.8196607	9.33E-07	-0.6658528	0.0001123
Cyth4	yellow	2.63076149	9.80E-19	2.60811421	2.73E-18
Rac3	skyblue	-1.8568126	4.46E-08	-1.2865923	0.00025353
Hist1h2bc	red	-0.9803651	6.70E-08	-0.8055581	1.82E-05
Baiap2l2	skyblue	-1.7014608	0.0019524	-1.9745993	0.00071136
Mafk	orange	0.89395458	1.21E-07	0.84802635	8.65E-07
Erbb3	red	-0.8122366	0.00028872	-0.4798368	0.04363821
Ikzf3	yellow	3.05209836	5.19E-35	3.26074409	4.10E-39
Vmp1	orange	1.68090537	2.02E-13	1.52107858	5.54E-11
Uchl5	orange	0.90192128	1.51E-05	0.52167037	0.01831381
Trove2	red	-0.779132	1.68E-05	-0.6422261	0.00060806
Pmp22	red	-0.9389685	5.29E-07	-0.6155278	0.00162921
Psmb6	orange	0.89322012	5.90E-08	0.6726801	8.09E-05
Pfn1	orange	1.62227837	6.00E-18	1.36920418	7.17E-13
Gpx3	orange	0.58791733	0.00106595	0.36356565	0.05683334
Il12rb2	yellow	2.45545644	6.77E-08	3.69875789	5.84E-13
Kpna2	orange	1.25248622	5.02E-18	1.03584937	2.12E-12
Smurf2	red	-0.944056	5.15E-16	-0.6543856	4.90E-08
Cuedc1	red	-0.6745216	4.33E-07	-0.4231659	0.00247729
Abi3	orange	0.63341695	1.57E-05	0.4956419	0.00114952
Shroom1	red	-1.4204712	1.16E-05	-0.801272	0.01641569
Kif3a	red	-0.6807974	5.86E-05	-0.7315366	2.53E-05
Sept8	red	-0.5895426	1.41E-07	-0.4789838	3.29E-05

Mapt	skyblue	-1.3939292	1.32E-11	-1.2219331	5.89E-09
Gid4	red	-1.3710655	7.84E-13	-1.1049884	1.70E-08
Myo1b	red	-1.3150891	4.29E-13	-1.0760647	6.64E-09
Ypel2	red	-1.0381106	3.84E-06	-0.6522758	0.00564123
Akap1	red	-0.810944	1.35E-14	-0.4394416	6.63E-05
C1qbp	orange	0.64231807	0.00067901	0.65054174	0.00081826
6330403K07I	skyblue	-1.3775818	2.14E-06	-1.1511719	0.00019313
Slc13a3	orange	1.05584523	0.00918925	0.56241021	0.2038109
Chd3	red	-0.7175084	3.86E-12	-0.4394002	4.55E-05
Wnt9b	orange	1.76415764	0.00533573	1.09194777	0.10377541
Adora2b	orange	0.52046402	0.02079103	0.65456916	0.00437556
Trpv2	orange	1.87614533	1.29E-19	1.64987643	3.67E-15
Cenpv	red	-0.5060084	0.0235158	-0.6171591	0.00549536
Trim37	red	-0.5943062	5.43E-09	-0.6094679	2.97E-09
Ybx2	skyblue	-1.9423955	5.21E-08	-1.3025616	0.0003713
Slc2a4	skyblue	-1.74977	2.53E-07	-1.4744361	2.45E-05
Dnah11	skyblue	-2.425973	2.49E-16	-1.2881938	2.00E-05
Atox1	orange	0.63692511	1.98E-06	0.5793465	2.61E-05
Tbx3	red	-1.1070259	3.57E-07	-1.0278853	3.82E-06
Dusp14	red	-1.1054092	8.26E-08	-0.8185198	0.0001279
Ikzf1	yellow	2.3551074	2.49E-33	2.54435674	1.52E-38
Copz2	red	-0.685888	4.65E-05	-0.4037183	0.02387607
Aatf	orange	0.61064236	0.00161326	0.78849492	6.09E-05
Slc25a35	red	-1.125318	3.43E-07	-0.8209096	0.00030377
Zbtb4	red	-1.0932783	3.92E-12	-0.8220419	3.96E-07
Mpdu1	orange	0.63547025	0.00029971	0.43664392	0.01819879
Cd68	yellow	2.72362212	4.17E-12	2.4035643	1.87E-09
Slc35g3	skyblue	-2.7462638	1.52E-06	-2.5393026	6.99E-06
Acsl1	skyblue	-1.5718329	1.88E-09	-1.3543398	4.18E-07
Abca5	red	-1.1418715	0.00065823	-0.7309645	0.03905287
Lsp1	orange	1.48626366	1.44E-15	1.28762458	1.03E-11
Sfrp5	skyblue	-1.1852715	0.02597384	-1.4548168	0.00688925
Myh11	red	-1.4513404	7.01E-16	-0.6395293	0.00078964
Unc45b	red	-0.8571411	1.47E-05	-0.7525467	0.00022863
Rars	orange	0.947443	1.03E-09	0.85544	6.90E-08
Wwc1	red	-1.1632059	3.72E-12	-0.6699094	0.00013122
Pnpla5	skyblue	-4.0106798	0.00277516	-0.5502616	0.67847563
Mrpl45	orange	0.82104864	1.20E-07	0.42236695	0.01086707
Mb	skyblue	-1.1127183	7.33E-06	-1.457134	5.07E-09
Irf1	yellow	2.2716982	3.68E-102	2.25454949	1.23E-100
Slc22a5	red	-0.5489639	0.00946126	-0.591044	0.00611305
Alox12e	yellow	4.11110519	2.16E-06	4.19568534	1.39E-06
Cxcl16	yellow	2.15630656	4.35E-15	1.83679573	5.17E-11

Med11	orange	0.71543303	1.75E-05	0.52903753	0.00244874
Alox15	orange	0.61299939	0.19409486	1.73432596	0.00012221
Heatr9	yellow	2.26529708	1.06E-05	2.40057662	6.06E-06
Ccl4	black	7.72705814	1.09E-57	6.48000458	3.67E-68
Natd1	red	-0.6346772	0.0012432	-0.4637645	0.02403674
E2f2	orange	0.53382778	4.63E-05	0.58035511	1.47E-05
Slfn3	yellow	2.76005863	2.56E-12	2.92371158	1.76E-13
Dnah1	skyblue	-2.133496	8.65E-09	-1.2112408	0.00148407
Dalrd3	red	-0.7818852	3.44E-06	-0.5278889	0.00262399
Rab3d	red	-0.590746	0.00016365	-0.4524414	0.00550002
Slc25a22	orange	1.31804984	4.79E-19	1.21346897	3.60E-16
Ccl9	orange	1.68963306	7.02E-06	1.61135356	2.83E-05
Scrn1	red	-1.3328928	2.89E-07	-0.9597912	0.00035935
Tmem160	orange	0.87434566	0.00024958	0.74921613	0.00258872
Rab5c	orange	1.07618241	2.53E-09	0.99261018	6.90E-08
Styxl1	skyblue	-1.6242927	0.00098498	-0.9727149	0.05348257
H13	orange	0.74748618	1.79E-08	0.80137006	2.41E-09
Scn1b	red	-1.3808935	7.83E-10	-0.7976477	0.00072088
Chtf18	orange	1.73875049	3.26E-10	1.88055674	2.55E-11
Ahr	red	-1.0049731	1.94E-07	-0.7185292	0.00033229
Nop9	orange	0.90180386	4.87E-16	0.73192165	1.21E-10
Grb7	red	-1.4282341	1.30E-08	-1.0644299	4.17E-05
Aoc3	skyblue	-2.7348818	8.02E-17	-1.9759014	2.99E-09
Gdpd2	orange	0.4257243	0.24637488	0.94520076	0.00653342
Tlcd1	red	-0.994991	6.55E-06	-0.7330658	0.00132991
Arhgef25	red	-0.9107072	1.73E-08	-0.7978057	1.39E-06
Arid3a	orange	1.09834781	4.04E-06	0.89560866	0.00027277
Mydgf	orange	0.62683213	0.0018676	0.52535422	0.01210618
Sema6a	skyblue	-1.4543342	2.00E-06	-1.1579286	0.00025151
Ccdc12	orange	0.64822648	7.39E-07	0.50045395	0.00024087
L3hypdh	red	-0.8887136	7.29E-05	-0.8504343	0.00026982
Lyst	orange	0.42386716	0.0010497	0.58048278	8.37E-06
Calr3	red	-1.2232886	0.00099279	-0.9112033	0.01985878
Iyd	red	-1.7247007	3.56E-05	-0.4972598	0.2603872
Ccdc170	skyblue	-2.3323032	1.82E-19	-1.008549	4.16E-05
Fbxo5	orange	1.45792261	1.85E-24	1.23909555	4.23E-17
Trdn	red	-0.9487697	0.00558777	-1.2441814	0.00036691
Stxbp5	orange	0.97091766	3.49E-11	0.89687573	1.56E-09
Katna1	orange	0.99033226	5.98E-12	0.84611932	1.00E-08
Adat2	orange	0.90088172	7.28E-05	0.91429052	8.42E-05
Ltv1	orange	0.83976758	2.49E-05	0.70622529	0.00061173
Plagl1	red	-1.2024027	3.20E-09	-0.9293657	9.04E-06
Utrn	red	-0.8097419	3.10E-09	-0.6475591	4.07E-06

Mical1	orange	1.15614838	3.26E-10	1.0442666	2.51E-08
Wasf1	skyblue	-1.1070773	0.00376979	-1.5234239	7.68E-05
Rab32	orange	1.32761266	6.07E-06	1.40266999	2.84E-06
Fyn	orange	0.69680544	4.14E-07	0.69660938	6.96E-07
Tube1	orange	1.05764778	0.05616682	2.09378047	0.00013593
Popdc3	red	-0.5995476	1.13E-05	-0.4026661	0.00549357
Tnfaip3	yellow	2.31649424	7.40E-30	2.085308	2.54E-24
Perp	red	-0.5858062	0.05067841	-0.802255	0.0076288
Hebp2	red	-0.4285114	0.15565868	-0.8136998	0.0064885
Fam184a	red	-1.1501873	1.06E-05	-1.028611	0.00011759
Qrsl1	orange	0.52658964	0.00489547	0.63080193	0.00109536
Aim1	orange	1.46894173	2.39E-14	1.26756707	9.33E-11
Vta1	orange	0.75929297	0.00068168	0.5048332	0.03246
Smpdl3a	red	-0.5971164	2.47E-05	-0.5895691	4.68E-05
Pkib	yellow	2.52770596	7.28E-11	1.90157997	1.58E-06
Rspo3	orange	1.57311856	0.00447976	1.81049445	0.00182277
Echdc1	red	-0.882677	0.00266565	-0.7896706	0.00902243
Slc6a15	skyblue	-2.6813458	8.97E-09	-2.2808888	1.26E-06
Lama2	red	-0.9254909	0.00016111	-0.5222205	0.04559019
Gprc6a	skyblue	-1.6141348	0.00073607	-0.9648976	0.05562189
Fam162b	skyblue	-2.1464051	1.01E-09	-1.618549	7.76E-06
P4ha1	red	-0.5930819	5.11E-11	-0.6180106	1.54E-11
Zwint	orange	0.69470072	0.00014368	0.53515463	0.00486039
Dcn	red	-1.1279534	3.12E-07	-0.7932571	0.00054434
Mrln	red	-1.4217091	0.00317859	-0.0814267	0.89505588
Cdk1	yellow	2.80820711	2.67E-24	3.75828674	4.13E-38
Rhobtb1	red	-0.9161282	1.04E-05	-1.1275857	7.27E-08
1700040L02F	skyblue	-2.0215174	1.01E-07	-1.4217923	0.00024387
Dusp6	orange	0.8438277	0.00017	0.76839775	0.00088164
Kitl	red	-0.9814712	8.51E-06	-0.7607119	0.00086573
Sgk1	orange	0.64238259	2.22E-07	0.2749987	0.03897522
Apaf1	orange	1.06602878	3.34E-15	0.91472063	2.89E-11
Arg1	black	5.02439117	6.73E-17	4.54693112	7.13E-14
Enpp3	skyblue	-1.6266545	9.46E-07	-1.2045981	0.00034298
Pde7b	red	-1.0963651	2.00E-10	-0.5239602	0.00432443
Mtfr2	yellow	3.14657234	2.37E-20	3.23486753	3.43E-19
Map7	red	-0.7500581	0.00014944	-0.4695022	0.02407771
Ctgf	orange	0.64591615	0.00355766	0.6838916	0.00250534
Vnn3	red	-1.4879395	3.20E-05	-0.4266107	0.27923497
Cfap54	skyblue	-2.243425	6.43E-15	-1.2951595	6.53E-06
Ntn4	skyblue	-1.6136872	2.66E-11	-1.1605349	3.48E-06
Usp44	skyblue	-1.6268028	0.00500155	-0.7905836	0.23211058
Nudt4	red	-0.6691216	1.73E-05	-0.8972257	9.01E-09

Nuak1	skyblue	-1.7283947	2.71E-10	-1.4702389	1.50E-07
Tcp11l2	red	-1.1778135	1.34E-05	-0.9847819	0.00042261
Timp3	orange	0.77105093	1.11E-08	0.54502308	9.87E-05
Ascl1	skyblue	-3.7491986	2.59E-05	-1.1719409	0.11043007
Igf1	orange	1.11532703	0.00044539	1.18271041	0.00028216
Slc5a8	skyblue	-6.0745439	9.82E-07	-3.2746029	1.15E-06
Mypn	red	-1.0929812	2.28E-06	-0.8424961	0.00047762
Hnrnph3	red	-0.7151779	6.14E-07	-0.4965978	0.00091978
Ddx21	orange	1.03717246	7.56E-08	0.96628425	9.47E-07
Srgn	orange	1.90117932	5.91E-16	1.62713597	9.44E-12
Ppa1	orange	1.91666722	4.52E-23	1.8636385	1.03E-21
Eif4ebp2	red	-0.7506011	1.53E-07	-0.6867679	2.69E-06
Sgpl1	orange	0.64823377	0.0003218	0.65846332	0.00036073
Pcbd1	red	-1.0479388	4.72E-05	-0.6844979	0.01268095
Slc29a3	orange	1.53055348	1.15E-06	1.31061392	5.13E-05
Slc16a7	skyblue	-2.2369173	3.10E-11	-1.4079386	4.97E-05
Lrig3	red	-0.8753102	5.52E-06	-0.4195036	0.04051185
Ddit4	orange	1.69907365	3.39E-07	0.72845599	0.04230973
Tbk1	orange	0.97128039	2.02E-14	0.80060604	6.49E-10
Pno1	orange	0.89015407	0.00018749	0.84191697	0.00061088
Plek	yellow	2.41260975	5.95E-16	2.01823581	2.93E-11
Avpr1a	skyblue	-1.6327662	0.00397082	-1.1534875	0.05231524
Vps54	orange	0.76481719	1.41E-09	0.5998642	4.00E-06
Pcsk4	red	-0.9348702	0.0004496	-0.4879623	0.08245111
Peli1	orange	0.65686028	2.57E-07	0.52797167	5.98E-05
Apc2	skyblue	-1.3556684	2.14E-07	-1.2882669	1.29E-06
Thap2	red	-0.8915996	1.95E-07	-0.8704981	5.85E-07
Slc1a4	red	-0.9335371	2.66E-09	-0.534829	0.00105441
Dock2	yellow	2.47238226	1.28E-22	2.37517596	8.41E-21
Gamt	skyblue	-1.3627903	6.07E-07	-1.5234026	7.70E-08
Actr2	orange	0.87426759	5.69E-07	0.70429035	9.58E-05
Ptprb	red	-1.158779	5.88E-07	-0.9515309	6.97E-05
Kcnmb1	red	-1.5081411	1.69E-10	-0.9539896	8.71E-05
Gabrp	skyblue	-1.9070565	5.80E-15	-0.9368197	0.00025379
Meis1	red	-1.2033903	7.79E-09	-0.8249469	0.00013786
Best3	skyblue	-2.0137226	1.24E-05	-0.4229407	0.41256379
Cobl	red	-0.8264181	2.92E-05	-0.6592907	0.00126808
Rab36	red	-0.7807876	1.83E-06	-0.481748	0.00454784
Adora2a	orange	1.7916193	6.33E-26	1.7018122	4.14E-23
Snrpd3	orange	0.63023363	0.00260372	0.49959846	0.02241468
Ddc	skyblue	-1.5757035	0.00018185	-1.7188603	6.88E-05
Cpm	skyblue	-2.1195646	9.62E-08	-1.4344354	0.00052581
E2f7	yellow	1.80654173	1.30E-10	2.27278222	4.90E-15

Phlda1	orange	1.02189254	5.96E-05	1.21459389	2.52E-06
Jsrp1	red	-1.187658	0.00016517	-0.7141545	0.03479706
Wif1	skyblue	-4.0522417	8.80E-14	-2.7457035	1.24E-07
Tmbim4	orange	0.69911921	7.66E-07	0.59725447	4.16E-05
Helb	orange	0.93215301	6.46E-12	0.81991968	3.71E-09
Prmt2	red	-0.7889717	3.64E-11	-0.4959403	6.85E-05
Fzr1	orange	0.63035801	3.22E-06	0.66750181	1.31E-06
Txnrd1	orange	0.74252923	2.26E-05	0.7830257	1.16E-05
Glt8d2	skyblue	-3.2657469	3.38E-16	-2.2017098	1.08E-08
Slc36a1	orange	0.70380742	0.00021446	0.54051622	0.00626408
Adarb1	red	-0.9401372	5.42E-06	-0.9414296	8.09E-06
Appl2	red	-1.0348894	9.36E-07	-0.7321418	0.00083339
Slc36a2	red	-0.9324381	0.00022996	-0.9229062	0.00037703
Hint1	orange	0.71877185	8.99E-07	0.46528889	0.00238699
Lyrm7	red	-0.3414895	0.30490242	-0.8448362	0.0084991
Stk10	orange	1.18127617	1.47E-07	1.1668721	3.51E-07
Rel	orange	1.281837	6.99E-10	1.16528266	4.20E-08
1700093K21I	yellow	0.31876556	0.73581625	2.84077542	0.00429329
Mpg	orange	0.66565598	2.82E-05	0.45549617	0.0059974
Nsg2	orange	0.43203658	0.47026587	1.8196399	0.00096939
Cdc34	orange	0.70740765	6.08E-05	0.56927478	0.00183915
Shc2	skyblue	-2.2226064	2.25E-17	-1.1692133	1.04E-05
Sptbn1	red	-0.8711198	1.04E-05	-0.6574266	0.00134273
Wdpcp	red	-0.9736794	1.37E-10	-0.741988	1.82E-06
Fstl3	orange	1.0817481	4.49E-06	1.25132073	1.43E-07
Nudcd2	orange	0.80843273	4.39E-05	0.51536718	0.01344426
Hmmr	yellow	3.31678963	6.33E-28	3.51684776	1.85E-27
Hcn2	red	-0.906826	0.00807747	-0.7546165	0.03945055
Zfp354b	skyblue	-1.3429729	1.63E-05	-1.3452072	2.62E-05
Cyfip2	orange	1.82842261	6.40E-34	1.6071308	2.98E-26
Hnrnpab	orange	0.7522464	0.00126373	0.97687712	3.42E-05
Gfpt2	orange	0.87874866	3.46E-05	0.97825895	5.75E-06
Zfp354a	red	-0.9347752	0.00114708	-0.8678365	0.00295649
Gnb2l1	orange	0.58819568	1.29E-07	0.50019303	1.25E-05
Il13	black	5.74821873	9.56E-05	2.3191206	0.0084676
Jade2	orange	0.62167976	1.99E-08	0.31855748	0.00642155
Cdkl3	orange	0.86246477	0.00021797	0.89389151	0.00016942
Kremen1	red	-0.7284294	3.37E-06	-0.2425757	0.16138401
Itk	yellow	2.55967704	7.89E-19	2.405104	1.34E-16
Nefh	yellow	3.24710416	2.44E-13	3.04574131	1.80E-13
Med7	orange	0.67169332	0.00071512	0.79998179	8.82E-05
Havcr2	yellow	3.74926452	1.28E-22	3.16553186	2.77E-16
Tnip1	orange	1.3756104	6.43E-13	1.22502106	2.96E-10

Fam71b	yellow	3.45491604	6.09E-07	3.92182063	1.42E-06
Upp1	yellow	2.53604164	4.43E-21	2.59051298	8.72E-22
Tns3	red	-0.7459515	6.42E-11	-0.4933731	3.17E-05
Igfbp3	skyblue	-1.3649469	2.32E-25	-1.2187163	1.91E-20
Pes1	orange	0.60381702	0.00058943	0.41755166	0.02385107
Adcy1	skyblue	-1.5191279	0.02351021	-1.7655027	0.00957316
Osbp2	skyblue	-1.6963775	0.03002868	-2.1416574	0.0066853
Myo1g	yellow	2.27600692	8.40E-32	2.15059232	2.43E-28
Smtn	red	-0.653385	0.00012709	-0.5284007	0.00277941
Arf5	orange	0.74381217	5.29E-06	0.5726686	0.00074247
Patz1	red	-0.6707452	3.03E-05	-0.3267495	0.05810767
Ogdh	red	-0.5962631	2.88E-06	-0.5198159	7.37E-05
Rps27a	orange	0.65668463	4.94E-07	0.50440445	0.00019338
Pnpt1	orange	1.03226683	8.47E-14	1.01748916	2.98E-13
Efemp1	skyblue	-1.5441403	4.86E-06	-1.0550518	0.00275197
Myl7	skyblue	-1.5803056	0.07308532	-2.3945308	0.00635518
Pold2	orange	0.68417799	6.11E-05	0.45472863	0.01112121
Aebp1	red	-0.8095185	5.27E-08	-0.4232593	0.0069959
Pgam2	skyblue	-1.7908524	1.16E-08	-1.3172919	5.24E-05
Dbnl	orange	0.95822863	6.39E-14	0.90843583	1.98E-12
Dynll2	red	-1.1025714	1.45E-17	-0.7930075	2.42E-09
Sept4	red	-0.9370337	5.44E-06	-0.7497649	0.00043808
Prr11	yellow	3.00635604	3.50E-22	3.01983289	3.20E-21
Tubd1	red	-0.3694042	0.06837476	-0.6558111	0.00102179
Acaca	red	-0.7602519	0.00957567	-1.0612126	0.00031792
Shmt1	orange	0.71614439	0.0008586	0.80608617	0.00023935
Tom1l1	red	-0.7667675	0.00070582	-0.2977125	0.23432108
Myocd	red	-1.0578015	0.00451015	-1.0136796	0.00699169
Cox11	orange	0.33396594	0.27020529	0.83289076	0.00451562
Stxbp4	skyblue	-1.5018543	2.81E-09	-1.2004136	2.16E-06
Efcab10	skyblue	-2.7908684	8.99E-06	-1.6852148	0.00665942
Atxn7l1	orange	0.74321712	9.64E-07	0.61225647	8.80E-05
Atp6v1c2	red	-1.5415859	6.36E-06	-0.9774569	0.00626378
Sypl	red	-0.572531	7.86E-07	-0.5842273	7.53E-07
Nampt	orange	1.70296664	6.14E-35	1.25356609	3.16E-19
Pik3cg	yellow	2.75937433	1.76E-26	2.03292993	7.26E-15
Tspan13	skyblue	-1.3704554	2.88E-09	-1.1824651	5.66E-07
Agr2	orange	0.01891873	0.96882077	1.60188068	4.41E-05
Lpin1	red	-0.91106	0.00727305	-1.0643055	0.00198861
Rgs9	skyblue	-1.8182835	1.19E-06	-1.2322571	0.00145995
Hs1bp3	red	-0.7195343	8.83E-08	-0.4435425	0.00157477
Fam84a	skyblue	-1.7220744	2.22E-30	-0.8839657	4.02E-09
Gna13	orange	1.19482945	4.35E-13	1.13209247	1.16E-11

Abca8b	red	-1.4369124	1.04E-09	-0.985271	5.52E-05
Map2k6	red	-1.0481278	9.02E-05	-1.2278701	8.15E-06
Klhl29	red	-0.5780553	0.04256182	-0.7423708	0.00919383
Adi1	red	-1.0714082	2.68E-13	-0.9580274	1.12E-10
Fkbp1b	orange	1.31429755	2.95E-06	1.04878317	0.00032302
Cmpk2	yellow	2.6295269	1.85E-37	2.66780921	1.68E-38
Rsad2	black	5.82808577	4.27E-64	5.45456007	2.44E-56
Rnf144a	skyblue	-1.7611913	4.70E-17	-0.9820607	7.47E-06
Id2	orange	1.41984114	6.94E-18	1.03571383	9.84E-10
Mboat2	red	-0.6999059	0.00097324	-0.7405773	0.0006704
Rrm2	yellow	2.97737961	4.20E-27	3.41865805	3.50E-34
Bcap29	orange	0.86442263	0.00039683	0.40673246	0.12679278
Slc26a4	yellow	3.49058307	1.35E-27	3.93736538	4.73E-33
Cenpo	orange	0.76429922	5.29E-05	0.58781952	0.00303962
Klf11	red	-0.7511798	3.06E-06	-0.4586702	0.00666795
Adcy3	red	-0.599408	0.00019366	-0.5010685	0.00271117
Efr3b	orange	0.82611098	0.00426493	1.07278325	0.00024315
Kif3c	red	-0.7961863	5.36E-12	-0.8084308	5.53E-12
Pxdn	red	-0.9701171	9.12E-14	-0.8191947	6.62E-10
Ccl11	orange	1.18838675	1.62E-06	1.58418222	1.59E-10
Hnf1b	red	-0.8997325	3.27E-06	-0.6376144	0.00132854
Ace	red	-0.4905314	3.25E-05	-0.6417503	6.38E-08
Mmp28	skyblue	-1.7428435	5.78E-09	-1.3195289	1.88E-05
Rasl10b	yellow	2.48444133	6.25E-09	1.56498621	0.00041539
Gas2l2	skyblue	-1.6475379	2.50E-07	-0.879502	0.0062221
Itgb3	orange	0.93808193	7.98E-05	0.85870434	0.00045706
Nle1	orange	0.85230756	0.00033701	0.90449353	0.00019601
Mrc2	red	-0.6692106	0.00820882	-0.4931352	0.06409948
Ccl1	black	8.48516845	3.85E-10	7.1445923	2.93E-07
Asic2	yellow	3.31551012	6.66E-05	2.75486996	0.00160296
Ftsj3	orange	0.81839458	2.02E-06	0.76015115	1.69E-05
Psmc5	orange	0.61259802	7.42E-06	0.45719963	0.00129459
Adap2	orange	1.18743809	7.93E-08	0.96341613	2.29E-05
Psmd12	orange	0.76663652	6.62E-07	0.63216794	7.16E-05
Cacng1	red	-1.1056862	2.84E-05	-0.6921711	0.01408352
Cep112	red	-0.7982737	6.05E-05	-0.7646753	0.00018053
Rab37	yellow	2.17630467	5.43E-07	2.01524325	6.04E-06
Slc9a3r1	orange	0.95826615	4.73E-09	0.75163188	8.28E-06
Nt5c	orange	0.79558114	4.71E-08	0.68658235	4.64E-06
Hn1	orange	1.22097622	6.72E-14	1.07427154	9.33E-11
Mif4gd	orange	0.88909783	5.57E-22	0.69043347	2.83E-13
2310067B10	red	-0.8181011	4.02E-08	-0.6270003	4.57E-05
Itgb4	red	-0.7711245	2.47E-10	-0.3373895	0.00892422

Aspa	skyblue	-1.5883007	7.84E-08	-1.1799695	8.23E-05
Fbf1	red	-0.6295765	1.95E-05	-0.5046128	0.00093085
Acox1	red	-0.5824988	0.00054858	-0.3794163	0.03278858
Camkk1	red	-1.2222801	8.60E-26	-0.5888821	1.01E-06
Atp2a3	skyblue	-1.5503895	5.02E-11	-1.2926161	8.73E-08
Ankfy1	orange	0.60141247	3.58E-07	0.62588999	1.87E-07
Spns3	orange	1.61297738	0.00470516	1.08702839	0.08090044
Tekt1	red	-1.0481737	2.23E-09	-0.9060601	3.44E-07
Rhbdf2	orange	1.21229866	4.38E-07	1.15587082	2.42E-06
Fam64a	yellow	3.07961971	4.70E-17	3.29309813	1.12E-17
Cygb	red	-0.8195906	1.30E-06	-0.5669005	0.00131453
Wscd1	skyblue	-2.4047478	2.99E-17	-1.9227514	3.20E-11
1810032O08	orange	0.95558221	3.48E-07	1.09516766	5.82E-09
Mxra7	red	-0.8476815	1.95E-06	-0.6869721	0.00019256
Nos2	yellow	2.07301706	3.99E-07	2.39469293	8.35E-09
Slc46a1	red	-0.6553702	0.00243979	-0.9174988	3.42E-05
Tmigd1	skyblue	-1.7242998	0.00253096	-0.7277195	0.25301268
Rph3al	red	-1.4546338	1.28E-19	-0.8723107	1.10E-07
Doc2b	yellow	3.45800304	1.60E-25	1.07516186	0.00128944
Cacna1g	skyblue	-1.3749408	0.01026509	-1.7531331	0.0013565
Lrrc59	orange	1.12938831	3.15E-06	1.12301891	5.60E-06
Slc35b1	orange	0.59135254	3.38E-06	0.54654904	2.82E-05
Scrn2	red	-0.6557557	1.61E-07	-0.4014202	0.00252962
Lrrc46	red	-1.4481579	1.01E-08	-0.7145894	0.00744123
Cacnb1	skyblue	-1.6620024	1.55E-14	-1.0203041	5.76E-06
Asgr1	skyblue	-3.6928568	8.89E-14	-3.4377982	9.01E-11
Nr1d1	skyblue	-2.3786861	1.00E-12	-1.5671008	5.93E-06
Tmem107	red	-1.0098835	1.54E-09	-0.8370862	9.55E-07
Aurkb	yellow	3.22753682	8.43E-24	3.70000994	1.85E-29
Myh10	red	-1.2705526	1.79E-09	-0.8348718	0.00014478
Pik3r5	yellow	2.42655688	6.79E-34	2.3140465	8.38E-31
Cfap52	skyblue	-1.5601668	6.29E-13	-0.9273746	2.54E-05
Krt19	orange	0.82052423	0.01054166	1.07297217	0.00090887
Top2a	yellow	3.15002794	1.11E-26	3.66567423	3.34E-35
Lsm12	orange	0.81858898	5.79E-07	0.6582216	0.00010426
Adam11	orange	0.62697642	0.00038431	0.14308555	0.48477605
Higd1b	skyblue	-1.6186573	1.32E-05	-1.5167234	6.77E-05
Ccdc103	skyblue	-1.5915892	5.66E-05	-0.7393967	0.07421833
Nmt1	orange	0.71331445	1.15E-09	0.52658247	1.41E-05
Map3k14	orange	1.21085338	1.94E-19	1.18454467	1.48E-18
Scfd1	orange	0.62251286	5.45E-07	0.52118978	4.69E-05
Pole2	orange	1.5325934	2.04E-06	1.26573371	0.0001802
L2hgdh	red	-0.8272035	4.00E-07	-0.6069527	0.00031643

Cdkl1	skyblue	-1.9163027	0.00019504	-1.5455282	0.00309114
Ttc8	red	-1.1473855	2.03E-09	-0.8282304	2.02E-05
Polr2h	orange	0.63296131	8.88E-06	0.4233977	0.00538015
Psma6	orange	0.82919011	2.22E-05	0.67266568	0.00088967
Nfkbia	orange	1.0447492	2.20E-10	0.60070062	0.00050097
Ngb	skyblue	-1.8045055	1.22E-06	-1.5904279	2.63E-05
Gstz1	red	-1.0832015	1.72E-05	-0.8193643	0.00168264
Nova1	red	-1.0201232	4.02E-07	-0.923945	1.69E-05
Esr2	skyblue	-2.0953256	0.00010462	-1.9059067	0.000585
Akap5	red	-0.9650822	0.00056119	-0.3463792	0.26667481
Atl1	red	-0.9928799	0.00043936	-0.7485904	0.0106068
Pygl	red	-0.7043453	0.00091056	-0.6483268	0.00299072
Timm9	orange	0.61833747	0.00314133	0.31155591	0.17402392
Rtn1	red	-1.0357044	0.00024458	-0.9945858	0.00062201
Serpina3n	yellow	3.15244253	8.17E-28	3.04438788	6.15E-26
Dhrs7	red	-0.6179714	3.51E-08	-0.6603458	6.43E-09
Clmn	red	-0.9563685	1.54E-06	-0.693648	0.00070122
Hif1a	orange	1.6498011	4.00E-17	1.46702886	1.44E-13
Mpp5	red	-1.0520161	4.40E-05	-0.9185555	0.00053906
Atp6v1d	orange	0.59795465	6.92E-05	0.36905314	0.0201881
Vrk1	orange	0.72838182	6.12E-11	0.62625993	4.75E-08
Eif2s1	orange	1.03505803	1.36E-08	0.73043423	0.00011404
Arg2	yellow	2.56482079	6.84E-13	1.50791827	1.74E-05
Erh	orange	0.86878037	4.47E-09	0.76746692	4.71E-07
Susd6	orange	0.6050992	1.86E-07	0.4245088	0.00043216
Smoc1	skyblue	-1.6119313	6.42E-07	-1.2202521	0.00022174
Zmynd11	red	-0.6456647	5.34E-06	-0.5560937	0.00014173
Cdca7l	orange	0.88561979	3.83E-08	0.74196665	6.46E-06
Psmc1	orange	0.7278851	5.13E-07	0.53643974	0.00036775
Fbln5	skyblue	-1.6553973	1.30E-08	-1.1862616	8.44E-05
Tc2n	orange	0.63843586	0.00401362	0.60415322	0.00901599
Lgmn	yellow	2.4479671	3.84E-11	2.08937498	3.39E-08
Pfkp	orange	1.51234927	2.25E-18	1.31563903	5.97E-14
Unc79	skyblue	-3.5188505	7.69E-13	-2.0428144	9.74E-06
Asb2	red	-0.7635579	0.00118116	-0.6778195	0.00564527
Otub2	red	-0.7212815	0.0015545	-0.6774828	0.00384655
Ifi27l2b	yellow	3.42580307	1.17E-06	2.76430767	0.00020143
Ppp4r4	red	-1.012483	8.40E-05	-0.798895	0.00277669
Akr1c13	orange	0.67806749	0.00810456	-0.1166204	0.7084278
Net1	red	-0.825797	0.00010464	-0.8470075	9.83E-05
Rgs6	skyblue	-1.4816588	6.45E-10	-1.5695249	8.43E-11
Fam161b	skyblue	-1.8938803	4.52E-07	-1.1562011	0.00269492
Entpd5	red	-0.8418624	3.97E-08	-0.7100093	6.35E-06

Aldh6a1	red	-1.447906	0.00015524	-1.0634933	0.0076681
Fcf1	orange	0.83383757	3.89E-06	0.82544454	8.75E-06
Mlh3	red	-0.836732	1.78E-12	-0.8565376	1.84E-12
Esrrb	skyblue	-0.9732317	0.22038364	-2.4410904	0.00264499
Vash1	red	-0.7212801	8.12E-05	-0.6123887	0.00120646
Angel1	red	-0.5814492	0.00018765	-0.4062772	0.01351907
Hhipl1	orange	1.08256697	0.00021783	0.90089226	0.00289367
Evl	orange	1.24277483	1.57E-11	1.29569237	3.39E-12
Wars	yellow	2.36925045	4.04E-66	2.3196848	2.32E-63
Meg3	skyblue	-1.7140899	2.91E-13	-1.0844558	5.31E-06
Traf3	orange	0.62309174	0.00023689	0.71224099	3.57E-05
Amn	yellow	3.40213366	4.22E-05	3.21674468	0.00022657
Exoc3l4	orange	0.87899126	0.00014795	0.84514215	0.00036096
Tnfaip2	yellow	2.40346466	4.21E-23	2.62582253	2.29E-27
Zfyve21	red	-1.0202581	0.00023809	-0.7735459	0.00742792
Kif26a	skyblue	-1.9293714	5.86E-14	-1.418247	7.46E-08
Gpr132	yellow	2.30569427	9.09E-31	2.26653665	1.43E-29
Gng4	skyblue	-2.0624442	7.48E-06	-1.3653661	0.00421081
Amph	red	-1.4634601	3.68E-07	-0.9771333	0.0010696
Sfrp4	skyblue	-3.8248314	3.07E-08	-2.0809554	0.00339118
Aoah	yellow	3.85330633	3.59E-15	3.83490122	1.54E-14
Irf4	orange	1.27815061	3.86E-07	1.26629083	9.88E-07
Mak	skyblue	-1.872215	3.69E-15	-1.1245446	2.62E-06
Edn1	red	-0.2701281	0.08556922	-0.7415766	9.03E-07
Cap2	skyblue	-1.4724747	1.24E-07	-1.389119	1.15E-06
Kif13a	red	-0.7518924	5.75E-05	-0.6014828	0.00187458
Dek	orange	0.79488012	5.50E-07	0.62597361	0.00014407
Id4	red	-1.3062991	5.61E-07	-1.141227	2.19E-05
Susd3	orange	2.03442632	2.05E-10	1.81796865	3.31E-08
Aspn	skyblue	-1.8170598	2.05E-11	-1.7038808	8.27E-10
Ogn	skyblue	-1.6415826	1.23E-06	-1.3478397	0.00011254
Serpinb9b	yellow	3.36308927	1.86E-09	3.75265812	1.07E-10
Ripk1	orange	1.15058305	6.95E-20	0.9506234	1.06E-13
Ly86	yellow	3.04202226	1.21E-31	2.64277047	1.46E-23
Riok1	orange	0.81827746	1.95E-09	0.69078405	8.74E-07
Sema4d	yellow	2.49827577	5.44E-30	2.39542473	1.83E-27
Gadd45g	orange	1.96502292	2.73E-24	1.79761918	2.09E-20
Syk	orange	1.12857676	1.16E-06	1.07761926	5.61E-06
Fancc	red	-0.9924391	1.34E-12	-0.6305339	1.82E-05
Ptch1	red	-0.9720768	1.35E-05	-0.5198765	0.02855703
Habp4	red	-0.6545505	2.40E-08	-0.4236633	0.00053776
Aaed1	skyblue	-1.2500001	2.86E-08	-1.3721114	1.59E-09
Mxd3	yellow	2.84303307	5.66E-18	3.27183482	8.23E-22

Prelid1	orange	0.88750167	2.11E-06	0.63178811	0.00118365
Pdlim7	orange	0.54785701	0.01840318	0.61409742	0.00939572
Ddx41	orange	0.73211251	2.30E-09	0.54089299	1.87E-05
Fam193b	red	-0.7084992	1.62E-06	-0.6343562	2.87E-05
Fastkd3	orange	0.60184909	0.00016439	0.33137914	0.05210296
1700001L19F	red	-1.4743155	5.80E-05	-0.4735958	0.23772295
Adcy2	red	-0.774173	1.21E-07	-0.8516993	9.49E-09
Nkd2	skyblue	-1.4244595	2.24E-09	-1.3873375	8.95E-09
Trip13	yellow	2.92095918	1.43E-27	2.86025272	5.54E-24
Tppp	skyblue	-2.2451849	0.00011589	-1.7871006	0.00305708
Pdcd6	orange	0.58234323	1.57E-06	0.44501461	0.0004156
Erap1	orange	0.8703294	2.80E-18	0.83986365	6.32E-17
Rhobtb3	red	-0.7249741	0.00085304	-0.6544463	0.00340913
Glrx	orange	2.00503335	3.10E-12	1.71075557	5.56E-09
Arsk	red	-0.9854825	2.55E-05	-1.2419151	1.24E-07
Srd5a1	red	-0.7054896	0.03592985	-1.0966631	0.00170943
Nsun2	orange	0.67026066	0.00024591	0.48071934	0.01198581
Lpcat1	red	-0.8916868	9.16E-05	-0.5685529	0.01781496
Hapln1	skyblue	-1.3461509	0.02261029	-1.6975255	0.00490076
Vcan	yellow	2.26762506	2.01E-13	2.29611056	1.42E-13
Ckmt2	skyblue	-2.8024942	1.52E-09	-2.0353778	2.22E-05
Cd180	yellow	3.63136321	5.72E-10	3.25264294	5.41E-08
Ocln	red	-0.7781958	9.88E-16	-0.5744625	7.93E-09
Smn1	orange	1.05401426	8.81E-09	0.69321568	0.00029304
Mccc2	red	-1.0048025	5.62E-06	-0.9667578	2.10E-05
Btf3	orange	0.68829723	1.46E-05	0.45942613	0.00573643
Arhgef28	red	-1.374576	2.93E-12	-0.8064045	8.35E-05
Hexb	orange	0.9305982	6.53E-05	0.55256295	0.02535688
Hmgcr	orange	0.65837279	1.66E-08	0.72520602	7.16E-10
F2rl2	orange	1.17274681	1.35E-05	0.76651007	0.01080797
Iqgap2	orange	0.98204846	4.85E-11	0.93501133	7.18E-10
Pde8b	skyblue	-1.7144006	2.65E-09	-1.3563377	4.67E-06
Jmy	red	-1.1550529	1.58E-08	-0.8162803	0.00011896
Depdc1b	yellow	2.59848645	1.21E-07	3.37306594	2.25E-10
Thbs4	skyblue	-2.1679181	2.00E-05	-1.0050062	0.06740693
Serinc5	red	-0.8877112	3.10E-06	-0.5007286	0.0126766
Dhfr	orange	1.8613128	5.57E-11	1.4605111	7.04E-07
Rasgrf2	skyblue	-1.7675423	3.73E-07	-1.2905856	0.00034724
Cenpk	yellow	3.0883551	2.35E-08	2.28804362	7.08E-05
Cwc27	orange	0.64149818	0.00385747	0.73974818	0.00114513
Rgs7bp	skyblue	-1.8444734	2.01E-05	-1.5501652	0.00050088
Rnf180	orange	1.8928958	4.74E-07	0.78988974	0.050507
Parp8	orange	0.71945947	1.23E-08	0.49835859	0.00015162

Hcn1	skyblue	-1.5540875	1.25E-05	-1.5536297	3.96E-05
Fgf10	red	-1.0493411	1.08E-07	-0.8952102	1.19E-05
Slc4a7	orange	0.80872476	2.83E-07	0.69533135	2.07E-05
4930452B06I	skyblue	-1.6570384	1.33E-09	-1.6272453	9.32E-08
Fam107a	orange	0.92646095	0.00264794	-0.3403305	0.32078262
Acox2	red	-1.2813349	2.22E-11	-0.7940874	4.68E-05
Gpx8	red	-0.8775298	1.84E-14	-0.5401721	6.25E-06
BC067074	red	-0.8121659	3.17E-07	-0.3889908	0.02049839
Fst	yellow	3.28339787	1.05E-13	2.11665414	3.40E-06
Kat6b	red	-0.6112611	0.00107421	-0.3916544	0.04754225
Nr1d2	skyblue	-1.5629382	1.08E-11	-1.3223052	1.81E-08
Thrb	red	-1.3227371	5.41E-10	-1.0337309	1.84E-06
Sftpa1	red	-1.0698106	0.00452703	-0.6141471	0.12935999
Dydc1	skyblue	-1.3678221	0.02691306	-1.5893011	0.00638978
Dydc2	skyblue	-2.2775564	0.00130476	-0.7586577	0.27901795
Fam213a	red	-1.1178325	2.96E-07	-0.956245	2.03E-05
Bmpr1a	red	-0.8426915	1.25E-08	-0.6540982	1.89E-05
Ldb3	skyblue	-1.5888358	7.73E-06	-1.1622613	0.00164872
Nid2	red	-1.0180367	7.75E-07	-0.777644	0.00025219
Dnajc9	orange	0.87521973	1.03E-10	0.60609792	1.81E-05
Plau	yellow	2.29908045	4.91E-14	2.35688288	1.70E-14
Vcl	red	-0.629016	0.00274388	-0.3065945	0.17980801
Txndc16	red	-1.039664	7.02E-09	-0.9663312	1.32E-07
Bmp4	red	-0.7899316	6.15E-05	-0.5070097	0.01448886
Samd4	red	-0.9633661	0.00010479	-0.7376881	0.0042369
Rnase4	skyblue	-1.583519	2.26E-11	-1.3506833	2.34E-08
Dnah12	skyblue	-1.8370718	2.03E-11	-0.7702379	0.00669709
Rnase6	orange	1.02387629	0.00017093	1.33353057	1.82E-06
Gpr65	yellow	3.73446345	3.92E-32	3.12208697	3.19E-22
Asb14	red	-1.2629505	2.81E-06	-1.0895886	9.36E-05
Btd	red	-0.5700976	0.00059515	-0.6262293	0.00022222
Sema3g	skyblue	-1.323555	1.94E-19	-1.2329983	7.30E-17
Glt8d1	red	-0.683734	4.73E-08	-0.6498817	4.10E-07
Kpna3	orange	0.58769394	0.00039731	0.32496912	0.06755748
Rnaseh2b	orange	1.00669056	1.36E-06	0.77208748	0.00039701
Ctsb	orange	1.48365795	2.98E-07	1.22052624	4.28E-05
Gdf10	skyblue	-1.8263364	3.25E-07	-0.9717583	0.00994214
Gata4	skyblue	-1.0940134	0.11572865	-1.8446454	0.00749588
Prkcd	orange	0.90286902	1.07E-08	0.77093169	1.94E-06
Anxa8	red	-0.7939498	0.00028253	-0.5344051	0.01990462
Pinx1	orange	0.87778255	0.00499725	0.62901599	0.06002996
Ska3	yellow	2.61066455	3.97E-10	3.0901325	1.12E-12
Mrpl57	orange	0.67174212	0.00016001	0.63488826	0.00056491

Cdadc1	red	-0.6465644	9.90E-05	-0.5455776	0.00147649
Amer2	yellow	2.26817325	0.00066998	1.5431864	0.02186601
Spata13	orange	1.08892795	3.05E-11	0.88300355	1.47E-07
Esd	orange	0.95962216	1.95E-06	0.78641429	0.00016143
Lcp1	orange	2.06436491	1.25E-16	1.65786104	7.24E-11
Nufip1	orange	0.90723099	3.94E-08	0.87064014	3.38E-07
Tsc22d1	red	-0.945306	3.54E-09	-0.9640656	2.67E-09
Dnajc15	orange	0.68835782	4.05E-05	0.41175238	0.02163624
Epsti1	yellow	3.52984546	1.80E-46	3.28190914	1.02E-39
Tnfsf11	yellow	3.77538153	6.76E-12	2.38178862	2.68E-06
Diap3	orange	1.66076908	1.64E-07	1.57691659	1.33E-06
Scara5	red	-1.2089614	7.44E-08	-0.6668567	0.00466193
Pbk	yellow	2.21007129	1.44E-07	3.42443068	3.79E-14
Esco2	yellow	2.74177761	3.78E-16	3.32285249	3.46E-20
Ccdc25	orange	1.22990932	3.29E-17	1.14728381	8.02E-15
Ephx2	skyblue	-1.9424786	8.27E-07	-1.7546409	1.54E-05
Chrna2	orange	-0.0682316	0.93190243	2.36706333	0.0016111
Stmn4	skyblue	-1.7381615	0.03156679	-2.3673519	0.00960255
Dpysl2	red	-1.119044	9.19E-15	-1.036768	1.32E-12
Bnip3l	red	-0.7514927	2.22E-06	-0.7466445	4.15E-06
Ebf2	skyblue	-2.346969	8.45E-07	-1.5061869	0.00235244
Adam7	skyblue	-2.8819767	0.00031442	-3.2820576	0.00013103
Adamdec1	skyblue	-1.901449	2.91E-11	-0.9694609	0.00079054
Bora	orange	1.52857028	9.34E-11	1.46420213	1.21E-09
Tnfrsf10b	orange	0.25197664	0.30004628	0.63587293	0.00684822
Bin3	orange	1.00220878	5.11E-10	0.76965272	3.78E-06
Ppp3cc	orange	0.93516818	1.66E-07	0.82647307	7.96E-06
Slc39a14	yellow	2.37587872	7.99E-17	2.23150639	7.94E-15
Hr	skyblue	-1.7040261	8.73E-13	-1.123573	5.64E-06
Sftpc	skyblue	-2.0050133	6.98E-05	-1.2378531	0.01993711
Dmtn	red	-1.3688185	2.31E-18	-0.9245632	8.93E-09
Dok2	yellow	2.25143081	2.80E-17	2.32383717	5.28E-18
Gfra2	red	-1.1390693	3.92E-05	-1.1894626	2.85E-05
Sucla2	red	-0.6723564	5.58E-09	-0.5244976	1.16E-05
Uchl3	orange	0.8868092	0.00036705	0.68742065	0.00828655
Ednrb	skyblue	-1.9077686	9.16E-08	-1.5181056	3.71E-05
Scel	red	-1.0840304	2.16E-18	-0.9686134	1.11E-14
Irg1	black	11.9299782	4.23E-18	9.59200186	2.49E-29
Cldn10	red	-1.2812045	4.96E-07	-0.6252484	0.01999532
Osmr	orange	0.71921609	4.16E-06	0.8195583	2.16E-07
Fyb	yellow	3.44431529	4.39E-33	3.11959437	4.06E-27
Dab2	orange	0.83911413	0.00110269	0.92787479	0.00040318
Ttc33	red	-0.5260279	0.01067507	-0.6664574	0.00147814

Rem2	red	-0.7898483	0.0108876	-1.0200811	0.00244522
Haus4	orange	1.15413245	1.05E-17	0.60268319	2.39E-05
Ajuba	red	-0.5821492	2.93E-09	-0.3876337	0.00014561
4931414P19I	red	-0.6330367	3.40E-05	-0.4643846	0.00406421
Slc7a8	yellow	3.62747131	2.10E-19	3.41604343	3.71E-17
C6	red	-0.5932127	0.00376891	-0.4925102	0.02075815
Oxct1	red	-0.8303979	9.29E-11	-0.7585471	6.79E-09
Psmb5	orange	0.70366067	3.19E-05	0.60877275	0.00050969
Pdzd2	red	-1.180141	0.00018123	-0.7890542	0.01712267
Slc22a17	red	-1.341644	5.42E-09	-1.1031044	2.91E-06
Ngdn	orange	0.67360886	4.69E-05	0.67799583	6.57E-05
Sub1	orange	1.02922466	8.04E-07	0.69907059	0.00131726
Npr3	skyblue	-2.1054855	0.00643402	-0.80452	0.35018466
Fitm1	red	-1.5298776	5.03E-05	-1.0080293	0.01138333
Psme1	orange	1.88973875	2.19E-45	1.70332928	6.23E-37
Emc9	red	-0.6180078	0.00288749	-0.5646711	0.00846472
Ripk3	yellow	2.23531182	1.87E-19	2.20482451	8.27E-19
Zscan26	red	-0.8479148	9.50E-06	-0.717165	0.00027921
Cmbl	red	-1.070293	6.90E-08	-0.9889025	1.20E-06
Ctnnd2	red	-1.3298543	6.34E-07	-0.9001007	0.00112673
Tars	orange	0.69185126	8.49E-09	0.63385834	2.51E-07
Amacr	red	-0.8736788	0.00178714	-0.7195673	0.01299873
Brix1	orange	0.62363674	0.005692	0.51589241	0.02845156
Nadk2	red	-0.7554613	0.00021879	-0.6833279	0.00124368
Laptm4b	red	-0.8507026	7.13E-12	-0.6895059	5.44E-08
Sdc2	skyblue	-1.6184191	9.61E-14	-1.2830598	8.13E-09
Dnah5	skyblue	-3.3511212	8.52E-15	-2.0521148	3.85E-06
Ank	red	-0.9742563	6.46E-08	-1.008361	3.72E-08
Pabpc1	orange	0.60088319	6.54E-08	0.37868114	0.00111885
Grhl2	red	-1.2199746	1.17E-10	-0.632427	0.00133451
Rrm2b	red	-0.8955664	4.25E-07	-0.8605056	2.11E-06
Fzd6	red	-1.1105462	1.23E-10	-1.0519617	2.33E-09
Dcaf13	orange	0.88060568	1.60E-08	0.68269196	2.39E-05
Zfpm2	red	-1.0517444	4.68E-06	-0.7793091	0.00093507
Oxr1	red	-0.7604342	6.02E-08	-0.5542571	0.0001381
Angpt1	skyblue	-1.3380705	7.69E-06	-1.3276362	1.41E-05
Shcbp1	yellow	3.98682478	1.76E-25	3.76994708	8.22E-22
Hrsp12	red	-1.064908	6.29E-07	-0.6881415	0.00226759
Matn2	red	-0.9422219	2.03E-09	-0.8466423	1.78E-07
Pop1	orange	0.71815285	8.21E-05	0.94868086	3.32E-07
Sybu	red	-0.8722042	0.00478279	-0.8198587	0.00981674
Myc	yellow	2.48935079	2.41E-15	2.68404982	1.67E-17
Mtss1	red	-0.7516598	1.92E-13	-0.5289994	5.81E-07

Klhl38	skyblue	-1.528464	0.01263824	-1.8018802	0.00771923
Atad2	orange	0.87551729	7.27E-07	0.82043862	6.87E-06
Has2	orange	1.52587328	0.02477263	2.46636187	0.00055894
Mtbp	orange	1.42165595	1.14E-14	1.12356262	3.17E-09
Mrpl13	orange	0.73318996	0.00402711	0.50952018	0.05931965
Col14a1	skyblue	-1.9981735	1.61E-21	-1.2382465	1.04E-08
Sla	yellow	2.60621381	7.37E-24	2.5134241	3.98E-22
Lrrc6	skyblue	-2.5389482	8.41E-10	-1.431156	0.00040558
Adcy8	red	-1.3245729	7.02E-05	-0.7954707	0.02373064
Fam49b	yellow	2.19122891	1.21E-20	1.83681853	1.33E-14
Ppara	skyblue	-1.6110043	3.00E-05	-1.1320461	0.00446616
Gtse1	yellow	3.00401636	2.34E-27	2.65026555	2.25E-21
Tef	skyblue	-1.5496759	5.17E-05	-1.2565197	0.00151231
Fam83f	orange	1.07746214	0.00162032	0.72456692	0.04584592
Deptor	red	-1.0244126	5.06E-05	-0.8926662	0.00061767
Dnal4	red	-0.664441	1.71E-14	-0.4440027	7.49E-07
Nptxr	red	-0.7139928	0.00047079	-0.4425911	0.04128306
Dscc1	yellow	2.60185393	5.03E-07	2.60067748	1.27E-06
Enpp2	skyblue	-1.6318772	2.80E-08	-1.2243254	5.54E-05
Sh3bp1	orange	1.3254862	4.34E-12	0.8687746	1.50E-05
Parvg	yellow	2.58145932	9.60E-25	2.49845305	5.30E-23
C1qtnf6	orange	0.88985526	0.00010422	0.77199371	0.00112936
Efcab6	skyblue	-2.1597244	1.42E-08	-1.2770208	0.00100776
Ttll1	red	-0.5941791	6.33E-05	-0.7951688	2.09E-07
Myh9	orange	0.61061699	0.00305522	0.70956958	0.00070867
Adamts20	skyblue	-2.1202025	1.38E-05	-0.7991775	0.13341746
Desi1	orange	0.60881399	0.00043267	0.66066374	0.00018548
Aco2	red	-0.6979137	3.32E-09	-0.6058765	5.35E-07
Vdr	orange	0.69846004	0.0002143	0.13797781	0.53320068
Col2a1	skyblue	-3.8175945	0.00070529	-2.7367511	0.01827306
Nckap1l	yellow	2.40492885	1.44E-18	2.32027554	3.41E-17
Pde1b	yellow	2.0278518	6.13E-09	2.10219881	2.49E-09
Litaf	orange	2.0143322	8.60E-17	1.94104111	1.62E-15
Prm1	black	4.68718221	0.00019339	5.65708094	8.76E-05
Nubp1	orange	1.07678263	3.67E-08	1.12219508	1.56E-08
Ciita	yellow	1.89136676	1.70E-22	2.0700855	1.00E-26
Emp2	red	-0.7439188	2.12E-06	-0.4423864	0.00735002
Bcl6	orange	-0.1366113	0.3415519	0.63758222	1.65E-06
Cldn1	red	-1.2451169	4.83E-06	-0.9404988	0.00086388
4930562C15	red	-1.297508	8.20E-07	-0.5190866	0.05939231
Srl	skyblue	-1.5134079	3.39E-06	-1.1906908	0.00042109
Fgf12	skyblue	-1.5082009	0.13092093	-2.642464	0.00756147
Hrasls	skyblue	-2.3556193	5.22E-08	-2.1540462	2.52E-06

Hes1	red	-0.5942906	0.00192251	-0.3558076	0.08201961
Mefv	black	4.89327972	8.68E-29	6.06842196	2.93E-35
Tmem44	skyblue	-1.3761909	2.47E-08	-1.1926531	2.42E-06
Lsg1	orange	0.69388528	1.97E-06	0.58763264	9.54E-05
4930451G09	skyblue	-1.9155551	4.62E-06	-0.7318126	0.08877305
Gpt	skyblue	-1.6726282	8.59E-07	-1.2315044	0.00047988
Apod	yellow	2.38213375	1.74E-37	2.40976234	3.28E-38
Sharpin	orange	0.74896425	5.15E-11	0.66591915	1.01E-08
Hgh1	orange	0.39067058	0.09814119	0.77661928	0.00083356
Bop1	orange	0.70585893	0.00045799	0.79391812	0.00010983
Slc52a2	orange	0.66714839	4.51E-05	0.40352642	0.02006499
Oplah	red	-0.7897095	4.69E-05	-0.6417985	0.00137202
Grina	orange	1.0492035	1.86E-20	0.90119999	3.34E-15
Tsta3	orange	0.62412015	1.28E-06	0.41213664	0.00219345
Gsdmd	orange	1.5414346	3.15E-33	1.28793387	2.25E-23
Gpihbp1	red	-0.7359135	0.00256302	-0.7634434	0.0022249
Ly6c2	black	5.64537496	5.03E-61	5.38729598	2.58E-55
Ly6i	black	5.48203406	1.98E-62	4.60788894	1.57E-45
Ly6e	orange	1.24373303	2.17E-29	1.07294893	5.77E-22
Lynx1	red	-1.433952	2.12E-12	-0.8539655	6.15E-05
Lypd2	skyblue	-1.8332717	7.73E-07	-1.3896274	0.0002953
Psca	yellow	2.61280025	0.00048717	2.27957318	0.0058691
Arc	orange	1.69393716	6.92E-07	1.04415324	0.00291651
Ptk2	red	-0.6856994	2.38E-13	-0.4290851	1.06E-05
Mapk12	red	-0.9086602	1.39E-09	-0.5836914	0.00018905
Tymp	skyblue	-2.8190755	7.81E-15	-1.8273763	9.16E-07
Rabl2	skyblue	-1.5080745	1.31E-11	-0.8915202	0.00011365
Acr	orange	1.45313936	9.28E-06	1.13623805	0.00070039
Kif21a	red	-1.3689115	8.41E-10	-1.0025958	1.38E-05
Alcam	red	-0.9541777	5.40E-07	-0.6197107	0.00181834
Cblb	orange	1.13201618	2.14E-11	0.61561658	0.00052865
Dubr	red	-0.7519308	6.51E-05	-0.7285927	0.00017601
Retnlb	black	6.10274154	9.91E-05	4.45870685	0.00279007
Retnlg	yellow	3.22767566	6.99E-11	2.69030485	5.31E-08
Morc1	orange	0.51954581	0.41502942	1.94453906	0.00144523
Pvrl3	red	-1.3038402	4.44E-09	-1.0803025	2.23E-06
Cd96	yellow	2.65412724	4.23E-13	2.74119712	3.02E-13
Ccdc80	red	-0.4163734	0.04264056	-0.6151685	0.00273085
Cd200r1	orange	1.67549012	0.00045394	1.80228574	0.00023283
Mcm4	orange	1.11923318	1.20E-16	0.73781405	1.46E-07
Snai2	red	-0.3922794	0.1024344	-1.0822206	3.64E-06
Mpv17l	red	-1.4671345	1.39E-07	-0.9814612	0.0007185
B3gnt5	orange	0.93666893	9.62E-06	0.92578593	1.85E-05

Sidt1	yellow	1.86169214	6.73E-17	2.24527857	1.96E-23
2610015P09I	red	-1.0725252	7.47E-07	-0.7005959	0.00204054
Mrpl40	orange	0.61393796	0.00035694	0.37052663	0.04316663
Gbe1	red	-0.6888035	3.98E-05	-0.616804	0.00035466
Zbtb20	red	-0.8337556	1.01E-05	-0.9168468	1.79E-06
Pmm2	orange	0.67962128	1.80E-09	0.5786756	6.11E-07
St3gal6	red	-1.1464169	1.86E-12	-0.886748	1.18E-07
Adgrg7	black	6.7836695	1.11E-05	3.09122928	0.0229785
Slc7a4	skyblue	-2.0204689	9.20E-09	-1.3891608	0.00013253
P2rx6	skyblue	-2.8347775	1.84E-14	-1.8012105	1.81E-06
Lrrc74b	skyblue	-2.6568773	2.80E-09	-1.4066332	0.00224185
Aifm3	red	-1.1224154	0.00099425	-0.9305134	0.00884865
Sdf2l1	orange	1.40191377	5.75E-08	1.37247878	2.02E-07
Spag6	skyblue	-2.4698531	4.08E-19	-1.5494644	1.28E-08
B4galt4	red	-1.1572726	8.85E-09	-1.1716845	9.14E-09
Maats1	skyblue	-1.5924026	2.48E-10	-0.858971	0.00095638
Nr1i2	skyblue	-2.6255201	3.99E-06	-1.9271495	8.54E-05
Umps	orange	0.94816364	1.62E-07	0.79420652	2.07E-05
Muc13	yellow	1.85460934	0.00111379	2.05240282	0.0006244
Rabl3	orange	0.48228364	0.02231504	0.79674633	0.00015621
Hcls1	yellow	3.04414216	1.03E-23	2.86493745	5.68E-21
Mylk	red	-0.7239023	5.44E-07	-0.5982325	5.91E-05
Adcy5	skyblue	-1.6722857	2.93E-07	-1.0732482	0.00158517
Clcn2	red	-0.8183064	2.57E-05	-0.5961694	0.00304926
Pdia5	red	-0.7954371	1.91E-06	-0.7211969	3.06E-05
Dirc2	red	-1.039713	7.03E-06	-0.9597084	5.32E-05
Dgkg	skyblue	-1.6206759	4.63E-05	-0.7070558	0.0889971
D16Ertd472e	orange	0.78508037	2.32E-11	0.59507221	9.83E-07
Cxadr	red	-1.0956749	4.94E-20	-0.6422128	2.37E-07
Usp25	orange	0.72386983	3.40E-14	0.57993243	2.97E-09
Fetub	skyblue	-1.9788692	5.87E-06	-1.1389284	0.01312875
Samsn1	yellow	2.86962834	4.73E-31	2.4977848	1.74E-23
Adipoq	skyblue	-2.8702884	0.00041214	-1.7578817	0.04078991
Rfc4	orange	1.35755666	7.49E-09	1.32176812	4.97E-08
Robo1	red	-0.9212905	5.07E-05	-0.9180354	7.59E-05
St6gal1	red	-0.6172828	2.37E-09	-0.3131955	0.00410127
Adamts5	red	-1.5486896	9.87E-09	-1.0040519	0.0003459
Cd86	yellow	2.76196345	6.32E-15	2.41030635	2.16E-11
Parp9	yellow	2.54673485	5.74E-78	2.35310213	1.16E-66
Pigp	red	-0.782442	1.22E-05	-0.7652902	3.15E-05
Ripply3	red	-1.1219659	1.49E-06	-0.8375265	0.00053986
Chaf1b	yellow	3.04138091	6.25E-29	2.25532228	8.05E-17
Cbr3	red	-0.908083	4.16E-06	-0.747583	0.00023834

Clic6	skyblue	-2.8159993	1.72E-34	-1.5647125	1.79E-11
Rcan1	orange	0.47470052	0.00269891	0.61041803	0.0001394
Runx1	orange	0.6368799	0.0001081	0.51274317	0.00263527
Itsn1	red	-0.723339	1.34E-12	-0.4004555	0.0001901
Donson	orange	0.6106862	8.19E-06	0.35724551	0.01503108
Tmem50b	red	-1.050985	6.35E-09	-0.895784	1.34E-06
Mis18a	orange	0.9421183	5.07E-08	0.75488522	2.75E-05
Adcy6	red	-0.7826924	4.58E-06	-0.6032185	0.00065367
Enah	red	-0.8917351	1.41E-08	-0.3927187	0.01905571
Wnt10b	skyblue	-2.4928809	1.19E-11	-1.6903959	7.79E-06
Tuba1b	orange	0.81017673	1.47E-08	0.66182844	7.01E-06
Prpf40b	red	-0.664335	7.38E-05	-0.6494947	0.00015394
Faim2	skyblue	-3.1936677	1.16E-07	-2.22811	0.00036073
Racgap1	yellow	2.84757332	5.57E-53	2.73808554	4.80E-48
Asic1	red	-0.6139943	0.00129487	-0.4260496	0.03672948
Gpd1	skyblue	-2.5802999	4.36E-05	-1.8953287	0.00388485
Cela1	yellow	2.21129029	1.91E-05	1.73751956	0.00126509
Krt7	orange	0.90395957	5.28E-05	1.09752313	1.20E-06
Krt6b	skyblue	-2.3457402	0.0040136	-1.2278751	0.16695183
Krt18	orange	1.97405063	1.05E-11	1.81536495	7.37E-10
Csad	red	-1.0678114	4.00E-08	-0.8533969	2.07E-05
Soat2	black	4.31312925	9.01E-37	4.82865854	4.32E-44
Igfbp6	skyblue	-1.8845916	0.00072961	-1.3644472	0.01948362
Prr13	orange	1.02852582	5.13E-09	0.8516071	2.58E-06
Map3k12	red	-0.6613866	2.32E-05	-0.385633	0.01812528
Calcoco1	red	-1.328262	1.25E-05	-1.0606792	0.00074643
Sncg	red	-0.5266814	0.01303527	-0.7372584	0.00042894
Cdkn1a	yellow	2.65926043	7.37E-58	2.2225687	1.69E-40
Cxcl13	yellow	3.11378354	2.92E-06	3.25384392	1.53E-06
Lrrc71	red	-1.3786842	1.70E-07	-0.5585856	0.04703664
Noct	yellow	2.25344633	2.92E-11	1.88800269	4.87E-08
Fhl1	skyblue	-1.5068859	1.13E-09	-1.103569	1.61E-05
Msrb2	red	-1.4665975	2.45E-11	-1.0483231	2.90E-06
Rfc2	orange	0.63872866	7.83E-07	0.42070565	0.00183353
Denr	orange	0.59780398	0.00051672	0.41147527	0.0233534
Prmt5	orange	0.58796664	8.13E-05	0.43442391	0.00523467
Gzma	yellow	4.23448179	1.14E-55	3.82118602	4.93E-45
Wrb	red	-0.8774686	3.76E-05	-0.8032708	0.00023368
Ivns1abp	red	-0.6721866	0.00050117	-0.6420986	0.00121064
Slc38a1	orange	0.83702525	9.94E-08	0.81182732	3.82E-07
Vwa5a	orange	0.70571157	1.06E-14	0.62408605	1.78E-11
P3h3	red	-0.8543696	1.56E-10	-0.6152792	8.51E-06
Il15ra	orange	1.49766355	5.43E-13	1.3253632	3.52E-10

Serping1	orange	1.03549099	1.12E-14	0.89603263	4.85E-11
Kcnk5	orange	0.57560807	0.00026213	0.84951975	9.42E-08
Parp3	orange	1.1389542	1.09E-11	1.25937235	6.45E-14
Frs3	red	-0.4593102	0.03380915	-0.7309285	0.00082163
Cd4	yellow	2.06879966	1.91E-06	3.26071601	4.19E-14
Twf2	orange	0.74573877	8.95E-05	0.39638974	0.05196715
Ube2j2	orange	0.81941162	7.16E-07	0.72465518	2.06E-05
March5	orange	0.74263998	2.47E-08	0.70584021	2.01E-07
Ache	red	-1.5526839	0.00012279	-0.524617	0.24483723
Wfdc1	skyblue	-2.8052143	1.21E-21	-1.8527321	6.24E-10
Mx2	yellow	4.03589189	9.89E-51	3.73047103	5.22E-44
Poc1a	orange	1.30547953	1.60E-08	1.04159812	1.39E-05
Clec4n	orange	1.80118371	3.14E-05	1.4620219	0.0010895
Slc17a9	orange	0.63012134	0.00012834	0.61116907	0.00031153
Pisd	orange	0.6935434	2.70E-05	0.71070859	2.55E-05
Celsr3	yellow	2.98346362	2.58E-05	1.70784645	0.00922741
Cdca3	yellow	2.94929951	3.01E-19	3.16629718	2.47E-21
Fam132a	red	-0.529878	0.0104078	-0.6763239	0.00130184
Mrps23	orange	0.68709347	0.00067242	0.45990657	0.03158392
Pigx	orange	0.87004153	1.03E-05	0.56257333	0.00677079
Rsph3b	red	-0.7626499	2.51E-06	-0.733179	1.36E-05
Park2	red	-0.7557113	0.00747588	-0.6008842	0.0399142
Slc22a3	skyblue	-1.184899	0.00025909	-1.363816	4.30E-05
Acat2	red	-0.5471504	0.01538104	-0.6274224	0.00618195
Lnpep	orange	0.62124948	1.26E-05	0.43038697	0.00378691
Pde10a	orange	1.55855925	0.00078286	1.46720595	0.00198098
Smoc2	red	-0.6785074	2.11E-05	-0.2909365	0.09216983
Mmp25	yellow	3.36367474	1.56E-14	2.84975241	5.54E-11
Hcfc1r1	red	-0.5864993	8.89E-06	-0.5195728	0.00015394
Tnfrsf12a	yellow	2.54968292	4.10E-13	2.7623898	4.64E-15
Pkmyt1	orange	1.25971594	2.10E-08	1.20892551	1.51E-07
Slc25a27	red	-1.2265236	0.00032765	-1.1017198	0.00172844
Pla2g7	yellow	3.82216061	1.96E-19	3.59164926	3.93E-17
Cenpq	orange	1.38205521	1.36E-06	1.08107175	0.00030601
Mut	red	-0.9062704	4.62E-05	-0.6876849	0.00291295
Satb1	orange	0.12738112	0.87132917	1.87981194	0.00525535
Sgol1	yellow	3.16755172	6.60E-16	3.7055058	5.48E-19
Slc29a1	red	-0.8564042	6.50E-13	-0.857583	8.54E-13
Nfkbie	yellow	2.25691331	1.83E-19	2.18477474	3.79E-18
Tcte1	skyblue	-2.7462822	3.36E-15	-1.3341902	0.00012057
Vegfa	red	-1.1115389	1.22E-09	-0.7156787	0.00017263
Gtpbp2	orange	0.69296062	8.03E-09	0.72067714	3.02E-09
Enpp5	red	-1.2460665	1.19E-06	-0.9538758	0.00032887

Cyp39a1	skyblue	-1.9101361	1.08E-06	-1.7074819	2.10E-05
Rsph9	red	-0.9266356	1.25E-05	-0.6746019	0.00212163
Crip3	skyblue	-2.0441336	0.00014492	-0.6431994	0.27388445
Guca1a	yellow	2.19687787	0.00284771	2.11157015	0.00590307
Bysl	orange	0.71654618	0.00022702	0.70982282	0.00037536
Trem2	yellow	4.05376491	2.00E-15	3.22008859	6.34E-10
Kif6	skyblue	-1.855221	4.39E-13	-0.9943887	2.44E-05
Ppil1	orange	0.84908657	1.46E-08	0.66813252	1.71E-05
Pi16	skyblue	-1.5591768	8.80E-19	-0.7815172	2.28E-05
Fgd2	orange	1.66730204	1.24E-12	1.65993347	2.46E-12
Pim1	yellow	2.36666466	6.63E-46	2.39564391	5.41E-47
Glo1	red	-0.6312045	3.02E-06	-0.4756646	0.00071618
Glp1r	skyblue	-2.3063579	1.63E-11	-1.820264	2.12E-07
Rsph1	skyblue	-1.8270041	2.93E-14	-1.2613459	3.38E-07
Tmprss3	black	5.19385602	0.00049886	3.23219885	0.011448
Cbs	skyblue	-1.7344979	0.00038752	-0.9057877	0.07848839
Cryaa	skyblue	-1.3806175	0.01623502	-1.5224562	0.00984258
Epb41l3	red	-1.1013619	8.90E-10	-0.9444437	3.47E-07
Myom1	red	-0.6803685	0.00131962	-0.5792142	0.00834943
Emilin2	orange	1.13064798	0.00263134	1.23265968	0.00130537
Smchd1	orange	0.74633113	1.77E-07	0.68168293	3.41E-06
Ndc80	yellow	3.07660915	6.19E-22	2.74745366	5.59E-17
Clip4	orange	0.58661252	0.00080747	0.55083737	0.0022494
Lbh	red	-0.7073183	1.47E-08	-0.607374	2.13E-06
Xdh	orange	1.77129058	3.70E-27	1.41406977	1.70E-17
Crim1	red	-0.9943608	3.73E-08	-0.6141618	0.00114513
Vit	skyblue	-1.2707916	0.00080969	-1.56601	7.97E-05
Eif2ak2	yellow	2.16723741	2.40E-61	1.95349547	1.06E-49
Qpct	red	-0.6822338	0.00044354	-0.5862392	0.00398773
Man2a1	orange	0.82092412	1.10E-13	0.78793716	1.68E-12
Twsg1	red	-1.0787094	7.38E-11	-0.8533658	5.34E-07
Nrxn1	red	-1.3235394	0.0007653	-1.0375861	0.01289517
Atp6v0c	orange	0.73126076	5.97E-05	0.6780523	0.00029914
Prss30	orange	1.69601021	0.0020602	1.58999745	0.00393318
Prepl	red	-0.6514393	3.04E-06	-0.6619944	3.12E-06
Abca3	red	-0.8275915	0.00338054	-0.4247662	0.16516266
Eci1	red	-0.835036	2.68E-09	-0.7046706	9.76E-07
Epas1	red	-0.9592363	0.00032685	-0.7452671	0.00724547
Spsb3	orange	0.45627774	0.00421312	0.58277505	0.00032778
C3	orange	1.33365881	1.41E-10	1.03214527	1.41E-06
Hn1l	orange	0.99826	0.00037649	1.08682662	0.00014749
Tmem204	red	-1.3314558	1.17E-05	-1.1181718	0.00035956
Tekt4	red	-1.1771247	1.63E-07	-0.6435108	0.00555548

Sox8	skyblue	-2.1631303	0.00019057	-0.7656933	0.17255184
Rgs11	skyblue	-1.9484871	1.32E-08	-1.1660914	0.00104977
Itfg3	red	-0.6252554	2.90E-06	-0.4644847	0.00081745
Bnip1	orange	0.61824936	0.00010242	0.62264177	0.00015865
Rfx2	red	-1.0042495	2.86E-12	-0.6968342	1.93E-06
Snrpc	orange	0.73570254	7.16E-08	0.72647924	2.04E-07
Anks1	red	-0.6092356	2.30E-09	-0.298273	0.00565549
Fkbp5	orange	2.11318748	2.77E-34	0.7793772	2.19E-05
Nudt12	red	-1.3269955	3.35E-07	-0.8929119	0.00082163
Bambi	red	-0.9168111	1.47E-08	-0.4870555	0.00379168
Map3k8	orange	1.42088488	2.95E-16	1.24109833	1.61E-12
Tmem178	red	-1.137499	3.87E-11	-0.8908966	5.52E-07
Pkdcc	red	-0.6112491	0.00013295	-0.3795489	0.02545113
Dync2li1	red	-1.2293357	3.36E-15	-0.9093649	1.77E-08
Zfp871	orange	0.65135353	0.00339873	0.95567775	2.14E-05
Adamts10	red	-1.1558388	6.88E-09	-0.8177899	7.70E-05
Myo1f	yellow	2.46726015	3.29E-18	2.28784221	1.28E-15
Kifc5b	orange	1.6113146	1.05E-14	1.97722691	5.52E-20
Dtna	red	-0.8244619	0.00352942	-0.6362129	0.03106623
Cdh2	skyblue	-1.727063	7.39E-05	-2.0176642	5.48E-06
Tapbp	orange	1.99665507	5.05E-25	1.82030916	7.76E-21
Wdr46	orange	0.68307396	0.00040792	0.65307293	0.00097615
Dsc2	red	-0.7062699	0.0878756	-1.1720062	0.00417826
Psmb8	yellow	3.01856519	1.68E-54	2.75484889	2.02E-45
Tap2	yellow	2.08308984	4.88E-75	1.97125336	3.94E-67
Btnl2	yellow	1.88928076	0.00088049	2.08605286	0.00069676
Tmem173	yellow	2.89255986	2.10E-35	2.77555401	1.56E-32
Slc23a1	skyblue	-1.622066	3.73E-06	-0.8994844	0.01412653
Etf1	orange	0.67646623	1.72E-06	0.49483613	0.00076104
Gfra3	skyblue	-2.283517	4.33E-05	-1.2917564	0.01957193
C2	orange	2.02505212	1.37E-21	1.80820036	5.65E-17
Epb41l4a	red	-0.9863455	3.31E-09	-0.694332	6.05E-05
Tslp	skyblue	-1.1445495	0.00080318	-1.6057872	8.93E-06
Proc	skyblue	-3.5638041	0.0001771	-2.2268493	0.01212049
Csnk2b	orange	0.73061185	2.26E-11	0.46606411	4.33E-05
Lims2	red	-0.9020336	0.00024354	-0.9154277	0.00027264
Aif1	black	4.8784305	9.26E-42	4.58583382	4.83E-36
Ltb	orange	1.66048059	1.17E-10	1.54148598	5.12E-09
Tnf	black	5.17196877	3.69E-30	3.20393407	1.32E-14
Riok3	orange	0.6458367	2.09E-09	0.45634969	4.41E-05
Psors1c2	skyblue	-1.8980118	0.00116824	-1.0520174	0.09369313
Aqp4	red	-1.4676966	2.05E-06	-0.9086295	0.00443407
Mrpl27	orange	0.59152821	9.72E-05	0.61198987	9.32E-05

Lama3	skyblue	-1.6676137	3.50E-07	-0.9588506	0.00533826
Ttc39c	orange	1.63143703	4.36E-17	1.35333328	7.13E-12
Spry4	orange	0.90182578	0.00013573	0.87067746	0.00033419
Cabyr	orange	2.07949765	2.07E-05	0.69161244	0.19660381
Nr3c1	red	-0.7137958	9.62E-08	-0.451628	0.0012424
Mrps18b	orange	0.75199421	0.00128006	0.71303942	0.0028161
Pcdh12	skyblue	-2.5822005	1.01E-22	-1.6276192	1.81E-09
0610009O20	red	-0.6613397	3.07E-05	-0.5119259	0.0018487
Arap3	red	-1.0195247	3.61E-08	-0.7919093	3.37E-05
Trim26	orange	0.8516767	2.01E-13	0.68999388	5.84E-09
H2-M5	skyblue	-1.7724663	0.00014104	-0.9615139	0.04477413
Myot	red	-1.0848355	0.00086083	-0.6540122	0.06019612
Dcp2	orange	0.92765855	2.98E-16	0.86870263	3.18E-14
Mal2	red	-1.0516509	2.41E-06	-0.5489853	0.02049839
Ap3s1	orange	0.62343485	0.0004463	0.57428454	0.00180783
Hbegf	orange	1.14520531	0.00019393	1.30860499	2.79E-05
Tcerg1	orange	0.90390256	3.04E-07	0.60631992	0.00099874
Ppp2r2b	skyblue	-1.8682335	3.04E-06	-1.2832002	0.00135555
Hsd17b4	red	-0.6656249	1.27E-06	-0.5334613	0.00017272
Rab27b	red	-1.0158587	2.09E-06	-0.7381858	0.00090468
Mbd2	orange	0.80200182	2.69E-07	0.7131043	8.21E-06
Sec11c	orange	0.69222312	6.15E-16	0.61692625	1.30E-12
Grp	red	-1.3053054	0.00042859	-0.7130922	0.07022881
Pmaip1	orange	1.42471646	4.64E-14	0.68955599	0.00051781
Impa2	orange	1.20209906	4.21E-05	1.21275686	5.66E-05
Cidea	skyblue	-3.1430831	0.00043235	-2.3814627	0.01053995
Lox	orange	2.06109172	5.07E-22	1.82227507	2.59E-17
Sncaip	skyblue	-1.7205482	8.51E-05	-1.8314948	4.43E-05
Ptpn2	orange	1.35563981	4.16E-19	1.26855251	1.28E-16
Cep192	orange	1.04568739	5.63E-15	0.88934316	7.05E-11
Me2	orange	0.92686083	1.58E-14	0.6673304	8.65E-08
Pcyox1l	red	-0.8742873	6.39E-12	-0.7715266	2.81E-09
Nars	orange	0.59708069	2.22E-05	0.52142624	0.00032775
Fech	red	-0.6159182	8.49E-08	-0.4164825	0.00050672
Lmnb1	yellow	2.08877091	2.69E-35	2.08365947	9.31E-35
C330018D20	red	-0.2223351	0.3313705	-0.7378507	0.00067446
Fbn2	skyblue	-2.2661357	0.00135711	-0.9261238	0.23723295
Cd74	orange	1.26209044	3.44E-14	1.03327037	1.22E-09
Tcof1	orange	1.12765344	1.15E-11	1.06104684	3.21E-10
Camk2a	skyblue	-2.256547	5.46E-09	-1.7306168	1.55E-05
Pdgfrb	red	-0.5853218	0.00211361	-0.3739231	0.06415961
Csf1r	orange	0.70299414	0.00609414	0.41920087	0.12714474
Psat1	orange	1.89543197	1.68E-26	1.67302856	9.83E-21

Cndp2	orange	1.67197334	2.21E-13	1.42464472	8.66E-10
Cyb5a	red	-0.9980745	1.81E-07	-0.8457205	1.72E-05
Scgb1a1	skyblue	-2.1567231	1.70E-08	-1.1431356	0.00458865
Anxa1	orange	0.8595003	1.78E-05	0.81086548	7.88E-05
Incenp	yellow	2.09062243	3.50E-23	2.17408263	9.20E-25
Rab3il1	orange	1.98746982	9.70E-16	1.8610749	9.10E-14
Fads2	red	-0.584923	0.00101907	-0.5157363	0.00498627
Cd5	yellow	2.67345559	5.91E-25	2.96530438	4.96E-30
Cd6	yellow	3.9278196	1.01E-44	3.95886855	3.92E-45
Ms4a7	black	4.68462702	9.44E-13	4.35025386	7.55E-11
Ms4a4c	black	5.99696033	6.10E-48	5.829883	2.39E-44
Ms4a6b	yellow	4.00905498	4.06E-45	3.70261893	1.26E-38
Ms4a6d	black	5.17471271	2.13E-19	4.69469714	5.02E-16
Mrpl16	orange	0.78638739	2.00E-05	0.42510625	0.03102756
Fam111a	orange	1.66101333	3.28E-15	1.36596577	2.64E-10
Lpxn	yellow	3.64544097	2.25E-23	3.42578153	1.75E-20
Ostf1	orange	0.64498621	9.49E-10	0.45255388	3.53E-05
Trpm6	red	-0.619189	0.0022015	-0.2329624	0.29937973
Ms4a8a	yellow	2.76636767	1.07E-13	2.47204158	5.23E-11
Ccdc86	orange	1.4739752	1.55E-07	1.40183118	1.06E-06
Slc15a3	yellow	3.05757683	3.82E-16	2.87768603	2.92E-14
Fen1	orange	1.27854803	0.00049719	0.86828917	0.02585589
Syt7	red	-1.1400379	2.72E-06	-0.7542262	0.00293358
Aldh1a7	skyblue	-1.6658607	3.15E-15	-1.1035108	4.24E-07
Lipo1	red	-0.4512849	0.00053897	-0.6216193	2.10E-06
Otub1	orange	0.62933345	3.38E-05	0.45854627	0.00374937
Cdc42bpg	red	-1.3837489	1.25E-06	-0.9911521	0.00083405
Fas	orange	0.5932394	0.00018702	0.22512779	0.19937565
Ak3	red	-0.8340729	9.43E-06	-0.8709673	5.61E-06
1700123I01R	yellow	2.93033701	2.01E-13	4.43468522	1.46E-21
Jak2	orange	1.12372335	7.58E-15	1.11660318	1.64E-14
Cdca5	yellow	3.12290191	8.52E-11	3.6910428	1.15E-13
Tnfrsf25	red	-1.2419683	2.43E-07	-1.0152967	3.89E-05
Kif20b	yellow	2.3627007	1.62E-21	2.65371112	2.18E-24
Vps51	orange	0.62837891	0.00755766	0.54647106	0.02237566
Htr7	yellow	3.70896994	3.94E-10	4.66110881	1.61E-13
Rpp30	orange	0.77652043	7.36E-06	0.56448958	0.00192002
Ankrd1	orange	1.69072301	2.05E-06	1.14622685	0.0020177
Pcgf5	orange	0.82963144	5.27E-10	0.61020457	1.06E-05
Mlana	skyblue	-0.7538376	0.25896592	-1.8801001	0.0034814
Gldc	orange	1.88700775	5.11E-07	0.62909177	0.12193749
Mrpl21	orange	0.60067319	0.0004677	0.40326909	0.02622545
Pola2	orange	1.10036144	7.58E-13	1.01299945	8.02E-11

Coro1b	orange	0.73270152	9.03E-07	0.51073426	0.00100953
Cabp4	yellow	3.7493299	1.35E-06	3.07923899	3.49E-05
Banf1	orange	0.68882862	0.00063902	0.60581167	0.00370052
Pitpnm1	orange	1.15528651	9.32E-10	0.90597429	3.07E-06
Pold4	orange	0.65430776	3.12E-06	0.27774521	0.07396877
Adrbk1	orange	0.87622	1.36E-14	0.75923778	5.35E-11
Acy3	red	-0.9346284	0.00179183	-0.757743	0.01476843
Doc2g	skyblue	-0.5351204	0.30567337	-2.2496585	4.75E-06
Rin1	orange	1.26026003	1.36E-06	1.53797504	4.37E-09
Pcx	red	-1.42332	5.74E-07	-1.001498	0.00070725
Papss2	red	-0.6633453	0.00183384	-0.660987	0.00240146
Peli3	skyblue	-1.9384891	1.01E-08	-1.5717952	5.46E-06
Ctsw	yellow	4.03752749	2.50E-44	3.44515101	2.03E-32
Smarca2	skyblue	-1.5118119	1.05E-13	-1.3183743	1.87E-10
Vldlr	red	-0.8068849	0.00076608	-0.826993	0.00075888
Slc1a1	red	-1.0442042	9.82E-08	-1.0765767	8.27E-08
Ehbp1l1	orange	0.50683702	0.0021429	0.65972777	7.72E-05
Ltbp3	red	-1.1123346	2.03E-09	-0.7612263	7.75E-05
Prdx5	orange	1.07972266	3.67E-06	0.89550341	0.00020025
Vegfb	red	-1.1428922	1.16E-08	-1.0779036	1.39E-07
Fermt3	yellow	2.54290387	4.32E-23	2.3665363	5.60E-20
Rcor2	red	-1.091668	0.00081389	-0.4763488	0.17398597
Lgals12	skyblue	-2.7110847	4.77E-06	-2.0236821	0.00086309
Gpam	red	-0.7768091	8.00E-05	-0.2227218	0.31560671
Cep55	yellow	3.57569098	1.01E-35	4.03125298	2.75E-37
Rbp4	skyblue	-2.4187972	1.87E-10	-1.5637385	6.25E-05
Plce1	red	-1.3314327	7.68E-08	-0.901951	0.00047101
Hells	yellow	2.61528751	3.29E-18	2.28958646	1.17E-13
Aldh18a1	orange	1.52991979	4.48E-12	1.51850351	1.01E-11
Tll2	skyblue	-4.4731844	7.02E-05	-1.8192171	0.08294389
Pik3ap1	yellow	2.66819662	8.49E-13	2.44972446	9.56E-11
Lcor	orange	0.601381	0.2162954	1.33579459	0.00527732
Mxi1	red	-1.0819374	7.11E-06	-1.1158809	5.50E-06
Add3	red	-0.5373454	0.00055715	-0.5916274	0.00019313
Xpnpep1	orange	0.73443313	4.35E-17	0.54694108	9.56E-10
Arl3	red	-0.7866665	2.57E-08	-0.7091046	1.05E-06
Maoa	red	-1.2024507	1.28E-16	-1.0538987	1.03E-12
Nt5c2	orange	0.59838172	0.00015701	0.45676902	0.00551077
Msr1	black	5.97567641	2.20E-18	5.37127985	4.41E-15
5430427O19	yellow	3.3208908	1.04E-14	2.8108081	8.11E-11
Gk	orange	1.00829138	0.00045621	0.80318305	0.0073869
Col17a1	red	-1.0930893	2.98E-05	-1.1893583	8.37E-06
Casp7	orange	0.77662225	2.11E-07	0.93984632	4.19E-10

Nhlrc2	red	-0.7952308	3.61E-11	-0.6167805	5.62E-07
Gfra1	skyblue	-2.1612364	2.27E-10	-1.1977205	0.00063307
Hspa12a	red	-1.0648587	3.54E-09	-0.7372873	7.36E-05
Slc18a2	red	-0.4417467	0.22666873	-1.1783014	0.0009477
Gcgr	skyblue	-2.1490186	0.00835587	-0.7370154	0.37619671
Arhgdia	orange	0.62953722	0.00038611	0.55660702	0.00234005
Alyref	orange	1.01792532	5.86E-09	0.83480608	3.54E-06
Lrrc45	red	-0.8197553	6.53E-06	-0.6202964	0.0009865
Cbr2	skyblue	-2.5228025	2.03E-06	-1.6353263	0.00323872
Fasn	skyblue	-1.4262264	0.00010436	-1.4653612	9.56E-05
Arhgap19	orange	1.44426206	1.48E-10	1.410987	7.94E-10
Slc16a3	orange	0.86521259	0.00021681	1.01871642	1.92E-05
Cd7	yellow	2.34135521	3.80E-19	2.39138387	4.10E-19
Sectm1a	black	7.18358726	3.53E-21	9.15129249	1.92E-12
Rab40b	red	-1.3992077	4.30E-06	-0.5096285	0.11413624
Ubtd1	orange	0.68742434	0.00011914	0.76883499	2.35E-05
Fn3k	skyblue	-3.4071157	1.96E-07	-1.789384	0.00694634
Hoga1	orange	0.70994838	6.66E-05	0.76481939	2.63E-05
Loxl4	orange	0.7084516	0.01802563	1.07904809	0.00035416
Hps1	orange	0.71541758	2.20E-05	0.70828191	4.18E-05
Entpd7	orange	0.77428058	0.00039561	0.92042867	3.51E-05
Dnmbp	red	-0.6014467	1.34E-07	-0.3821874	0.00129302
Cpn1	skyblue	-1.9783985	4.15E-11	-1.1212972	0.00026425
Cyp2c44	skyblue	-3.2919495	0.0003309	-2.7214186	0.00098479
Scd2	red	-1.1739436	0.00142471	-0.9173911	0.01672506
Peo1	red	-0.7454619	1.60E-09	-0.4227087	0.00103874
Kazald1	skyblue	-1.6309947	2.21E-09	-1.3064429	3.02E-06
Nfkb2	orange	1.41191491	2.34E-12	1.35331674	3.11E-11
Fam120c	red	-1.1607846	4.38E-07	-0.6894299	0.00393374
Apex2	orange	0.59942529	0.00019841	0.30297261	0.0799365
Tro	skyblue	-1.8752689	2.06E-07	-1.5976968	1.17E-05
Flnb	orange	1.22214686	1.17E-06	1.19900984	2.97E-06
Dnase1l3	yellow	2.87936978	3.25E-11	2.35871683	1.05E-07
Sat1	orange	0.95973647	8.98E-11	0.6874253	7.07E-06
Acot9	orange	0.77127194	3.99E-12	0.69747733	7.60E-10
Ptprj	orange	0.70280023	0.00017168	0.65835543	0.00062591
Banp	red	-0.7788183	2.68E-05	-0.6849594	0.00034896
Atp10a	orange	1.1928809	3.85E-11	1.04842777	1.05E-08
Padi4	orange	0.61589925	0.09021588	1.35512361	0.00014287
Rdh5	red	-1.1638652	4.35E-05	-0.8483288	0.00397459
Cd63	orange	0.84591815	0.00056594	0.83047069	0.00097026
Mmp19	orange	1.09102352	0.00171323	0.81433944	0.02526513
Rps26	orange	0.70987342	5.49E-13	0.589841	4.64E-09

Pa2g4	orange	0.69285205	1.00E-05	0.55262949	0.00066726
Esyt1	orange	1.03603606	5.61E-08	0.84515205	1.70E-05
Nabp2	orange	0.594568	0.00019107	0.3367656	0.0479603
Aatk	red	-0.6393457	1.45E-05	-0.3251335	0.03899809
Il23a	yellow	3.42116677	0.00024605	3.27442352	0.00021492
Prim1	orange	1.83082079	1.40E-21	1.2465045	2.41E-10
Nab2	orange	0.64895266	0.00010816	0.69213928	5.48E-05
Shmt2	orange	0.89362259	2.99E-11	0.75718596	3.87E-08
Gli1	red	-1.3733257	3.65E-06	-0.7269699	0.02015234
Katnal2	red	-1.0194119	0.00348474	-0.3068408	0.42944803
Agap2	yellow	2.85731659	1.06E-32	2.73764491	7.81E-30
Pstpip2	orange	0.70073322	0.02927222	1.13786771	0.00037839
Crisp1	red	-1.5971823	0.00144872	-0.6461752	0.23432108
Avil	red	-0.6594388	0.00695188	-0.8049385	0.00120855
Paox	orange	0.60443634	0.00053149	0.42528512	0.02056676
Adam8	yellow	2.3305673	5.14E-06	1.89193498	0.00034151
Inpp5a	red	-0.6114923	4.52E-06	-0.6159124	6.02E-06
Psmd13	orange	0.83201192	6.00E-11	0.60481883	4.31E-06
Cox8b	skyblue	-2.1427324	2.08E-13	-1.907707	2.52E-10
Ifitm5	orange	0.95566809	0.00920152	0.22954166	0.59052005
Ifitm1	orange	1.26307875	5.20E-12	0.84267689	9.07E-06
Ifitm3	yellow	2.14252192	1.95E-35	2.0909679	9.97E-34
Drd4	yellow	3.60831636	0.00011975	4.47426418	0.00011496
Irf7	black	5.32734858	1.88E-98	5.0690693	3.24E-89
Pidd1	yellow	1.90822049	1.35E-17	2.14293092	1.29E-21
Rplp2	orange	0.66753382	1.56E-07	0.54082617	3.81E-05
Tmem192	orange	1.18121756	1.05E-07	0.86436872	0.0001939
Asl	orange	0.60946349	4.91E-06	0.51721842	0.00018073
Gusb	orange	0.73472942	0.00056215	0.44467273	0.04975381
Phkg1	skyblue	-2.0219738	6.78E-05	-2.1820177	2.60E-05
Clybl	red	-0.8667321	6.53E-09	-0.756746	1.34E-06
Tk1	yellow	2.09514147	3.80E-25	1.95139297	1.34E-21
Rbfox3	red	-0.2449971	0.60625381	-1.3331037	0.00181078
Cbx2	red	-0.7954643	0.0002847	-0.8870476	6.01E-05
Cbx8	red	-0.7261558	0.00011203	-0.7255707	0.00020023
Gaa	red	-0.7458441	1.83E-08	-0.5988407	1.16E-05
Nptx1	skyblue	-3.5238886	5.17E-49	-2.4432773	5.47E-25
Cpeb1	red	-0.6162721	0.00021437	-0.6029109	0.00039165
Tma16	orange	0.80087167	0.00194604	0.96128621	0.00026009
Map3k7cl	skyblue	-1.679039	2.68E-10	-0.8148578	0.00326568
Hprt	orange	0.6874734	3.55E-05	0.41084077	0.01958429
Shisa5	orange	1.26431729	1.96E-18	1.17243288	7.89E-16
Col7a1	red	-0.9970846	5.35E-05	-0.4891725	0.06525863

Arhgef9	red	-1.2471431	2.04E-06	-0.9149122	0.00064337
Cnksr2	skyblue	-1.7830518	0.00363062	-0.6836736	0.27951202
Rps6ka6	skyblue	-0.9489117	0.08815324	-1.8264137	0.00119507
Tmem47	red	-1.0551702	5.72E-09	-0.7310726	1.00E-04
March8	red	-0.6297631	3.52E-11	-0.4414352	7.30E-06
0610011F06l	red	-0.8214444	1.63E-05	-0.6426324	0.00119278
Fam195a	red	-0.799202	9.38E-05	-0.9558348	7.35E-06
Jmjd8	red	-1.1181285	4.48E-11	-0.7830238	7.71E-06
Fbxl16	red	-1.1695655	1.57E-05	-0.6698622	0.01681535
Prps2	red	-0.6391837	0.0031096	-0.7225972	0.00103315
Sdc3	orange	0.78731901	0.00030159	0.60774026	0.0072998
Il6	black	5.14651871	5.87E-15	7.85106891	2.46E-16
Tyms	orange	1.56543491	7.73E-11	1.45618832	3.32E-09
Hspa4l	red	-1.4521266	5.24E-15	-1.0097167	1.07E-07
Plk4	orange	1.28490498	1.64E-07	1.54829093	7.64E-10
Jade1	red	-0.862817	1.12E-06	-0.7433475	4.56E-05
D3Ertd751e	red	-1.1147924	5.69E-05	-1.1023993	0.00010058
Itih5	red	-1.1453355	0.00012131	-0.7922228	0.01101929
Clec3b	skyblue	-2.3834738	6.87E-19	-1.5484355	2.32E-08
St8sia2	skyblue	-1.9866759	1.88E-06	-1.2980551	0.00271131
Slc25a10	red	-0.6219736	4.38E-05	-0.5132599	0.00110065
Rassf3	red	-1.2226318	8.94E-12	-0.9274823	4.95E-07
Ccr1	yellow	4.05551869	1.85E-24	4.26585541	9.57E-27
Nrp1	red	-1.0701272	6.34E-11	-0.8670343	2.44E-07
Homer2	red	-1.48746	7.56E-07	-0.7071711	0.02868853
Dhtkd1	skyblue	-1.5779654	0.00385837	-1.1951891	0.03305597
Pdgfa	red	-1.0624068	5.04E-11	-0.8734262	1.49E-07
Cplx2	red	-0.8641203	2.39E-06	-0.5068064	0.00821226
Nop16	orange	0.56861196	0.00257934	0.63641983	0.0009939
Tspan17	red	-0.6521435	1.69E-08	-0.6123627	2.36E-07
Hk3	black	5.29987359	7.24E-52	5.39745103	2.78E-53
Smad7	red	-0.6530414	1.06E-08	-0.5676283	1.15E-06
Myo5b	red	-0.7592208	0.00322209	-0.3897799	0.16242991
Casp12	orange	0.79178514	1.18E-10	0.8218485	2.91E-11
Casp1	yellow	2.30347932	2.44E-15	2.21885419	4.93E-14
Kbtbd3	red	-1.3994513	1.60E-05	-0.7309871	0.03616911
Rp1	skyblue	-2.9528495	2.43E-12	-2.0158346	1.97E-06
Sox17	red	-0.7080303	3.06E-07	-0.7976252	1.20E-08
Adhfe1	red	-0.9686714	0.00031801	-0.8222181	0.00304561
Ppp1r42	skyblue	-2.3096684	0.00068642	-1.8662993	0.0062426
Stau2	red	-0.8438966	1.37E-05	-0.7474462	0.00018159
Rdh10	red	-0.8101501	3.51E-11	-0.3247939	0.01248189
Paqr8	red	-0.7795479	1.85E-07	-0.3082373	0.05795071

Eya1	skyblue	-1.5447085	5.63E-08	-0.8604119	0.00508614
Tmem14a	skyblue	-1.4544303	3.57E-08	-1.5694594	6.85E-09
Gsta3	skyblue	-1.4814738	0.00031136	-1.2333831	0.00369034
Gm4956	skyblue	-1.6453443	0.00014152	-1.4244167	0.00138858
Slco5a1	skyblue	-1.5937219	5.71E-08	-1.3020011	3.78E-05
Idh1	red	-0.8740345	1.07E-09	-0.5524938	0.00021152
Mdh1b	skyblue	-2.4752431	6.89E-08	-1.3600507	0.00348952
Eef1b2	orange	0.65066685	1.01E-06	0.44351876	0.00139985
Rftn2	red	-1.3069251	3.21E-05	-1.0994337	0.00068976
Wdr75	orange	0.58218544	0.00011412	0.38551147	0.01522452
Ikzf2	orange	1.31250301	4.42E-07	0.91760926	0.00066411
Lancl1	red	-0.7014246	4.45E-07	-0.4449002	0.00219887
Icos	black	5.61394466	3.47E-39	5.12221125	1.76E-34
Ctla4	black	7.18181704	2.45E-52	6.78987048	6.07E-53
Cd28	yellow	3.57918367	2.72E-10	3.11495247	4.49E-08
Wdr12	orange	0.6754628	0.00040887	0.7057073	0.00033135
Nop58	orange	1.32105864	2.02E-11	1.10500007	4.69E-08
Stradb	red	-0.7130409	0.00486065	-0.4564838	0.09036791
Casp8	orange	1.48927519	3.39E-14	1.28301867	1.42E-10
Cflar	orange	0.62621064	8.11E-08	0.56344349	2.43E-06
Sgol2a	yellow	2.39210644	4.52E-10	2.6267161	4.97E-10
Col5a2	orange	0.72082847	0.02470606	0.8735143	0.00715021
Col3a1	orange	0.61430051	0.06933169	1.06641572	0.00139667
Tex30	orange	1.23626026	2.11E-09	0.91914024	3.00E-05
Slc9a2	skyblue	-1.708381	5.45E-05	-0.9113445	0.03917126
Il18rap	yellow	3.36987945	1.38E-42	3.37395558	2.36E-42
Il1rl1	orange	0.85765175	4.03E-06	0.81144459	2.63E-05
Il18r1	orange	0.96040245	1.10E-07	1.03732171	1.45E-08
Il1r2	yellow	2.29171409	1.89E-08	2.55842546	5.67E-10
Chst10	orange	1.06545343	0.00583843	1.79548898	5.43E-06
Mrpl30	orange	0.63161319	0.00016032	0.52298236	0.00264051
Mitd1	orange	1.90508092	7.05E-30	1.6136289	1.60E-21
2010300C02	red	-1.1897817	5.28E-09	-0.7852273	0.00020085
Stk17b	orange	1.56459702	4.47E-20	1.34954037	6.57E-15
Pms1	orange	0.96143389	0.00583216	0.94650754	0.0083065
Inpp1	orange	1.00426287	1.06E-22	0.88720263	8.68E-18
Stat1	yellow	2.32873497	4.33E-39	2.40935317	1.12E-41
Nabp1	orange	0.81749064	1.68E-10	0.80881323	5.40E-10
Tmeff2	skyblue	-2.0440867	1.39E-18	-1.5916234	2.65E-11
Mgat4a	orange	1.09831693	7.51E-10	0.88277356	1.44E-06
Zap70	yellow	3.02392723	2.17E-66	2.80664237	6.30E-57
Sema4c	orange	0.89498818	5.50E-07	0.83704798	4.61E-06
Ptpn18	orange	0.66048375	1.06E-05	0.52588527	0.00078284

Prim2	orange	1.38020337	1.61E-15	1.12869962	2.45E-10
Ogfrl1	orange	0.937649	3.48E-06	0.52634882	0.01399719
Ccl20	black	6.18444999	1.90E-11	5.87201685	4.83E-10
Cyp27a1	red	-0.7402943	0.00181734	-0.6706964	0.00620267
Plcd4	red	-1.4808337	3.92E-06	-0.7035208	0.04665444
Rqcd1	orange	0.76789376	2.43E-06	0.56794296	0.00079801
Slc11a1	yellow	3.29930458	4.90E-10	3.16175883	4.26E-09
Pnkd	red	-1.1881747	7.11E-07	-1.1129667	5.77E-06
Cxcr2	yellow	2.61261386	5.74E-12	2.29293377	1.22E-09
Igfbp5	skyblue	-1.5133566	1.11E-17	-1.2320793	7.55E-12
Bard1	yellow	2.56381041	1.37E-11	2.21818638	9.05E-09
Ankzf1	red	-0.4909014	0.00248536	-0.6278775	0.00012892
Dnajb2	red	-0.7936104	4.87E-09	-0.6002222	1.87E-05
Speg	red	-0.9743217	4.49E-09	-0.7520702	1.18E-05
Des	red	-0.934743	4.66E-06	-0.5387731	0.01238949
Obsl1	red	-0.699788	0.00043913	-0.6831504	0.0008265
Sp100	orange	1.87182585	4.11E-79	1.81315972	5.16E-74
2810459M11	skyblue	-2.8067755	0.00609757	-1.7946641	0.0786753
Ncl	orange	1.00168203	1.35E-09	0.80875274	1.92E-06
Nmur1	skyblue	-1.9661086	0.00014626	-1.9820088	0.00026723
Eif4e2	orange	0.60161175	4.66E-05	0.51685738	0.0007177
Ngef	skyblue	-1.7421997	1.86E-11	-1.067346	7.20E-05
Gpr35	yellow	3.11133302	2.24E-21	2.97017473	1.88E-19
Pask	orange	1.58216173	2.36E-06	1.4561459	2.04E-05
Bok	red	-0.6311034	0.00222414	-0.5560771	0.00901121
Dtymk	orange	0.58256219	6.38E-05	0.37367637	0.01534389
Pdcd1	black	7.7919514	1.81E-66	6.52463904	6.92E-55
Inpp5d	orange	1.20629827	7.40E-14	0.93195177	1.73E-08
Iqca	skyblue	-2.8365373	1.00E-07	-2.0325614	0.00019311
Rab17	skyblue	-1.6395037	4.33E-05	-1.5109591	0.00039259
Lrrfip1	orange	0.53755422	0.00113006	0.65540125	9.08E-05
Scly	orange	0.80837273	1.05E-13	0.62407858	2.23E-08
Klhl30	skyblue	-1.3865203	6.85E-06	-1.3796003	2.40E-05
Tnfrsf11a	yellow	2.24665001	1.54E-07	1.86091378	2.43E-05
Pam	skyblue	-1.2943523	0.00055278	-1.6165926	2.03E-05
Actr3	orange	1.43961013	5.61E-15	1.23519889	4.36E-11
Tmem163	red	-0.7483763	0.01360208	-1.0095418	0.0009654
Mcm6	orange	1.62826153	4.80E-27	1.37812571	1.85E-19
Rgs1	black	5.30560631	5.21E-28	4.17088832	1.77E-19
Nifk	orange	0.7352035	6.65E-05	0.68850407	0.00029907
Epb41l5	red	-1.0479853	3.77E-09	-0.6875393	0.00020551
Ptpn4	red	-0.7479781	0.000161	-0.7179821	0.000474
Steap3	skyblue	-1.5504105	5.73E-32	-1.3743694	7.05E-25

Ptprc	yellow	2.47692632	6.67E-54	2.50500815	7.15E-55
Cd55	red	-1.4643144	4.59E-17	-1.0172232	1.29E-08
Pigr	orange	-0.3751016	0.10855447	0.70480807	0.00194957
Il24	black	6.76803021	6.33E-05	5.75051364	0.00102342
Gpr37l1	skyblue	-2.0504378	7.25E-05	-1.225091	0.0267181
Ube2t	orange	1.6148251	1.91E-10	1.72415428	5.78E-11
Rassf5	orange	1.21680704	6.17E-13	1.09902311	1.62E-10
Rab29	orange	0.79579542	5.85E-08	0.58957981	0.00012202
Lrrn2	red	-1.2974065	0.00030491	-0.9251776	0.01483816
Pik3c2b	red	-0.9462238	8.24E-11	-0.5043921	0.00100761
Ppfia4	orange	2.0443587	2.81E-09	1.6645321	2.75E-06
Myog	skyblue	-2.8510376	8.55E-05	-2.4386996	0.0032624
Tor1aip1	orange	0.86473268	3.23E-14	0.7023157	1.67E-09
Mr1	red	-0.5994919	7.97E-06	-0.4954256	0.00031733
Rgs16	yellow	3.93351057	8.99E-38	4.03773943	5.76E-38
Ncf2	yellow	2.09406691	2.79E-13	1.81508386	4.97E-10
Adck3	red	-1.3410772	1.40E-06	-1.1379529	7.32E-05
Cdc42bpa	red	-0.8424203	2.22E-06	-0.71604	9.31E-05
Tfb2m	red	-0.7249191	4.77E-07	-0.7570916	2.64E-07
Kif26b	skyblue	-1.9791958	1.25E-12	-1.6856321	3.17E-09
Efcab2	red	-1.1696101	1.83E-05	-1.1882314	2.19E-05
Trp53bp2	red	-1.0796359	1.14E-09	-0.8245944	6.53E-06
Srp9	orange	0.64930833	1.06E-07	0.41067823	0.00136079
Rgs7	skyblue	-3.0217304	4.48E-05	-2.6577289	0.0008621
Spta1	skyblue	-0.9076004	0.1194254	-2.4195927	0.00015851
Mnda	black	5.61382305	1.03E-45	5.2648848	3.65E-40
Cfap45	red	-0.7714071	3.93E-05	-0.3100802	0.12846313
Slamf9	yellow	3.41767715	2.69E-09	2.83731484	1.51E-06
Vangl2	red	-0.8227055	2.18E-06	-0.6095122	0.0007329
Uck2	orange	0.71033526	0.00018333	0.78335622	5.18E-05
Dusp27	skyblue	-1.3769567	1.46E-08	-1.1497931	1.10E-05
Mpc2	red	-0.5466377	0.00012744	-0.593804	5.02E-05
Xcl1	black	4.55110773	1.64E-15	4.5739231	3.33E-12
Dpt	red	-1.1303013	7.50E-12	-0.8628518	3.83E-07
Atp1b1	red	-0.6066488	0.00045799	-0.2456016	0.19697203
Ccdc181	red	-0.6925333	0.00014273	-0.5899278	0.0016643
Selp	yellow	2.5947715	1.46E-22	3.02212729	6.23E-30
Sell	yellow	2.10996344	1.17E-38	2.37623214	2.86E-48
Sele	orange	1.99826512	0.00055678	1.74481284	0.00288814
Scyl3	orange	0.66343882	5.80E-06	0.57122406	0.00013499
Kifap3	red	-0.9293088	3.14E-08	-0.9079071	1.14E-07
Prrx1	red	-0.8908372	2.52E-07	-0.5729234	0.00159921
Sec16b	red	-0.7778467	1.15E-05	-0.6234548	0.00075317

Abl2	orange	1.08313838	4.46E-08	0.87915717	1.65E-05
Cenpf	yellow	2.67631248	1.21E-20	3.28665498	8.60E-28
Esrrg	skyblue	-2.0118556	4.19E-05	-2.3243479	4.17E-06
Spata17	skyblue	-1.48003	0.00011257	-1.1898875	0.00198098
Cr2	skyblue	-1.7924005	0.00333565	-1.5642635	0.01605409
Nek2	orange	1.27255046	1.54E-05	1.38369751	3.61E-06
Tmem206	red	-0.5683534	0.00063448	-0.697171	5.26E-05
Atf3	yellow	3.25820199	4.42E-85	2.4898596	5.38E-52
Batf3	yellow	2.82267153	4.57E-22	2.71445074	1.08E-19
Irf6	red	-0.8940074	8.39E-06	-0.5100234	0.01611685
Plxna2	orange	0.79759206	3.26E-07	0.58503095	0.00029611
Suv39h2	orange	1.51904189	0.00018444	1.32128585	0.00157488
Dclre1c	orange	1.42770999	7.02E-15	1.18064013	2.24E-10
Cfap126	skyblue	-1.6986369	1.02E-13	-1.1015356	2.55E-06
Meig1	skyblue	-2.1030198	9.95E-11	-0.9914455	0.00097495
Fam107b	orange	0.84026107	1.89E-09	0.87782756	5.76E-10
Fcgr2b	yellow	3.16646103	8.33E-27	3.10827507	1.00E-25
Frmd4a	red	-0.4803047	0.00081305	-0.5893313	5.02E-05
Mcm10	yellow	2.93317826	5.53E-25	3.36039899	8.65E-30
Ddr2	red	-0.7820304	5.22E-14	-0.4652647	1.81E-05
Hsd17b7	orange	0.96374508	0.00066086	0.9528712	0.00102349
Ccdc3	skyblue	-2.6552639	5.31E-09	-1.4028946	0.00163211
Rgs5	skyblue	-1.5936439	1.73E-08	-1.5401776	9.84E-08
Enkur	skyblue	-1.4273852	6.93E-06	-1.507766	3.31E-06
Nuf2	yellow	3.26258501	8.73E-25	3.60072001	6.45E-27
Fmo3	skyblue	-1.8234105	1.93E-05	-0.9065679	0.04674546
Myoc	skyblue	-1.8385897	0.00017216	-2.3781194	2.33E-06
Prdx6	red	-0.6417354	0.00468063	-0.5350455	0.02323841
Cenpl	orange	1.77346022	2.91E-22	1.41048272	2.63E-14
Tnn	skyblue	-2.3701346	1.22E-12	-1.5356453	5.12E-06
4930562F07l	yellow	2.37306878	0.00378877	1.60326307	0.01854755
Pip4k2a	orange	0.91649844	9.88E-08	0.74814316	2.57E-05
Plxdc2	skyblue	-1.3723887	5.29E-22	-1.1530881	1.19E-15
Nek6	orange	1.2222891	8.16E-07	1.30536162	2.11E-07
Psmb7	orange	0.78743192	4.58E-05	0.68775242	0.00056215
Lypd6b	red	-0.8744081	0.00127437	-0.5043473	0.07734984
Fam188a	orange	0.61254491	7.05E-08	0.54107747	3.67E-06
Itga8	skyblue	-2.1339515	6.22E-09	-1.4730836	0.00011115
Il2ra	yellow	4.36457139	2.42E-64	4.21532681	3.17E-59
Pfkfb3	orange	1.30389468	4.25E-18	1.13467515	9.17E-14
Prkcq	orange	0.97800393	7.58E-08	1.08821953	3.56E-09
Mastl	yellow	2.52553286	1.45E-07	2.77153768	4.65E-08
Pdss1	orange	0.92606447	0.00058386	0.93493246	0.00075317

Pkn3	red	-0.6562704	4.46E-05	-0.4671242	0.00540602
Apbb1ip	orange	2.00650728	6.29E-17	1.78911152	1.90E-13
Lrsam1	red	-0.7922173	4.95E-09	-0.541182	0.00011694
Ddx31	orange	0.67707951	8.11E-06	0.49288588	0.00189613
Ak8	skyblue	-1.9232243	1.15E-06	-1.1312878	0.00507573
1700026L06F	skyblue	-1.7416928	3.39E-14	-1.3180951	8.28E-09
St6galnac6	red	-0.7694265	4.19E-11	-0.7654926	1.16E-10
Eng	red	-0.6012699	3.09E-07	-0.5280559	1.19E-05
Ak1	red	-1.319115	7.83E-17	-1.0103786	5.31E-10
Slc25a25	orange	1.00549229	2.26E-06	0.96706453	8.64E-06
Lcn2	yellow	3.65905501	3.15E-68	3.17019467	2.53E-51
Dnm1	red	-0.8283729	2.69E-06	-0.7941341	1.16E-05
Galnt5	skyblue	-2.1059823	7.44E-05	-0.7972734	0.14721002
Ermn	black	6.04321859	2.37E-05	5.48439769	0.00019125
1700007K13I	skyblue	-2.1294201	5.03E-13	-1.5042772	6.57E-07
Cytip	orange	1.50269343	4.21E-32	1.23566423	7.48E-22
Acvr1c	skyblue	-2.5943883	0.00278352	-1.0951938	0.23505792
Upp2	skyblue	-1.6522781	4.48E-09	-1.4127576	9.84E-07
Crat	red	-0.8971858	3.48E-10	-0.7247893	8.08E-07
Ntmt1	orange	0.53487184	0.00571894	0.59761332	0.00251806
Fam73b	red	-0.8011165	3.22E-06	-0.5282909	0.00328117
Kynu	orange	1.7366438	2.34E-05	1.39107696	0.00085839
Cutal	skyblue	-2.1402377	3.39E-11	-1.4514886	1.18E-05
Phf19	orange	0.7553749	3.39E-05	0.7730224	3.87E-05
Hc	skyblue	-2.2697397	0.00798853	-1.3212073	0.15046723
Gsn	red	-1.2529339	1.64E-05	-0.9521612	0.00159685
Dab2ip	red	-0.962565	3.20E-08	-0.5963462	0.00103923
Ttll11	orange	0.30604702	0.31207267	0.99126469	0.00059813
Grb14	red	-1.1247838	4.01E-05	-0.5459841	0.06367628
Lhx6	orange	1.04318169	4.82E-08	1.32808082	1.45E-11
Ifih1	yellow	1.99664093	1.82E-20	1.9962778	2.31E-20
Slc4a10	skyblue	-3.7939623	0.00274288	-0.7818859	0.50811705
Psmd14	orange	1.1803096	2.02E-10	0.9357642	9.87E-07
Strbp	red	-0.7251425	1.09E-07	-0.5164226	0.00024731
Brd3	red	-0.61983	0.00010605	-0.5327709	0.00125079
Lcn4	yellow	3.19963517	1.62E-05	3.24945915	0.00021638
Agpat2	red	-0.6571265	0.00180635	-0.560407	0.01010329
Card9	orange	0.94906736	4.08E-08	0.87953301	5.97E-07
Gpsm1	red	-0.6533491	0.00180313	-0.6761782	0.00155851
Tmem141	red	-0.6611896	0.00571717	-0.4378471	0.08544177
Traf2	orange	0.66119645	1.17E-11	0.61248697	7.67E-10
Nmi	orange	2.02184037	1.19E-36	1.8377808	2.97E-30
Neb	red	-0.9746073	0.0032317	-0.5396036	0.13032403

Sapcd2	yellow	2.25668346	3.02E-12	2.7296409	2.24E-16
Lrrc26	red	-1.2517954	4.81E-07	-0.6653385	0.01115202
Rbms1	orange	0.64349473	5.28E-06	0.48075977	0.00105791
Itgb6	orange	1.41414117	2.65E-06	1.62516963	9.97E-08
Zmynd19	orange	0.76830484	8.10E-05	0.84256737	2.31E-05
Psd4	yellow	2.21100081	9.08E-27	2.05239577	4.69E-23
Ly75	orange	1.21082266	4.52E-10	1.42947335	3.08E-13
Il1rn	black	4.5362517	7.66E-30	4.53418203	1.50E-29
Il1f5	red	-1.1819998	0.00944699	-0.6847258	0.15911466
Il1f6	skyblue	-1.8035572	9.22E-05	-0.9170043	0.06135728
Hnmt	red	-1.0117047	0.00089167	-1.037336	0.00096017
Wdsub1	red	-0.9330898	3.56E-08	-0.7174835	3.98E-05
Dusp19	red	-0.5893647	0.00066148	-0.6054286	0.00079041
Frzb	skyblue	-1.595957	0.00031248	-0.8753056	0.06344341
Itga4	yellow	1.94476345	7.25E-26	2.04887702	2.82E-28
Cybrd1	skyblue	-2.1142513	2.77E-12	-2.1117183	4.82E-12
Zfp385b	red	-1.3248345	0.00474208	-1.0892627	0.02575576
Hat1	orange	1.41957337	4.96E-10	1.06754692	6.07E-06
Xirp2	skyblue	-1.8908941	9.77E-07	-1.3314879	0.00093952
Stk39	orange	1.13856633	2.31E-07	0.7021307	0.00234654
Cers6	orange	1.24843189	1.30E-10	1.37977899	1.44E-12
Timm10	orange	0.71075594	0.00363769	0.69787777	0.00586624
Smtnl1	red	-1.2041125	0.00277446	-0.6358823	0.14531556
Ube2l6	yellow	2.24248144	8.06E-32	2.02487774	5.30E-26
Clp1	orange	0.67707893	1.22E-06	0.4408802	0.00255173
Tfpi	red	-0.5974701	3.35E-05	-0.5831219	7.82E-05
Hoxd8	red	-1.1796596	0.00023242	-0.8508856	0.01573086
Chrna1	red	-0.9623802	8.10E-05	-0.4288344	0.13850723
Ola1	orange	0.78891918	0.00010749	0.6061438	0.0043131
Kif18a	orange	1.38892554	0.00012537	1.30703074	0.000474
Lpcat4	orange	0.60411323	1.48E-06	0.38120765	0.00383903
Ccdc34	orange	1.02658183	1.44E-05	0.50603958	0.04786208
B230118H07	red	-0.7260319	0.00053483	-0.6249504	0.00403687
Depdc7	red	-0.9881848	0.0019641	-0.8099521	0.02268116
Elf5	skyblue	-1.4895116	1.33E-06	-1.1079713	0.00052411
Cat	skyblue	-1.5393353	4.53E-07	-1.2195191	0.00010917
Pamr1	skyblue	-2.3334534	3.42E-42	-1.8223541	6.27E-26
Gatm	yellow	3.57173147	1.20E-17	3.46161759	2.60E-16
Dut	orange	1.54729606	7.19E-30	1.39204415	4.79E-23
Fbn1	orange	1.13786734	0.00016104	1.43828481	2.32E-06
Fgf7	red	-0.953474	7.33E-07	-0.773973	9.64E-05
Meis2	red	-0.7096721	1.30E-05	-0.4488948	0.00850731
Tspan18	skyblue	-1.8639797	4.44E-13	-1.5530431	3.32E-09

Slc28a2	yellow	3.28417913	2.67E-20	3.3523582	8.00E-21
Syt13	yellow	-1.5351684	0.18553218	3.32845227	0.00337089
Chst1	red	-0.8738108	3.91E-07	-0.5709205	0.00148888
Mapk8ip1	red	-1.3777559	6.16E-10	-0.8657852	0.00017476
Sord	skyblue	-2.1970714	3.65E-11	-1.7165632	4.66E-07
Creb3l1	orange	1.52375151	4.79E-10	1.54309743	4.69E-10
Frmd5	skyblue	-1.4628532	0.00350695	-1.2004382	0.02069082
Wdr76	orange	1.12510663	4.71E-11	1.03889934	3.62E-09
Map1a	red	-1.2108422	9.71E-14	-0.3578464	0.0438651
Pacsin3	red	-0.8051267	3.07E-06	-0.5454864	0.00256139
Mkks	skyblue	-1.5013394	2.89E-10	-1.5686946	1.28E-10
Jag1	red	-0.7709761	3.72E-05	-0.4561981	0.02049839
Slx4ip	orange	0.47556569	0.04816868	0.69482109	0.00388331
Ehd4	red	-0.7519104	4.70E-09	-0.7450818	1.05E-08
Itpka	red	-0.7927714	1.28E-06	-0.3791122	0.0262349
Ltk	red	-0.7977193	0.00910101	-0.3597309	0.28296486
Tyro3	red	-0.9525972	1.33E-13	-0.3897251	0.00351618
Nusap1	yellow	2.45866276	1.53E-18	2.54408953	5.82E-19
4930402H24	red	-0.7871811	5.72E-19	-0.6077806	1.90E-11
Atrn	red	-0.6129502	7.96E-05	-0.4299809	0.00805848
Dll4	skyblue	-1.7692534	9.35E-18	-1.335679	2.26E-10
Gfra4	skyblue	-1.3492403	0.0073938	-1.6867486	0.00151227
Ppp1r14d	orange	1.21558726	0.03296245	1.78744085	0.00239615
Adam33	skyblue	-1.5574695	4.59E-12	-1.1010978	2.09E-06
Siglec1	yellow	3.39517186	1.29E-07	3.09231855	2.57E-06
Rad51	yellow	3.17697201	2.02E-24	2.92791691	2.82E-20
Casc5	yellow	3.59663215	1.32E-17	3.82898707	4.27E-18
Spef1	skyblue	-1.5951839	4.29E-14	-1.2236978	1.58E-08
Knstrn	yellow	2.54370282	2.44E-20	2.67424007	2.43E-21
Ivd	red	-1.1001146	1.04E-05	-0.8740909	0.000712
Smox	orange	0.99775129	0.00047947	1.09976862	0.00015847
Adra1d	skyblue	-1.4682776	8.15E-05	-1.1450739	0.00275612
Rassf2	orange	1.37599776	6.01E-11	1.18090303	4.04E-08
Slc23a2	red	-0.847978	1.43E-09	-0.569351	9.02E-05
Pcna	orange	1.30863308	2.11E-16	1.04954283	1.12E-10
Fsip1	red	-1.3285758	0.00023766	-1.0607468	0.00420746
Rasgrp1	orange	1.31127663	1.20E-08	1.8438599	1.18E-15
Fermt1	red	-0.776034	0.00362322	-0.4270995	0.13574805
Slc27a2	skyblue	-2.1288147	1.81E-07	-1.1440389	0.00767897
Hdc	orange	0.69822131	2.55E-07	0.78603522	9.92E-09
Sppl2a	orange	0.69639935	4.73E-11	0.67208117	3.85E-10
Dusp2	yellow	4.11609364	6.39E-42	3.93549964	2.79E-37
Mall	red	-0.9685441	2.17E-06	-0.627838	0.00338924

Bub1	yellow	2.51562672	1.59E-21	2.81903266	2.03E-23
Acoxl	skyblue	-2.3406099	9.13E-08	-1.8100635	6.39E-05
Bcl2l11	orange	0.68286785	3.04E-08	0.36927338	0.00448162
Fbln7	skyblue	-1.8793955	0.00034954	-1.617248	0.00472462
Ttl	red	-0.973383	6.12E-13	-0.7747619	2.16E-08
Slc20a1	orange	1.04609963	1.38E-09	0.90803526	2.71E-07
Il1b	yellow	3.16729043	1.00E-24	2.6192458	7.36E-19
Il1a	orange	0.17705233	0.62172165	1.50227289	1.86E-06
Nop56	orange	0.81569445	2.36E-07	0.7073838	1.29E-05
Cpxm1	orange	0.3501494	0.2288674	0.74842046	0.00818788
Vps16	orange	0.61228716	6.06E-15	0.47177446	4.50E-09
Rrbp1	orange	0.9816198	5.02E-06	0.92335498	2.80E-05
Snx5	orange	1.45513579	3.19E-12	1.10622641	2.63E-07
Mgme1	red	-0.3994836	0.02343065	-0.7396396	2.96E-05
Rbbp9	red	-0.8102744	9.49E-07	-0.7389861	1.31E-05
Sec23b	orange	0.61781659	1.61E-08	0.58507226	1.54E-07
Xrn2	orange	0.8000451	5.06E-15	0.43876868	4.34E-05
Cst8	skyblue	-2.6586	4.96E-07	-2.473142	2.24E-06
9230104L09F	skyblue	-1.4430732	0.00018024	-1.2499471	0.00166922
Acss1	red	-1.3138232	4.65E-08	-0.9012118	0.00031376
Gins1	yellow	2.5476872	5.89E-29	1.95518216	2.35E-17
Sdcbp2	orange	1.46593705	2.64E-11	1.29864897	5.21E-09
Tpx2	yellow	3.15184228	2.73E-37	3.49727021	1.48E-43
Kif3b	orange	0.58131324	5.31E-06	0.53661997	4.18E-05
Bpifa1	skyblue	-4.1591807	4.30E-30	-3.1776704	8.31E-18
Bpifb1	skyblue	-2.8953319	3.14E-08	-1.2974441	0.02009403
E2f1	orange	0.77837563	0.00078942	0.76120995	0.00163274
Aurka	yellow	2.6328452	1.25E-39	2.62200949	3.10E-37
Pkia	red	-1.3294802	2.72E-11	-1.030893	5.52E-07
Stmn2	skyblue	-2.5524991	3.12E-20	-2.135738	2.36E-14
Rtfdc1	orange	0.64267033	1.39E-09	0.44132338	6.26E-05
Tpd52	orange	0.81594895	0.00159061	0.78258836	0.00321913
Pck1	skyblue	-2.8970678	0.00049938	-1.9568094	0.0252842
Zbp1	black	5.68035632	3.19E-121	5.67629748	5.06E-121
Edn3	skyblue	-1.7276328	0.00011811	-1.4666673	0.0015614
Ptpn1	orange	1.25333908	1.20E-12	1.07706124	2.09E-09
Nfatc2	orange	1.43671045	4.64E-13	1.11441521	5.61E-08
Atp9a	red	-0.7916293	9.87E-10	-0.5010475	0.00020551
Car13	orange	1.0691209	0.00010032	0.59479351	0.04214749
Car3	skyblue	-2.7019647	0.00011085	-1.8095731	0.01360025
Psma7	orange	1.14989412	2.90E-13	0.87924134	5.64E-08
Col9a3	orange	1.1255738	0.00045687	0.60047572	0.08115039
Nkain4	skyblue	-1.5145302	5.94E-08	-1.4930934	1.73E-07

Srms	red	-1.1759262	6.52E-05	-1.1848529	6.06E-05
Helz2	orange	1.49789223	2.25E-22	1.50001517	2.34E-22
Zbtb46	red	-0.6667721	2.43E-05	-0.4637033	0.00480059
Pcmtd2	red	-0.973674	8.00E-05	-0.8847813	0.00050277
Ahcy	orange	1.19982996	7.66E-14	0.9611218	4.75E-09
Map1lc3a	red	-0.9904057	1.66E-08	-0.743444	4.20E-05
Acss2	red	-1.3286996	1.48E-06	-1.0260844	0.00032605
Procr	orange	1.30831168	2.06E-06	1.27611281	5.63E-06
Mmp24	yellow	1.64363118	0.0785576	3.15957942	0.00177083
Eif6	orange	1.16344369	1.65E-08	1.02554469	1.19E-06
Dsn1	orange	1.5747656	1.55E-14	1.136092	6.39E-08
Sla2	yellow	2.7744775	1.22E-15	3.41531387	1.72E-20
Samhd1	yellow	2.41887731	1.16E-101	2.24425411	1.83E-87
Rbl1	orange	1.54280919	3.70E-15	1.41347606	1.14E-12
Ctnnbl1	orange	0.61752371	6.13E-05	0.61657358	9.37E-05
Fam83d	orange	1.52639173	1.78E-06	0.67456824	0.04904642
Wisp2	orange	0.77033925	0.00686865	0.91339784	0.00159781
Ocstamp	black	6.51065088	4.20E-13	5.9820777	1.12E-11
Ccdc39	red	-1.1468388	8.11E-08	-0.7472449	0.00062175
Mecom	red	-0.8754674	4.35E-15	-0.6160988	8.74E-08
Tnik	orange	0.64961576	0.00187566	0.7189737	0.00073947
Ect2	yellow	2.15102236	1.08E-16	2.50418929	8.08E-21
Lrrc34	skyblue	-2.4410811	2.66E-07	-1.394651	0.00311598
Mccc1	red	-0.8974706	4.38E-08	-0.698972	3.64E-05
Exosc9	orange	0.76179694	3.40E-08	0.53393401	0.00024168
Ccna2	yellow	3.06108674	6.54E-26	3.20110437	1.66E-27
Trpc3	skyblue	-1.8348851	2.05E-06	-0.7628089	0.06321614
Il21	black	7.28566282	2.24E-08	7.09119416	9.31E-08
Spata5	orange	0.7724285	0.00010154	0.66493702	0.0012962
Slc7a11	orange	1.68426267	1.66E-07	2.3878042	2.82E-13
Postn	red	-1.0249378	3.02E-09	-0.6304465	0.00048068
Exosc8	orange	1.30042096	4.03E-16	0.92770671	1.94E-08
P2ry1	skyblue	-1.2423024	8.04E-06	-1.656505	1.40E-08
Il12a	skyblue	-1.6167191	2.82E-05	-1.3624702	0.00120212
Ift80	red	-0.7935146	0.00013857	-0.549157	0.01155879
Ppm1l	red	-1.0162977	1.21E-05	-0.6059164	0.01318132
Nmd3	orange	0.97172406	1.02E-10	0.72659479	2.99E-06
Bche	skyblue	-1.3187571	0.00116887	-1.2267185	0.00338851
Ccna1	orange	1.44021285	0.00577891	2.26217017	2.87E-05
Smad9	skyblue	-2.4660886	2.80E-09	-1.6591941	9.05E-05
Dclk1	orange	1.06214467	0.00454728	0.95728863	0.01369654
Nbea	red	-0.857202	2.50E-11	-0.7729862	2.49E-09
Wwtr1	red	-0.5937282	1.39E-05	-0.3422994	0.01769469

Pfn2	red	-0.8831288	1.40E-05	-0.5017857	0.01985224
4930579G24	orange	1.40636051	3.24E-05	0.66907467	0.07073885
Mme	skyblue	-1.5448333	4.66E-05	-1.2949526	0.00094887
Ptx3	black	3.67995015	1.72E-15	5.13342934	3.46E-26
Shox2	skyblue	-1.6582856	0.0061025	-1.7513961	0.00451551
Pdcd10	orange	0.71163018	4.55E-07	0.47589887	0.00127407
Ptpn22	yellow	3.83089522	1.59E-44	3.41980799	2.41E-35
Dclre1b	orange	0.74256668	8.31E-06	0.55979314	0.00140218
Tspan2	red	-1.3091754	5.09E-05	-0.9891741	0.00321222
Vangl1	red	-1.0315198	3.37E-12	-0.6423418	2.74E-05
Casq2	skyblue	-1.7093481	0.01200861	-2.2402145	0.00111124
Cd2	yellow	2.71677011	1.13E-52	2.75813975	1.02E-52
Ptgfrn	red	-0.820951	4.52E-13	-0.7245642	3.31E-10
Spag17	skyblue	-1.6700772	1.13E-07	-0.6717793	0.04181325
Hmgcs2	red	-1.1061863	0.00136175	-0.6826179	0.06199639
1700013F07I	skyblue	-2.1038509	1.05E-08	-1.1216686	0.00180435
Sypl2	skyblue	-2.124887	5.72E-11	-1.2711926	0.00020071
Slc6a17	skyblue	-2.5421893	1.68E-26	-1.8942711	9.98E-17
Kcnc4	red	-1.3589955	0.00164166	-0.5595206	0.24059728
Dennd2d	orange	0.77402017	1.54E-06	0.95408258	3.95E-09
S100a11	orange	0.83620974	5.14E-05	0.84876012	5.82E-05
Crct1	red	-0.866586	0.00262904	-0.5397202	0.07868237
Lce1g	skyblue	-1.8989518	4.21E-06	-0.9292571	0.03337837
Lce1b	red	-1.2440742	9.04E-05	-0.6036145	0.07881701
Npr1	red	-1.2545866	1.99E-11	-0.947954	8.56E-07
Slc27a3	red	-0.4997874	0.00839633	-0.5801661	0.00275612
Creb3l4	red	-1.0312626	0.00227028	-0.2574848	0.49747867
Tpm3	orange	1.07943824	1.97E-09	0.93614114	3.64E-07
Chrnb2	orange	1.18940523	2.29E-06	0.8718494	0.00067516
Adar	orange	1.59872781	8.52E-35	1.51300148	3.06E-31
Pmvk	orange	0.87674166	0.00110024	0.70899979	0.01097136
Efna1	skyblue	-1.5753139	1.08E-14	-1.3178895	2.16E-10
Fam198b	red	-1.1199154	4.11E-09	-0.7775674	8.52E-05
Tmem144	red	-1.1301624	0.00045911	-0.5002668	0.14929956
Sass6	orange	1.36633326	2.76E-06	0.96529068	0.0014042
Lrrc39	skyblue	-1.3404923	5.42E-05	-1.2034545	0.00068816
Vcam1	orange	1.17434116	9.72E-07	1.19363785	1.03E-06
Larp7	orange	0.69479139	1.67E-06	0.58262869	0.00012402
Cyp2u1	red	-0.9473364	0.00526273	-0.8493946	0.01665082
Hadh	red	-0.9540894	8.87E-08	-0.8353024	5.09E-06
Ccdc109b	orange	1.36889959	1.37E-10	1.04594628	2.55E-06
Tlr2	yellow	2.29759126	7.06E-12	1.84068461	8.34E-08
Sfrp2	skyblue	-1.6382995	5.50E-09	-2.0010005	2.18E-12

Fga	black	5.03812752	1.40E-06	3.39298003	0.00023535
Lrat	skyblue	-2.8025844	7.28E-11	-2.4761674	1.67E-08
Gucy1b3	red	-1.3149027	5.84E-08	-1.0034608	6.17E-05
Gar1	orange	0.8040405	0.00072375	0.83231191	0.00066261
Egf	red	-0.9821002	0.0014973	-0.4366296	0.20410636
Pitx2	red	-1.4258249	0.00022072	-0.5262737	0.23207412
Enpep	skyblue	-1.5410399	1.96E-07	-1.0882158	0.0004034
Alpk1	orange	0.74218814	0.0005392	0.74106966	0.00074399
Dkk2	skyblue	-2.7957819	4.75E-10	-1.688101	0.00011224
Papss1	red	-0.5843046	1.14E-08	-0.3722605	0.00052127
Kcnq5	red	-0.6607323	0.00841973	-0.8558679	0.000779
Ptgfr	skyblue	-2.9050548	2.22E-05	-2.4495745	0.0005258
Ifi44	yellow	3.66771846	1.10E-50	3.90565927	1.56E-56
Adam15	orange	0.82278861	1.41E-07	0.67501242	2.77E-05
Cks1b	yellow	2.3715481	3.13E-25	1.99636558	1.89E-17
Thbs3	skyblue	-2.4298186	3.97E-17	-1.5979421	8.38E-08
Gba	orange	0.80554495	7.08E-06	0.68388534	0.00022199
Sema4a	orange	1.56428038	1.83E-17	1.38014621	1.35E-13
Pmf1	orange	1.46875708	7.60E-15	1.16988109	1.66E-09
Iqgap3	yellow	2.04844712	1.94E-08	2.68172137	5.24E-13
Gpatch4	orange	0.75762387	2.16E-05	0.78784496	1.56E-05
Sh2d2a	yellow	4.31842731	1.98E-76	4.17482948	9.44E-71
Dclk2	orange	1.01942581	5.05E-06	1.04387003	3.97E-06
Sh3d19	red	-1.2260686	1.21E-10	-0.8043471	4.75E-05
Fmo5	skyblue	-2.0196551	4.57E-06	-1.6904109	0.00019783
Acp6	red	-0.6011019	7.53E-05	-0.5085617	0.00119731
Rnf115	orange	0.80192463	8.43E-07	0.70564866	2.56E-05
Polr3c	orange	0.80820647	2.53E-07	0.67266633	3.14E-05
Bnipl	red	-1.0405034	1.24E-05	-0.7921586	0.00140521
Myoz2	skyblue	-1.4094051	0.00054101	-2.0215734	8.64E-07
Abcd3	red	-0.7974374	7.80E-05	-0.5347629	0.01145708
F3	orange	2.0047208	9.13E-11	1.77274073	1.90E-08
Tmem56	red	-0.7288339	0.00029732	-0.2335345	0.29264392
Riiad1	skyblue	-1.5884464	3.61E-11	-1.3137635	7.61E-08
Them5	red	-1.0928169	0.00584067	-0.9148296	0.02681502
Mttp	red	-0.9789825	9.67E-05	-0.6185278	0.01641561
Dapp1	orange	1.17987811	2.63E-14	1.14734316	3.38E-13
Wls	red	-0.7603371	7.41E-10	-0.5859968	3.99E-06
Depdc1a	yellow	3.7791719	7.07E-17	4.17934855	4.80E-15
Uox	skyblue	-2.4942856	0.00547869	-1.5105738	0.07793209
Bcl10	orange	0.77588837	5.05E-08	0.51975126	0.00046545
Cyr61	orange	1.03561192	1.42E-05	1.21798228	4.38E-07
Cryz	red	-0.6964635	0.00045687	-0.522753	0.01253056

Asph	red	-0.7402269	6.80E-07	-0.5432416	0.00043729
Ccne2	orange	0.74413902	0.00527658	0.37645991	0.20991613
Gem	orange	0.52485163	2.61E-07	0.63057062	7.46E-10
Cdh17	black	5.18140655	1.77E-08	3.32659774	9.97E-08
Decr1	red	-0.7214225	7.33E-06	-0.7415067	6.59E-06
Rmdn1	red	-1.0532525	4.16E-05	-0.9743635	0.00022389
Rps20	orange	0.74286277	2.28E-15	0.62997035	4.17E-11
Faxc	red	-1.3716083	0.00058822	-0.6899032	0.08554221
Sdcbp	orange	0.87863491	7.11E-06	0.68405102	0.00074247
Tstd3	orange	0.81342767	0.00151116	0.20351126	0.49759145
Clca3a2	yellow	2.42914617	0.00849033	2.67652362	0.00085408
Lmo4	orange	0.87739404	3.32E-07	0.6600042	0.00021314
Gbp3	yellow	3.44887892	8.64E-89	3.35806653	2.21E-84
Gbp2	yellow	4.3244958	6.93E-193	4.13672949	7.06E-177
Rragd	red	-1.3598405	4.10E-09	-1.1006594	4.18E-06
Casp8ap2	orange	0.54882055	3.39E-06	0.60421025	4.81E-07
Slc35a1	red	-0.6544555	2.50E-11	-0.3513037	0.00072977
1700003M02	skyblue	-2.2809868	2.40E-12	-1.4458632	1.41E-05
Smc2	orange	2.02062335	8.66E-20	1.6011434	1.81E-12
Polr1e	orange	0.82437018	3.89E-07	0.72468469	1.41E-05
Exosc3	orange	0.82906301	1.79E-07	0.65583132	6.82E-05
Stra6l	black	4.3593537	9.22E-13	4.60986195	4.39E-13
Tmod1	red	-1.0633783	0.00742779	-0.7708506	0.06512161
Anp32b	orange	0.87854113	7.92E-08	0.69202897	4.32E-05
Nans	orange	0.97047627	1.11E-05	0.66688274	0.00382305
Coro2a	orange	1.96330114	1.30E-18	1.4312962	3.61E-10
Col15a1	skyblue	-1.5736867	4.52E-10	-0.8035033	0.00254451
Invs	red	-0.9455134	8.48E-09	-0.8515935	2.92E-07
Tex10	orange	0.74258849	9.55E-07	0.51919452	0.00111028
Tmeff1	skyblue	-1.2573205	8.64E-13	-1.278248	5.29E-13
Murc	red	-1.1827427	1.83E-07	-0.6313686	0.00961384
Fmn2	skyblue	-1.7235736	0.00401765	-1.9867586	0.00166547
Zfp618	orange	1.20282901	1.07E-05	1.04599954	0.00014405
Tnfsf8	yellow	3.21455767	1.74E-16	2.18605384	6.12E-09
Tnc	yellow	1.96368746	0.000762	2.45457729	3.21E-05
Txn1	orange	1.08905788	3.20E-19	0.99250353	6.09E-16
Pappa	orange	0.90706332	0.00404814	0.81380906	0.01012041
Astn2	red	-0.9119418	1.48E-06	-0.7237769	0.00021615
Ptgr1	red	-0.9883298	6.54E-07	-0.6744516	0.0011287
Ugcg	orange	0.85977126	1.41E-12	0.63329395	4.57E-07
Ptbp3	orange	0.78711177	1.37E-08	0.60075751	2.78E-05
Snx30	orange	0.50441863	0.00395431	0.60625053	0.00066322
Wdr31	red	-1.1061586	1.49E-05	-0.4029911	0.14279171

2310002L09F	skyblue	-2.3941282	1.03E-05	-1.3324127	0.02102977
Ptprd	red	-0.7203967	3.63E-05	-0.7269764	5.03E-05
Mpdz	red	-0.8634131	6.16E-10	-0.7478801	1.72E-07
Zdhhc21	orange	0.65413532	2.00E-05	0.77424025	7.45E-07
B4galt1	orange	0.98979995	5.49E-08	0.66302046	0.00047456
Fktn	red	-0.8285963	3.79E-07	-0.5575491	0.00106087
Aqp7	skyblue	-3.1470368	2.39E-08	-2.1041535	0.00032626
Epb41l4b	skyblue	-1.565367	4.84E-08	-1.4036665	1.69E-06
Kif24	orange	1.00310855	0.00048668	0.93363182	0.00096254
1110017D15	skyblue	-1.8660387	2.12E-17	-1.3717415	5.76E-10
Cntfr	red	-1.0914406	0.00018621	-1.0626474	0.00036967
Unc13b	red	-0.7018474	5.33E-06	-0.4719762	0.00341132
Cd72	yellow	4.33624166	5.04E-16	4.23870198	3.98E-15
Sit1	yellow	2.3385861	0.00015948	3.21426488	6.18E-07
Tpm2	red	-1.1048329	1.79E-05	-0.5580457	0.04290622
Creb3	orange	0.62813378	3.99E-05	0.51269267	0.00120574
Npr2	red	-1.2639384	2.21E-06	-0.9069885	0.00108436
Reck	skyblue	-1.5831401	3.28E-07	-1.1844066	0.00022396
Clta	orange	0.66549474	2.04E-09	0.45718269	7.54E-05
Glipr2	yellow	2.88290405	4.98E-35	2.73690078	1.71E-31
Sh3gl2	skyblue	-2.2573875	2.42E-11	-1.3301681	5.21E-05
Plin2	orange	1.87321764	1.75E-08	1.79175023	1.20E-07
Rps6	orange	0.59015277	9.22E-08	0.47108242	3.59E-05
Ppap2b	red	-0.9077488	3.78E-09	-0.6257288	9.05E-05
Prkaa2	red	-0.997509	0.00024207	-0.7635771	0.0069683
Sgip1	red	-0.9833913	1.91E-05	-0.4929494	0.04435374
Ak4	red	-0.875964	0.00659256	-0.6955805	0.0397134
Dnajc6	red	-1.3687174	0.00231753	-0.287851	0.58249852
Cachd1	red	-0.6691933	1.63E-06	-0.6576436	3.81E-06
2610528J11F	red	-0.8186639	0.00032899	-0.7186145	0.00222948
Dph2	orange	0.91013809	3.56E-05	0.71937922	0.00157797
Ttc39a	skyblue	-1.5498641	1.85E-15	-0.9496385	1.68E-06
Usp1	orange	0.58531262	8.41E-05	0.57609237	0.00016678
Nfia	red	-0.8054853	9.72E-08	-0.9560155	3.25E-10
Fggy	red	-0.7030441	0.00033608	-0.921799	5.50E-06
Plaa	orange	0.85204075	6.07E-10	0.68790426	1.18E-06
Laptm5	yellow	2.28683185	1.12E-22	2.07436242	1.12E-18
Orc1	orange	1.54741331	4.08E-05	1.54706358	6.07E-05
Tnfrsf1b	yellow	2.33733181	4.40E-19	2.10604017	1.61E-15
Podn	red	-0.5083113	0.07508426	-0.758515	0.0076681
Echdc2	red	-0.9762285	4.72E-08	-0.8358854	5.37E-06
Tnfrsf8	black	3.91413192	9.91E-22	5.10014354	2.42E-26
Cpt2	red	-0.6144166	0.00043169	-0.5701169	0.00148903

Magoh	orange	0.75919518	5.44E-07	0.58158005	0.00023114
Lrp8	yellow	3.01135896	3.98E-15	3.13234363	5.35E-16
Tmem59	red	-0.6590589	2.97E-07	-0.6407923	1.05E-06
Cyb5rl	red	-0.7604114	2.54E-05	-0.9069889	7.42E-07
Col9a2	skyblue	-2.2308404	0.00218165	-0.9854805	0.19614548
Dyrk2	red	-0.5978111	6.62E-09	-0.4569824	1.83E-05
Kcnq4	red	-0.9981201	0.00019085	-0.9841892	0.00033633
Ctps	orange	1.56337813	1.17E-16	1.5775065	8.22E-17
Hivep3	orange	0.99984689	0.00020884	0.90937594	0.00106138
Ccdc30	skyblue	-1.5809817	2.84E-11	-0.78703	0.00129566
Ccdc23	orange	0.82301005	3.67E-06	0.55182252	0.00315263
Slc2a1	orange	1.37147957	1.30E-10	1.14792252	1.50E-07
Macf1	red	-0.697256	7.81E-05	-0.5155279	0.00503309
Ppie	orange	0.64228227	0.00066407	0.33089066	0.10587659
Mycl	red	-0.9788098	0.00132218	-0.7433602	0.01911201
Cap1	orange	0.89083473	5.63E-07	0.76034235	3.31E-05
Lypla2	orange	0.6687576	4.32E-06	0.46206695	0.00237775
Gale	orange	1.04770629	1.27E-08	1.07515913	8.92E-09
Kif2c	yellow	3.44056655	6.79E-30	4.56416104	8.40E-41
Plk3	orange	2.09036667	2.67E-15	1.72942048	1.25E-10
Mmachc	red	-0.7581528	5.71E-05	-0.6497159	0.00093804
Nasp	orange	1.11443177	5.41E-14	1.0261063	1.12E-11
Pik3r3	red	-0.5895186	0.0008203	-0.654709	0.00027955
Tspan1	red	-1.2988845	7.54E-11	-0.6321539	0.00228536
Lurap1	red	-1.177116	3.86E-05	-0.8196037	0.0057546
Rad54l	yellow	2.78785465	4.94E-15	2.38899791	3.58E-11
Cyp4b1	red	-0.9515869	0.00187032	-0.7336886	0.02156498
Pdzk1ip1	red	-1.1317322	3.80E-05	-0.8435256	0.00317351
Tal1	red	-0.8366554	3.80E-07	-0.7642468	5.77E-06
Stil	yellow	3.42185406	5.09E-19	3.2465678	2.97E-17
Cmpk1	orange	0.74570076	5.17E-07	0.70734209	3.38E-06
Ebna1bp2	orange	0.65751077	0.000228	0.46918363	0.01199588
Cfap57	skyblue	-2.2690217	5.11E-11	-1.4230477	5.76E-05
Aldh4a1	red	-1.0825155	1.39E-06	-0.8797919	0.00014383
Mrto4	orange	1.05713411	5.64E-06	0.92038012	0.00012689
Akr7a5	red	-1.0437141	1.01E-12	-0.6121387	6.08E-05
Pqlc2	orange	0.82088649	1.30E-05	0.5546356	0.0047989
Capzb	orange	0.65250445	0.00034245	0.50380735	0.0078765
Pla2g2f	skyblue	-1.4008146	0.00153901	-1.2338065	0.00966508
Pla2g2c	skyblue	-1.2573104	0.15278508	-2.6271015	0.00435309
Pink1	red	-0.9590349	0.00017045	-0.9069912	0.00054149
Hspg2	red	-0.7109395	0.00011678	-0.1708877	0.42052201
Alpl	orange	1.52431296	1.20E-05	2.06588845	3.03E-09

Fabp3	skyblue	-1.6437394	0.0001979	-1.8402524	4.37E-05
Tinagl1	orange	1.55785551	6.53E-17	1.50711395	9.49E-16
Hcrtr1	yellow	0.85870967	0.40178155	2.83127537	0.00260405
Sema3c	red	-1.3821134	5.67E-19	-1.0217168	1.31E-10
Hpca	yellow	2.81297955	1.19E-05	3.68249088	8.26E-07
Azin2	red	-0.6222107	0.00094266	-0.2462328	0.23757871
Ak2	orange	1.21502075	2.33E-09	1.08005084	2.09E-07
Rnf19b	yellow	2.89270122	4.34E-34	2.79615396	8.46E-32
Phc2	orange	0.79325188	2.68E-14	0.67219434	2.34E-10
Eif3i	orange	0.60016975	3.22E-08	0.51157097	4.58E-06
Stpg1	skyblue	-2.3101718	2.13E-05	-1.5774544	0.00338924
Nipal3	red	-0.7870506	9.52E-05	-0.6763688	0.0011434
Yars	orange	0.79687447	1.49E-06	0.70322036	3.58E-05
Trim63	red	-0.7000708	0.01940251	-1.2698072	2.65E-05
Slc30a2	orange	1.0474029	0.02171513	1.26055888	0.00728937
Psmb2	orange	1.2893377	1.99E-16	0.96788906	1.79E-09
Extl1	red	-1.1756756	8.47E-08	-0.6503672	0.005004
Cnksr1	orange	0.32887796	0.0644357	0.72094012	3.24E-05
Sh3bgrl3	orange	1.60725748	5.52E-24	1.05816553	1.08E-10
Tekt2	red	-1.3936668	2.11E-05	-0.9377653	0.00600034
Gpn2	orange	0.60153095	2.21E-05	0.42482765	0.0042765
Gpatch3	orange	0.84863835	0.00027046	0.74390875	0.00228536
Slc9a1	orange	0.61029467	7.88E-05	0.55029261	0.00054428
Csf3r	yellow	2.84168277	5.36E-18	2.59500217	3.84E-15
Map3k6	orange	0.94805293	1.40E-07	0.34861173	0.0749479
Cdca8	yellow	2.95040101	1.86E-42	2.9920538	2.04E-40
Fgr	yellow	2.96388927	8.80E-20	2.81133661	8.33E-18
Sema3a	red	-0.9428426	1.81E-09	-0.6324955	0.00010306
Rpa2	orange	1.18994733	2.98E-16	0.87449966	6.44E-09
Smpdl3b	orange	1.76645725	3.86E-08	1.58878339	1.31E-06
Yrdc	orange	0.92028481	7.16E-08	0.65591694	0.00022122
Sesn2	orange	0.71235206	1.21E-06	0.39752071	0.00976916
Rcc1	orange	1.28043066	5.18E-11	1.42523272	4.95E-13
Sf3a3	orange	0.6049432	1.56E-06	0.59192205	4.45E-06
Ptpru	skyblue	-1.530632	2.06E-07	-1.287027	2.13E-05
Arhgef19	skyblue	-1.6843322	4.49E-16	-1.1617231	4.58E-08
Necap2	orange	0.66322716	5.34E-09	0.57014497	1.02E-06
Cdk14	red	-0.5423444	1.32E-05	-0.6370524	4.21E-07
Padi2	yellow	2.80107022	3.19E-29	2.50163104	4.21E-23
Kcnab2	yellow	2.43041789	3.34E-38	2.28026459	1.29E-33
Psmc2	orange	0.74614982	9.49E-08	0.55049361	0.00014697
Hes2	skyblue	-3.2307578	0.00014524	-2.8490861	0.0011567
Espn	red	-1.0023594	4.35E-06	-0.8992525	6.70E-05

Prkag2	red	-0.6349621	0.00032404	-0.83292	3.10E-06
Smarcd3	red	-0.6794246	0.00019365	-0.3939238	0.04349012
Tas1r1	red	-1.1035712	0.0098522	-0.6083892	0.16535684
Per3	skyblue	-2.0424355	3.05E-07	-1.582278	0.00012362
Pgd	orange	0.61452417	0.00146383	0.52443336	0.00870464
Tnfrsf9	black	4.48940356	8.08E-46	5.5165712	3.17E-54
Errfi1	orange	0.75700922	0.00017572	0.40942288	0.05763078
Abcb1b	orange	1.32644361	3.29E-08	1.08982007	1.01E-05
Casz1	red	-0.6668761	2.88E-05	-0.3553303	0.03628659
Slc25a33	red	-0.98276	7.96E-06	-1.0776123	1.66E-06
Klhl7	red	-0.7630533	9.19E-12	-0.5322138	3.78E-06
Angptl7	red	-0.2724503	0.51839664	-1.3541574	0.00058425
Rbp7	skyblue	-3.0287623	0.00035937	-4.0113711	1.42E-06
Mad2l2	orange	1.29929645	4.40E-15	1.07444804	2.09E-10
Draxin	orange	2.15110427	0.0001671	0.16839377	0.80917766
Mthfr	orange	0.81642295	4.64E-05	0.73181379	0.00038776
Dnajc2	orange	1.16078665	1.27E-08	0.93939544	8.62E-06
Miip	orange	0.61140992	2.66E-07	0.38156535	0.0020675
Trp73	skyblue	-1.976975	2.69E-14	-1.2859906	9.41E-07
Tprgl	red	-0.7429689	1.15E-10	-0.5593714	2.50E-06
Pex10	red	-1.0204617	7.80E-11	-0.8131233	5.23E-07
Morn1	red	-1.1828993	7.12E-07	-0.9197988	0.00016175
Prkcz	red	-1.5573695	7.88E-09	-1.0152133	0.00029905
Plch2	red	-1.3176183	3.34E-06	-0.728044	0.01471564
Fam213b	red	-0.7520422	0.00014081	-0.4892787	0.01959683
Mib2	red	-1.1248001	1.35E-09	-1.0205302	6.88E-08
Mmp23	red	-0.6310796	0.00186238	-0.3810715	0.07587952
Mxra8	red	-0.965194	1.65E-07	-0.7195997	0.00016503
Ttll10	red	-1.1295416	0.00400829	-0.216012	0.63972609
Tnfrsf4	yellow	3.29539712	1.27E-70	2.53925996	1.44E-47
Bst1	orange	1.9803397	2.85E-09	1.70242118	6.17E-07
Prom1	skyblue	-1.8516593	2.98E-12	-1.1056415	5.36E-05
Adgra2	red	-0.7094695	0.00836011	-0.4390386	0.12616601
D5Ertd615e	yellow	2.24841904	0.05926686	4.02845689	0.00957667
Ablim2	red	-1.1816258	3.19E-12	-0.8433096	1.68E-06
Htra3	red	-0.995062	6.39E-06	-0.9559393	2.30E-05
Htt	orange	0.9332273	1.19E-10	0.91363269	4.80E-10
Pcdh7	red	-0.7865189	0.00063809	-0.8147362	0.00055362
Ppp2r2c	skyblue	-1.0943295	0.01804987	-2.4160463	8.50E-08
Crmp1	yellow	2.83458985	4.47E-20	2.02123207	8.53E-11
Evc	red	-1.3653645	4.95E-13	-1.0781637	2.38E-08
Stk32b	skyblue	0.47083555	0.69507448	-4.2756063	0.0041133
Stx18	orange	0.59660079	6.24E-05	0.34855329	0.02726685

Rnf32	red	-0.9530935	0.00665815	-1.1884831	0.0009629
Fosl2	orange	0.89925842	6.59E-10	0.80235457	6.50E-08
Rbks	orange	0.98671381	0.00012336	0.57924654	0.03489359
Ppm1g	orange	0.64235181	2.87E-08	0.52465237	1.08E-05
Ociad2	skyblue	-1.6855322	5.84E-13	-1.0369463	2.52E-05
Spata18	skyblue	-2.3885086	1.42E-14	-0.9631432	0.00229323
Yipf7	skyblue	-1.2477174	0.06838568	-2.2177452	0.00155275
Emilin1	orange	0.77086872	0.00890243	0.8470685	0.00477786
Mapre3	red	-0.8675276	3.75E-17	-0.5804909	5.04E-08
Ppargc1a	red	-1.1337185	0.00057026	-0.8562226	0.01278962
Dpysl5	red	-0.867685	0.04295395	-1.1550279	0.00721704
Tbc1d1	orange	0.61741025	0.0005138	0.40826619	0.02916142
Slc35f6	orange	0.74174904	0.00013925	0.66162211	0.00098223
Cenpa	yellow	2.21450938	2.15E-37	1.98964082	6.44E-30
1700001C02	skyblue	-2.0440568	1.85E-09	-1.5124563	1.12E-05
Pi4k2b	red	-0.6002794	9.95E-05	-0.4676166	0.00354299
Slc34a2	red	-1.2722221	0.00035411	-0.7185849	0.05869862
Sel1l3	red	-1.2361348	2.56E-07	-0.1786965	0.53164944
Tbc1d14	orange	0.68461059	0.00043209	0.58953248	0.00335275
Cckar	skyblue	-2.1228837	4.02E-16	-1.1487705	2.38E-05
Klb	skyblue	-1.6474993	0.0024469	-1.5350131	0.01169121
Rhoh	yellow	2.11035044	4.70E-12	2.86351387	1.04E-20
Tec	orange	1.24760008	9.97E-09	0.96220423	1.98E-05
Lnx1	red	-0.985466	0.00015617	-0.5582932	0.04030695
Chic2	orange	0.65325057	5.53E-07	0.55072375	4.33E-05
Thegl	skyblue	-1.8821256	0.00760501	-1.1069341	0.11472406
Cenpc1	orange	0.66447547	0.00028247	0.3583239	0.06834015
Stap1	orange	0.98994486	0.00022501	1.38128875	7.59E-07
Pigg	red	-0.7097775	1.70E-05	-0.47018	0.00633347
Sult1d1	skyblue	-2.2846078	7.84E-08	-1.909575	1.09E-05
Gfi1	yellow	2.88649373	7.74E-11	4.10148754	2.39E-18
Glmn	orange	1.29400107	7.04E-05	0.62750058	0.07154206
Cdc7	orange	1.42123482	7.26E-09	1.07763151	2.04E-05
Tgfbr3	red	-0.7147809	0.0041586	-0.6753288	0.00848929
Rufy3	orange	0.70225576	0.00177386	0.77340393	0.00076758
Gbp9	yellow	2.23113011	4.60E-47	2.31488438	1.37E-50
Abcg3	red	-1.122505	0.00302188	-0.343773	0.42326176
Spp1	orange	2.09524979	5.61E-08	1.52135431	0.00014052
Hsd17b11	red	-0.5312612	2.07E-05	-0.6184136	1.02E-06
Klhl8	red	-1.0585135	2.17E-06	-0.73849	0.00137683
Agpat9	red	-0.7358525	4.63E-05	-0.2097197	0.30490797
Plac8	yellow	3.75298729	3.65E-44	3.80261857	3.64E-45
Bmp3	skyblue	-1.7318463	1.76E-09	-1.1299164	0.00016107

Antxr2	red	-1.129878	3.83E-13	-0.9645241	1.16E-09
Srrd	orange	0.40870236	0.06716095	0.66849857	0.00315361
Crybb3	skyblue	-1.7331174	9.40E-08	-1.1499251	0.00052507
Rfc5	orange	1.6286037	1.90E-19	1.43268537	6.57E-15
Dck	orange	1.81224928	1.51E-11	1.96087024	4.66E-13
Cxcl5	yellow	4.39491616	5.43E-15	4.19329305	1.34E-19
Ppbp	red	0.04260219	0.88747658	-1.019683	5.99E-05
Ereg	orange	1.73982006	0.00405517	1.51369188	0.01530564
Areg	yellow	2.12263936	1.45E-05	2.58552703	1.80E-07
Cxcl3	black	5.40938834	3.18E-06	7.76851641	1.65E-07
Cxcl1	yellow	2.9206574	1.92E-21	3.81787166	1.25E-33
Tctn2	red	-0.711747	3.63E-05	-0.3900668	0.0320844
Rilpl1	red	-0.6717476	9.65E-05	-0.5781568	0.00116141
Rilpl2	orange	1.11497884	2.19E-12	0.99611206	6.60E-10
Cdkl2	red	-1.069707	1.11E-05	-0.8699231	0.00055059
G3bp2	orange	0.74742495	3.64E-06	0.62299445	0.00018852
Naaa	orange	0.85495411	3.96E-07	0.75104165	1.51E-05
Kntc1	yellow	2.87639995	5.84E-21	2.8323872	4.24E-19
Sdad1	orange	1.15275338	5.56E-07	0.98142253	3.44E-05
Cxcl9	black	8.52371505	3.32E-96	7.23535103	3.14E-81
Rimbp2	skyblue	-2.3215186	0.00079437	-0.415504	0.54149282
Ran	orange	1.17163114	3.85E-10	1.07063844	2.00E-08
Gbas	red	-0.702916	7.37E-06	-0.7049017	1.09E-05
Bcl7a	red	-1.398817	1.67E-06	-0.9372523	0.00206908
Wdr66	skyblue	-1.8647158	5.67E-14	-1.0425489	4.95E-05
Psph	orange	0.7320747	0.00061747	0.41963986	0.06774657
Cct6a	orange	0.58893466	0.00023033	0.39240101	0.01972635
Rhof	yellow	2.07326777	9.10E-24	2.05166292	5.10E-23
Aldh2	red	-0.8055961	0.00081065	-0.6507138	0.00917888
Acad10	red	-1.0869877	5.12E-10	-0.9811829	3.14E-08
Arpc3	orange	0.78315496	1.02E-10	0.71674213	6.44E-09
Ift81	red	-1.281067	1.92E-13	-0.9388803	1.51E-07
Rnf34	orange	0.61546419	0.00681594	0.38268535	0.11527882
Morn3	skyblue	-1.4919262	9.66E-08	-1.4627563	3.41E-07
Anxa3	orange	0.66258922	3.32E-06	0.73403426	3.73E-07
Mfsd7a	orange	0.86537037	4.85E-05	0.7996787	0.00029142
Pxmp2	red	-1.110927	0.00107841	-1.0184492	0.00378844
Ep400	orange	0.59066444	5.40E-07	0.45752079	0.00017677
Pus1	orange	0.74253962	0.00234374	0.81140273	0.00114737
Ulk1	red	-0.6045476	0.00215214	-0.3821362	0.06748333
Cit	orange	0.97626971	4.64E-09	0.69816809	5.12E-05
Pla2g1b	skyblue	-1.8277961	0.0005801	-1.2158691	0.02815754
Sirt4	red	-0.7519682	2.60E-05	-0.605933	0.00106959

Ccr9	yellow	-0.4670977	0.73236893	3.46769016	0.00444269
Srsf9	orange	0.67742986	3.92E-06	0.50148278	0.00107332
Tes	orange	1.17709859	2.00E-13	0.91818666	2.32E-08
Tfec	orange	2.07698228	2.24E-09	1.51181539	2.30E-05
Oasl2	yellow	3.46265124	1.03E-93	3.45947281	1.90E-93
Foxp2	skyblue	-1.2202087	2.30E-07	-1.3852805	1.02E-08
Lfng	orange	0.77985918	3.01E-10	0.44758919	0.00057679
Mmab	red	-0.8439513	3.68E-06	-0.4063028	0.0369049
Radil	red	-1.1729967	3.18E-05	-0.5708847	0.05984284
Actb	orange	1.32325685	3.43E-14	1.08025871	1.36E-09
Fscn1	orange	1.54924773	4.93E-11	1.4117147	4.20E-09
Zfp12	red	-0.5980013	0.00051249	-0.5708696	0.0012167
Ung	orange	1.21058673	0.00035704	1.05033686	0.0029951
Rita1	red	-0.9089466	1.43E-06	-0.5724435	0.0039453
Iqcd	skyblue	-2.5752971	4.01E-11	-1.4064813	0.00020958
Oas1b	yellow	2.04718971	1.44E-40	2.14323843	8.09E-45
Rpl6	orange	0.72241059	4.47E-08	0.46513235	0.00074247
Erp29	orange	0.90553361	1.24E-14	0.64620454	1.04E-07
Ccz1	orange	0.73279339	5.35E-10	0.47832584	0.00010506
Arpc1b	orange	1.55833019	5.35E-13	1.30816838	2.96E-09
Cpsf4	orange	0.8785612	1.90E-12	0.69287769	5.94E-08
Wasf3	skyblue	-1.6561524	2.21E-09	-1.3439922	2.27E-06
Pomp	orange	1.07398216	1.21E-08	0.8508463	1.22E-05
Slc46a3	red	-0.6973694	1.99E-06	-0.4883349	0.00125124
Mtus2	skyblue	-1.6356316	0.0007436	-2.0661364	2.58E-05
Hsph1	red	-1.060138	5.92E-07	-1.1117724	2.63E-07
Tex26	skyblue	-1.2250093	0.00488549	-1.3340266	0.00161749
Tfpi2	orange	1.56634253	0.00327734	1.35488396	0.01405996
Tspan12	skyblue	-1.9587273	1.74E-26	-1.6046898	5.50E-18
Auts2	red	-1.0357689	0.00339964	-0.0051596	0.99114126
Limk1	orange	0.72414075	0.00010251	0.36749865	0.06589018
Eln	orange	2.03677434	0.00106152	1.83945551	0.00412022
Lmod2	red	-0.7257163	0.02189539	-0.8613284	0.00742969
Asb15	skyblue	-2.3354091	0.0001983	-2.0412697	0.00154797
Ezh2	orange	1.50208739	6.09E-16	1.42263867	4.00E-14
Aass	skyblue	-2.6937917	7.68E-05	-1.3151655	0.06782833
Ssc4d	orange	1.20005376	0.00054055	0.82854234	0.02049127
Ephb4	red	-1.0416505	1.39E-11	-0.8779478	2.47E-08
Pop7	orange	0.81676631	1.69E-05	0.85391554	1.48E-05
Pcolce	red	-0.6459248	4.30E-05	-0.3097862	0.0685469
Agfg2	orange	0.62171791	8.06E-06	0.49654917	0.0005688
Tsc22d4	orange	0.58376545	5.50E-05	0.5090701	0.00065572
Mcm7	orange	1.34612703	5.64E-26	1.03475387	1.61E-15

Tpk1	red	-0.7390269	4.71E-05	-0.5365647	0.00367343
Asns	orange	0.79314003	7.49E-08	0.88281374	3.24E-09
Pon3	red	-0.7030507	2.03E-05	-0.6191967	0.00027033
Akr1b8	orange	0.77950703	0.00597062	0.84904941	0.00328913
Ccdc136	red	-1.0384477	2.30E-05	-0.7005989	0.00788897
Irf5	yellow	2.57261272	1.84E-14	2.73363534	5.34E-16
Gars	orange	1.05815091	2.00E-09	0.9584644	1.01E-07
Adcyap1r1	skyblue	-1.6408067	8.71E-10	-0.7181945	0.00888122
Nt5c3	orange	1.14854757	1.89E-15	0.9519892	1.35E-10
Fkbp9	red	-0.6910305	3.20E-12	-0.4943849	1.41E-06
Sspo	skyblue	-2.1607375	4.33E-07	-1.5102153	0.0005291
Herc6	yellow	2.77275405	2.75E-92	2.84064924	5.52E-96
Herc3	orange	1.03415776	8.04E-14	0.78267582	3.49E-08
Igf2bp3	orange	1.25364268	3.01E-05	0.9079699	0.00291651
Malsu1	orange	0.77459782	1.88E-05	0.63840443	0.00063934
Gpnmb	orange	1.6532211	3.24E-06	1.44818826	7.31E-05
Dfna5	orange	1.01180581	0.00187347	0.243506	0.52381811
Osbpl3	orange	0.82478465	3.65E-06	0.40462904	0.03363885
Zc3hav1	orange	1.31746292	3.09E-23	1.1627484	3.79E-18
Tmem213	yellow	3.40297014	0.00081614	0.96705523	0.23077053
Nfe2l3	red	-0.9262184	9.53E-05	-0.3868728	0.1193793
Ptn	red	-1.2540837	4.60E-06	-0.8926068	0.00186044
Tcaf2	red	-0.7096781	0.00015204	-0.5360483	0.00633554
Epha1	skyblue	-1.3653616	1.90E-13	-1.1669292	6.10E-10
Fam131b	skyblue	-1.146721	0.01203838	-1.6280178	0.00033516
Clcn1	skyblue	-1.3245402	5.13E-07	-1.2708627	4.73E-06
Gstk1	red	-1.2205254	5.19E-09	-0.8262503	0.00013879
Ccdc184	orange	1.03753329	0.00510717	1.33396329	0.00037182
Mad2l1	orange	1.90913921	3.95E-24	1.82769355	1.30E-21
Clec5a	yellow	3.71587427	7.50E-12	3.24848886	3.87E-09
Hpgds	orange	2.06345845	1.64E-07	1.53623007	0.00017702
Rab19	orange	1.4433583	8.45E-16	1.2982441	1.21E-12
Tbxas1	yellow	2.16929198	1.84E-09	1.7678739	1.91E-06
Pcyox1	red	-0.9346421	8.76E-09	-0.8825596	9.58E-08
Add2	skyblue	-0.9173144	0.32264548	-2.7803121	0.00950464
Nat8	skyblue	-2.1426549	0.00066998	-1.0900625	0.07642089
Pradc1	red	-0.6468328	7.69E-06	-0.5541356	0.00025379
Prickle2	red	-0.6886722	1.25E-06	-0.4215459	0.00467592
Adamts9	orange	1.36653111	1.05E-06	1.38521661	1.19E-06
Lrig1	red	-1.0103402	2.63E-09	-0.5206566	0.00349558
1700003E16I	skyblue	-1.5879454	6.89E-10	-1.0574412	5.45E-05
Wdr54	red	-0.8399622	0.00171808	-1.0744691	9.81E-05
Wbp1	red	-0.6368855	5.86E-07	-0.6278351	1.51E-06

M1ap	orange	0.38285766	0.42727138	1.51680648	0.00111233
Tacr1	skyblue	-4.5859018	1.36E-06	-2.2381826	0.00364222
Mrpl19	orange	0.81589381	3.63E-08	0.57363163	0.00021372
Bmp10	orange	9.82627151	0.00121101	-10.742795	0.00048251
Arhgap25	orange	1.27983118	2.88E-15	1.13978446	4.68E-12
Gkn3	skyblue	-3.9098689	7.35E-11	-7.4370597	2.22E-08
Rab43	orange	1.71107005	2.33E-08	1.6727655	9.61E-08
Hmces	orange	0.58054546	4.06E-06	0.35990805	0.00671882
Foxp1	red	-0.7229771	8.93E-07	-0.6893183	4.62E-06
Gxylt2	skyblue	-1.6224271	1.41E-10	-0.9079192	0.00079837
Chl1	orange	1.20971742	0.00061846	0.54417818	0.15871249
Plxna1	red	-0.7651453	1.65E-06	-0.3789263	0.02586575
Chchd6	red	-0.654028	0.00014762	-0.3817841	0.03733686
Nup210	orange	1.2649421	2.62E-22	1.25811032	7.79E-22
Cntn6	skyblue	-1.8530184	0.0885198	-3.4200678	0.0095846
Xpc	red	-0.9242107	9.49E-06	-0.6059754	0.00544605
Grip2	skyblue	-1.6779047	1.15E-09	-0.8612214	0.00111246
Bhlhe40	orange	0.51841391	0.00517748	0.58638129	0.00188191
Edem1	orange	1.23003585	9.85E-22	1.09737756	2.66E-17
Usp18	yellow	3.03309456	8.25E-59	3.00089597	2.57E-57
Slc6a12	yellow	2.44798666	0.00629008	3.36190867	0.0005893
Ret	red	-0.9969143	0.00013108	-0.7551985	0.00553924
A2m	yellow	3.40058846	0.00024976	2.75460429	0.00400887
Klrg1	orange	1.77652581	3.47E-06	1.30211463	0.00123253
Mfap5	red	-0.6427945	0.00092249	-0.3080674	0.14421519
Gdf3	black	5.432136	0.00063448	3.31155009	0.00677079
Plxnd1	red	-0.5855988	0.00172804	-0.3320741	0.097391
Lag3	black	4.73500328	2.52E-64	4.50369151	4.65E-57
Lrrc23	skyblue	-1.648105	1.71E-10	-1.1623756	1.31E-05
Rasgef1a	skyblue	-1.7173165	2.40E-20	-1.3813238	2.53E-13
Clec4e	black	5.52238957	9.74E-23	4.4059342	4.64E-17
Clec4d	yellow	4.00745755	7.56E-16	3.51895888	8.22E-13
Zfp248	red	-1.0134222	0.00010477	-1.022748	0.00013813
Clec4a2	yellow	3.48013377	3.82E-16	2.69461023	6.71E-10
Klrk1	yellow	3.8774696	1.90E-48	3.53257781	1.04E-39
Klrb1f	orange	1.78022302	2.67E-05	1.78567446	0.0001106
Cd69	yellow	3.03477559	1.11E-17	3.29036105	1.90E-19
Clec12b	red	-1.0858261	0.00115013	-1.2820783	0.00016175
Clec1b	red	-0.2295159	0.35323928	-1.0879266	2.69E-06
Gabarapl1	red	-0.74104	6.97E-06	-0.5857295	0.00059635
Klrd1	yellow	3.14350741	2.13E-44	2.74158128	1.86E-32
Klrc1	black	5.04939511	1.11E-31	4.7918127	5.11E-27
Klra13-ps	orange	1.28427564	3.00E-05	1.99956186	3.41E-09

Klra2	yellow	3.64688861	1.77E-18	2.94996224	2.41E-12
Magohb	orange	1.74912677	2.67E-14	1.41037571	4.23E-09
Etv6	orange	1.32911412	6.47E-13	1.2365671	4.01E-11
Bcl2l14	orange	0.89682487	0.00055555	0.86099803	0.00114502
Lrp6	red	-0.9192465	7.43E-09	-0.71225	1.43E-05
Emp1	orange	0.76056816	0.00945643	0.8535115	0.00419239
Plbd1	orange	1.26816443	2.46E-09	1.03106689	2.52E-06
Art4	skyblue	-1.4707708	8.38E-12	-1.2825113	4.83E-09
Mgp	orange	0.56555091	0.02812293	0.7199717	0.0055993
Arhgdib	yellow	2.30395588	3.43E-27	2.31216667	3.47E-27
Rerg	red	-0.8934456	6.63E-05	-0.7792076	0.00074331
Ptpro	orange	1.74895157	9.68E-06	1.29800143	0.00159849
Dera	orange	1.60836886	7.76E-13	1.38469277	1.48E-09
Aebp2	orange	0.71851177	7.22E-09	0.59232505	3.57E-06
Ldhb	skyblue	-1.6593725	5.40E-07	-1.1189703	0.0011811
Abcc9	red	-1.4117483	1.72E-08	-1.0934454	2.55E-05
Rad18	orange	1.62902984	6.79E-10	1.74289207	7.50E-11
Sspn	skyblue	-1.7174248	1.96E-07	-1.6589427	8.46E-07
Bhlhe41	skyblue	-2.3250326	3.48E-19	-1.8199108	5.95E-12
Srgap3	orange	0.70981437	2.75E-05	0.33994279	0.06077967
Rassf8	red	-1.183507	1.02E-09	-0.921521	3.82E-06
Lrmp	yellow	2.04344378	9.24E-21	2.38606928	2.32E-27
Bcat1	yellow	2.40049017	3.22E-08	2.58064961	6.52E-09
Ttll3	red	-0.8502716	7.80E-05	-0.5986066	0.00705883
Cidec	skyblue	-2.3523216	0.00348584	-1.3561717	0.11603848
St8sia1	orange	0.94214502	0.0006625	1.55097528	5.25E-08
Creld1	red	-0.6667175	3.24E-05	-0.6318126	0.0001279
Ccdc91	red	-0.9373402	4.62E-08	-0.758992	1.86E-05
Atp2b2	skyblue	-3.6169843	8.65E-18	-1.8789065	6.05E-06
Caprin2	red	-0.8503119	0.00470179	-0.8603995	0.00485234
Tamm41	orange	0.62817899	9.51E-05	0.58941431	0.00042659
Timp4	red	-0.8651618	0.00315351	-0.7083943	0.01811731
Cand2	red	-1.0140845	9.32E-05	-0.5638679	0.04181228
Efcab12	red	-1.211849	1.24E-06	-0.8013646	0.00103539
Ift122	red	-0.990645	3.40E-17	-0.6900733	8.67E-09
Klrb1c	orange	1.14168187	0.00415011	1.76995456	1.82E-05
Pianp	red	-1.3318963	0.00047921	-0.6062061	0.11736503
Cd27	yellow	2.43488522	1.93E-14	2.99669662	1.62E-20
Tnfrsf1a	orange	0.68829969	1.12E-07	0.72395371	3.63E-08
Rad51ap1	yellow	2.58525098	9.92E-14	2.80101264	6.28E-15
Fkbp4	red	-0.980978	4.65E-16	-0.813708	4.00E-11
Klrb1a	yellow	1.10445229	0.18047469	3.58031817	0.00076565
Clec2i	yellow	1.8629233	3.08E-19	2.27011841	2.36E-25

Slc8a2	red	-1.3649121	1.02E-05	-0.7348395	0.02022941
Zfp606	red	-0.6718606	5.56E-05	-0.6220075	0.00031141
Ckm	red	-1.1667208	0.00012385	-0.8351933	0.00851912
Rtn2	red	-1.1569314	1.53E-05	-1.1317295	3.99E-05
Vasp	orange	1.13493125	8.28E-09	1.0084634	5.59E-07
Dmpk	red	-1.4679612	1.50E-15	-1.0561695	2.46E-08
Pglyrp1	orange	1.46957846	2.69E-10	1.25478953	1.46E-07
Pdcd5	orange	0.58070169	0.00010154	0.51952869	0.00077229
Pop4	orange	0.64183089	4.45E-07	0.47800687	0.00031695
Lilra6	orange	1.53687818	0.00032282	1.2643772	0.00353344
Rpl28	orange	0.67180984	2.57E-05	0.53008848	0.00135235
Sbk2	skyblue	-1.854689	7.35E-08	-1.5742019	9.27E-06
Zfp583	red	-0.9118339	0.0034664	-0.7374192	0.02122449
Zfp273	red	-0.8196734	0.00418404	-0.8214352	0.00665188
Nipa2	orange	0.86375554	2.77E-06	0.78607896	3.37E-05
Psd3	red	-1.0626422	1.00E-09	-0.951179	8.37E-08
Zfp719	red	-0.6510426	0.00401514	-0.4102861	0.09017366
Csrp3	skyblue	-2.0615436	2.33E-08	-1.7634328	3.33E-06
Zdhhc13	orange	0.60036065	6.98E-06	0.59832813	1.36E-05
Siglece	orange	1.31971926	0.00029756	1.13546177	0.00256066
Cyp2b10	skyblue	-1.4827135	0.00885927	-1.2759437	0.02988239
Zfp108	skyblue	-2.0986252	5.63E-08	-0.9573542	0.01534389
Faap24	orange	0.98137718	1.36E-08	0.66318248	0.00026836
Slc7a10	skyblue	-2.7175573	3.99E-12	-2.2882686	1.04E-08
Kctd15	red	-0.6821914	1.02E-08	-0.5753511	2.87E-06
Snrpa1	orange	1.12506864	2.27E-10	0.80886637	1.29E-05
Pcsk6	skyblue	-1.8621045	2.10E-15	-1.2468238	2.39E-07
Tarsl2	red	-0.879671	7.73E-06	-0.7624522	0.00021159
Tjp1	red	-0.7443029	1.55E-05	-0.5165387	0.00401979
Apba2	skyblue	-1.6571659	0.00173766	-0.6396192	0.23509991
Mphosph10	orange	0.74150348	1.60E-05	0.37843996	0.04027914
Chrna7	yellow	1.92366612	0.00058605	2.88425857	6.21E-07
Blm	orange	2.00224755	2.29E-13	1.6789603	1.87E-09
Furin	orange	1.02274974	9.43E-07	0.84297776	8.88E-05
Hddc3	red	-1.4086911	0.00050483	-0.9888597	0.01783903
Cib1	orange	0.60606554	0.00016381	0.42791569	0.01133761
Idh2	red	-0.9371183	4.00E-08	-0.8105617	3.65E-06
Mesp2	skyblue	-1.6747995	9.54E-05	-1.3262247	0.00300698
Mesp1	skyblue	-3.6224556	1.76E-08	-1.4045243	0.02111241
Plin1	skyblue	-3.0274686	7.07E-05	-2.0031319	0.0120582
Nr2f2	red	-0.8097704	3.39E-11	-0.6455531	2.62E-07
Pgpep1l	skyblue	-1.903134	5.40E-08	-1.5074689	0.00024059
Synm	skyblue	-2.2731173	2.77E-09	-1.6735397	2.32E-05

Ttc23	red	-0.8022472	7.10E-06	-0.4563635	0.0151002
Rab38	red	-0.891071	7.39E-05	-0.85816	0.00019828
Ctsc	orange	1.4320098	1.03E-05	1.26574736	0.00015109
Nox4	red	-1.1265059	0.00048079	-1.0256491	0.00199972
Tyrobp	yellow	2.75623947	1.25E-17	2.48909016	2.18E-14
2200002D01	orange	0.99536074	1.04E-05	0.79485627	0.00072047
Psmd8	orange	0.86419384	8.71E-08	0.75714264	4.85E-06
Ryr1	red	-0.7231578	0.00674464	-0.3059664	0.30003162
Nfkbib	orange	1.21352856	8.76E-15	1.01857966	1.80E-10
Psmc4	orange	0.88505651	8.26E-09	0.74310396	2.50E-06
Mfge8	red	-0.7769267	8.80E-12	-0.666346	9.66E-09
Aen	orange	0.83833744	5.05E-07	0.75907589	9.61E-06
Mrps11	orange	0.63053575	0.00220337	0.19884753	0.39268573
Ccdc90b	red	-0.5986258	0.00085125	-0.6209784	0.00075241
Eed	orange	0.70691731	1.61E-07	0.52369015	0.00019544
Me3	red	-1.22719	0.00484566	-0.8213806	0.07514179
Ddias	yellow	2.74059337	4.84E-08	1.5228549	0.00283622
Anapc15	orange	0.84977443	7.64E-06	1.01430685	2.06E-07
Tmc5	red	-1.3145099	0.00583603	-0.4621644	0.37452102
Xylt1	orange	1.42303394	3.23E-08	1.31626659	7.09E-07
Calca	orange	1.50875554	7.91E-05	1.26299794	0.00055603
Mylpf	red	-1.452933	0.00010376	-1.0241106	0.00889071
Qprt	orange	1.67989521	4.64E-05	1.71851172	8.30E-05
Kif22	yellow	2.7132151	3.41E-31	2.99102754	4.71E-36
Mvp	orange	1.23158083	7.38E-14	1.15692431	3.67E-12
Stard10	red	-1.10143	1.55E-05	-1.0204683	9.52E-05
Ppp4c	orange	0.75921203	3.38E-07	0.59664847	0.00010806
Tbx6	red	-1.3666544	3.35E-06	-0.7973679	0.00843595
Plekhb1	skyblue	-1.9213134	8.63E-09	-1.2334793	0.00038567
Coro1a	yellow	2.82304986	2.66E-44	2.74345689	9.21E-42
Klk7	skyblue	-1.8174651	0.00298746	-1.2582319	0.05254871
Nupr1	orange	0.79363102	1.62E-07	0.74486576	1.54E-06
Cln3	orange	1.01215898	1.13E-08	0.97723175	6.38E-08
Pold3	orange	0.89535485	1.06E-09	0.55784445	0.00027483
Pgm2l1	skyblue	-1.3294562	5.35E-05	-1.2620498	0.00018811
Syt3	skyblue	-1.2857409	0.00321185	-1.4852818	0.00086195
Slco2b1	red	-1.4520502	0.00019582	-0.7937464	0.05592166
Myh14	red	-1.2422954	4.04E-06	-0.7972822	0.00472948
Lat	yellow	2.67247289	8.17E-13	3.42834641	3.89E-20
Il21r	yellow	2.94239359	1.31E-17	3.58509302	4.29E-25
Dgat2	skyblue	-1.9071743	8.66E-10	-1.7642625	2.48E-08
Il4ra	orange	1.77075085	1.63E-24	1.65828051	1.55E-21
Nsmce1	orange	0.75580844	5.09E-13	0.66005242	6.75E-10

Psma1	orange	0.9223171	6.83E-07	0.66296053	0.00060166
Parva	red	-0.7077399	8.87E-08	-0.5056353	0.00023041
Dkk3	red	-0.1725131	0.48450253	-0.6339541	0.0056278
Pak1	orange	0.606749	0.00374562	0.36170802	0.10795072
Cox6a2	skyblue	-1.173406	0.00288071	-1.4316366	0.00034807
Itgam	yellow	3.45628272	2.68E-08	3.61967836	9.68E-09
Lyve1	orange	1.00037951	4.59E-08	0.99194648	9.66E-08
Rnf141	red	-0.6411716	0.00011121	-0.5272903	0.00211193
Itgax	yellow	2.23198302	9.44E-09	1.82166323	5.23E-06
Adm	orange	1.49933526	3.98E-09	1.45411113	1.22E-08
Pycard	orange	1.81297018	2.80E-23	1.48566072	2.35E-15
Tead2	red	-1.292317	5.83E-15	-1.1956104	9.26E-13
Cd37	orange	1.19686059	9.78E-08	1.20273378	1.45E-07
Prss8	red	-1.4287264	1.52E-06	-0.9879301	0.00139985
Itgal	yellow	2.32730446	2.46E-26	2.23847868	2.52E-24
Abcc6	skyblue	-3.2333684	0.00012244	-0.8263654	0.28318488
Sergef	orange	0.78903418	5.73E-05	0.65757736	0.00120855
Rgs10	orange	0.98796618	6.34E-06	0.78939166	0.00054985
Fgfr2	red	-1.4333452	2.33E-06	-0.7627288	0.01778265
Cpxm2	red	-1.2366901	6.95E-05	-0.7097073	0.03026601
Chp2	skyblue	-2.2289654	4.04E-05	-1.3929077	0.01234153
Plk1	yellow	2.60109993	1.10E-26	2.64685092	5.90E-27
Scnn1b	red	-0.9817028	0.00029701	-0.5918982	0.03959877
Vwa3a	red	-1.4403119	9.09E-07	-0.9103303	0.00272695
Hpx	orange	0.76726668	0.01090471	1.00712396	0.00090151
Tmem159	red	-0.7998761	5.87E-06	-0.6293069	0.00057036
Trim30a	yellow	2.78122713	9.33E-60	2.72232028	4.35E-57
Lyrm1	orange	0.78258411	0.00677473	0.71755159	0.01679439
2610020H08	orange	0.89123469	0.00113728	0.57998551	0.04941739
Acsm3	skyblue	-1.4508873	0.0137203	-1.6091172	0.00756147
Trim21	orange	1.5403653	4.20E-40	1.65852959	1.76E-46
Rrm1	orange	1.46930869	1.63E-25	1.19684668	5.46E-17
Uros	red	-0.7757079	4.90E-07	-0.6970997	1.39E-05
Art1	red	-1.26338	0.00323908	-1.0031605	0.02536736
Mki67	yellow	3.22656009	7.95E-44	3.66543711	3.78E-55
Wee1	red	-0.2835942	0.13404963	-0.7323154	5.97E-05
BC051019	red	-1.1942614	8.87E-07	-0.6776363	0.00742792
St5	red	-0.8138986	1.43E-05	-0.5666098	0.00373984
Stk33	skyblue	-2.0790266	7.45E-11	-1.5268526	2.20E-06
Mrgprf	skyblue	-2.0902841	5.71E-17	-1.0215482	7.15E-05
Ano1	red	-1.1251635	1.07E-05	-0.654962	0.01512419
Dock11	orange	1.37496964	6.61E-26	1.33783348	6.39E-24
Tnni2	red	-1.3228721	7.91E-05	-0.863371	0.01428355

Syt8	orange	1.23386206	0.00759078	1.51737427	0.00122065
Smarca1	skyblue	-1.51988	0.00267476	-1.113198	0.03441917
Sash3	yellow	2.09282611	6.96E-33	2.44817312	6.48E-44
Elf4	orange	1.55818727	3.16E-13	1.47688257	8.62E-12
Rbmx2	orange	1.02009477	2.84E-05	1.0022323	8.39E-05
Igsf1	skyblue	-1.9175551	0.00019595	-1.8103812	0.00072153
Stk26	orange	1.32152558	2.94E-10	0.79034763	0.00031269
Cd40lg	yellow	2.40159608	0.0003975	3.2936925	5.92E-06
Syp	red	-1.1131508	4.91E-05	-1.164957	3.87E-05
Magix	skyblue	-3.3854487	2.95E-19	-2.2487393	2.17E-09
Pim2	orange	0.63579836	0.00427975	0.59607362	0.01021087
Was	yellow	2.62763893	2.21E-17	2.47241732	2.84E-15
Rbm3	orange	1.9071651	3.56E-21	1.62253928	2.03E-15
Porcn	red	-0.6032081	3.02E-09	-0.6083792	3.00E-09
Slc38a5	skyblue	-2.2601748	4.84E-24	-1.6050193	2.19E-12
Rpgr	red	-0.8995619	0.00024632	-0.7599938	0.00270343
Dynlt3	red	-0.7973188	4.81E-16	-0.6399819	1.77E-10
Heph	red	-1.1360628	5.23E-05	-0.9972321	0.00055724
Stard8	red	-1.0091763	0.00026725	-0.6415977	0.02802595
Pbdc1	orange	0.78835376	0.00459827	0.58239293	0.04680769
Magee1	red	-0.9415124	9.32E-09	-0.8044975	2.20E-06
Fgf16	skyblue	-1.7644531	0.00337777	0.0986804	0.89105193
Srpx2	red	-1.2627915	2.83E-07	-0.9445347	0.00019478
Sytl4	red	-0.956414	2.77E-07	-0.5565796	0.00424558
Cenpi	yellow	2.32456685	5.21E-09	2.6276115	8.36E-11
Btk	orange	1.59076388	1.92E-09	1.61078757	2.81E-09
Gla	orange	1.54925305	3.23E-08	1.41550415	7.95E-07
Col4a6	skyblue	-2.1807847	2.89E-16	-1.0596083	0.00012492
Col4a5	red	-1.0515147	0.00015292	-0.6374098	0.02989418
Chrdl1	skyblue	-2.0875533	4.00E-06	-1.5161764	0.00123362
Glt28d2	red	-0.6798211	0.01789624	-1.1154887	0.00011895
Lrch2	red	-1.3483082	7.52E-05	-0.9616603	0.00519135
Cdkl5	skyblue	-1.9577097	3.05E-08	-1.3619292	0.00020487
D630029K05	skyblue	-2.1407875	4.10E-07	-1.6854674	0.00013693
Phka2	orange	0.74267929	3.25E-11	0.51665286	7.87E-06
Pdha1	red	-0.6387063	0.00040675	-1.405222	1.06E-15
Il2rg	orange	1.91084166	1.87E-20	1.63634344	4.80E-15
Itgb1bp2	red	-1.0449127	7.91E-06	-0.8170545	0.00081829
Gabre	red	-0.907691	0.00049424	-0.344826	0.22519106
Gabra3	skyblue	-1.6872482	2.24E-16	-0.6045066	0.00384251
Gabrq	red	-1.7509221	0.00038054	-0.553643	0.28440814
Xlr4c	yellow	2.9183785	3.03E-12	2.45558143	2.15E-09
Haus7	orange	0.55879077	0.00048033	0.66878197	4.97E-05

Bmx	skyblue	-1.9211784	1.48E-09	-1.4319655	8.52E-06
Pir	red	-1.2271144	3.81E-08	-1.1746959	2.43E-07
Asb11	red	-0.7087071	0.0310905	-0.9633904	0.00487556
Arhgap4	orange	1.35983864	1.66E-17	1.67042826	1.18E-25
Avpr2	yellow	2.23537208	2.85E-07	2.36568536	1.02E-07
Plxna3	red	-0.9187669	0.00014478	-0.738999	0.00320631
G6pdx	orange	0.63088771	5.58E-05	0.54486181	0.00072664
Dkc1	orange	0.61493549	0.00208876	0.31849132	0.14272206
Plp1	red	-1.1008132	2.51E-05	-0.7078341	0.01031583
Zcchc18	orange	0.98709014	0.00121389	0.85931431	0.00755825
Psmd10	orange	0.71808422	0.00304984	0.32729247	0.22052191
Morc4	red	-1.4582941	9.49E-11	-0.8886538	9.28E-05
Mcf2l	skyblue	-1.2889566	5.85E-05	-1.4881219	4.70E-06
F7	orange	1.09001734	0.00017387	0.73397223	0.01586236
F10	yellow	3.45394044	1.45E-17	2.89071866	1.32E-12
Gas6	red	-1.0130854	7.44E-06	-0.5759525	0.01593696
Rasa3	orange	0.55096125	2.21E-06	0.58358817	8.08E-07
Myom2	red	-0.968078	0.00212476	-1.1330191	0.00040777
Thsd1	red	-0.9122229	8.63E-08	-0.6763469	0.00012921
Adrb3	skyblue	-3.1779797	6.93E-06	-2.2573705	0.00205646
Cd209a	skyblue	-3.2661037	4.28E-13	-2.7834739	1.26E-09
Col4a1	orange	0.97828681	6.35E-07	1.10413756	2.66E-08
Col4a2	orange	0.82058527	1.77E-05	0.9554523	7.91E-07
Rab20	yellow	2.18635698	1.76E-19	1.97051916	8.29E-16
Ptpn7	yellow	2.72097398	1.77E-25	3.01887067	1.19E-30
Gpm6a	skyblue	-1.5768898	5.62E-09	-1.3803542	6.50E-07
Asb5	red	-1.1561818	0.00147159	-1.0139568	0.00839866
Vegfc	red	-0.6267286	0.00661394	-0.4918411	0.04112175
Dlc1	red	-0.9132913	6.25E-12	-0.6625494	1.34E-06
Eri1	orange	0.66391935	5.15E-05	0.68041665	5.10E-05
Plat	orange	1.55429128	6.52E-08	1.72308474	2.88E-09
Ank1	skyblue	-1.128475	0.00308466	-1.6452378	2.03E-05
Sfrp1	yellow	2.32075186	1.62E-15	1.84703525	5.59E-10
Ido2	yellow	2.55400157	4.17E-06	2.15781256	9.17E-05
Ido1	black	5.54105876	3.81E-124	5.95923996	2.70E-127
Adam3	skyblue	-1.3916658	0.00083033	-1.6128222	0.00011894
Adam5	skyblue	-2.1056016	4.45E-05	-2.2007477	4.42E-05
Slit2	red	-0.6714342	0.00057811	-0.5276663	0.00936017
Tenm3	red	-1.1761093	2.08E-05	-0.8170013	0.00581608
Dctd	yellow	2.11849346	8.92E-06	2.21716219	5.46E-06
Mak16	orange	0.85670428	4.23E-06	0.65193818	0.00070867
Wrn	orange	0.59294728	0.00015928	0.44125593	0.0070598
Gsr	orange	0.84559436	4.84E-05	0.93912484	8.99E-06

Fgl1	yellow	3.68068538	9.84E-06	1.95440127	0.00188057
Galnt7	orange	0.9532752	1.34E-06	0.55474293	0.00771822
Sap30	orange	1.46899408	3.05E-07	1.14998484	0.0001173
Nr3c2	red	-1.3106629	7.90E-06	-1.0476164	0.00054834
Irf2	orange	0.72214961	8.12E-12	0.52840759	1.23E-06
Casp3	orange	1.93642801	1.60E-14	1.91046966	6.25E-14
Cenpu	orange	1.2986388	6.50E-05	1.41144062	1.83E-05
Pdlim3	red	-0.8939488	0.00025348	-0.663358	0.00946768
Tlr3	orange	1.02616668	4.75E-08	0.96661851	4.53E-07
Nek1	red	-0.9363282	6.54E-10	-0.8792617	1.30E-08
Mfap3l	red	-1.3836034	0.00024884	-0.839157	0.03542594
N4bp1	orange	1.27446652	1.63E-14	1.09022856	1.08E-10
Adcy7	orange	0.5765469	0.00203345	0.61895981	0.00117852
Snx20	yellow	2.75444599	3.94E-29	2.52406616	1.84E-24
Aktip	skyblue	-1.4509383	1.25E-10	-1.2117499	1.53E-07
Gins3	orange	0.59913002	0.02398348	0.86889065	0.00148336
Orc6	orange	0.592503	2.02E-05	0.37244306	0.01115821
Itfg1	red	-0.6654934	6.52E-15	-0.6301653	4.85E-13
Gab1	red	-0.7228706	0.00050535	-0.573264	0.00790424
Hp	red	-0.7500038	0.00016325	-0.1840984	0.42042746
Ces1f	skyblue	-3.1565829	1.05E-09	-2.1120321	6.86E-05
Irx3	red	-1.1850039	1.54E-07	-0.6802871	0.00413949
Crnde	skyblue	-1.5185253	1.56E-05	-1.1018571	0.00235481
Irx5	skyblue	-1.5103381	6.99E-12	-0.8952726	8.16E-05
Il34	red	-1.1411641	1.41E-06	-0.9926438	4.51E-05
Bbs2	red	-1.1928103	4.02E-07	-0.9813708	5.12E-05
Cenpn	yellow	2.23327941	2.16E-21	2.27910497	3.50E-21
Mt4	red	-1.1770014	0.00058445	-0.8277723	0.02133633
Cdyl2	orange	0.90881708	7.08E-05	1.14295241	1.30E-06
Mt2	yellow	3.6175676	2.87E-20	3.21718201	1.25E-16
Mt1	yellow	2.51496187	2.44E-44	2.13903694	2.40E-32
Nudt7	red	-0.9601881	5.23E-05	-0.8127052	0.0008996
Herpud1	red	-0.9218837	0.00063226	-0.8923571	0.0012624
Pllp	red	-1.1541832	4.01E-05	-1.0116725	0.0004375
Ciapin1	orange	0.86821309	2.08E-08	0.71372001	7.72E-06
Coq9	red	-0.8818781	3.69E-11	-0.7462528	4.66E-08
Drc7	skyblue	-2.0331614	8.80E-24	-0.8315061	5.64E-05
Katnb1	orange	0.68938214	3.61E-09	0.67281928	1.20E-08
Cngb1	skyblue	-1.7701323	0.00052362	-1.6072885	0.00255518
Mmp15	skyblue	-1.8920782	4.02E-11	-1.2126124	4.73E-05
Tmem38a	red	-1.260192	2.04E-11	-1.0950819	1.31E-08
Usb1	orange	1.11096611	7.29E-09	1.06163666	5.34E-08
B3gnt3	orange	0.86120316	2.26E-06	0.76645465	4.17E-05

Jak3	orange	0.9794156	2.21E-12	0.774091	6.35E-08
Slc27a1	red	-0.5838657	0.00716466	-0.7105102	0.00122749
Mvb12a	orange	0.73236879	6.52E-10	0.51159914	3.26E-05
Gins2	yellow	2.3998873	1.03E-11	2.2555799	3.16E-10
6430548M08	red	-0.8112944	3.23E-05	-0.738407	0.00023193
Crispld2	orange	0.666943	0.00093143	0.40248346	0.06042649
Cotl1	yellow	2.79839074	3.36E-22	2.41256518	1.39E-16
Dnaaf1	skyblue	-2.101146	5.42E-18	-1.172141	1.29E-06
Pik3r2	red	-0.6096796	1.52E-06	-0.6466533	5.34E-07
Ifi30	yellow	2.15413602	7.97E-41	1.66186882	1.52E-24
Rab3a	skyblue	-1.484961	2.02E-08	-1.219751	6.89E-06
Cdh13	skyblue	-1.652095	3.02E-07	-1.0373386	0.00205857
Mphosph6	orange	0.71765734	0.00018537	0.5941485	0.00296149
1700030J22F	red	-0.8723041	0.00604647	-0.7614949	0.01589888
Lsm4	orange	0.85480423	4.81E-06	0.7882298	4.18E-05
Comp	skyblue	-1.2300033	0.0094267	-1.9178388	7.11E-05
Ntpcr	orange	1.09510895	2.36E-05	0.79993758	0.00311105
BC021891	skyblue	-1.7074306	0.0003193	-1.1425662	0.01661621
Atp13a1	orange	0.75577943	7.91E-08	0.75396786	1.36E-07
Pgr	skyblue	-1.3328766	0.02004688	-1.5505035	0.00850312
Cdh5	red	-0.6149081	7.64E-05	-0.4551237	0.00491752
Bean1	yellow	2.19065411	1.79E-05	1.75992859	0.0006221
Ces2g	skyblue	-1.6384592	3.02E-07	-1.1197321	0.00076295
Cdh16	skyblue	-2.7625922	3.06E-06	-1.5720275	0.010878
Cbfb	orange	0.80962881	8.96E-06	0.63697875	0.00075488
Ces2e	skyblue	-2.4758385	2.13E-06	-2.8915624	1.65E-07
Tradd	orange	0.76443337	1.09E-05	0.56955166	0.00170338
D230025D16	red	-0.980706	1.96E-05	-0.6993072	0.00341653
Hsd11b2	orange	1.73940096	0.00397175	0.02454015	0.97477362
Tsnaxip1	skyblue	-2.0242513	9.84E-12	-1.1676427	0.00011264
Ctrl	orange	1.0123714	0.01188873	1.38009952	0.00087538
Psmb10	yellow	2.23710606	5.13E-75	2.04840341	8.02E-63
Dpep3	yellow	2.46912937	0.00013465	1.4846336	0.04379761
Slc7a6	orange	0.59906091	5.27E-08	0.66025473	2.58E-09
Smpd3	red	-0.6341268	0.00890691	-0.2388636	0.37414889
Zfp90	orange	0.45027171	0.10487137	0.79906837	0.00370789
Has3	red	-1.4440686	6.02E-08	-1.0174158	0.00016435
Nip7	orange	0.76368702	0.00015283	0.68340063	0.00103577
Mre11a	orange	0.65245066	0.00012493	0.43823789	0.01461758
Ankrd49	orange	0.58168429	0.00140733	0.40426702	0.03611018
Izumo1r	yellow	3.22747684	7.16E-16	2.94907489	4.99E-13
Panx1	orange	1.52454692	2.16E-15	1.52679425	5.03E-15
Vstm5	orange	1.00383657	0.00485213	0.41296265	0.28423471

4931406C07	red	-1.0578704	2.53E-12	-0.8083946	1.81E-07
Taf1d	orange	1.19297152	1.25E-12	1.04399324	1.25E-09
Kars	orange	1.04335053	1.21E-09	0.92153928	1.48E-07
Tmem231	skyblue	-1.7254597	1.17E-19	-1.2231922	1.01E-10
Ldhd	red	-0.8008078	0.00498938	-0.6685144	0.02387961
Aars	orange	0.92470009	8.41E-10	0.93957478	7.04E-10
Glb1l3	skyblue	-3.0444105	3.59E-06	-1.8706291	0.00460951
Afg3l1	orange	0.58464111	0.00015884	0.50870421	0.00147099
Acta1	red	-1.1307921	0.00153315	-0.6921598	0.06836149
Urb2	orange	0.70142495	5.61E-05	0.96402878	5.05E-08
Gnpat	red	-0.6661061	3.20E-08	-0.4411841	0.00044436
Egln1	red	-0.6400563	2.63E-07	-0.6132921	1.39E-06
Jam3	red	-0.9387214	7.21E-08	-0.862511	1.69E-06
Adamts8	red	-1.0900059	8.62E-06	-0.6977577	0.00647695
Aplp2	red	-1.1879659	8.88E-13	-0.8840022	2.40E-07
Trpc6	red	-1.0275217	5.53E-14	-0.904623	6.54E-11
Birc3	orange	1.31123189	8.27E-27	1.14789907	9.97E-21
Pdgfd	skyblue	-1.1857647	2.96E-13	-1.2556964	1.50E-14
Sesn3	orange	0.41349265	0.00507601	0.60196256	5.32E-05
Usp2	red	-1.0705383	5.03E-05	-1.2450403	3.39E-06
Thy1	yellow	2.66608224	3.21E-16	3.05153257	8.76E-21
Ubash3b	yellow	2.24669607	2.02E-21	1.8741648	5.46E-15
Crtam	yellow	4.54198276	1.33E-20	4.06980517	3.12E-17
4931429I11R	red	-0.9752041	0.00485892	-0.5052124	0.14719645
Nxpe2	skyblue	-1.4594445	7.92E-06	-1.2937395	0.00011465
Barx2	red	-0.9680851	0.00209699	-0.7714101	0.01948904
Kirrel3	red	-1.4762985	0.00022857	-0.1595447	0.73338212
Abhd12	orange	0.7381072	0.00014188	0.61163399	0.0023379
4833427G06	skyblue	-1.6025106	3.01E-08	-1.0963059	0.00018071
Cryab	red	-0.7785107	2.80E-06	-0.5675276	0.00101965
2310030G06	red	-0.6756747	0.00026367	-0.3313729	0.09441347
Dixdc1	skyblue	-2.6377922	1.08E-10	-1.6087174	0.00014449
Plet1	red	-1.0352646	4.68E-05	-0.5781187	0.03117299
Cadm1	red	-1.4711483	4.46E-11	-1.0607575	4.29E-06
Bace1	red	-0.7335607	4.56E-06	-0.6321488	0.0001263
Il10ra	yellow	3.40486514	2.83E-35	2.91014657	6.51E-26
Cd3e	yellow	3.0336001	2.09E-24	3.16545561	7.57E-26
Cd3d	yellow	3.38090879	1.43E-24	3.71255432	1.83E-28
Nlrx1	orange	0.68659813	3.75E-06	0.44679298	0.0040822
Chek1	orange	1.89372488	1.73E-07	1.87113278	6.31E-07
Fez1	skyblue	-1.926269	2.20E-07	-0.7825037	0.04623936
Tmem218	red	-0.7145238	8.70E-06	-0.5549437	0.00089528
Slc37a2	yellow	2.33146002	5.54E-10	1.99119612	2.21E-07

Olfm2	skyblue	-1.7200251	0.00038954	-2.4190743	6.39E-07
Icam5	orange	0.66767371	0.17240801	1.54860332	0.0014106
Pde4a	red	-0.7266458	1.88E-05	-0.7941633	4.16E-06
Bmp5	red	-1.0855314	1.07E-10	-0.746736	1.95E-05
Scg3	skyblue	-1.4231912	0.00715687	-1.3413811	0.01774919
Gnb5	red	-0.9486697	3.58E-06	-0.7063245	0.00083177
Ldlr	orange	0.55858474	0.01045965	0.62862262	0.00457663
Kank2	red	-0.7887041	1.10E-05	-0.6489005	0.00046612
Dock6	red	-1.0376633	8.14E-08	-0.8400701	2.49E-05
Aqp9	orange	0.98184377	0.01902973	1.54644262	0.00045838
Nedd4	red	-0.9121798	4.66E-12	-0.7501193	2.75E-08
Ccnb2	yellow	3.53827404	1.08E-57	3.82187596	4.54E-62
Fam81a	skyblue	-2.3609717	9.36E-19	-1.4583404	2.31E-08
Anxa2	orange	0.65453919	0.00013574	0.6099261	0.00054599
Cgnl1	red	-0.8273988	2.44E-09	-0.3775537	0.01046814
Rora	red	-0.5906169	0.00022557	-0.6973684	1.81E-05
Itga11	red	-0.8918707	0.00105568	-0.8590605	0.00222899
Calml4	red	-1.5125995	8.73E-08	-1.0697282	0.00022652
Irak1bp1	red	-0.7744803	0.00312808	-0.2410603	0.41691565
Kif23	yellow	1.88696375	1.92E-19	2.10990251	5.43E-23
Lca5	red	-1.2169324	1.45E-14	-0.5319348	0.00078183
Fam46a	orange	1.48064958	1.40E-13	1.10648308	8.24E-08
Tmprss5	skyblue	-1.768821	2.65E-05	-0.9797562	0.03154098
Paqr5	red	-0.9991589	0.00518763	-1.0209354	0.00581608
Tle3	orange	0.66720399	1.70E-07	0.88175329	4.33E-12
Dnaja4	skyblue	-1.3965756	1.52E-14	-1.1757635	2.17E-10
Thsd4	red	-0.9257729	3.54E-06	-0.5108483	0.01505384
Pkm	orange	1.25646211	8.14E-10	1.06889817	3.33E-07
Commd4	orange	0.62332348	5.59E-09	0.58339521	1.02E-07
1700017B05I	orange	1.96685196	1.22E-14	1.51149584	7.34E-09
Psma4	orange	1.13305214	2.11E-12	0.90545716	4.64E-08
Csk	orange	0.61377057	2.69E-06	0.60375295	6.23E-06
Cyp1a1	skyblue	-4.5267367	1.70E-10	-3.5212443	1.07E-06
Rcn2	red	-0.5850002	1.61E-08	-0.4286731	6.78E-05
Pstpip1	yellow	3.47880003	6.92E-43	3.46417261	3.11E-42
Hmg20a	red	-0.5936537	0.00026617	-0.457321	0.00696476
Col12a1	red	-1.1992567	9.83E-07	-0.4866911	0.06683918
Loxl1	orange	0.72139756	0.07212407	1.15495815	0.00367502
Hcn4	skyblue	-3.320111	9.13E-09	-1.752245	0.00215364
Mb21d1	orange	2.17342022	2.11E-16	1.34699638	6.39E-07
Gsta4	red	-0.5427126	0.00997901	-0.6191567	0.00381496
Elovl5	red	-0.5900725	7.04E-05	-0.5259254	0.00058521
Lrrc1	red	-0.9613273	1.32E-08	-0.7287949	3.16E-05

Mlip	red	-1.0916356	0.001685	-1.1007521	0.00217891
Tinag	skyblue	-2.2242875	8.66E-17	-1.7321659	3.22E-10
Ctsh	orange	1.67369269	3.43E-11	1.40518558	5.36E-08
Adamts7	red	-0.6396894	0.00659256	-0.8721921	0.00024236
Tpm1	red	-0.8267051	2.09E-08	-0.5568651	0.00028888
Lactb	orange	0.70847456	7.40E-06	0.49266033	0.00313918
Plod2	red	-0.9387689	3.11E-07	-0.9392375	5.12E-07
Aph1b	red	-1.0612478	2.53E-07	-0.9915159	2.16E-06
Plscr4	skyblue	-1.5021777	3.12E-13	-1.3774305	4.37E-11
Fam96a	orange	0.87671856	6.77E-06	0.68776291	0.00069004
Rbpms2	red	-1.1556874	7.45E-10	-0.8097827	2.70E-05
Spg21	orange	0.66182529	4.82E-06	0.44035345	0.00368169
Tipin	orange	0.87888009	5.75E-06	0.77519681	0.00011167
Zwilch	yellow	2.3765427	4.50E-12	2.11979988	1.40E-09
Lctl	skyblue	-3.1561161	0.00155038	-2.9699365	0.0014556
Me1	red	-1.0760571	3.43E-07	-1.1906702	2.35E-08
Nt5e	orange	0.71492618	0.0025666	0.7645877	0.00157538
Snx14	red	-0.6787387	0.00011165	-0.5533306	0.00234014
Zfp949	red	-0.631915	0.00524655	-0.391758	0.10527907
Cmtm7	orange	1.7319061	2.48E-19	1.4284928	5.38E-13
Stt3b	orange	0.8182978	1.74E-13	0.40315269	0.00057499
Eomes	yellow	2.6279439	1.90E-20	2.08687484	3.70E-13
Slc25a36	red	-0.8138309	1.21E-07	-0.5239428	0.00108997
Clstn2	skyblue	-1.5164355	1.23E-06	-1.3608091	2.14E-05
Mrps22	orange	0.70284875	5.86E-05	0.55591865	0.00214478
Faim	orange	0.96227732	1.19E-06	0.90957249	7.98E-06
Dbr1	orange	0.77263558	1.46E-09	0.49461263	0.00020551
Cldn18	red	-0.9252993	9.81E-08	-0.6180227	0.0006365
Cdc25a	orange	0.58783479	7.15E-05	0.74350313	7.73E-07
Cspg5	red	-1.4495384	0.00839235	0.17337116	0.80917766
Ngp	yellow	2.25956392	0.00608516	3.56924817	0.00081895
Ptgs2	orange	0.6490943	0.00098867	0.80032409	3.70E-05
Kif9	red	-1.1933377	4.07E-09	-0.9817305	1.87E-06
Tdgf1	orange	1.41584236	0.00028178	0.95299118	0.01710863
Lrrc2	skyblue	-1.342158	1.96E-06	-1.2842438	1.23E-05
Dclk3	skyblue	-1.7004452	4.85E-05	-1.7273849	6.19E-05
Trib1	orange	0.73894918	0.00026389	0.79689797	0.00011438
Stac	skyblue	-2.7265641	2.54E-05	-0.6848064	0.24943829
Myd88	orange	2.02456214	1.16E-36	1.79678644	5.57E-29
Scn5a	skyblue	-2.3280783	0.00140008	-1.7957654	0.01787464
Ttc21a	skyblue	-1.952902	2.26E-16	-0.8440619	0.0007034
Csrnp1	yellow	2.55000307	4.22E-30	2.00912715	6.19E-19
Rpsa	orange	1.02007767	1.64E-10	0.74948306	5.46E-06

Hhatl	skyblue	-1.1383993	0.00216436	-1.6763315	7.93E-06
Nktr	orange	0.68573133	2.62E-07	0.67164631	7.45E-07
Pccb	red	-0.937737	3.11E-07	-0.7727992	4.37E-05
Vipr1	red	-1.292996	0.00070486	-0.9381801	0.01851602
Ryk	red	-0.680327	8.31E-07	-0.654124	3.66E-06
Rab6b	skyblue	-1.6938094	2.72E-09	-1.3721702	2.73E-06
Trf	red	-0.9012384	0.00288705	-0.6465827	0.04464584
Topbp1	orange	0.93271367	1.83E-12	0.84366418	3.92E-10
Dnajc13	orange	0.66278804	3.63E-07	0.648201	1.09E-06
Acpp	red	-0.8653275	0.00187585	-0.081048	0.81019753
Aste1	orange	0.93638475	0.00014843	0.58993456	0.02299474
Manf	orange	0.931134	2.12E-07	0.63041023	0.00076168
Mapkapk3	orange	1.1738594	4.13E-08	1.0347658	2.43E-06
Cish	orange	0.81750664	5.94E-08	0.49424585	0.0017023
Traip	yellow	2.28802275	1.69E-13	1.85507117	2.59E-09
Bsn	orange	1.25818868	0.00017652	1.09451564	0.00151286
Mst1	red	-0.9797815	0.0019968	-1.0908549	0.00062469
Cdhr4	red	-1.349028	1.89E-08	-0.5518431	0.02867905
Uba7	yellow	2.14919849	2.43E-61	2.12771802	3.80E-60
Nicn1	red	-1.0455921	5.15E-14	-1.0622222	3.05E-14
Amt	red	-0.9402238	7.56E-06	-0.9947852	3.68E-06
Klhdc8b	skyblue	-1.6259075	1.08E-11	-1.3045468	1.06E-07
Pygm	red	-1.0730563	5.26E-05	-0.840232	0.00227319
Colgalt2	skyblue	-2.8243163	4.94E-13	-2.0848896	1.87E-07
Crebl2	red	-1.2976534	1.05E-05	-0.9654835	0.00157335
Oas3	black	6.53452424	1.38E-34	6.17445452	5.47E-31
1700025G04	red	-0.7028376	0.00014574	-0.603985	0.00159921
Cd59a	skyblue	-1.5243768	8.70E-09	-1.3912662	2.77E-07
6820408C15	red	-0.8965704	6.36E-05	-0.9274982	5.02E-05
Malt1	orange	1.70215753	3.55E-23	1.39904863	9.04E-16
Oas2	yellow	3.7181752	7.87E-39	3.82395303	6.53E-41
Nlrp3	yellow	2.89768173	4.48E-14	2.78630425	5.92E-13
Kank1	red	-0.8346273	3.14E-06	-0.575444	0.0020177
Exd2	red	-0.8251732	4.70E-17	-0.7881001	1.04E-15
2810474O19	orange	0.64257647	0.00121257	0.59041915	0.00390452
Trib3	orange	0.91064588	5.14E-05	0.82302897	0.00034986
Mansc1	red	-0.688093	3.87E-05	-0.4397162	0.01168347
Sbspon	red	-1.1639729	0.00442212	-1.0153824	0.01061428
Abtb2	orange	1.67016039	6.14E-14	1.56753651	3.14E-12
Snx33	red	-0.6306255	0.00019951	-0.4952059	0.00484565
Ablim3	red	-0.9375992	3.44E-06	-0.5561439	0.00888342
Tpcn1	red	-1.1982281	1.64E-09	-1.0668529	1.43E-07
Chrm1	skyblue	-3.2886243	9.06E-10	-2.6471305	4.66E-07

Mctp2	orange	1.97973908	8.55E-10	1.69844666	2.30E-07
Troap	yellow	3.59814003	2.93E-35	4.2639286	5.78E-40
Alas1	red	-0.7223023	0.00075633	-0.7116084	0.00120782
Pdxk	red	-0.5620827	0.00041408	-0.7562421	2.14E-06
Lama1	yellow	2.17702001	4.64E-06	2.73601238	6.17E-08
Srxn1	orange	0.69505156	0.02858374	0.95088889	0.00285183
Fanca	orange	1.30152658	2.39E-12	0.87068297	4.57E-06
Igdcc4	red	-1.3651654	3.68E-14	-0.9743191	4.24E-07
Ank2	red	-0.9316028	0.00036478	-0.7361329	0.00723169
Ppp1r9a	red	-1.1245667	0.00024332	-0.7148105	0.02707912
Trpc1	red	-1.0117557	6.90E-05	-0.6765278	0.01087342
Abcc4	orange	0.62534712	0.00205966	0.74019778	0.00034083
Ugt8a	skyblue	-3.4643538	2.57E-07	-1.384363	0.01173294
Pkd1	red	-0.6516685	0.00081149	-0.550373	0.00629147
P2ry2	orange	0.58594293	0.06005977	0.93468356	0.00251067
Smap2	orange	0.92047157	3.37E-12	0.69348491	3.63E-07
Arhgef17	red	-0.8360736	3.15E-08	-0.6127403	8.94E-05
Ccdc85a	skyblue	-1.2322734	3.43E-07	-1.2378681	4.64E-07
Acsl3	red	-1.1993665	3.56E-07	-0.8558411	0.00046837
Fbxo21	red	-0.8899206	1.06E-07	-0.6845323	7.50E-05
Styk1	yellow	2.05531576	8.60E-10	2.1246276	5.30E-10
Cspg4	red	-0.8172552	1.80E-07	-0.5786327	0.00036865
Nup93	orange	1.00659559	5.35E-11	0.78447077	7.09E-07
Rasgrp2	red	-0.6310519	0.00859821	-0.7280718	0.00284567
Sox10	skyblue	-1.5564999	5.40E-07	-1.083703	0.00074131
Polr2f	orange	0.61030766	0.00089221	0.26497875	0.19010798
Klrc3	yellow	2.5946192	0.00165314	0.85600518	0.30469946
C330027C09	yellow	2.63651938	1.01E-25	2.42752881	3.30E-21
Afap1l1	red	-1.1409667	9.70E-10	-0.8365615	1.44E-05
Calhm2	red	-0.5530833	0.00074592	-0.7693616	3.89E-06
Dhrs7c	skyblue	-2.2458543	3.37E-09	-2.4888632	1.34E-10
1700028P14I	skyblue	-2.0049512	5.96E-09	-1.7173285	3.21E-07
Pygb	red	-0.6261453	0.00024334	-0.6880788	7.58E-05
Lmo7	red	-0.5836273	5.07E-06	-0.3694891	0.00588384
Pfkm	skyblue	-1.4165196	3.38E-07	-1.2432438	1.32E-05
Clec1a	red	-1.1228104	8.53E-05	-0.8947424	0.00251129
Tbc1d4	red	-0.3206532	0.14704545	-0.6570519	0.00232903
Triobp	orange	0.68458601	1.06E-06	0.56926909	8.33E-05
Nol12	orange	1.41319839	2.53E-09	1.14638627	2.74E-06
Ybey	red	-0.6128455	0.00406246	-0.8260191	0.00019223
Phldb2	red	-0.6611926	1.65E-07	-0.3096409	0.02166238
Podxl2	skyblue	-1.9333539	2.22E-09	-1.0512846	0.00202925
Dis3	orange	0.66078009	0.00025592	0.54014216	0.00407392

Card10	red	-0.9541043	6.46E-09	-0.7993728	2.10E-06
Mgll	red	-0.9217689	1.60E-13	-1.0726124	8.34E-18
Tie1	red	-1.1431519	6.41E-19	-1.058415	3.39E-16
Lpcat2	orange	1.6343695	1.12E-08	1.322676	7.72E-06
Myh2	skyblue	-3.9414964	0.0070713	-2.9157449	0.05818557
Mamdc2	skyblue	-2.3681919	3.31E-16	-2.0270773	5.77E-12
S100b	skyblue	-2.227575	2.69E-07	-1.8063338	0.00012869
Ttc28	red	-0.9123866	2.80E-05	-0.6564848	0.00377402
AA467197	black	8.22770642	2.14E-29	7.07526833	1.43E-23
Eefsec	orange	0.58826646	2.38E-05	0.25396485	0.09238624
Rac2	yellow	2.84439823	1.62E-34	2.68453612	1.18E-30
Ttf2	orange	1.03570517	7.54E-09	0.90218941	1.01E-06
Trim45	red	-0.9510279	1.27E-06	-0.6645119	0.00129566
Hsf4	red	-1.0218287	5.99E-08	-0.6645164	0.00067477
Duox1	red	-0.8036078	0.06198696	-1.5435488	0.00032775
Stk36	red	-1.1983429	1.54E-05	-0.9848586	0.00052257
Ptprm	red	-1.2402788	8.21E-16	-0.9743127	6.08E-10
Rpgrip1l	red	-1.0297713	4.11E-08	-0.4852709	0.01395711
Noc4l	orange	1.76371387	1.61E-23	1.57473469	8.20E-19
Ptprf	red	-0.8836137	3.62E-05	-0.4201351	0.06804784
Mif	orange	1.03868683	3.49E-12	0.73570596	1.92E-06
Dpyd	red	-0.9182277	0.0006942	-0.8909548	0.00136295
Ctdp1	orange	0.7730023	1.80E-06	0.66109288	7.22E-05
Tnxb	skyblue	-2.3099293	6.12E-23	-1.7148633	7.15E-13
Chst2	red	-0.8287092	1.57E-07	-0.545216	0.0008971
Rtp4	yellow	3.09744982	5.23E-59	2.84429772	7.02E-50
Pus7l	orange	0.96028696	2.56E-05	0.81450794	0.00058755
Palmd	red	-1.1764537	9.29E-07	-0.80896	0.00121382
Frrs1	orange	0.87610711	0.00021474	0.72404714	0.00324877
Arhgap44	red	-0.6238711	0.00063046	-0.3069726	0.12008155
Agl	red	-0.6465133	5.50E-06	-0.4517735	0.00242445
Snap91	skyblue	-2.2955811	0.00204123	-2.1986674	0.00379966
Antxr1	red	-1.2436205	9.11E-12	-0.9764053	1.84E-07
Mcee	red	-1.2658325	6.04E-07	-1.0249446	9.45E-05
Trmt13	orange	0.67891116	0.0025392	0.68115806	0.0028259
Specc1l	red	-0.9697521	7.24E-09	-0.6843306	8.25E-05
Lpar6	orange	0.19505306	0.24875927	0.58517011	0.00032743
Tagap	yellow	2.53998338	1.79E-23	1.99658185	1.04E-14
Adamts15	orange	1.76484952	2.56E-12	1.26899697	1.06E-06
Armcx1	red	-0.865953	1.28E-06	-0.6254915	0.00075482
Crlf2	orange	1.94784624	1.63E-16	1.7471271	2.85E-13
Cysltr2	orange	2.05545666	6.56E-11	1.645854	3.82E-07
Fndc3a	orange	0.60435645	0.00020592	0.55625174	0.00089509

BC026585	red	-0.4720748	0.01349654	-0.6132819	0.00154327
Prss35	skyblue	-1.8001118	2.62E-05	-1.3844251	0.00175348
Cdc14a	red	-0.7686671	2.96E-10	-0.7044917	1.30E-08
Asprv1	red	-0.8492766	0.00746778	-0.6280244	0.05992819
Ppip5k1	red	-0.6914868	2.06E-07	-0.4687339	0.00072153
Acsm1	red	-1.3606664	5.57E-09	-0.0613298	0.83151616
Casp4	yellow	2.66544729	6.43E-32	2.50936731	2.87E-28
Angptl1	skyblue	-2.2431146	3.01E-05	-2.5538275	1.70E-05
Znrf1	orange	0.66860096	7.67E-08	0.82541289	3.65E-11
Dph5	orange	0.98047387	0.00110631	1.22569431	5.91E-05
Rbfox2	red	-0.6298367	9.03E-17	-0.4074429	2.26E-07
Apol6	yellow	3.23047961	2.01E-32	3.43387359	2.53E-36
Myo6	red	-1.0899739	1.37E-06	-0.8692183	0.00019537
Tmem35	skyblue	-2.9369421	1.92E-10	-2.7945623	4.03E-09
Igf2bp2	orange	1.0797884	6.08E-08	0.78328925	0.0001267
Ndn	red	-1.3018856	6.20E-15	-1.1207998	4.16E-11
Myo5c	red	-1.3641824	6.55E-08	-0.7439182	0.00511835
Lgi3	skyblue	-2.2612536	0.00074754	-1.4926382	0.03480823
Rfwd3	orange	0.65322236	1.08E-06	0.39515517	0.00501138
Caskin1	skyblue	-1.0449008	0.00634231	-1.3227383	0.00071414
Pank1	skyblue	-1.9614877	2.86E-11	-1.4673081	1.48E-06
Cplx1	skyblue	-0.9586148	0.13046927	-1.8103522	0.0029951
Map3k13	red	-0.9722082	0.00088427	-0.6977599	0.02149035
Hacd3	red	-0.5279137	3.86E-05	-0.5884113	6.78E-06
Piwil2	yellow	2.81255071	3.05E-07	2.74455803	8.17E-07
Qsox1	orange	0.74216437	6.80E-09	0.43857932	0.00106716
Ucp2	orange	1.3451984	1.46E-23	0.91405648	3.58E-11
Stard9	red	-0.824094	1.20E-05	-0.6328685	0.00118085
Adra2a	skyblue	-2.1268936	4.16E-06	-0.500315	0.2953029
Egr3	orange	1.32137773	2.94E-10	1.16479998	6.41E-08
3300002A11I	skyblue	-2.7345194	7.33E-09	-1.4201091	0.00179464
Recql4	orange	1.92739236	6.50E-15	1.9686942	1.61E-15
Mtss1l	skyblue	-1.7497609	6.69E-18	-1.2082132	7.44E-09
Calm4	red	-1.1052097	0.00640934	-0.5761351	0.19026681
Nrxn2	red	-1.0180315	0.00378717	-1.0426513	0.00380994
Tlr13	yellow	4.11528315	4.16E-20	3.53687879	4.96E-15
Dysf	orange	0.69741644	0.00072501	0.72986335	0.0005499
Atp7a	orange	0.11425381	0.60252149	0.60271026	0.00225214
Fam208b	orange	0.54877224	0.00146145	0.85994906	5.95E-07
Ephx4	skyblue	-2.5843545	1.39E-05	-1.0883511	0.06417172
Pla2g4c	yellow	1.9186633	0.0001503	3.67332888	2.72E-10
B3galt2	skyblue	-3.2639272	2.57E-06	-1.3803141	0.05520079
Fgg	yellow	2.52277777	2.10E-05	2.04644504	0.00061409

Klf9	red	-0.6785916	0.00161655	-0.9514241	1.09E-05
Lgals3bp	yellow	2.74146671	1.95E-27	2.60784771	7.76E-25
Map9	red	-1.1811342	1.26E-08	-0.6329988	0.00294806
Zdhhc15	orange	1.10124189	2.49E-05	0.85166185	0.00159407
Gucy1a3	skyblue	-1.4525622	4.39E-06	-1.167396	0.00035714
Gde1	red	-0.7765379	7.21E-06	-0.5724762	0.00143734
Parl	orange	0.69745578	0.00012156	0.54550603	0.00385057
Rbm34	orange	0.55203761	0.01278652	0.61025727	0.00725959
Zswim5	red	-1.0890047	0.00030202	-0.6688969	0.03457806
Aspm	yellow	2.90160433	1.43E-13	3.37704891	1.68E-16
9430020K01I	red	-0.5890914	5.68E-07	-0.7732649	5.04E-11
Slc16a2	red	-1.1897048	5.33E-18	-1.0175124	2.64E-13
Cdkl4	skyblue	-2.0850843	7.56E-09	-1.1560993	0.00184297
Rfc3	orange	1.61044779	1.66E-17	0.97863627	7.09E-07
Dnah17	orange	1.44516939	8.77E-10	1.37859631	6.13E-09
Fancd2	yellow	2.15623017	1.36E-10	1.80322085	2.34E-07
Cct2	orange	0.67020725	4.41E-09	0.52261342	9.15E-06
Cd226	yellow	2.42297366	5.39E-14	1.70716179	2.75E-07
Ccdc17	skyblue	-1.6071784	3.19E-07	-1.0712226	0.00104037
Fgd5	red	-0.656434	9.90E-07	-0.6300303	4.22E-06
Wbscr17	red	-1.3762756	6.03E-06	-0.4706778	0.16216365
Phka1	red	-0.9573353	6.62E-07	-0.5646976	0.00542364
Farp2	red	-1.2862902	3.83E-05	-0.9669207	0.00260405
Golim4	red	-0.7675073	2.70E-17	-0.5477092	4.30E-09
Kctd7	red	-1.149111	2.56E-12	-0.6743905	9.05E-05
Atp2c2	red	-1.2956242	3.42E-11	-0.6558064	0.00110477
Vav1	yellow	3.25474405	6.33E-46	3.19254797	6.69E-44
Bzrap1	orange	0.61836915	0.00056369	0.33603796	0.07902611
Emid1	red	-1.1567613	2.42E-05	-1.0360391	0.00025288
Faah	red	-0.6023909	2.59E-05	-0.3211313	0.03508142
Zbed5	red	-0.0480676	0.88738247	-0.9387176	0.00127994
Rnf43	red	-1.3668856	1.95E-07	-0.7233431	0.00887716
Lsm3	orange	0.70579904	9.64E-05	0.57036896	0.00256139
Polq	yellow	2.39460698	4.38E-20	2.54832845	1.72E-18
Rasl10a	red	-0.7818193	0.02495469	-0.9567578	0.00666795
Ankmy1	red	-1.3706605	0.00241428	-0.5953486	0.21105521
Rhov	orange	1.62490137	6.12E-05	1.59368993	0.00013506
Foxj1	red	-1.222248	2.63E-07	-0.4151788	0.11065705
Hdac11	skyblue	-1.9164432	1.95E-11	-1.6225303	2.39E-08
Mfsd7c	red	-0.5275349	0.02099675	-0.6725433	0.00350401
Vwa9	orange	0.61644089	2.77E-05	0.4354807	0.00455426
Zdhhc14	skyblue	-1.7450674	3.10E-16	-0.8660464	0.00011115
Batf	yellow	3.23293197	9.88E-32	2.96834104	1.22E-26

Dnajc17	orange	0.83853054	5.36E-06	0.67348485	0.00040283
Traf3ip1	red	-1.1417017	5.38E-12	-0.6623302	0.00012361
Fhod3	skyblue	-1.9992836	5.07E-06	-2.1169592	2.24E-06
Sdr42e1	red	-0.6967447	0.00855809	-0.5305983	0.0564671
Kif4	yellow	2.28061477	8.89E-14	2.93389589	1.17E-20
Iqsec1	red	-0.588065	9.12E-05	-0.5748248	0.00018786
Trim59	orange	1.62623011	1.67E-10	1.62589778	1.07E-09
Brip1	yellow	2.20261054	5.68E-08	2.02830655	1.31E-06
Plcg2	orange	1.4173063	3.35E-08	1.18574116	7.03E-06
Zbed4	orange	0.61245534	0.00033508	0.59588332	0.00066323
Fam151b	red	-0.7230646	0.0024448	-0.6097824	0.01398177
Ube2f	orange	0.58836994	1.13E-07	0.32398591	0.00579221
Smc4	orange	0.89319955	1.71E-07	0.64167318	0.00031414
Ramp1	red	-1.3677228	0.0001075	-0.7183586	0.05782609
Cpne2	orange	1.89901376	1.99E-10	1.72623925	1.40E-08
AI661453	red	-0.7706076	1.23E-05	-0.3745058	0.04715928
Ssu2	skyblue	-3.8332086	0.00031801	-3.066229	0.00080643
Lif	yellow	2.34997227	9.42E-12	3.09934471	1.34E-19
Neurl1b	red	-0.6734756	3.24E-07	-0.6159086	5.94E-06
Pkd1l2	orange	1.88999276	0.00626105	1.99716751	0.00386196
Parp14	yellow	2.34376412	3.47E-143	2.38037313	1.55E-147
Tmem30b	red	-0.7540663	7.64E-05	-0.2046625	0.34120337
Gbp8	yellow	3.66239242	6.08E-63	3.88690214	9.31E-71
Ifit1	yellow	4.0171973	9.82E-107	4.01138642	2.75E-106
Six4	skyblue	-2.3915804	2.30E-08	-1.5834851	0.0003401
Pkd2	red	-0.6556907	4.78E-07	-0.5589991	2.96E-05
Scara3	red	-1.1310401	8.39E-08	-0.3130425	0.17846891
Dynlrb2	skyblue	-1.93857	1.34E-20	-1.3027636	8.28E-10
Rasd2	skyblue	-1.6935971	1.24E-05	-1.0644048	0.00831528
Snx2	orange	0.58120173	1.02E-05	0.45659424	0.00082954
Kdelc2	red	-0.9113445	1.00E-10	-0.7873131	4.93E-08
Edil3	skyblue	-1.8913957	9.50E-12	-1.061856	0.00022498
Pcnxl4	red	-0.7189244	1.23E-09	-0.3637689	0.00327445
Mad2l1bp	orange	0.62377567	0.00017168	0.52862703	0.00221382
Gjc1	red	-0.6217434	3.66E-08	-0.5897548	2.97E-07
Zfp395	red	-1.0466744	2.78E-06	-0.845933	0.00024828
Zfyve9	red	-0.5929784	7.77E-07	-0.5980518	1.04E-06
Inpp5j	orange	1.84327408	7.57E-05	1.33176676	0.00597391
Papd7	orange	0.61638349	1.91E-07	0.56168877	3.43E-06
Exph5	red	-1.0983751	0.00918975	-0.5671997	0.21515782
Hid1	red	-0.7052653	3.71E-05	-0.2900525	0.11961776
Slc41a2	orange	0.66173101	0.00638183	0.55410843	0.02969249
Myo5a	orange	1.03489954	1.85E-09	0.69354668	0.00011534

Ppp1r18	orange	1.61008537	5.18E-16	1.43182427	1.09E-12
Ppm1h	orange	0.60970414	8.50E-05	0.57187799	0.00034581
Setmar	red	-0.3254702	0.18997683	-0.753047	0.00166074
Cd300ld	orange	1.53792007	0.00153329	1.55765884	0.00172833
Ankrd12	red	-0.4994504	0.01329138	-0.5975787	0.00350645
Lrrn1	red	-1.2043715	8.58E-05	-0.8696113	0.01173637
Cd300a	yellow	2.65926006	3.31E-13	2.3437214	2.29E-10
Ythdc2	orange	0.38372108	0.02376042	0.58843523	0.00055017
Cacna1a	red	-1.4432099	3.32E-06	-0.9716885	0.0028956
Tmem109	red	-0.7257082	1.06E-11	-0.6543555	1.73E-09
Bmp2k	orange	0.85233285	4.65E-08	0.77144033	1.43E-06
Itga2b	orange	0.77585204	4.65E-06	0.18505512	0.33893427
Tdg	orange	0.60973077	6.87E-06	0.6737723	1.11E-06
Dbn1	orange	0.67684588	0.05101389	0.97525197	0.00493796
Rnps1	orange	0.76897374	8.92E-06	0.6861092	0.00011884
Fam171a2	skyblue	-1.6851356	1.63E-12	-1.4984213	8.28E-10
Prr7	orange	1.4694751	2.10E-05	1.48792384	4.88E-05
Fras1	skyblue	-1.2582112	0.00012051	-1.6396928	1.68E-06
Dnaic2	skyblue	-1.8259991	3.07E-16	-1.1360274	6.02E-07
Gns	orange	0.8140009	9.91E-05	0.4999459	0.02354956
Grn	orange	1.74171721	1.00E-11	1.37669703	1.60E-07
Adgrb1	orange	1.13245916	0.01884764	1.32962468	0.00566145
Tle6	red	-0.8805994	4.69E-05	-0.9455233	2.11E-05
Map4k5	red	-0.6277734	7.38E-07	-0.3742544	0.0047989
Dusp5	yellow	1.95058904	6.73E-21	2.05539475	7.81E-23
Asb16	red	-1.4455616	2.09E-05	-1.0339725	0.00467293
Tle2	skyblue	-1.6377313	4.00E-08	-1.1321257	0.00025616
BC030867	yellow	3.3576637	1.84E-21	3.61368565	4.69E-20
B3galt1	red	-1.0038166	0.00299828	-0.3847266	0.29745209
Gpsm3	orange	1.44001528	1.71E-11	1.31766772	1.61E-09
Gna15	orange	1.54312303	1.90E-15	1.39399597	2.41E-12
Cpne7	orange	1.3444046	0.00144411	0.68887131	0.13605879
Unc13a	red	-0.988006	8.17E-07	-0.8062804	0.0001016
Scn7a	skyblue	-2.1499921	7.66E-15	-1.8140229	1.19E-10
Celf5	skyblue	-1.0128787	0.09764513	-1.6885541	0.00827518
Nup54	orange	0.68361417	7.64E-06	0.47053508	0.00320683
Tespa1	orange	1.17934581	0.00160615	1.80789599	2.84E-06
Larp6	red	-1.1025255	1.96E-05	-0.2125906	0.48282081
Mfsd12	orange	0.67025101	0.00401449	0.6072424	0.0115224
Cxcl10	black	8.02361014	7.63E-119	8.03714332	1.25E-119
Fam214a	red	-0.7306172	0.00418136	-0.7656364	0.00333181
Gipc3	red	-0.78849	4.78E-07	-0.7829898	1.03E-06
Nudt19	orange	0.65829429	4.56E-06	0.32306858	0.0380084

Tbxa2r	red	-1.3910243	1.16E-05	-0.7335935	0.02993191
Lrr1	yellow	3.16602259	1.89E-08	4.2980019	6.24E-08
Cobll1	red	-0.6225862	0.00271382	-0.5699569	0.00772179
Ncaph	orange	1.79605102	8.72E-15	1.88840206	8.95E-16
Pygo1	skyblue	-1.3915196	5.50E-10	-1.2688237	2.99E-08
Ushbp1	skyblue	-1.7725901	7.86E-16	-1.4446113	1.19E-10
Rtkn	red	-0.9191562	2.50E-08	-0.6322649	0.00020885
Mrpl54	orange	1.01889541	1.43E-08	0.73064069	9.71E-05
Tmem106a	yellow	2.28425501	9.34E-18	2.04611007	2.89E-14
Zfr2	red	-1.1961573	3.07E-10	-0.8147319	2.54E-05
Cebpa	red	-0.6588205	6.40E-09	-0.434397	0.00024129
Atcay	orange	0.49472517	0.18801588	1.04477729	0.00533378
5031414D18	yellow	2.01242413	1.39E-08	2.06529845	1.28E-08
Lbx2	red	-1.0832463	0.0076482	-0.4943964	0.23695094
Dapk3	orange	0.61106251	1.65E-05	0.6057117	3.15E-05
Parm1	red	-0.9712402	2.45E-05	-0.7476572	0.0016512
Hrh2	yellow	3.69871017	5.01E-24	3.96660387	3.33E-27
Dpp4	red	-1.2292395	2.23E-11	-1.0195681	5.92E-08
Igsf6	yellow	2.94704009	5.78E-11	2.73066296	2.24E-09
Baz1a	orange	1.66811739	9.80E-27	1.4238283	1.60E-19
Nek11	skyblue	-2.0455361	2.94E-11	-1.0366111	0.00037936
Ccl5	yellow	3.29421841	8.21E-38	3.20334408	3.41E-35
Kri1	orange	0.66106395	0.0012031	0.63617877	0.00245725
Rrp12	orange	1.13280204	2.27E-08	1.31190061	1.31E-10
Eef2k	red	-0.9298249	3.47E-08	-0.7516368	1.48E-05
Xkr6	red	-0.9754337	1.95E-05	-0.4986242	0.03474257
1700020L24R	skyblue	-1.1871385	0.00080514	-1.2668782	0.00047536
Dcbld2	red	-0.8003774	1.84E-06	-0.5510186	0.00160274
Wnk4	red	-0.6350693	0.00100506	-0.4965815	0.01353065
Wdr78	skyblue	-1.8858652	1.26E-11	-1.1120251	9.88E-05
Gpr33	yellow	3.00819293	0.00376113	1.18797124	0.25193381
Kcne3	red	-1.4784365	0.00316798	-0.6939726	0.19631309
Ppp1r32	red	-1.5332081	4.19E-08	-0.737312	0.00924933
Ubd	black	5.23404096	1.36E-36	5.6865258	3.94E-36
Nkx6-1	red	-1.2351237	0.00220246	-0.6262335	0.16142154
Ano4	skyblue	-2.092211	0.02743899	-3.3964536	0.00142
Slfn8	black	5.03744774	6.55E-30	4.32552851	9.29E-25
Lsm7	orange	0.64670562	2.39E-05	0.30866583	0.06358522
Rims4	skyblue	-2.7346204	0.00515371	-1.7041132	0.06579209
Pdk3	orange	0.5957197	7.78E-07	0.6003876	1.18E-06
Eogt	red	-0.5901089	3.98E-05	-0.4853134	0.00106457
Abi3bp	skyblue	-2.5059799	1.79E-12	-1.6478724	7.62E-06
Pkig	orange	0.61082086	0.00048637	0.34142324	0.06838406

Hpse	orange	1.54289041	1.94E-09	1.30182872	8.29E-07
Tpbg	red	-1.0828875	0.00622263	-0.380594	0.38758988
Raver2	red	-1.0365919	0.00012759	-0.4954243	0.08909016
Plekhj1	orange	0.60796727	0.00015292	0.49186606	0.00328481
Adrb1	skyblue	-1.5494588	1.29E-16	-0.8745703	7.47E-06
Nat14	red	-0.9239141	1.62E-07	-0.8831566	2.04E-06
Sgcg	skyblue	-1.3117527	7.72E-05	-1.3888822	4.60E-05
Mid1	orange	0.93918697	4.33E-07	1.34550251	1.45E-13
Gnptab	orange	0.85008782	1.26E-05	0.64996011	0.00128388
Nup37	orange	0.92251297	2.55E-05	0.81340692	0.00037841
Ccl12	black	5.91632869	2.91E-15	5.44722767	5.53E-13
Kcnh4	orange	2.05701442	0.00011534	1.73286574	0.00160909
Nfkbiz	orange	1.41328322	1.33E-17	1.326793	1.55E-15
Parpbp	yellow	3.86205775	5.45E-13	4.10028015	5.21E-12
Ccl7	black	7.52306965	5.43E-67	7.69567528	1.22E-64
Ccl2	black	7.09275848	3.39E-31	6.80610205	3.80E-29
Dennd1a	orange	0.61759515	0.00013954	0.39561454	0.02053066
Cfap53	red	-0.7819815	0.00239717	-0.310218	0.27758012
Kank4	skyblue	-2.6847471	7.21E-06	-1.6860402	0.00708105
Tmem98	red	-0.616312	0.0001874	-0.6154209	0.000276
Ccr3	black	3.70617384	0.00604517	5.58368807	0.00018125
Foxa1	red	-0.9921894	2.55E-12	-0.3823393	0.01083305
Fignl1	yellow	2.74680273	1.23E-12	3.14930171	2.25E-15
Prdm8	red	-1.5033987	2.20E-08	-0.9578721	0.00062314
Tnni3	skyblue	-1.3049753	0.05651485	-1.9295676	0.00468641
Galm	red	-0.6033775	0.00697519	-0.6077508	0.00825433
Plk5	skyblue	-3.0850237	4.52E-13	-1.9405657	1.65E-06
Tgfbi	yellow	3.73624135	9.23E-22	3.26047923	1.19E-16
Reep6	skyblue	-1.6529534	1.78E-08	-1.1872167	7.77E-05
Cox18	orange	1.13602426	1.35E-10	0.82385675	5.77E-06
Tdrd7	orange	0.85365594	1.03E-10	0.69251417	3.31E-07
Ccdc180	skyblue	-1.9337699	2.40E-19	-0.9906531	6.88E-06
Krt17	yellow	2.08718509	8.57E-08	2.33532586	3.48E-09
Aldh1b1	orange	1.20944695	0.00189309	0.37169301	0.3992404
Iqcg	skyblue	-1.7813452	2.98E-12	-1.1671833	6.76E-06
Ky	skyblue	-3.0022915	1.36E-12	-2.0871111	1.62E-06
Frmpd1	red	-1.4654081	1.96E-06	-1.0751858	0.00066834
Grhpr	red	-0.6729185	3.42E-05	-0.6783828	4.17E-05
Cbarp	red	-0.6153284	0.00275976	-0.3307983	0.1337142
Aamdc	red	-0.805053	0.00012297	-0.8155422	0.00016289
Zswim4	orange	0.47295871	0.00048133	0.62135567	4.75E-06
Sbno2	yellow	2.02200391	3.64E-24	2.0591496	5.70E-25
Tnfsf9	yellow	2.13013639	1.22E-11	1.7761657	4.56E-08

Melk	yellow	2.7227919	7.42E-25	2.37040898	8.96E-19
Thrsp	skyblue	-3.5154841	3.20E-05	-2.7046538	0.00199196
Isg15	black	5.35382118	1.44E-119	5.21394305	3.01E-113
Caps2	red	-1.0498005	0.02951245	-1.2722815	0.00904504
Hmha1	orange	1.17157167	1.33E-31	1.33537077	1.12E-40
Alg8	orange	1.03508881	1.78E-08	0.89858989	1.71E-06
Dok3	yellow	2.30263519	4.28E-13	2.05983413	1.81E-10
Dagla	red	-0.9262005	6.02E-11	-0.6621486	5.79E-06
Bbs10	red	-0.8639612	0.00491901	-0.536032	0.09607135
Krt20	yellow	2.66263816	0.01633908	4.07152039	0.00037353
Cd99l2	red	-0.9337425	2.62E-12	-0.7288543	1.10E-07
Acta2	red	-0.9135144	3.54E-06	-0.1601057	0.48976545
Mlc1	red	-0.9074926	0.00324382	-0.5722223	0.08106271
Plekhs1	yellow	2.90498851	2.16E-05	3.09899186	6.38E-06
Pim3	orange	0.60482825	0.00169656	0.40187313	0.04843531
Ppp1r26	red	-1.0183427	1.49E-07	-0.9548402	1.31E-06
Polr3g	red	-0.4823545	0.05813849	-0.9554467	0.00014422
Ddx11	orange	0.85115441	8.46E-07	0.6995439	9.85E-05
Ids	red	-1.0723326	5.94E-09	-0.7990053	2.70E-05
Misp	yellow	2.5720257	7.10E-34	2.38885437	8.68E-30
Cdhr3	skyblue	-2.7767469	2.16E-16	-1.6213954	3.36E-06
Syt1	skyblue	-2.3746655	0.0017679	-1.7728237	0.02129217
3110052M02	red	-0.8071075	0.00601373	-0.5331031	0.08101599
Zhx3	red	-0.6156973	0.0008615	-0.6730899	0.00035239
Hykk	skyblue	-1.6124293	0.0001033	-1.2945679	0.00250068
Rnf126	orange	0.64467988	6.43E-05	0.57784308	0.00050839
Cerk	orange	0.8140182	6.63E-08	0.91473282	1.73E-09
Gramd4	orange	0.64349255	0.00092726	0.67567576	0.00066824
Dcdc2a	skyblue	-2.4920395	1.23E-07	-1.7294564	0.0003128
Cd276	orange	1.34313915	0.00014156	1.2927967	0.0003607
Bbs9	red	-0.7493154	0.00138167	-0.7163085	0.00284816
Myf6	skyblue	-1.7332153	1.29E-06	-0.7124337	0.08118936
H2-Q4	yellow	2.43344273	1.76E-47	2.24343903	2.17E-40
Chst4	skyblue	-2.0381549	5.39E-06	-2.1154128	4.95E-06
Pknox2	red	-0.8203398	9.91E-05	-0.8622455	5.81E-05
Aldh5a1	red	-1.2499761	1.92E-08	-0.98653	1.43E-05
Ttc38	red	-0.8168544	0.00151012	-0.4825885	0.07909184
Apex1	orange	0.69342509	0.00868917	0.74713209	0.00600527
Tmem59l	red	-1.0019288	0.00024726	-0.7321149	0.00931787
Rusc2	red	-0.7820074	4.10E-11	-0.6481217	9.11E-08
Nme5	skyblue	-1.6919426	5.83E-13	-1.0178991	1.74E-05
Fam65b	orange	1.12821839	2.51E-20	1.29165681	6.45E-26
Tmtc2	red	-0.9519768	2.82E-07	-1.2249034	4.34E-11

Tmem63b	red	-0.6700633	1.15E-06	-0.4271887	0.00301476
Fmnl2	orange	1.1059807	7.47E-20	0.75654262	1.13E-09
Phf24	red	-1.173694	0.00861053	-0.6921915	0.14657881
Slc2a6	yellow	2.72628972	2.33E-17	2.41019718	1.40E-13
Zranb3	orange	0.95305351	0.0022946	0.28224844	0.43087446
Dgkb	skyblue	-2.5591928	6.72E-09	-1.7599138	5.81E-05
Colec12	red	-0.6048597	4.59E-05	-0.471474	0.00219887
Prr5	orange	0.6125033	0.00150512	0.13956481	0.53418626
Mbnl3	orange	1.72371412	1.69E-09	1.42350754	1.41E-06
Meox2	red	-1.4133305	4.71E-21	-0.8144091	1.36E-07
Tm6sf2	red	-0.8747026	0.00942842	-0.6707578	0.05411585
Prickle1	skyblue	-1.7441749	2.45E-18	-1.4317339	1.68E-12
Gatad2a	orange	0.58244635	2.03E-05	0.45211136	0.00139968
Sh3bp4	red	-0.7116458	6.51E-15	-0.4268343	7.36E-06
BC027231	orange	1.01195653	7.31E-06	0.68423617	0.00350998
Ska1	black	3.39677503	7.92E-07	4.91466516	5.73E-10
Agr3	skyblue	-2.0909099	1.64E-06	-0.9661808	0.02366325
3632451O06	skyblue	-1.8612851	3.03E-09	-1.5091985	2.32E-06
Gmip	orange	1.59003534	7.02E-18	1.4237705	2.39E-14
Rbm43	orange	0.68732753	7.73E-07	0.65449934	4.48E-06
Pnpla8	red	-0.6158162	8.64E-05	-0.670358	2.78E-05
Macrod1	red	-1.257115	5.01E-10	-0.9970781	1.88E-06
Gramd1c	red	-0.9564227	2.83E-05	-0.557515	0.01994266
Lrrn3	skyblue	-2.1964698	6.22E-08	-1.6328796	9.13E-05
Slc2a13	red	-0.8147126	0.00056285	-0.8743396	0.00034368
Lzts1	red	-0.604423	0.00100842	-0.3583907	0.07275897
Skp1a	red	-0.6002839	1.56E-06	-0.5540191	1.58E-05
Slc18a1	skyblue	-2.0014865	6.04E-05	-0.2137263	0.708635
Igsf10	skyblue	-2.0813683	1.57E-12	-1.2309564	6.31E-05
P2ry12	orange	1.7153047	0.00012005	0.77795952	0.10288344
P2ry13	yellow	2.2225734	6.39E-09	2.05588648	1.77E-07
Rmdn2	red	-0.8365966	0.00081044	-0.4230151	0.11429364
Serbp1	orange	0.59928532	6.86E-05	0.4210652	0.00745023
C530008M17	skyblue	-0.9159996	0.10737662	-1.8858102	0.0008215
P2ry14	orange	0.89180084	6.27E-05	0.54679635	0.02049127
Glb1l2	skyblue	-1.598222	0.00019582	-1.3668838	0.00199489
Arsi	yellow	0.42507815	0.21739489	2.457075	3.33E-14
Tbcc	orange	0.6637662	0.00035588	0.60139327	0.00178994
Exoc1	orange	0.64834267	4.62E-06	0.64104487	9.24E-06
Npy1r	skyblue	-2.1201299	2.54E-06	-1.9631988	1.75E-05
Lum	skyblue	-2.3914507	2.04E-15	-1.8361741	2.83E-09
Elf1	orange	0.77518299	3.67E-16	0.66705679	4.19E-12
March1	yellow	2.56954614	2.12E-10	2.16771056	1.64E-07

Btg1	orange	1.21434904	4.61E-11	1.13295814	1.52E-09
Eea1	orange	0.58396451	3.59E-08	0.54045998	6.35E-07
Card11	orange	1.84183572	4.12E-10	1.83036029	8.96E-10
Ppfibp2	red	-1.0048893	4.18E-07	-0.5439799	0.00945603
Akap14	skyblue	-2.3642744	1.09E-05	-1.6237063	0.0028889
Lgi4	red	-1.2560001	4.10E-08	-0.5234942	0.03334002
Ndrg4	skyblue	-1.1895361	0.00578156	-1.641974	0.00015678
Gltscr1l	red	-0.6290471	0.00039931	-0.5056519	0.00603018
Fxyd1	skyblue	-1.3097256	9.89E-08	-1.2104902	1.48E-06
Fxyd7	red	-0.8062572	0.00437823	-1.0518081	0.00033132
Fgf1	skyblue	-1.8551878	3.32E-07	-1.3211997	0.0004681
Fut7	yellow	2.77039562	1.69E-11	2.55759157	1.92E-09
H2-Aa	orange	1.34692036	2.26E-19	1.05576594	4.67E-12
Ccdc113	skyblue	-1.6019734	5.15E-13	-1.0516531	3.46E-06
Tssc1	orange	1.00938484	1.91E-12	0.79080344	8.64E-08
Etl4	red	-1.2954229	4.27E-13	-1.0234995	2.30E-08
Alg5	orange	0.79420069	1.81E-05	0.50365811	0.00976916
Clcn7	orange	0.79852604	1.07E-05	0.66368574	0.00039418
Nudt1	orange	0.80015866	0.0009878	0.57801455	0.02435682
Ccdc148	red	-1.1961605	0.00398033	-1.0278911	0.01344824
Tcaf1	red	-0.855644	2.96E-10	-0.7613616	3.89E-08
Cenpt	orange	0.66287514	2.47E-05	0.64789415	5.49E-05
Aaas	orange	0.65453998	9.19E-06	0.51221016	0.00084488
Tmem184a	red	-0.6447642	0.00258592	-0.4156644	0.06548634
Nop14	orange	0.69022794	4.91E-07	0.56717749	6.41E-05
Cyld	orange	0.59880866	1.05E-06	0.6309357	4.50E-07
Dner	skyblue	-1.4436605	0.00231129	-1.5970668	0.00170943
Kif15	yellow	2.84299927	5.97E-37	2.89513573	3.43E-34
Anln	orange	1.21955489	1.85E-07	1.14792327	1.54E-06
Rps27l	orange	0.61343411	0.00058373	0.60435499	0.00098325
Klhl13	skyblue	-1.5338163	3.02E-12	-1.078738	1.82E-06
Amdhd2	orange	0.86984309	9.65E-09	0.63747555	5.04E-05
Lpar3	skyblue	-2.2962	5.03E-11	-1.5221031	2.44E-05
Pnpla7	orange	0.88719897	1.01E-09	0.65383861	1.31E-05
Mcoln3	skyblue	-1.7785463	0.00389519	-1.0011117	0.12295362
Hspb6	skyblue	-1.6477142	3.87E-09	-0.9636075	0.00103056
Wnt4	red	-0.9911104	7.35E-07	-0.9037638	1.23E-05
Dchs1	red	-1.0025299	1.69E-06	-0.6128919	0.00522399
Syde2	red	-1.3783093	1.46E-07	-1.0798	6.06E-05
Proser3	red	-0.8487646	0.00010793	-0.5050538	0.02916031
Smad6	red	-1.3534675	4.21E-15	-1.1290047	1.26E-10
Dna2	orange	1.46924562	1.00E-12	1.17118123	2.98E-08
Phkb	red	-0.8793147	6.16E-10	-0.7968733	4.49E-08

Acaa2	red	-0.7972356	1.72E-05	-0.7630463	5.95E-05
C1qa	yellow	3.66522925	9.95E-21	2.8996783	3.93E-13
Rap2b	orange	1.34002271	6.60E-05	1.23590463	0.0003676
C1qc	yellow	3.36842899	1.70E-18	2.87095967	1.64E-13
Fzd8	red	-0.8534247	5.49E-05	-0.7171909	0.00098305
C1qb	yellow	3.82427099	3.39E-31	3.3151297	1.57E-23
Unc93b1	orange	1.66611034	4.11E-14	1.37741482	9.31E-10
Piwil4	orange	0.84245984	0.00027895	0.73491744	0.00212541
Zfp280c	orange	0.61646618	0.00066061	0.51788663	0.00538987
Stox1	red	-1.3786702	6.71E-07	-0.8900453	0.00158576
Stag3	yellow	2.55385165	0.00011278	2.26601303	0.0006849
Nfkbid	yellow	2.42928262	1.06E-23	2.3582267	2.02E-22
Rab8b	orange	1.26944312	1.00E-11	0.94071252	1.02E-06
Tmem71	orange	0.58774034	6.61E-07	0.48962308	7.15E-05
BC037034	orange	0.83492841	5.67E-07	0.85048434	6.00E-07
Kif1bp	red	-0.5485994	2.97E-06	-0.6305234	1.12E-07
Bcorl1	red	-0.8216842	6.37E-06	-0.6885371	0.00024545
Cfap221	skyblue	-2.0817119	7.40E-11	-1.575284	8.74E-08
Trim17	red	-1.0491223	0.00939378	-0.6147763	0.14731655
Pml	orange	1.85122303	4.33E-39	1.78248265	2.93E-36
Nxt1	orange	0.75851585	0.0002698	0.57486432	0.0086579
Asap3	orange	0.73592398	4.82E-06	0.93653252	6.73E-09
Zfp39	red	-0.8519854	0.00057746	-0.4205131	0.11616105
Tns2	red	-0.8209677	0.00095336	-0.7010271	0.0063878
Zfp113	red	-0.7875021	3.19E-07	-0.3848732	0.01960104
Apln	red	-1.463988	1.78E-07	-0.7985529	0.00693929
Hk1	orange	0.82175116	4.89E-06	0.70476785	0.00014256
Sstr4	red	-0.9823463	0.00125591	-0.9471504	0.00226644
Frem2	skyblue	-2.5471107	8.03E-09	-1.6782562	0.00019783
Wdr62	orange	1.15712634	1.11E-09	0.88583869	5.61E-06
Mmaa	red	-1.0330052	1.06E-07	-0.9068436	5.51E-06
Foxa2	red	-0.8155473	3.31E-07	-0.3014384	0.0829628
Apbb1	red	-1.3045798	2.88E-09	-1.1109867	7.97E-07
Clca3b	yellow	2.1105162	0.01579943	2.83500709	0.00118505
Inhbb	orange	0.96877427	0.01034508	1.27762552	0.00080194
Paip2	red	-0.8355525	1.08E-06	-0.8030773	4.54E-06
Prkcdbp	orange	0.79244818	0.00105443	0.73514948	0.00323349
Rbmxl1	orange	0.85954447	1.07E-05	0.73077113	0.00029981
Scd1	skyblue	-1.7710485	7.71E-06	-1.7128985	2.37E-05
Prr32	skyblue	-2.8116072	0.00496506	-1.8076294	0.08720812
Lrg1	orange	1.54099206	1.74E-21	1.09961886	3.23E-11
Rab11fip3	red	-0.9610535	3.35E-10	-0.6804309	1.76E-05
Ttc29	skyblue	-3.4376679	3.09E-11	-2.0105624	0.0001332

Dcaf15	orange	1.41613628	1.00E-28	1.22160089	2.03E-21
Psd	orange	0.76413976	1.66E-05	0.62085564	0.00076498
Tmprss13	red	-1.1331543	0.00123982	-0.7777658	0.03485583
Myom3	skyblue	-2.5639988	1.31E-05	-1.2865381	0.04084059
Cfap61	skyblue	-2.1456321	5.90E-06	-1.6184786	0.0008011
Lrrc20	red	-0.7213927	4.54E-10	-0.5831827	1.35E-06
Ppp1r14a	red	-0.6838807	0.00027028	-0.5230879	0.00747818
Mycn	skyblue	-1.7318358	1.82E-07	-1.1038176	0.00154658
Cyb561d2	orange	0.68580131	4.03E-07	0.50146877	0.0004528
Pacrg	skyblue	-1.3813824	6.61E-05	-1.2552141	0.0004094
Prf1	yellow	4.13059328	4.76E-38	4.12231929	1.12E-37
Islr	red	-1.0459942	9.80E-15	-0.5250764	0.00021573
Spry1	red	-0.7272354	0.00101617	-0.321078	0.18512681
Spred3	orange	0.93977319	0.00012977	0.91650278	0.00027264
Clic4	orange	0.77782812	1.02E-06	0.65361841	6.66E-05
Itih2	red	-1.2767254	3.33E-06	-0.5336046	0.06615458
Dzank1	skyblue	-2.3066946	6.41E-14	-1.1453598	0.00032701
Galnt6	yellow	2.16447188	9.81E-18	1.94813982	2.20E-14
Tbcel	red	-0.785204	5.16E-05	-0.560188	0.00565993
Tacc3	yellow	2.76726554	2.29E-41	2.52248336	8.04E-34
Traf3ip3	yellow	1.81260125	7.42E-38	2.24921935	1.26E-53
Tap1	yellow	3.34477665	1.16E-104	3.15172065	5.61E-93
Bbs7	red	-1.0792017	4.31E-11	-0.5818143	0.00057484
Larp1	orange	0.87006454	8.28E-12	0.72763879	2.31E-08
Map4k1	yellow	2.23395206	8.61E-32	2.2198963	5.21E-31
Slc12a9	orange	0.91182162	1.25E-09	0.79969421	2.02E-07
Chst7	red	-1.1407885	0.00015162	-0.9392147	0.0025685
Paqr7	red	-1.0409965	3.04E-06	-0.864894	0.00017154
4930578C19	red	-1.2346576	4.57E-11	-0.721782	0.00032919
Sf3b6	orange	0.630137	1.58E-06	0.48712491	0.00038115
Nov	skyblue	-1.6106253	2.57E-08	-0.7174652	0.01874194
Letm2	red	-0.7222899	4.12E-05	-0.6841846	0.00016538
Pafah2	red	-0.9686249	3.89E-07	-0.6922224	0.00047456
Trmt6	orange	1.239832	2.46E-10	0.82418489	5.12E-05
Icam1	orange	0.87197771	1.33E-06	0.931227	3.63E-07
Serpine1	yellow	1.99737419	2.02E-09	2.00414388	2.73E-09
Fer1l5	red	-1.0595519	1.51E-06	-0.9714154	1.82E-05
Vnn1	red	-0.9667847	0.00379923	-0.2905842	0.44335274
Cep85	orange	2.01234948	1.82E-25	1.33270141	1.81E-11
Arid5a	yellow	2.35397328	3.76E-27	2.50127795	1.97E-30
Slc18b1	red	-1.0793481	1.15E-11	-0.7708359	2.31E-06
4930427A07I	yellow	2.19052518	2.31E-22	2.26760627	3.45E-23
Papl	skyblue	-1.7036934	0.00521443	-0.8919812	0.16557242

Dtl	yellow	2.54143247	5.90E-14	2.80021624	1.30E-15
Slc2a12	skyblue	-2.0708465	9.50E-12	-1.288071	3.11E-05
Zmat4	skyblue	-2.6624315	1.11E-05	-1.5409072	0.01634503
Cib2	skyblue	-1.638215	2.74E-10	-1.0228141	0.0001605
Arhgef4	red	-0.7260993	0.0092285	-0.2515919	0.41249627
Mavs	red	-0.5855781	9.69E-06	-0.4873838	0.00036796
Atg14	red	-0.6600044	0.00177008	-0.4178327	0.06183987
Rapgef6	orange	0.60231335	7.30E-06	0.47920448	0.00057349
Shank2	skyblue	-2.3005242	3.63E-19	-0.9905822	0.00015025
Dlgap5	yellow	3.67737046	2.68E-51	4.14544062	1.38E-55
Rps16	orange	0.59400704	4.20E-05	0.41153524	0.00662766
Vash2	skyblue	-1.83205	1.24E-05	-1.6924323	0.00013072
Wdhd1	yellow	2.30384678	1.19E-25	1.79117484	1.29E-15
Tob1	red	-1.2472463	1.97E-10	-0.9278888	4.30E-06
Kcnh3	red	-1.2864647	8.86E-05	-0.5966182	0.08636439
Gch1	orange	1.4372693	9.04E-11	1.22385928	6.39E-08
BC022687	red	-0.797044	0.00019125	-0.8120091	0.00027144
Nme1	orange	1.27689791	4.96E-09	0.79912234	0.00051166
Osbpl5	red	-0.7158592	1.25E-05	-0.5067892	0.00297024
Kcnmb2	skyblue	-2.5184036	2.25E-11	-1.8563864	8.14E-07
Tnfrsf23	orange	1.37758855	0.00058165	1.32516696	0.00131096
Wdtc1	red	-0.6586282	5.29E-08	-0.6089203	8.64E-07
Kcnk2	skyblue	-3.0898259	4.88E-13	-1.8004437	4.68E-05
Cldn11	skyblue	-3.4253522	0.00070044	-2.6758615	0.00291295
Rgs22	skyblue	-2.255154	3.33E-09	-1.5427554	6.09E-05
Cdkn3	yellow	3.27269514	7.06E-21	3.24298809	1.98E-17
Slc25a43	orange	1.23943632	7.66E-05	0.93539118	0.00502783
Zfp60	red	-0.9453937	5.52E-07	-0.6222428	0.00165476
Prkci	red	-0.7225033	1.16E-10	-0.4517056	0.0001122
H2-DMa	orange	1.60404384	1.04E-13	1.31644668	2.45E-09
Kctd8	skyblue	-2.7505287	1.67E-07	-1.6261203	0.00165054
Gpr160	orange	0.91858752	2.82E-07	1.18282751	1.31E-10
Cdkn1c	red	-0.7661179	0.00141908	-1.0064333	3.23E-05
Esyt3	red	-1.2317732	0.00012391	-0.9101924	0.00617297
Armc3	skyblue	-1.8309447	9.04E-14	-1.5860325	6.77E-11
Cd81	red	-0.6502518	3.10E-06	-0.4463356	0.00214478
Fam13a	skyblue	-2.2156065	6.64E-08	-1.6956572	6.13E-05
Cisd1	red	-0.5917894	0.00015161	-0.4919571	0.00249923
Ccdc33	skyblue	-2.1095311	2.77E-11	-1.183459	0.00015741
Ckap2	yellow	3.35923397	8.22E-25	2.75867987	3.27E-17
Themis2	yellow	3.09792854	2.52E-24	3.34349549	4.97E-28
Limch1	red	-1.0983776	3.96E-05	-0.855757	0.00201122
Nek5	red	-1.3503632	9.93E-09	-0.8070801	0.00084848

Ptger2	orange	1.03720081	0.00034776	0.80486546	0.00798054
Slc16a9	red	-1.1052962	0.00047346	-0.9629747	0.00315308
Dzip1l	red	-0.7960076	1.40E-06	-0.4626527	0.00728115
Iqch	skyblue	-3.3042394	0.00023638	-1.8421217	0.03178823
Rpl10a	orange	0.63617257	3.25E-07	0.5086241	7.66E-05
D630003M21	skyblue	-1.35057	1.25E-05	-1.4784489	4.59E-06
Fbxw17	orange	1.36931284	3.75E-19	1.22892888	1.52E-15
Tgm2	orange	1.80527087	4.44E-14	1.73075255	7.48E-13
Smim14	red	-0.6249173	1.71E-05	-0.6183564	3.19E-05
Rtkn2	skyblue	-2.7963074	0.00087201	-2.1442828	0.01436256
Nmrk1	orange	0.62010058	0.00020972	0.48288689	0.00718103
LOC1026395	yellow	4.23219923	8.50E-44	3.9956066	5.40E-39
Cpe	skyblue	-1.3467285	5.29E-15	-1.1931359	9.13E-12
Zfp365	red	-1.2240121	6.02E-13	-0.8479149	1.51E-06
Aim2	yellow	2.82701324	1.48E-29	2.70208724	2.30E-26
Egr2	yellow	2.4330051	0.00152843	2.7820941	0.00055612
Ackr1	yellow	2.2499875	5.58E-10	2.44048824	3.65E-10
Wdr19	red	-0.8422298	3.16E-05	-0.4283423	0.04648501
Pcdh18	red	-0.9305976	0.00019422	-0.7462483	0.00389022
H2afz	orange	1.11349967	1.28E-09	1.03607511	2.97E-08
Sirpa	orange	1.1032679	2.32E-05	0.95903689	0.00035917
Bri3bp	orange	0.93456998	9.19E-10	0.83565553	1.14E-07
Tmem156	orange	1.87671589	2.05E-07	1.72471759	3.31E-06
Ddx60	yellow	2.6702349	1.15E-33	2.71954418	1.12E-34
Inpp4b	orange	1.1581915	2.30E-06	0.63794803	0.01429936
Ccr7	orange	1.80778357	7.42E-22	1.8438003	2.95E-22
Fgd3	yellow	2.08392038	9.55E-11	2.15241141	3.71E-11
Ccdc55	orange	0.7136818	0.00031148	0.40739054	0.05499579
1110007C09	orange	0.62091269	0.00349664	0.57697505	0.00852762
Fam101a	red	-1.2896858	3.20E-05	-0.8797986	0.00673619
Zc3h7a	orange	0.92983384	1.36E-23	0.88611353	2.42E-21
Ninj1	orange	1.12261117	1.49E-06	0.9471919	8.12E-05
1110032A03I	red	-0.8582973	6.05E-09	-0.6897796	5.17E-06
Ccdc129	skyblue	-2.8615344	3.46E-07	-1.835969	0.00130927
Ccdc92	red	-0.8501806	4.12E-06	-0.7919181	2.83E-05
Wnk2	red	-0.9907036	0.0039972	-1.103705	0.00167956
Rmi2	yellow	2.2150252	0.00159835	4.14054804	5.48E-08
Slc24a2	skyblue	-1.879941	0.00520399	-1.7557078	0.01375874
Parp11	orange	1.81363	1.58E-42	1.8208729	1.51E-42
2700029M09	orange	1.01651068	1.52E-08	0.5906073	0.00179153
Acer2	red	-0.643236	0.00805386	-0.7879319	0.00133636
Dnah10	skyblue	-1.7729769	8.37E-08	-1.1716948	0.00063236
Fam188b	red	-0.7203649	0.000108	-0.7368779	9.05E-05

Socs1	black	4.6214377	4.27E-82	5.26110486	2.58E-98
Ptpdc1	red	-1.2393881	7.13E-18	-0.7094132	1.60E-06
Sult6b1	yellow	2.11895851	0.0033239	2.19328681	0.00977605
Haus6	orange	0.67426892	3.15E-07	0.55749652	4.49E-05
Nod1	orange	0.62285812	1.03E-18	0.65917929	9.02E-21
Smim3	orange	0.97979706	9.44E-05	0.78638944	0.0025408
Dlec1	skyblue	-2.5144649	6.58E-10	-1.1014013	0.00921886
Mturn	skyblue	-2.2432185	1.39E-07	-1.942826	8.66E-06
Csf3	yellow	3.99739971	6.53E-07	3.48345594	4.79E-07
Cntln	red	-0.8382158	9.81E-07	-0.5279117	0.0029946
Kcna6	skyblue	-2.9975408	1.25E-12	-2.0543756	3.58E-07
Cnbd2	orange	1.42620056	1.14E-09	1.08153678	1.44E-05
Atp13a4	skyblue	-1.3327946	0.00049836	-1.3635178	0.00059589
Ano2	skyblue	-0.5627212	0.50523529	-2.411775	0.00926687
Cdon	red	-0.7681229	2.93E-06	-0.5585951	0.00111086
Fam210a	red	-0.6768376	1.36E-09	-0.6235223	6.30E-08
Tbc1d32	red	-1.1325466	8.63E-07	-0.8335397	0.00043744
Camk4	yellow	1.76948623	8.95E-06	2.6312618	7.47E-11
Rbm24	skyblue	-1.2824257	1.07E-05	-1.391357	2.79E-06
Stox2	red	-0.7647565	1.01E-05	-0.7038561	7.71E-05
Notch3	skyblue	-1.3831464	8.54E-08	-1.1615701	1.21E-05
Cd84	yellow	2.440806	2.22E-22	2.15852619	1.70E-17
Ormdl3	red	-0.7465681	2.94E-09	-0.5819463	7.74E-06
Gstp2	skyblue	-1.4894296	5.00E-07	-1.2577679	4.15E-05
P3h2	red	-1.518693	6.92E-07	-0.9638767	0.00240604
Ttc39b	orange	2.05204344	1.43E-23	1.82680281	1.01E-18
Enpp6	skyblue	-0.5472098	0.47577982	-2.9701775	3.88E-05
Fam126b	red	-0.6657079	0.00051516	-0.4488777	0.02590412
Slamf7	yellow	3.35296406	6.48E-21	2.97168541	2.22E-16
Chpf2	orange	0.78602278	1.67E-07	0.74076328	1.32E-06
Rilp	red	-1.0395174	1.14E-05	-0.6806368	0.00608691
Kcna7	skyblue	-1.8779519	1.20E-05	-0.7137563	0.12971707
Asb10	skyblue	-1.7082659	9.72E-06	-2.0488575	2.98E-07
Tapbpl	orange	1.63282148	1.37E-38	1.64336891	6.11E-39
Tlcd2	red	-0.5513074	0.02967973	-0.707576	0.00685817
Fam198a	skyblue	-1.7384266	2.74E-09	-0.8341175	0.00626537
Hrc	skyblue	-1.8914027	1.79E-10	-1.9564216	7.55E-11
Sobp	red	-1.1259869	0.00142709	-0.691612	0.06836149
Ncapd2	yellow	2.02839765	4.55E-28	2.18158804	6.53E-32
Hoxa5	red	-1.1124539	4.58E-09	-0.9616792	7.63E-07
Trpm4	red	-1.1482231	1.35E-10	-0.7979928	1.61E-05
Sema7a	orange	2.0933365	8.39E-25	1.37710715	4.60E-11
Slc22a23	red	-0.9962604	2.44E-06	-0.6073933	0.00619218

Fau	orange	0.85128812	8.89E-09	0.53607818	0.00053118
Nop2	orange	0.85983035	2.04E-07	0.75272822	9.44E-06
Ostm1	orange	0.79517457	2.46E-05	0.63419064	0.00114737
Bphl	skyblue	-1.5727387	1.35E-18	-1.1133876	1.12E-09
Snx25	red	-1.1767633	4.38E-06	-0.8984425	0.0007214
Ccdc155	skyblue	-2.3495727	6.55E-06	-1.241532	0.02123631
Snx10	yellow	2.27331588	3.48E-18	2.0014559	3.37E-14
Lace1	red	-1.2572653	5.02E-10	-1.1417689	2.96E-08
Cd160	yellow	3.43252339	3.89E-11	2.82867713	7.98E-08
Kcnh2	skyblue	-1.397774	9.17E-05	-1.4761292	5.02E-05
Tsr1	orange	0.61411085	0.00156387	0.80683011	3.98E-05
Txlng	red	-0.6951584	0.00110156	-0.5600507	0.01154568
Tcte2	red	-0.8575562	0.0066359	-0.6097636	0.06698444
Plcl1	red	-0.8629215	3.51E-05	-0.8040935	0.00017476
Arl5c	orange	1.14752467	0.00378425	1.59454014	6.66E-05
Lasp1	orange	0.69586094	0.00056301	0.73780657	0.00034533
Pcp4l1	skyblue	-2.5823158	1.04E-19	-2.2331806	6.95E-15
Gmds	orange	0.84455282	1.07E-07	0.57706434	0.00047917
Trp53inp2	red	-1.2042169	1.05E-12	-0.9726299	1.92E-08
Ttk	yellow	2.56778831	3.83E-14	2.9281863	1.67E-15
Mpp6	orange	0.71209047	0.00198822	0.58854481	0.01422301
Pmepa1	orange	0.83055003	0.00036811	0.7416644	0.00204486
Foxf2	red	-1.3352865	4.53E-15	-0.9164346	1.85E-07
Hfe2	skyblue	-1.591042	2.81E-07	-1.3450908	2.74E-05
Egr1	orange	1.13576583	4.07E-06	0.88505558	0.00051175
Srcin1	red	-1.3428805	3.38E-07	-0.6222993	0.02604227
Dennd2a	red	-1.1365971	7.44E-16	-0.95348	2.63E-11
Tmem255b	skyblue	-1.5115372	0.0002135	-1.9865425	4.33E-07
Chmp4b	orange	1.360056	5.96E-17	1.27405385	7.90E-15
Cdk19	red	-0.3919842	0.01991003	-0.6050554	0.0003278
Tfdp1	orange	0.57243755	3.52E-05	0.59178565	2.89E-05
Tmco3	red	-0.7482433	2.13E-09	-0.6564523	2.73E-07
Ptov1	red	-0.7225363	4.13E-08	-0.5685197	2.89E-05
Dcun1d2	red	-0.7699883	6.22E-06	-0.7104697	5.01E-05
Parp12	orange	1.78785564	1.80E-42	1.8292908	2.14E-44
Gdf15	yellow	3.29040865	4.92E-11	3.36872524	2.86E-11
Rpf2	orange	1.18551613	2.09E-07	1.07303436	5.12E-06
Grtp1	red	-1.0082839	0.00480873	-0.5174763	0.18068813
AI317395	red	-1.1110918	0.00033143	-0.6962929	0.03370497
Car14	skyblue	-1.4333014	5.61E-07	-1.6598783	9.68E-09
Rgs4	red	-0.791608	4.94E-05	-0.7307159	0.00027668
Atf5	red	-0.6619139	1.60E-05	-0.4373068	0.00650243
BC028528	red	-1.2201994	3.01E-07	-1.0731428	1.27E-05

Ciart	skyblue	-1.4640894	4.33E-08	-1.26233	3.93E-06
Ift172	red	-0.934568	1.15E-10	-0.7852855	1.20E-07
Saxo2	red	-1.5004818	2.97E-07	-0.999846	0.00085415
Susd4	red	-0.8856559	1.30E-07	-0.2803244	0.12788774
Susd1	orange	0.76832328	0.00281885	0.74997858	0.00387934
Pln	skyblue	-1.5219389	0.13619182	-3.0916386	0.00204202
Akap12	orange	0.74198097	0.03011862	0.97938659	0.00444563
Colec10	skyblue	-6.3500378	3.46E-05	-4.0103658	0.00614707
Slc35f1	skyblue	-2.5745978	0.00112293	-1.9112663	0.02426082
Dock10	orange	1.26258663	5.36E-11	1.08135723	4.05E-08
Mcl1	orange	0.59365346	1.83E-06	0.50346076	8.60E-05
Rassf7	red	-0.8195917	1.20E-05	-0.4499819	0.02346764
Med30	orange	0.40083458	0.0521969	0.62486402	0.00261047
Tm6sf1	orange	0.74202667	0.00097993	0.84942019	0.00021159
Lrrc56	red	-0.9720464	1.08E-07	-0.6731531	0.00032743
Ctss	yellow	3.82191139	1.08E-27	3.32726763	4.44E-21
Creb3l2	red	-0.6861085	2.05E-08	-0.53493	2.26E-05
Rnh1	orange	0.86274857	1.50E-08	0.75909509	1.13E-06
Fsd2	skyblue	-1.5077658	0.00054146	-1.8921858	2.07E-05
Mybpc2	red	-0.9302816	0.00173411	-0.4426101	0.1713231
Scube3	red	-1.5446307	6.73E-16	-0.5911456	0.00231773
Trps1	orange	0.67199344	2.34E-05	0.43827468	0.00895643
Hoxb4	red	-0.5931554	6.28E-08	-0.4279368	0.00016208
Taf5l	orange	0.64162945	0.00023606	0.53178508	0.00321091
Hoxb5	red	-0.8314081	0.00016964	-0.6719315	0.00332939
Dsel	red	-0.7844374	0.0001096	-0.4948283	0.0204091
Bud31	orange	0.68339799	2.68E-05	0.45126718	0.00848437
Pkhd1l1	red	-1.190005	0.00070049	-1.1924171	0.00097439
Mvb12b	red	-0.8032425	3.20E-10	-0.4207884	0.00168207
Ttll6	skyblue	-1.6016989	3.81E-05	-0.8580164	0.036435
Nup205	orange	0.87682011	1.44E-08	0.79700265	4.66E-07
Alpk3	skyblue	-1.4698231	1.66E-07	-1.2769602	1.07E-05
Ptpn3	red	-0.9423821	2.48E-08	-0.6074859	0.00059262
Ascc3	orange	0.83753323	9.92E-10	0.93996349	7.69E-12
Vill	orange	0.87370045	2.66E-05	0.69147537	0.00131164
Ephx1	skyblue	-2.3576591	1.22E-10	-1.8349212	1.11E-06
Sema6c	skyblue	-2.2900177	1.80E-09	-1.3747572	0.00052464
Scgb3a2	skyblue	-2.9590729	1.05E-14	-2.0431108	2.31E-07
Lefty1	red	-0.9550447	0.00415584	-1.3081311	0.00011264
Zscan2	red	-0.7029819	4.17E-05	-0.3524198	0.06239963
Rap1gap2	orange	1.16315206	5.99E-05	0.84462277	0.00518513
Gngt2	orange	1.11121074	1.21E-07	1.0553153	9.39E-07
Ctnnal1	red	-0.8268477	0.00010605	-0.6301513	0.00454639

Agbl3	red	-0.6935455	0.00405237	-0.6786087	0.00594386
Gcnt1	orange	1.7173562	2.40E-09	1.75271248	2.02E-09
Baiap2l1	red	-0.7905202	2.36E-05	-0.5473887	0.00498189
Zcchc2	orange	1.22474516	1.53E-16	0.94208482	5.15E-10
Bpgm	red	-0.7075383	0.00088325	-0.7304711	0.00080595
Nipal2	red	-0.7755304	2.71E-07	-0.5616597	0.00030106
Mrps34	orange	0.38770625	0.11698487	0.73353449	0.00262235
A230050P20I	orange	0.52092104	0.00013938	0.62716227	5.70E-06
Fam117a	red	-0.6684225	8.47E-05	-0.4388053	0.01446123
Rpl12	orange	0.80524236	9.05E-09	0.67612294	2.60E-06
Plcl2	orange	1.52509062	6.06E-14	1.49567507	3.94E-13
Sccpdh	red	-0.5652089	5.65E-06	-0.6617598	2.06E-07
Prc1	yellow	2.4488486	8.93E-21	2.34493119	6.87E-19
Cnst	red	-0.6715053	0.00039106	-0.5856843	0.00270157
Slco4a1	yellow	1.71375215	7.76E-09	2.27478638	4.74E-14
Pdk2	skyblue	-1.5487013	2.33E-05	-1.4095311	0.00018073
Cldnd2	yellow	2.66009973	6.78E-05	2.26977994	0.00158395
Rabggtb	orange	0.58972496	7.92E-07	0.56410869	4.22E-06
Rbbp8nl	skyblue	-0.8977459	0.22337414	-2.0716483	0.00365966
Bloc1s5	red	-0.6596953	3.59E-05	-0.4093324	0.0159898
Tspyl5	skyblue	-1.2307477	0.00112664	-1.7105958	1.17E-05
1700019L03F	red	-1.41598	2.25E-11	-0.6032548	0.00617734
Bmp6	skyblue	-1.6185499	2.38E-12	-1.3307521	1.74E-08
Tlr4	orange	0.71245586	1.06E-05	0.83302928	3.41E-07
Ttc16	red	-1.4779157	3.35E-10	-0.9629933	7.21E-05
Adrm1	orange	0.82617079	4.10E-09	0.67703266	2.76E-06
Eme1	yellow	3.04773381	3.60E-16	3.69612447	1.01E-18
Myo16	skyblue	-2.149621	8.04E-05	-1.8241308	0.00098373
Ak5	red	-1.4511241	0.00214632	-0.8896676	0.08359975
Echdc3	skyblue	-1.5655813	1.41E-06	-0.8117744	0.01665082
Zfp503	red	-0.8943979	1.30E-05	-0.4287571	0.05154671
Chad	skyblue	-2.7180332	3.07E-10	-1.8789406	2.46E-05
Sptlc3	red	-1.1546623	0.00058227	-1.2701862	0.00024477
Wdr93	skyblue	-2.2513339	2.88E-09	-1.3490811	0.00035605
Nexn	red	-0.6438149	0.00067915	-0.6119603	0.00180634
F13a1	orange	0.61606653	0.032723	0.82449017	0.00447075
Mycbpap	skyblue	-2.162218	5.43E-13	-1.4469764	2.47E-06
Nrn1	skyblue	-1.9006694	3.97E-10	-1.1334743	0.00030929
Adgrg6	red	-1.1706	6.33E-14	-1.0052788	2.55E-10
Gipc2	red	-0.5000935	0.00361232	-0.6029234	0.00057655
Whrn	red	-0.7311535	0.00317547	-0.3077215	0.25321974
Camk1d	orange	0.60005163	0.00329463	0.59700639	0.00464828
Runx2	yellow	2.76247613	1.21E-21	2.43455546	6.44E-17

Cdh26	skyblue	-4.3567068	3.30E-20	-4.5016055	1.40E-18
Akna	orange	1.43407899	3.52E-14	1.18251431	9.04E-10
Akap7	red	-0.9507835	7.75E-10	-0.6258598	9.32E-05
Tbc1d19	red	-0.8659712	4.10E-13	-0.4935683	7.83E-05
AW209491	red	-0.6822883	0.00181527	-0.635838	0.00480865
Nubp2	orange	1.08135392	6.04E-09	0.96729411	3.60E-07
Fanci	yellow	3.09288651	1.57E-11	4.35404613	1.29E-17
Rbpj	orange	0.59063975	0.00028247	0.45766326	0.00691874
Nlrc4	orange	1.52605726	6.86E-05	1.31220896	0.00107914
1110008P14I	orange	0.69444237	0.00016713	0.49091906	0.01146246
Orm1	orange	1.30770836	0.0098192	0.17422292	0.77633674
Adk	red	-0.8587018	2.74E-12	-0.7218467	9.11E-09
Zdhhc1	red	-1.2063011	5.59E-10	-0.79649	8.04E-05
Metrnl	orange	1.34276677	1.92E-06	1.52907214	9.19E-08
Suv39h1	orange	0.96250655	2.28E-08	0.77007601	1.55E-05
Stx11	orange	1.63638575	7.72E-12	1.68710206	2.57E-12
Isg20	yellow	2.213804	1.65E-29	1.91513257	2.89E-22
Lyplal1	red	-1.2379516	0.00430892	-0.6631935	0.14835159
Lgi2	red	-0.9590069	6.78E-08	-0.4919729	0.00878117
Fn3krp	red	-0.7473123	0.00223939	-0.6717719	0.00794639
Pomt1	red	-0.6721857	4.55E-07	-0.4369513	0.00149583
Vstm2b	skyblue	-3.0949337	2.70E-08	-2.1765181	6.15E-05
Gimap3	yellow	2.15545884	1.03E-23	2.21261371	9.72E-25
Megf9	red	-0.8132511	0.00014567	-0.6560722	0.00310386
Pcsk1n	skyblue	-1.7504649	0.00122415	-0.8174669	0.1681277
Azi2	orange	0.76767239	1.20E-13	0.59613904	1.90E-08
BC017643	orange	0.82973308	3.73E-11	0.66449807	2.40E-07
Ndst2	orange	0.63470293	1.37E-05	0.62332408	2.86E-05
Clnk	orange	1.39613209	0.0167842	2.00371428	0.00385095
Rftn1	orange	1.00436388	1.98E-12	0.96301935	2.94E-11
Rab3gap2	orange	0.48885528	0.00308336	0.62275791	0.00019552
Atp6v0e2	red	-1.0872661	1.19E-06	-0.8403698	0.00028481
C130074G19	red	-0.9975963	2.42E-20	-0.7941989	5.21E-13
Exosc2	orange	0.69102273	2.14E-06	0.45513042	0.0029769
Picalm	orange	1.06929163	2.59E-09	0.90160582	9.71E-07
Sectm1b	black	4.12369642	2.63E-09	5.20976747	5.43E-14
March4	skyblue	-2.3678359	2.12E-18	-1.1659329	8.20E-06
Synpo2l	skyblue	-1.5148469	0.00027502	-1.10986	0.0107862
Wdr45	red	-0.7879073	0.00011469	-0.7628494	0.00027508
Dusp10	orange	0.88184824	3.28E-07	0.96390491	5.91E-08
Cdh6	red	-1.2976437	3.28E-06	-0.8864512	0.00212121
Ccdc81	skyblue	-2.2921709	1.19E-12	-1.6225528	3.65E-07
Neil3	yellow	3.20261026	4.55E-18	4.31746082	1.38E-24

Prdm16	red	-1.2090519	1.59E-15	-0.6373138	5.29E-05
Dcxr	red	-1.1554521	0.00011414	-0.7627683	0.01520513
Snx22	red	-1.1462846	2.47E-05	-0.502023	0.08730022
Morc3	orange	1.10643713	3.80E-11	0.89086414	2.16E-07
Col10a1	skyblue	-2.6115049	0.01863648	-3.8842553	0.00104912
Prrx2	orange	0.41627638	0.50319832	1.88889419	0.00172041
Micu3	red	-0.4374371	0.02077554	-0.6165349	0.00119956
Tspyl4	red	-1.1430728	5.34E-06	-0.7516367	0.00413651
Cdnf	red	-1.3452139	2.35E-05	-1.0570193	0.00173958
Znfx1	orange	1.57771329	3.03E-30	1.59890208	5.47E-31
Cyp7b1	yellow	2.1517242	5.50E-11	1.94285301	7.42E-09
Foxp3	yellow	3.914145	2.18E-23	3.54594339	9.41E-21
Atp8b1	orange	0.75160321	3.81E-05	0.55353042	0.00360689
Zufsp	orange	1.45381349	2.16E-40	0.98584797	1.12E-18
Ncam1	red	-0.7675069	7.76E-06	-0.8447815	4.04E-06
Cfap70	skyblue	-2.4074148	8.26E-16	-1.3850864	3.82E-06
Ajap1	red	-0.9060587	0.03806461	-1.1564309	0.00844907
Rsph4a	skyblue	-2.7214859	1.32E-19	-1.4528827	1.19E-06
Ccser1	red	-0.9706898	2.63E-06	-0.482664	0.02490086
Rcan2	skyblue	-1.5571066	0.00028547	-1.5865931	0.00031026
Rbms3	red	-1.0878459	8.08E-12	-0.9130983	2.07E-08
Tmem246	red	-1.0776516	1.10E-09	-0.8144563	8.04E-06
6430573F11I	red	-1.3744863	1.14E-05	-0.862346	0.00661722
Prex1	orange	1.79870457	2.73E-17	1.66536908	8.67E-15
Hs3st6	red	-1.4410537	0.00056667	-1.0604865	0.01512422
Hnrnpu	orange	0.5818189	8.17E-05	0.54856112	0.00030322
Ccdc151	red	-1.3417859	4.41E-10	-0.9214497	2.91E-05
Lonrf1	red	-0.7897959	1.38E-06	-0.907211	5.12E-08
Zfp189	red	-0.7043777	0.00607258	-0.4248762	0.12170264
Coro7	orange	1.16951657	5.87E-15	1.01122775	3.21E-11
Ccbl1	red	-1.0765192	1.45E-05	-0.8140329	0.00155127
Cpeb3	red	-0.776636	1.25E-05	-0.6709926	0.00027264
Dusp26	skyblue	-1.952643	0.00018152	-1.7308224	0.00089966
Kcne2	skyblue	-2.0574264	3.40E-06	-1.0138253	0.03044067
Capsl	skyblue	-1.6007931	2.10E-09	-1.0140941	0.00024731
Lap3	orange	1.67821637	3.68E-24	1.59089081	1.02E-21
Ncoa7	orange	0.66316862	9.85E-05	0.78983211	4.90E-06
Batf2	yellow	4.02520941	2.73E-75	4.11272569	9.44E-79
Ldb2	red	-0.8224672	1.58E-06	-0.6396728	0.0003138
Plekhg5	red	-1.1057443	8.27E-11	-0.9458066	5.44E-08
Wdr34	red	-0.9468567	2.91E-12	-0.881527	1.45E-10
Slx4	orange	0.86498296	4.51E-05	0.81254365	0.00018617
Bahcc1	red	-1.1289389	5.20E-06	-0.8489638	0.000938

Orai2	orange	1.0776144	9.87E-14	1.09099951	5.66E-14
Exo1	yellow	3.22143289	6.44E-13	2.31340936	1.03E-07
Alkbh4	orange	0.50489331	0.00011414	0.6093587	5.73E-06
Dnajc28	skyblue	-1.9942291	0.00053881	-1.9833992	0.00078964
Cc2d2a	red	-1.1914336	1.39E-09	-0.7756954	0.00013993
Galnt12	orange	1.05297746	0.00012602	1.1047214	9.63E-05
Cep131	red	-0.6527293	7.30E-06	-0.4229734	0.00548159
Kmo	orange	0.71705062	0.0148362	1.49945734	3.41E-07
Tbc1d2	orange	0.7740339	0.00169425	1.12099433	5.64E-06
Xkr5	yellow	3.52380196	1.20E-06	3.23069936	8.96E-06
Nhsl1	red	-1.0122812	1.93E-08	-0.3729895	0.05640137
Mcph1	orange	0.7638606	0.00032885	0.77827023	0.00040283
Endov	red	-0.718085	3.03E-06	-0.6273886	6.93E-05
Rere	red	-0.7950968	7.50E-06	-0.5943043	0.00125425
Trim14	orange	1.13826342	1.43E-07	0.92416393	3.29E-05
Slc44a3	skyblue	-1.6152131	3.15E-10	-1.3266253	4.42E-07
Neurl2	red	-0.924689	1.46E-05	-1.0017167	7.99E-06
Lrrc17	skyblue	-2.991089	1.53E-32	-2.3799556	2.06E-22
Alg14	red	-0.8996346	1.55E-08	-0.7846374	1.40E-06
Txlnb	skyblue	-1.0338666	0.00057	-1.3725881	6.14E-06
Fgl2	yellow	2.23251646	4.35E-19	1.89346853	8.06E-14
Gpr37	skyblue	-2.9222265	0.00722594	-3.5027515	0.00219308
Slc26a11	red	-0.7784408	6.68E-09	-0.5774262	3.24E-05
Spsb1	orange	0.69500887	8.26E-07	0.36782739	0.01345297
Coq10a	red	-0.747492	5.12E-08	-0.7402506	1.37E-07
Urb1	orange	0.69157199	7.09E-06	0.60705338	0.00012682
Pik3cd	orange	2.0084118	5.73E-24	1.86739282	9.41E-21
Ptger4	yellow	2.19050978	1.96E-19	2.07216139	3.03E-17
Dag1	red	-0.614692	0.00080168	-0.3418459	0.08182587
Clstn1	red	-1.211619	6.90E-09	-0.8648078	6.59E-05
Stk32a	red	-1.0325545	0.00521796	-0.4486214	0.26487625
Mettl20	red	-1.1265811	1.18E-08	-0.9344544	4.13E-06
Rhou	orange	0.75456346	0.00027417	0.62136636	0.00386717
Ccdc40	skyblue	-1.5597109	1.03E-10	-0.8636278	0.0005727
Tbc1d16	red	-1.2156837	4.34E-08	-0.9903305	1.44E-05
Zc3h12d	yellow	3.25515687	3.86E-28	3.28829242	1.79E-28
Dtx4	orange	0.74572888	0.00265136	0.90449713	0.00032401
Phtf2	red	-1.2024061	2.47E-08	-0.6557669	0.00406744
Timeless	orange	1.2171242	3.11E-11	1.06290685	1.30E-08
Ifi203	yellow	2.30067562	9.82E-35	2.07180765	3.68E-28
Magi2	skyblue	-1.3510001	3.39E-07	-1.3370397	1.12E-06
Gnaz	red	-1.2269226	8.96E-07	-0.6074894	0.02456797
Slc7a5	yellow	2.24731415	5.36E-25	1.96605456	2.74E-19

Ptger3	skyblue	-1.5057221	4.47E-05	-1.1687332	0.00214336
Saa3	black	8.62825322	2.38E-192	8.054942	4.19E-173
Stat2	yellow	2.2683768	4.91E-43	2.19912052	1.57E-40
Nup43	orange	1.02011422	0.00083326	0.71121185	0.02795735
Negr1	skyblue	-2.8267826	1.58E-07	-2.0146434	0.00026451
Ift88	red	-0.9553746	3.66E-08	-0.6756364	0.00014729
Gjb6	red	-1.0405565	0.00081261	-0.7563478	0.01989989
Bub1b	yellow	2.49196379	5.40E-27	2.47512638	3.16E-26
Tnni3k	skyblue	-1.7379946	0.08416401	-2.6295825	0.00923128
Bmf	red	-0.9181501	2.60E-05	-0.7089293	0.00173207
Klhl42	red	-0.7048993	1.13E-08	-0.5475631	1.56E-05
Mettl11b	skyblue	-2.4458784	0.00307405	-1.1974563	0.20211957
Cacna2d1	red	-1.038095	3.35E-07	-0.8644655	3.98E-05
Gpr176	yellow	1.82512214	0.00090866	2.05820497	0.00034675
Abcc8	skyblue	-1.4816525	0.17678111	-2.7770117	0.00992267
9430038I01R	orange	0.90224361	3.67E-06	0.7836659	0.00011462
Rgl3	red	-0.946591	5.41E-09	-0.5738539	0.00067076
Maob	skyblue	-1.7953253	1.40E-10	-1.3834569	1.55E-06
Hs2st1	red	-1.0174466	3.42E-27	-0.7745447	6.37E-16
Thbs1	yellow	1.99165898	0.00013194	2.36111449	7.74E-06
Cd209c	skyblue	-5.0270131	1.63E-08	-1.4632144	0.01013972
Fmo2	red	-1.1005244	0.0025615	-1.1902583	0.00138198
Alkbh3	orange	0.5954988	6.57E-07	0.52267393	2.35E-05
Fmo1	skyblue	-2.0421631	1.38E-07	-1.6822958	2.50E-05
Ankrd6	red	-1.1368793	3.86E-05	-0.6639783	0.01959683
Scamp2	orange	0.78298446	1.02E-06	0.57891832	0.00051781
Ccdc114	red	-1.1202132	7.98E-14	-0.5271569	0.00073214
Tmem194	orange	0.66839594	1.48E-05	0.48589841	0.00260586
2810417H13	yellow	3.41269032	2.03E-09	3.73921952	9.55E-11
Zfp704	red	-1.3161964	2.19E-12	-1.0575934	3.59E-08
Emp3	orange	1.37079059	2.81E-07	1.00910797	0.00027433
Ttc12	red	-0.9290334	4.59E-08	-0.8305925	1.61E-06
Tm7sf3	red	-0.8043816	1.14E-08	-0.6630216	4.66E-06
Tbc1d10c	yellow	2.17870252	8.62E-40	2.27349575	6.41E-42
Lrp1	orange	0.6329984	0.00060824	0.56464278	0.00302247
Gbp7	yellow	2.77432941	3.62E-59	2.57297902	5.46E-51
Sema3d	red	-0.7817038	0.00014724	-0.5293837	0.01431699
Daam2	red	-0.964222	0.00030814	-0.7797401	0.00486138
Klhdc4	orange	0.76546484	1.92E-07	0.61912439	4.65E-05
Gbp2b	black	4.46208756	1.19E-20	4.77572561	4.85E-19
Dnm3	red	-0.6971648	0.00035963	-0.4687385	0.0225338
Pacsin1	yellow	2.24413265	2.17E-11	3.02051865	2.20E-18
Ndufa4l2	skyblue	-1.2515657	7.68E-06	-1.2334984	1.66E-05

BC052040	orange	0.43103732	0.09130012	0.75595036	0.0034709
Btnl9	skyblue	-2.0818507	3.46E-05	-1.7256596	0.00110893
Stac3	red	-1.4090916	1.52E-06	-0.8230706	0.00838504
Hey1	skyblue	-2.7539914	3.22E-16	-2.3105488	1.53E-11
Ddx58	orange	1.60102936	7.57E-29	1.52007155	5.44E-26
Btbd16	yellow	1.10295792	0.20540674	3.28271889	0.0009792
Cchcr1	orange	0.50070726	0.00240008	0.6133954	0.00025595
Olfr56	black	4.91275807	1.02E-78	4.61386871	5.16E-74
Arhgap9	yellow	2.40849241	5.43E-33	2.19929939	1.94E-27
Trim7	red	-0.7485767	0.00674957	-0.2922888	0.35438601
Mars	orange	0.52412777	8.20E-05	0.6407037	1.93E-06
Lyrm5	red	-1.1731919	1.19E-11	-1.2141401	5.91E-12
Klhl32	red	-1.5339884	0.0010417	-0.3055601	0.54670268
5330417C22	skyblue	-2.1018663	1.81E-09	-1.2519782	0.00060223
Slc25a28	orange	0.63993738	1.87E-05	0.64447279	2.47E-05
Plekha4	orange	0.81853351	0.0001761	1.15353402	1.38E-07
Pitpnc1	red	-0.7231455	5.09E-08	-0.7335009	5.48E-08
Slc26a10	red	-0.6396854	0.0071876	-1.324502	7.74E-08
Spns2	red	-0.9943052	3.97E-22	-0.6530502	7.23E-10
Mybbp1a	orange	0.87905035	2.46E-06	0.7382568	0.00012448
Blvrb	orange	0.68102757	0.0003366	0.52176827	0.00840168
Ggt6	red	-0.9715585	0.00276872	-0.9652557	0.00364019
Cfap69	red	-0.8664227	0.00049652	-0.6982231	0.00655836
Xaf1	yellow	2.74259972	1.07E-58	2.72889412	5.24E-58
Ltbp4	skyblue	-1.9492635	4.57E-09	-1.3350469	0.00011108
Chrm4	yellow	2.97546635	0.00023622	1.89101012	0.01359585
Pvr	orange	1.5558691	7.77E-09	1.48397164	6.30E-08
Tlr8	yellow	2.75194516	1.89E-06	2.0585181	0.00052424
Milr1	yellow	3.41105696	1.64E-18	2.84871163	4.65E-13
Adam22	skyblue	-1.9130149	2.61E-10	-1.3793355	7.47E-06
Pitpnm3	red	-1.2554803	8.24E-05	-0.735788	0.02926869
C3ar1	yellow	4.43027159	1.29E-12	3.75473846	3.67E-09
Wbscr27	red	-0.9332904	0.00939025	-0.6417261	0.09188018
Gstm2	red	-0.7634644	0.00020689	-0.4929867	0.02289136
Lppr2	red	-0.8643932	2.73E-08	-0.8182461	1.72E-07
Apoc1	red	-1.0525612	0.00588886	-0.7765178	0.05348277
Rundc3b	orange	1.69642684	3.66E-05	1.49878096	0.00033929
Abcb1a	red	-0.720323	4.57E-09	-0.6614578	1.21E-07
1110051M20	red	-0.9390723	4.56E-07	-0.6073389	0.00161691
Bace2	red	-0.8286394	3.10E-06	-0.7979466	1.42E-05
Apobec1	yellow	2.74722857	3.24E-18	2.30590363	5.81E-13
Tmem51	orange	1.18317621	2.34E-05	0.73493417	0.01268095
Aicda	yellow	5.14521619	0.00114055	0.77870785	0.5268967

Oaz2	red	-1.1845815	6.32E-18	-0.8942702	2.08E-10
Ppp1r14c	skyblue	-2.1902343	2.51E-10	-1.5092292	2.60E-05
Efhd2	orange	1.83400115	5.21E-12	1.59275173	4.16E-09
Sh3bgr	skyblue	-2.3926557	4.04E-16	-1.7032595	1.67E-08
Mthfd1l	orange	1.22665968	2.53E-09	0.81556594	0.00013656
Kremen2	orange	1.65750656	9.71E-07	1.0741463	0.00126509
Tbl3	orange	0.71736966	7.64E-06	0.77151443	2.16E-06
Col16a1	red	-0.7329452	1.42E-07	-0.4683999	0.0012845
Slco4c1	skyblue	-1.5816269	2.22E-06	-0.8202283	0.02066076
Apobec2	skyblue	-2.4339855	1.76E-08	-1.4475375	0.00139247
Limd2	orange	0.93357235	6.74E-14	0.85457378	1.51E-11
Ap1g2	orange	0.76332342	2.16E-10	0.61784531	6.29E-07
Cyp2s1	skyblue	-2.0711566	1.41E-21	-1.3866125	4.99E-10
A930016O22	skyblue	-1.1684159	0.03683756	-1.5358008	0.00849792
Sh3pxd2b	orange	1.76776139	2.40E-07	1.43283017	4.86E-05
Il17rd	red	-1.1603213	1.14E-05	-0.8153939	0.00306084
Zfhx2	red	-0.6720212	0.00037621	-0.1991544	0.34888193
Kcna2	red	-1.0248684	0.00030733	-0.917261	0.00171657
Cep126	skyblue	-2.0226396	3.15E-15	-1.1082199	2.20E-05
Rnf167	red	-0.8092253	0.00021152	-0.6099588	0.00728853
Cd53	yellow	2.88043622	2.79E-28	2.85846207	1.18E-27
Lat2	yellow	2.24216584	1.61E-10	2.24531885	2.70E-10
Cmtm5	skyblue	-2.2863031	0.00011561	-2.0261973	0.00087998
Snrnp25	orange	0.76353374	3.48E-06	0.41771481	0.01769114
Ttc3	red	-0.7920046	1.98E-08	-0.6945499	1.67E-06
S100g	skyblue	-2.0331265	2.77E-05	-1.5082617	0.00275131
Chil3	orange	1.54059738	2.53E-05	1.03766106	0.00667395
Agbl2	skyblue	-1.6204288	3.35E-07	-1.0092212	0.00194617
Snrpd2	orange	0.62275427	0.00169729	0.45547501	0.02879462
Zmynd15	orange	2.04741561	1.17E-12	1.62286916	3.96E-08
Gpr161	red	-0.5877994	0.00180137	-0.0965008	0.66403357
Six5	skyblue	-1.8009515	1.21E-21	-1.4286988	8.05E-14
Plekhh2	skyblue	-1.5532294	2.56E-07	-0.8594902	0.0066853
Reps2	skyblue	-1.8141344	2.66E-08	-1.5014575	7.62E-06
Crocc	red	-0.8462804	1.30E-08	-0.5646666	0.00026115
Osbpl10	red	-0.6139805	0.0048728	-0.4266658	0.06227677
Tmem205	red	-0.9172364	1.68E-07	-0.861392	1.54E-06
Kcnd3	red	-1.3190983	0.00056826	-0.4010061	0.33114872
Atp1a3	yellow	2.27453902	0.00011473	1.91176794	0.00169985
Ulk4	skyblue	-1.758092	6.95E-11	-1.0854079	7.84E-05
Slc16a11	red	-1.4706786	1.43E-07	-0.973517	0.00081858
Tet2	orange	0.60014635	1.35E-06	0.5884606	4.00E-06
Mgl2	skyblue	-2.2136844	3.72E-08	-1.356838	0.00118467

Cables1	red	-1.1181582	4.51E-10	-0.82276	8.81E-06
Arhgef38	skyblue	-1.965468	1.14E-08	-1.2020144	0.00077239
Gm11992	skyblue	-1.8707051	1.56E-06	-1.5013125	0.00012464
Mill2	red	-1.1325736	1.86E-06	-0.9232047	0.00016726
Sh3kbp1	orange	1.12441443	4.71E-09	0.97004246	8.62E-07
Abhd4	red	-0.761392	3.21E-05	-0.6156805	0.00113917
Npnt	red	-1.5020795	1.29E-07	-1.0233897	0.00055059
Cmtm8	red	-1.3124614	2.35E-14	-0.9827835	2.66E-08
Iffo2	orange	0.60499975	0.01850126	0.74010867	0.004382
Fam117b	red	-0.0767584	0.70193805	-0.5805902	0.00106069
Ramp3	orange	0.9255248	6.60E-05	0.72399813	0.00263113
Wdr43	orange	0.81519741	1.71E-07	0.75359958	2.41E-06
Wwp1	red	-0.8777518	3.46E-05	-0.4996452	0.02603649
Mslnl	yellow	2.5584806	0.00169479	3.35972077	0.00010002
Pif1	black	3.49334886	2.71E-21	5.11012889	2.79E-28
Nacad	red	-1.0040573	0.00017045	-0.5463491	0.05054142
Fzd7	red	-1.3010408	6.93E-12	-1.0263084	1.36E-07
Rwdd2b	orange	0.94035988	4.00E-07	0.99062645	2.50E-07
Ostc	orange	0.66676972	0.00072569	0.54478288	0.00795784
Ccdc8	red	-1.0486953	1.17E-15	-0.9414453	1.44E-12
Nbl1	red	-0.9800886	1.58E-11	-0.6896939	4.58E-06
Cyyr1	red	-0.7225879	3.59E-06	-0.6141563	0.00013281
Ripk2	orange	0.81730681	1.75E-10	0.73376491	3.00E-08
Tmco4	orange	1.12165666	8.35E-08	1.22049818	8.66E-09
Dnah7b	red	-1.4287559	3.75E-08	-0.5166322	0.05814212
Brca2	orange	0.91422911	0.00218258	0.82616622	0.00793612
Pla2g5	skyblue	-0.5453569	0.2929509	-3.1022589	1.04E-07
2310036O22	orange	0.63482289	4.04E-07	0.5639929	1.20E-05
Arhgap11a	yellow	2.46929768	1.11E-25	2.40526737	5.07E-24
Elovl6	red	-0.9137916	0.00764667	-0.9802825	0.00498585
Chd7	orange	1.61444126	1.04E-16	1.53309238	5.44E-15
Rbbp8	orange	1.13013392	1.44E-13	0.591153	0.00023247
Lamp3	red	-0.9096243	0.0001911	-0.6837891	0.00709476
Car8	skyblue	-1.9776577	4.93E-15	-1.3932935	8.37E-08
Tox	yellow	2.2120317	1.97E-29	2.07690727	5.53E-24
Katnal1	red	-0.7309089	1.36E-06	-0.5181952	0.00095638
Slc7a1	orange	1.45829852	1.85E-09	1.39580127	1.48E-08
Thoc6	orange	1.1012044	4.79E-06	1.30171295	9.65E-08
Ak7	skyblue	-2.5291161	6.84E-15	-1.5665001	2.83E-06
Inhba	orange	1.25112586	0.0015716	1.39375402	0.00059356
Atp1b2	skyblue	-1.458674	5.47E-13	-1.2679024	9.67E-10
Ankrd42	red	-1.0974228	2.69E-11	-0.682174	5.49E-05
Wrap53	orange	0.61871516	1.53E-05	0.47821762	0.00121424

Bdkrb1	orange	1.33212613	3.02E-05	1.44163394	1.17E-05
Ssr2	orange	0.62979448	0.00047436	0.57130222	0.00210403
Myzap	red	-1.1945491	0.00010991	-0.9565439	0.00278619
BC055324	orange	1.82468284	4.79E-07	1.45325953	9.43E-05
Paqr6	skyblue	-1.2981711	6.32E-06	-1.3673624	3.76E-06
Hibch	red	-0.8441047	2.17E-07	-0.6999718	3.44E-05
Nthl1	red	-0.7597312	1.08E-06	-0.5888631	0.00027228
Ccnb1	yellow	3.48102365	3.05E-40	3.80900097	1.01E-44
Cirh1a	orange	0.75476563	9.42E-05	0.68639014	0.00057818
Mfsd6	red	-0.6608748	1.68E-08	-0.4848338	6.28E-05
Arhgap32	red	-0.764034	0.00011523	-0.5145808	0.01306643
Mmrn2	red	-0.7016293	7.38E-07	-0.6044473	3.29E-05
Serpina3h	yellow	3.66397567	1.89E-18	3.39128305	5.60E-16
Cacna2d4	skyblue	-1.8928689	4.37E-06	-1.0457659	0.01520513
Smpx	skyblue	-1.9008906	1.20E-05	-1.7172258	0.00013058
Serpina3g	black	5.13266768	2.39E-64	4.23650002	1.48E-44
Piezo2	red	-0.8328795	0.00012214	-0.6596808	0.00335764
Zfp281	orange	0.95099592	1.11E-07	1.00931535	2.75E-08
Kif14	yellow	3.33652619	5.75E-25	4.0069147	7.79E-30
Rrp9	orange	0.56514755	0.00089739	0.58175142	0.00085121
Irf8	yellow	2.36035004	3.47E-39	2.20259036	3.90E-34
Upk2	skyblue	-2.1786311	0.00242996	-1.6602286	0.0255686
Ago1	red	-0.8148844	7.25E-09	-0.6460194	8.54E-06
H2-Ob	red	-0.6648722	0.00131658	-0.176518	0.46208699
Fmod	skyblue	-1.8215031	7.91E-05	-1.4972188	0.00166002
Sepw1	orange	0.71950511	0.00257337	0.55510835	0.02598613
Prelp	skyblue	-1.8077953	1.51E-07	-1.3367298	0.00017879
Sdk2	orange	0.75424993	1.96E-05	0.85292475	2.03E-06
Cdc42ep4	orange	0.84271986	4.57E-07	0.60893272	0.00045024
5730559C18	orange	0.7333982	0.01162718	0.82185727	0.00565212
Entpd3	orange	-0.00609	0.98388608	0.93905786	0.00012689
Ccdc74a	red	-0.8477757	0.00046377	-0.313239	0.23738202
Kctd12b	red	-1.4796499	1.35E-06	-0.9870997	0.00191748
Kif21b	yellow	1.94261852	4.84E-32	2.10648691	2.85E-37
Slc5a12	skyblue	-3.9314971	0.00138414	-3.5416472	0.00482379
Ddx24	orange	0.64142912	1.73E-13	0.50512867	1.49E-08
Pcca	red	-0.6217989	0.00028957	-0.5633745	0.00144874
Pnpla3	skyblue	-2.3962698	7.50E-07	-2.3409452	2.12E-06
Bbox1	skyblue	-2.5787129	0.00080013	-2.2053711	0.00392089
Prima1	skyblue	-0.8735071	0.16276005	-2.3654316	0.00108359
Lrrc18	red	-1.488588	3.90E-05	-0.519314	0.15189185
Fcho2	red	-0.5909195	0.00141886	-0.5103569	0.00769887
Amot	red	-0.9861762	8.14E-06	-0.5142081	0.0304049

Rasl12	red	-0.9307903	1.71E-06	-0.7172061	0.00037272
Lhfpl1	skyblue	-1.5282218	0.02412405	-2.7044234	0.00083942
Alg13	orange	0.68260593	0.00187099	0.63695772	0.00479434
Coro2b	red	-0.6616428	0.00193284	-0.56252	0.01089993
Pgm5	skyblue	-1.6381494	5.27E-13	-1.4196182	8.28E-10
Tspo	orange	1.50517378	1.19E-10	1.3098496	4.11E-08
Tmem45b	skyblue	-4.028869	3.14E-06	-2.0054651	0.02567331
Trem3	yellow	3.65901806	4.81E-11	2.16624176	2.23E-05
Plekha6	skyblue	-1.6682458	4.35E-13	-0.9919914	3.66E-05
Gpr155	skyblue	-1.6577961	3.88E-07	-1.7057042	2.65E-07
Ppp2r2d	orange	0.6759113	4.86E-06	0.45543971	0.0032073
Enc1	orange	1.70031364	1.44E-08	1.7360282	1.10E-08
Ydjc	orange	0.77581011	0.02399433	1.1386251	0.0009609
Mapk1ip1	red	-0.8225058	6.49E-05	-0.7501472	0.00037093
Cpsf2	orange	0.86960537	6.91E-08	0.62741098	0.00018236
2700046A07I	skyblue	-4.51352	0.00032701	-4.9156344	0.00010578
Abca9	skyblue	-1.5853134	1.39E-05	-1.5527104	3.18E-05
Gck	red	-1.4736752	4.56E-06	-0.4802	0.14505033
Efhc1	skyblue	-1.8210402	2.88E-08	-1.3124045	0.00010005
Oasl1	black	5.37644034	9.18E-62	5.44623564	1.61E-62
Abca8a	skyblue	-1.9398234	1.52E-10	-1.5423637	7.21E-07
Sytl3	yellow	3.27310583	5.20E-18	3.60862494	1.14E-20
Ptpre	orange	0.55843147	0.0042821	0.72534841	0.00025893
Fhdc1	skyblue	-1.7997563	1.75E-10	-1.1819354	5.12E-05
Mcm3	orange	1.75352205	7.99E-19	1.54202466	1.50E-14
Slc16a6	orange	1.48520318	3.37E-08	1.51753737	2.91E-08
Rnpep	orange	0.68780758	6.35E-07	0.62360695	1.13E-05
Fam222a	skyblue	-1.9671906	1.60E-08	-1.3668991	0.00012099
Tnfrsf18	yellow	2.61654242	1.82E-33	2.0348614	1.74E-22
Pkp2	red	-0.6301592	0.00127217	-0.522042	0.01025288
S100a10	orange	1.14803865	3.90E-11	0.95101812	9.29E-08
Znrf3	red	-0.6093104	0.0001465	-0.2259256	0.20412044
Mettl8	red	-0.9445608	8.70E-06	-0.5239883	0.01949919
Elmod1	skyblue	-1.9236858	8.08E-07	-1.1527837	0.00364824
Foxn4	orange	1.72220071	0.00018116	1.6339587	0.00021544
Acacb	skyblue	-1.3282737	4.91E-06	-1.3299726	7.42E-06
Ncapg2	yellow	2.79065277	1.61E-23	2.48983105	1.38E-18
Lce3b	red	-0.9402408	0.0047591	-0.6576249	0.06252158
Igsf3	red	-0.7300223	1.82E-05	-0.3612899	0.04913914
Sln	red	-0.611215	0.1298588	-1.4097715	0.0003155
Myo3b	orange	1.82107076	0.00039106	0.82292459	0.06755222
Abhd14b	skyblue	-1.4030875	7.49E-10	-1.2378384	8.73E-08
Hnrnpf	orange	0.58398767	3.41E-05	0.41824823	0.0044326

Arsb	orange	1.0055214	1.04E-05	0.58766746	0.01480186
4933440N22	orange	1.67989165	1.78E-05	1.46016514	0.0003044
Lce1c	red	-1.1229565	0.00053486	-0.8515846	0.01163667
Kank3	red	-0.9607795	5.87E-05	-0.8743555	0.00037751
Uggt2	red	-0.6305264	0.00498818	-0.2272022	0.36453992
Klhdc8a	orange	1.79168466	5.82E-07	1.79238216	9.32E-07
Vwa1	red	-0.9543735	3.66E-08	-0.4420948	0.01672506
Lce1f	skyblue	-2.1900285	2.92E-08	-1.1679667	0.00446923
Rassf4	orange	0.59838275	0.00098266	0.34832573	0.07317261
Klhl23	red	-0.986448	5.01E-06	-0.7334916	0.00106706
Dzip1	red	-1.2234345	2.81E-05	-0.7156715	0.01933886
Papd4	orange	0.70642859	7.80E-05	0.50563841	0.00677757
Tekt3	skyblue	-1.9206196	0.00115542	-1.3319072	0.02297122
Slc35f2	yellow	2.08198751	9.11E-10	1.85553278	6.09E-08
Chchd7	red	-0.5111744	0.00011853	-0.5964315	1.14E-05
Abhd14a	red	-0.7024448	2.00E-05	-0.6617667	9.51E-05
Zfand4	orange	0.78125223	0.00131581	0.59474098	0.01605276
Bag2	red	-0.786432	2.33E-07	-0.5176975	0.00090838
Sgsm1	red	-1.330397	2.09E-16	-0.5189597	0.00185448
Ammecr1	orange	0.62531919	0.00533573	0.35916837	0.13829776
Lyn	orange	0.64618726	0.00154894	0.50376732	0.01801915
Pm20d1	skyblue	-1.9338839	5.66E-06	-1.3637419	0.00174723
Cilp	skyblue	-2.7471217	1.53E-18	-1.0653708	0.00133636
Ccr8	black	5.12638906	5.13E-24	2.82658223	4.19E-10
Trem1	yellow	4.21479088	3.65E-08	4.36597265	2.52E-09
Fam92b	skyblue	-1.8356634	6.03E-05	-1.231626	0.00986802
Nxt2	red	-0.7518577	5.37E-07	-0.7576798	6.82E-07
Ehbp1	red	-0.6958875	0.00022316	-0.7852289	4.28E-05
Tnfrsf14	orange	1.16325605	1.11E-08	1.16669893	1.44E-08
Ctf1	red	-0.871154	0.00020364	-0.5412291	0.0311547
Ubash3a	yellow	4.42596379	1.04E-31	4.2908811	5.80E-29
Arl15	skyblue	-1.2696501	3.39E-18	-1.3699593	1.29E-20
Ikbke	yellow	2.13626144	4.71E-21	1.96158077	7.97E-18
Arel1	orange	0.66281244	3.21E-06	0.63707482	1.18E-05
Grap2	yellow	2.23265548	8.73E-31	2.23422084	1.61E-30
Gnl3	orange	1.13908758	1.59E-07	1.14352183	2.32E-07
Osbpl6	skyblue	-2.2331156	5.31E-09	-1.4728335	0.00021138
Esm1	skyblue	-2.0290977	5.55E-14	-1.7874229	6.23E-11
Gzmk	black	8.36891779	6.69E-28	8.11521894	7.86E-22
Atf4	orange	0.84198612	1.36E-08	0.80757674	8.90E-08
Zmym6	red	-0.5745089	0.00436597	-0.5911814	0.00400597
Ccno	orange	0.65714125	0.0212655	1.28066844	4.48E-06
Mgat3	skyblue	-2.0023675	2.30E-15	-0.9675021	0.00025644

Pih1h3b	skyblue	-3.3250173	0.00018254	-2.3560706	0.00631895
Zfp532	red	-1.1486269	1.56E-13	-0.8065793	4.60E-07
Fam63b	red	-0.6348006	0.00049927	-0.4895906	0.01001651
Mybph	skyblue	-3.2516335	8.55E-08	-1.2628022	0.05153961
Reln	red	-1.0580146	3.93E-05	-0.38806	0.16782533
Dctpp1	orange	1.30587392	1.23E-06	0.8439603	0.00301023
Fcmr	red	-0.8921554	0.00206739	-0.0328112	0.9323009
Abcb4	skyblue	-1.4825084	0.00016529	-1.1582598	0.0062958
Mustn1	red	-1.2250389	2.10E-17	-0.8483163	1.16E-08
Leo1	orange	1.07129469	3.83E-06	1.31621026	2.30E-08
Clspn	yellow	3.65779805	6.30E-38	4.22536127	4.93E-45
Ago4	red	-1.3038457	2.06E-10	-0.9167138	1.60E-05
Mum1l1	skyblue	-1.5620001	1.79E-06	-1.0538055	0.00152008
Dnal1	red	-0.7641934	0.00017985	-0.5183149	0.01525317
Kcnj12	red	-0.7832491	0.0036241	-0.8514705	0.00194063
Shfm1	orange	0.63090903	6.52E-09	0.43882087	0.00011128
Fam227a	red	-1.1880884	0.00022264	-0.6200643	0.06967166
Nek10	skyblue	-1.8289337	8.00E-05	-1.2404436	0.00507378
Cux2	skyblue	-1.6331233	0.00013047	-0.9867548	0.02756991
Kdm7a	orange	0.6120652	3.86E-06	0.26950554	0.05958685
Asb4	yellow	2.57348175	1.33E-05	3.032079	2.20E-06
Oscp1	red	-1.1356404	1.77E-08	-0.6589877	0.00167647
Maff	orange	1.11971831	2.80E-05	1.17510215	1.62E-05
Acad12	skyblue	-1.2786812	1.81E-08	-1.3295595	7.49E-09
Arrdc4	orange	0.48951044	0.0102157	0.65913008	0.00059381
Rgs8	black	4.71084253	3.37E-07	5.39769321	3.32E-06
Ypel3	red	-0.9065511	2.17E-07	-0.73556	4.59E-05
Zc3h12a	orange	1.80132519	3.07E-24	1.58077146	7.74E-19
Jph1	red	-0.9579678	0.00046848	-1.0479725	0.00021398
Mapk6	orange	0.81038813	3.41E-05	0.94408348	1.92E-06
Dnali1	skyblue	-2.3074154	1.06E-19	-1.6947215	3.61E-11
Wbp5	orange	0.74168423	2.88E-06	0.55683917	0.00072557
Ppp1r3a	red	-1.1864168	0.00051273	-0.9293369	0.00934734
Naa25	orange	1.32593354	1.39E-09	1.33709007	1.50E-09
Trafd1	yellow	2.24041725	9.87E-43	2.16416493	7.47E-40
Sgtb	red	-0.6270918	0.0047923	-0.3689121	0.11717971
Bex2	skyblue	-3.1851529	1.38E-09	-1.6749443	0.00212076
Nmnat2	red	-0.8551374	2.55E-07	-0.3770548	0.03844976
Apobr	yellow	2.38837385	3.01E-14	2.24734916	1.50E-12
Hebp1	red	-0.8856323	6.24E-07	-0.6506278	0.00044012
Muc1	red	-1.0373827	4.01E-06	-0.6840847	0.00361443
Fam166b	skyblue	-1.9333089	2.26E-14	-1.6254862	8.01E-11
Lgr6	skyblue	-1.8146202	4.31E-11	-1.4839155	1.15E-07

Flt3	orange	1.02301824	0.00053462	0.86365946	0.00489531
Fgf11	skyblue	-1.6096474	7.12E-16	-1.0397551	4.08E-07
Trim72	red	-1.1018683	2.63E-05	-0.7583371	0.00591569
Nrep	red	-0.9890667	7.23E-07	-1.0127932	6.64E-07
Lhfpl4	red	-0.9242824	0.00269898	-1.3189657	2.85E-05
Abra	red	-1.2998883	0.00022414	-0.6758262	0.07771448
Aida	orange	0.87439079	1.25E-15	0.71758108	1.15E-10
Foxo4	red	-1.1488404	1.77E-08	-0.9612663	4.43E-06
Egflam	red	-1.1204018	6.95E-07	-0.9774651	2.54E-05
Upk3b	skyblue	-1.9626179	1.80E-10	-1.7606996	2.09E-08
Notum	red	-1.0554499	5.71E-05	-1.1478336	2.52E-05
Rasef	skyblue	-1.7815861	5.06E-15	-1.0124048	1.47E-05
Gng2	orange	0.73262899	0.00127569	0.23878679	0.35137033
Klhl6	yellow	2.39106268	8.45E-21	1.99933216	1.25E-14
Wdr63	skyblue	-2.5937361	4.87E-13	-1.3483086	0.00026282
Trpv3	red	-0.9880601	0.00637344	-0.8910628	0.03106623
Ccdc63	orange	2.25458685	0.00978529	1.27943893	0.18621469
Zfp513	orange	0.77729823	2.05E-05	0.78990487	2.35E-05
Spice1	red	-1.2780256	3.12E-10	-1.0095707	1.15E-06
Tmem82	orange	1.01766772	0.01347178	1.25368552	0.00222948
Il17re	red	-0.9507244	1.36E-05	-0.4757481	0.04155561
Tuba1c	yellow	2.35908779	1.42E-21	2.05229405	2.32E-16
Qrfp	yellow	2.87641505	6.03E-12	1.6662136	9.52E-05
A530016L24I	skyblue	-3.0568754	5.96E-06	-2.3965861	0.00032775
Ispd	red	-0.9188653	0.00211361	-0.7905096	0.01125055
Ppp2r3a	red	-0.9893455	3.25E-06	-0.9254623	2.23E-05
Arl11	yellow	2.51414761	2.34E-09	1.95904109	6.05E-06
Pyurf	red	-1.0821807	5.05E-10	-0.3582803	0.0578672
Tmem212	skyblue	-2.0360945	3.22E-16	-1.1536634	6.23E-06
Lor	red	-0.6635672	0.00728793	-0.1927368	0.49752832
Fam129c	red	-0.9077138	0.00131282	0.02397341	0.950103
Exoc3l	red	-0.8316321	5.29E-06	-0.4923006	0.01061247
Tmem64	red	-0.9314682	8.41E-11	-0.7850415	8.61E-08
Fam13c	red	-1.1316073	0.00036431	-1.23102	0.00014583
Pyhin1	yellow	3.72358667	7.86E-36	3.59115498	4.51E-33
Trim56	orange	0.46212942	8.38E-05	0.61757844	1.72E-07
Pcdhb19	red	-1.2226621	0.00899358	-0.2157546	0.68771617
6530409C15	orange	0.58471278	0.32651735	1.59324825	0.0062221
Hoxd9	red	-1.3325489	0.00784478	-0.7503733	0.17336977
Olfr1342	skyblue	-1.965411	0.00183384	-1.1206529	0.08189391
Gprasp1	skyblue	-1.4810041	1.58E-16	-1.1909889	7.84E-11
2510009E07I	red	-0.7659294	1.13E-14	-0.5233686	3.33E-07
Otud1	red	-1.1254718	9.86E-10	-0.8774651	3.84E-06

Hilpda	red	-0.6666077	6.88E-06	-0.4299543	0.00571834
Gjc2	skyblue	-1.368561	3.73E-05	-1.1804447	0.00087928
Lce3d	skyblue	-1.8528788	1.50E-05	-0.8069379	0.08264014
Acot6	red	-0.9227501	0.00263688	-0.3551033	0.29618097
Tril	skyblue	-3.1976728	1.14E-06	-2.4121046	0.00038702
Lgals2	red	-1.2274127	0.00111951	-0.5894085	0.14829133
Gimap5	orange	0.79564739	3.98E-10	0.94547751	1.77E-13
Casc1	red	-1.3483357	5.44E-09	-0.9592443	4.51E-05
Zc2hc1a	red	-0.9425884	2.47E-09	-0.6492735	7.01E-05
Fbxl7	red	-1.1147515	4.66E-08	-0.7239389	0.00056798
Pxylp1	red	-0.6649143	4.28E-05	-0.7202512	1.33E-05
Mmp3	orange	0.28712691	0.29518256	1.05698553	4.06E-05
Ubxn10	skyblue	-2.5747542	5.65E-20	-1.6381289	8.79E-09
Ecm2	skyblue	-2.023011	4.76E-07	-1.576146	0.00014528
Adamts3	orange	1.50809489	5.17E-06	0.82803073	0.0178705
Rbm20	skyblue	-1.3111847	0.0675586	-2.3317075	0.00101936
Tmem221	red	-0.8350142	0.00012489	-0.8109664	0.00032456
Tox3	skyblue	-1.7558323	1.99E-05	-0.9190176	0.03423572
Kcns3	skyblue	-1.6973088	1.19E-11	-1.0004631	0.00010971
Fam25c	red	-1.1056632	0.00060552	-0.6302813	0.06789439
Capn13	skyblue	-3.1353259	0.00038967	-0.9744118	0.18894506
Col6a6	skyblue	-3.3865189	2.23E-11	-2.0671724	1.39E-05
B430306N03	black	5.19649422	2.34E-10	3.76295065	7.93E-07
Ly6g5b	orange	1.29990073	0.00818509	1.035759	0.04085119
Adamtsl5	red	-1.2243241	1.49E-10	-0.8923501	5.94E-06
Clec4a3	yellow	2.21420221	1.82E-11	2.30324584	6.64E-12
Taf10	orange	0.80693748	2.25E-05	0.641526	0.00116941
Ccdc60	skyblue	-2.0493003	5.30E-11	-0.8676427	0.00435633
Gimap7	yellow	3.23690933	1.70E-20	2.56001363	3.43E-13
Klri2	orange	1.35404147	0.00701231	1.86225435	0.00029685
Wdfy3	red	-0.6083201	7.97E-05	-0.4681774	0.00343306
Naalad2	skyblue	-1.2119793	7.16E-07	-1.4084895	1.97E-08
2900052L18F	red	-1.2761674	1.89E-05	-0.5716557	0.07350939
Mgat2	orange	0.6730175	0.00013792	0.63584146	0.00045706
Cilp2	skyblue	-2.5832001	0.0516669	-4.7318991	0.00035769
Slc35g1	orange	1.06425699	4.88E-06	0.55598354	0.02296852
Ccdc141	red	-0.9038341	0.00688713	-0.7365537	0.0342582
Als2cl	red	-0.6994191	2.38E-06	-0.3167268	0.04672432
Pcdhb14	red	-0.8010149	0.00169729	-0.5999523	0.02290554
Ccr10	skyblue	-1.7454382	0.00148411	-1.415958	0.01202275
Cep68	red	-0.6608847	8.24E-08	-0.591909	2.90E-06
Zrsr1	red	-1.3040836	3.44E-07	-1.156286	1.01E-05
Efcab8	yellow	2.35707911	0.00066201	2.61161525	0.00036126

Lmod3	skyblue	-1.9910355	1.70E-07	-1.8423627	3.34E-06
C130050O18	orange	1.56457893	6.33E-05	1.67651985	2.45E-05
Proca1	red	-1.5094775	1.03E-06	-0.9882965	0.0020355
Fam109a	red	-0.6641533	0.00156988	-0.8358639	8.99E-05
Arf6	orange	0.98242285	3.60E-10	0.79969025	6.78E-07
Hepacam2	skyblue	-3.6732129	1.38E-09	-2.5025763	1.65E-05
Tnip3	black	4.22605718	4.92E-29	4.57896174	2.40E-31
Bcl2l15	yellow	4.49967959	0.00011854	2.30563346	0.01282693
Cdc25c	yellow	4.26977561	8.70E-21	3.85014834	4.72E-17
Nxpe4	skyblue	-1.5481353	3.80E-19	-1.6395406	5.18E-21
Il20rb	orange	0.73724629	0.00030896	0.6796446	0.00130395
Osbpl1a	red	-0.6004082	1.46E-05	-0.6123232	1.51E-05
Zfp879	skyblue	-2.3239996	3.06E-05	-1.6138602	0.00300662
Mab21l3	orange	1.5669643	0.00443762	1.76036269	0.00162326
Ackr3	orange	1.29190652	3.80E-08	1.3099545	4.07E-08
Aplnr	skyblue	-4.6186406	1.83E-33	-3.5127477	7.83E-20
Phlpp1	red	-0.9656345	2.61E-09	-0.7901964	2.07E-06
Marveld1	red	-1.0981266	4.27E-13	-1.0186074	3.20E-11
Snhg11	red	-1.4346085	0.00068734	-0.9500378	0.03289882
Lacc1	yellow	2.76225059	5.35E-19	2.01968721	1.87E-10
Sowaha	skyblue	-2.8354106	1.72E-05	-1.0122453	0.11174706
Cxxc4	skyblue	-2.639874	1.93E-06	-1.6746267	0.00217445
Dsg2	red	-1.1306641	0.00015771	-0.900209	0.00348415
Adig	skyblue	-1.9998329	0.00068709	-1.8223643	0.00264183
Sptssa	red	-0.4926847	1.51E-05	-0.6363413	3.30E-08
Dock5	orange	0.648582	4.93E-06	0.43914333	0.00299959
Rin3	orange	0.62584481	1.43E-05	0.28795179	0.06348288
Shisa2	skyblue	-2.144078	8.99E-08	-1.4589191	0.00049755
Tnfaip8l1	orange	1.3413003	4.55E-09	0.92100478	0.00013613
A730020M07	orange	1.90702833	0.00552401	0.79041696	0.29149149
Tram1l1	skyblue	-1.4878193	0.0001679	-1.3758951	0.0005281
Rps2	orange	0.88648295	4.18E-05	0.85852597	0.00010802
Tceal3	skyblue	-2.4065427	1.52E-06	-0.9820144	0.05814212
Tlr7	yellow	3.01926413	3.54E-11	2.46186212	1.42E-07
Rnf208	red	-1.0497853	4.51E-05	-0.4508273	0.10761431
Cnrip1	red	-0.6304399	0.00028397	-0.4040273	0.03167462
B530045E10	skyblue	-2.4089726	8.26E-05	-1.670265	0.00656321
Zfp612	skyblue	-1.8914011	1.72E-10	-1.4143275	2.82E-06
2010007H06	skyblue	-1.5831758	1.29E-08	-0.8205819	0.00482379
Il27	black	4.78427496	3.88E-20	4.47602005	5.73E-18
Palb2	orange	1.33058654	1.09E-05	1.49268706	2.33E-06
Phf11a	black	6.1011627	1.73E-39	5.1349743	1.84E-32
Serpinb1a	red	-0.7969773	9.17E-06	-0.4855087	0.01077919

Scml4	orange	1.05422263	8.88E-11	1.45167337	3.84E-18
Sntn	skyblue	-2.1822633	2.70E-06	-1.9460427	3.96E-05
Zfp36	orange	1.35231116	1.77E-08	1.11749878	5.70E-06
Fads6	red	-0.8306481	0.00128083	-0.5767783	0.03274161
Isca1	red	-0.5784032	4.31E-07	-0.6889839	2.47E-09
Zfp354c	red	-0.6896601	0.004825	-0.8550407	0.00057655
AF251705	yellow	2.96725926	1.21E-18	2.49425176	2.90E-13
Tlr1	yellow	2.90880902	2.30E-20	2.77673709	1.88E-18
Ankrd45	skyblue	-1.9617417	2.03E-11	-1.3409732	2.76E-06
1700056E22l	orange	1.34195641	0.00415769	0.92003916	0.10777131
Ankrd50	red	-0.6292787	0.0004937	-0.218135	0.2793846
Gimap1os	orange	1.47309981	4.18E-07	0.93295412	0.00230716
Rassf9	red	-1.4818395	1.23E-12	-0.5687932	0.01013972
Ttc41	red	-0.5375501	0.03899741	-0.7051142	0.00673193
Klhl31	skyblue	-1.8103776	2.91E-08	-1.5617313	4.22E-06
Mylk4	skyblue	-2.22035	1.13E-09	-1.6947112	6.60E-06
Kctd21	skyblue	-1.5849458	2.51E-08	-1.2153418	3.07E-05
Fbxo48	orange	2.16333596	0.0004893	1.32265435	0.03668595
Wdr72	skyblue	-2.148272	2.52E-09	-1.7976192	1.36E-07
Tst	red	-0.9477632	3.41E-08	-0.7671035	1.46E-05
1110034G24	red	-0.4822606	0.02123653	-1.0779316	2.76E-07
Prss22	yellow	3.72394779	8.41E-11	3.73369741	3.70E-10
Cetn4	red	-0.6907523	0.01850126	-0.9623533	0.00132903
Tmem232	skyblue	-2.8652854	3.17E-10	-2.1096485	2.54E-06
Prkce	red	-0.7931571	4.61E-09	-0.7842069	1.12E-08
Megf8	red	-0.7019481	3.70E-11	-0.495949	6.20E-06
Rpsa-ps2	orange	2.51725097	0.00257915	-0.1090415	0.92182732
Zc2hc1c	red	-0.963955	0.00010094	-1.034184	3.89E-05
9930022D16	black	3.29056431	0.00077511	5.51856401	8.24E-05
S1pr1	orange	0.58311664	0.00011638	0.41837679	0.00807681
Arhgef37	orange	1.96282973	5.68E-15	1.85431417	3.80E-14
Magi1	red	-0.7456393	3.90E-05	-0.5680662	0.00255236
Slc25a26	red	-0.599648	0.00013031	-0.4915119	0.00276397
Dmd	red	-0.704963	0.00153816	-0.6536234	0.00434795
Rpl18a	orange	0.59844	5.19E-06	0.4247113	0.00189631
Tubb2b	orange	0.41532748	0.08109941	1.03524536	7.74E-06
AI467606	orange	0.57945387	7.84E-08	0.60263878	5.31E-08
Lrrc3b	skyblue	-2.7678531	0.00223714	-3.8306828	0.00021455
Kcng4	skyblue	-2.0997703	0.00951843	-1.6278126	0.05220313
Morn2	red	-1.2505446	3.00E-05	-0.5801948	0.07211015
Cenph	yellow	2.46290269	5.00E-07	2.33995247	5.27E-06
Lca5l	red	-1.1131057	9.55E-06	-0.0931878	0.75925746
Dcaf12l1	skyblue	-1.9353803	0.00565799	-2.0545711	0.0079273

Rtn4rl1	red	-0.9077803	3.33E-06	-0.6076775	0.00271131
Insig1	red	-0.7948404	3.06E-05	-0.4421113	0.0288559
Preb	orange	0.65865608	1.48E-12	0.50473995	1.32E-07
Fahd1	red	-0.684213	8.65E-06	-0.7971293	5.23E-07
Tlr9	yellow	3.35660067	1.57E-08	3.1459156	2.08E-07
Cenpe	yellow	3.43978037	7.19E-29	3.62640503	7.80E-30
Sh2d5	yellow	2.70225583	3.76E-08	2.71827182	3.70E-08
Tnfrsf26	yellow	1.80319405	2.65E-06	2.23800157	5.59E-09
Olfr1033	red	-0.6630956	0.00258185	-0.0987738	0.71045773
Kcnk13	orange	1.72861288	1.02E-05	1.14638044	0.00512564
1190002N15	skyblue	-1.7892917	3.54E-12	-1.3110411	7.64E-07
2810002D19	red	-0.6897982	0.00647209	-0.8435102	0.00124232
Ttll13	red	-1.2594766	0.00115174	-0.9337194	0.01521098
Lce3c	red	-0.94556	0.0074065	-0.666559	0.07420993
Pcdhb3	skyblue	-1.6118669	0.0026197	-1.4543232	0.0078712
Hcar2	orange	1.65258402	1.88E-08	1.57626874	1.02E-07
Sys1	orange	0.71356679	2.74E-08	0.49737577	0.0002308
Kcna5	skyblue	-1.4959836	0.00010729	-2.0643739	1.72E-07
Ddx28	orange	0.88307637	1.96E-08	0.61989361	0.00018491
Krt14	red	-0.8880228	0.00151933	-0.8362223	0.00379966
Mettl24	skyblue	-1.9598254	5.06E-15	-1.5388781	1.71E-09
Penk	red	-0.7433363	0.00625766	-0.6697648	0.01879095
Olig3	skyblue	-2.3886735	0.00015087	-0.9970336	0.09331292
Chrm2	red	-0.5714987	0.06048987	-0.9699192	0.00125396
Odf3l1	skyblue	-3.0080279	0.00625795	-2.5440635	0.00978171
Esf1	orange	0.6262064	0.00013317	0.189476	0.30757687
Sh3tc2	red	-1.1615255	9.65E-08	-0.9870747	1.03E-05
Mtus1	red	-1.1095954	4.27E-08	-0.8013099	0.00013408
Fam216b	skyblue	-2.4618453	7.85E-10	-1.4622381	0.00032384
Pid1	red	-0.414836	0.01007947	-0.6297109	9.86E-05
Col27a1	skyblue	-1.3952392	8.45E-14	-1.2670721	3.00E-11
Tcf21	skyblue	-1.2739325	3.26E-07	-1.292326	3.50E-07
Pcdhb4	skyblue	-1.6578436	1.32E-05	-0.9197756	0.02022007
Thtpa	red	-0.6129564	0.015786	-0.7872296	0.00180309
Smkr-ps	skyblue	-1.5853009	5.19E-05	-0.8985163	0.02392329
Mms22l	yellow	2.27217595	9.85E-22	2.36424136	3.48E-22
Zfp764	red	-0.4925958	0.00299652	-0.6084959	0.00029672
Fam179a	red	-1.2509597	7.85E-10	-0.6586737	0.00183101
Basp1	yellow	2.41657557	2.19E-09	2.51223666	7.80E-10
Slc16a5	skyblue	-3.0004332	0.00056002	1.41467123	0.12105763
Lrtm1	red	-0.8213686	0.00280628	-0.5004517	0.0951453
Ccdc149	red	-0.6357118	0.00124229	-0.4755002	0.02177577
Whamm	orange	0.68458264	1.32E-12	0.72581498	4.66E-14

Zfp36l2	orange	0.69795607	0.00358874	0.64788831	0.00868644
Ptprcap	yellow	2.60096738	2.87E-25	2.72172216	3.85E-26
Serpinb9	orange	1.0349298	1.26E-10	0.98162186	1.85E-09
Gvin1	yellow	2.56940512	5.84E-20	2.37491237	3.18E-17
Slitrk6	skyblue	-1.4595309	7.39E-06	-1.6736626	6.83E-07
Adra1a	red	-0.9496626	0.00631572	-0.5659801	0.12858455
Ccdc42	skyblue	-2.1322881	1.99E-08	-0.9504703	0.01605319
6330416G13	orange	0.97794679	1.13E-05	0.9610469	2.44E-05
Clec14a	red	-1.3609564	7.02E-08	-1.0544335	5.24E-05
Ifit2	yellow	4.15147551	3.47E-39	4.16654465	2.00E-39
Sdpr	red	-1.3586705	1.09E-05	-1.1053187	0.0005399
Slc25a51	red	-0.6725596	8.89E-08	-0.5059762	0.00010243
C2cd2	red	-0.892224	3.60E-10	-0.7832092	7.30E-08
Tmem104	orange	0.96034019	5.47E-05	0.57677069	0.02177577
Gapt	orange	0.6611528	0.30092181	2.20980532	0.00073108
Fam26f	black	5.95310499	1.01E-79	5.9214366	2.65E-74
Otulin	orange	0.90420269	6.71E-12	0.69536842	2.70E-07
Ppp1r15b	orange	0.67032005	4.13E-09	0.54229452	3.81E-06
Igfals	skyblue	-1.5987724	0.01362763	-1.7580415	0.00465738
Clec9a	red	-1.2591631	2.70E-06	-1.0209557	0.00022853
Hpcal4	skyblue	-3.4386724	1.35E-19	-2.1497475	4.30E-09
Krt32	skyblue	-1.7204638	0.00230497	-1.0227164	0.08832356
Cep295	orange	0.7533473	3.50E-07	0.7024683	3.32E-06
Fut10	red	-1.0911786	1.13E-07	-0.903704	1.68E-05
Tmem229b	orange	0.96603718	3.04E-08	0.85984786	1.52E-06
Chrm3	skyblue	-1.6920271	0.00066044	-2.099056	4.34E-05
Kcnrg	skyblue	-1.9344127	2.30E-12	-0.9699109	0.00048409
Adamts6	orange	1.6332276	1.43E-23	1.4298065	2.26E-18
E2f8	yellow	2.85486332	8.93E-31	2.92310081	7.95E-31
Gsg1l	skyblue	-2.892675	2.79E-06	-3.6982163	1.29E-08
Cd109	red	-0.8405852	0.00018708	-0.7640606	0.00121025
Pcdhb20	skyblue	-1.3908202	1.83E-08	-1.2880019	2.42E-07
Iqub	skyblue	-2.2010229	2.12E-13	-1.2508577	1.91E-05
Rprml	skyblue	-2.1412053	6.71E-12	-0.8747307	0.00684294
Plaur	orange	1.81584584	9.40E-09	1.75914796	4.59E-08
Scand1	orange	1.174877	0.00019858	1.4258943	1.18E-05
Hepacam	skyblue	-1.8611357	0.00021122	-0.6831349	0.2071387
1600029I14R	skyblue	-2.4314125	1.56E-13	-1.6550725	9.03E-07
Pilra	orange	2.06656837	9.50E-05	1.76376818	0.00122749
Tusc5	red	-1.2725907	3.66E-05	-1.1745365	0.00020025
She	red	-1.127515	3.76E-17	-0.8687885	2.09E-10
Ankle1	yellow	1.99006179	1.02E-13	3.31579827	8.88E-28
AI464131	red	-1.1318233	8.37E-05	-0.9941762	0.00081652

Stxbp6	red	-1.0390724	0.00031106	-0.5664578	0.06585494
Slc25a23	skyblue	-1.723698	3.65E-11	-1.0974499	4.79E-05
Fam178b	skyblue	-2.0476301	1.27E-06	-0.6425367	0.12403751
Smco1	skyblue	-1.3251881	0.12890946	-3.2586249	0.00359195
Asphd1	yellow	3.35591106	0.00180632	-0.0666134	0.9534411
Rbp1	red	-0.9978033	3.17E-12	-0.873061	2.43E-09
Irx3os	red	-0.9531899	0.00061118	-0.3642409	0.22945895
Lrrc75a	red	-0.9354804	5.73E-05	-0.7124988	0.0033641
Hnrnpa1	orange	0.62237221	5.47E-09	0.63702997	3.73E-09
Ppm1e	red	-1.562884	0.00111461	-0.6140025	0.2343724
Camk2n1	red	-1.1124003	2.18E-17	-0.8639618	1.15E-10
Sox18	skyblue	-1.6688763	2.10E-13	-1.3416782	7.81E-09
Scn4b	skyblue	-1.8663301	0.00028412	-0.8244228	0.14190572
C1qtnf2	red	-1.2502904	1.06E-06	-0.9634619	0.0003044
Ar	red	-1.3380506	9.05E-06	-0.9370934	0.00300698
Fam43a	red	-0.808872	1.55E-13	-0.681285	1.24E-09
Zfp319	red	-0.584011	0.00022565	-0.4330563	0.00863311
4930430F08I	red	-0.5626931	0.027109	-0.6797314	0.00975094
Cfap58	skyblue	-2.1928438	2.05E-10	-1.4035223	6.06E-05
Lrrc8e	red	-0.7002948	2.77E-05	-0.5542294	0.00153616
Ticrr	yellow	3.5858467	1.82E-23	4.15805107	1.16E-26
Olfml2a	skyblue	-2.5977209	1.36E-19	-1.7440468	2.69E-09
Ttc34	skyblue	-2.5761219	1.58E-08	-0.9814921	0.03594934
Zfp316	red	-0.8147206	1.44E-07	-0.5410016	0.00081202
Cxxc5	red	-0.9266662	1.28E-10	-0.4517485	0.00302696
Tmem251	orange	1.0800935	1.69E-05	0.75416077	0.00422169
Lce1l	skyblue	-2.0655258	1.18E-06	-2.064277	3.75E-06
C87436	red	-0.5918109	0.00151679	-0.3789688	0.05258304
Gm5424	orange	2.06908801	3.39E-05	0.44838403	0.4185153
Tifa	yellow	2.11669626	1.33E-29	2.10273658	1.20E-28
Mapk10	skyblue	-1.7196422	0.00074635	-1.778684	0.0007033
Hmga1	orange	2.05164855	1.44E-12	1.9403606	3.68E-11
Foxc2	red	-1.4235476	1.81E-05	-0.8759666	0.01201456
Bst2	yellow	2.53459333	4.74E-43	2.30153635	1.49E-35
Nxph3	red	-0.8530133	7.11E-05	-0.991502	1.26E-05
Cdc42se1	orange	0.61952784	1.22E-07	0.47069414	0.00010496
Fat4	red	-1.2253907	1.98E-18	-1.0169497	9.39E-13
Epm2aip1	red	-0.5873845	0.00113841	-0.3169909	0.10174106
Phgr1	yellow	3.04402274	0.00067256	1.28841919	0.10559857
Mpeg1	yellow	2.88654841	1.96E-13	2.63601413	3.58E-11
Lrrc75b	red	-1.1848647	0.00519243	-0.7378493	0.09737018
Ddit4l	skyblue	-1.8068175	3.37E-08	-1.1521734	0.00082251
Ckap4	orange	0.69118339	0.00223964	0.58541721	0.01256485

Vat1l	skyblue	-2.3780313	1.44E-06	-1.4398053	0.00382646
Fbl	orange	1.22219333	2.42E-07	1.10510337	5.30E-06
Irgm1	yellow	3.8854562	2.77E-117	3.57868399	3.28E-100
Zfp474	red	-1.1549447	2.14E-09	-0.4852523	0.01448522
Ltb4r1	yellow	2.27454054	1.45E-11	2.35579153	4.80E-12
Myct1	skyblue	-1.8016145	8.93E-32	-1.4091581	2.38E-19
Wdr27	skyblue	-1.5715661	2.00E-05	-0.6962988	0.07216343
Gm5637	yellow	2.25635374	0.00025131	1.7467706	0.00506391
1110032F04I	orange	2.27681195	0.00017013	1.32078057	0.04863519
Ccdc108	red	-1.4936249	2.06E-06	-0.74289	0.02599963
Nipa1	red	-1.1153587	1.69E-06	-0.7022488	0.00412588
Dusp28	orange	0.77442645	1.95E-09	0.70262104	1.28E-07
Lrrc4b	skyblue	-1.3346677	0.00090628	-1.4271846	0.00065019
Rnf31	orange	0.92892954	4.86E-23	0.93645676	2.09E-23
Fam221a	red	-1.1472301	1.74E-05	-0.8537549	0.00204766
Tet1	skyblue	-2.1527667	9.99E-07	-0.9566514	0.04094549
Neurl3	yellow	2.0374723	1.23E-16	2.22452408	1.77E-19
Irs3	red	-1.4950144	0.00172043	-0.6719412	0.18859746
Dync2h1	red	-0.9354251	2.59E-06	-0.9748524	1.33E-06
Gjd3	yellow	3.98944776	0.00205534	0.99237156	0.38422982
Dusp18	skyblue	-1.5971743	1.10E-18	-1.0723337	5.56E-09
Cdh19	skyblue	-1.1166313	0.00691538	-1.3882465	0.00122587
Gm527	red	-0.6268171	0.05894414	-0.9223468	0.00749147
BC048546	skyblue	-2.8103087	6.95E-24	-1.9908718	3.06E-13
Cldn2	orange	1.58055582	0.00152534	2.27122232	9.47E-07
Mageh1	red	-0.7379297	8.99E-06	-0.4186639	0.01699661
Hist1h2be	skyblue	-2.3043713	9.92E-06	-1.4622744	0.00594364
Ptgs1	orange	0.67699604	1.66E-07	0.51083699	0.00013103
Gap43	skyblue	-2.1387724	4.55E-12	-2.0964345	2.59E-11
Sfn	orange	0.72956191	0.00872639	0.75367286	0.00813306
Gpr15	yellow	4.12357841	1.69E-10	2.6644091	3.01E-07
Kcne4	orange	0.68687862	0.04068867	1.0814678	0.00114448
Lancl3	orange	2.18978377	0.00088798	1.1645418	0.09769153
Gm973	skyblue	-2.3862728	6.34E-17	-1.6338009	1.49E-08
Dnah14	orange	0.50072547	0.22563135	1.0790159	0.00817445
B4gat1	red	-0.7467393	1.63E-08	-0.6532939	1.51E-06
Odf3b	skyblue	-1.9524914	1.18E-08	-1.2916875	0.00026451
Tgif1	orange	1.50607142	2.90E-07	1.38089212	4.40E-06
Ctdspl	red	-1.1414965	0.00010316	-0.8736668	0.00421081
Gpr68	yellow	3.48053126	3.56E-21	3.0631056	3.45E-16
Cmya5	red	-0.85576	0.00110412	-1.0492576	8.25E-05
Fam180a	skyblue	-0.9491807	0.04270654	-2.5555104	1.37E-06
Zfp30	red	-1.5268511	4.71E-08	-1.016864	0.00032748

Klhl34	skyblue	-2.1967489	0.00310235	-1.3507494	0.08106451
Adamts12	orange	1.079689	0.00516066	1.31100656	0.00094042
Cldn4	orange	1.4181113	3.48E-06	1.43711052	3.99E-06
Mis18bp1	yellow	3.58792986	4.96E-18	2.87689249	4.78E-12
Cltb	orange	0.61513504	0.00028532	0.56706414	0.00115254
Mafa	red	-1.3234947	0.00353421	-0.3924891	0.45243506
Nxpe5	yellow	2.72055206	5.14E-07	3.07401497	4.16E-08
Zfp235	red	-0.6457567	0.00930167	-0.583746	0.02220696
Apof	black	5.49066552	0.00061568	2.75548334	0.05640654
2810006K23l	red	-0.7031307	0.00603436	-0.8178391	0.00183353
Krt83	orange	2.04036076	2.44E-06	1.75998019	4.61E-05
D930020B18	skyblue	-3.2183401	2.25E-06	-0.940223	0.16801699
Fbxo30	red	-0.9463243	8.07E-08	-0.8158331	6.26E-06
Trpt1	red	-0.66227	0.000181	-0.5263653	0.00371869
Gal3st3	skyblue	-1.4324686	0.01265361	-1.5593689	0.00626177
Tctex1d4	red	-1.2260619	3.20E-06	-0.7300468	0.00726805
Rpsa-ps10	orange	1.11731772	3.19E-08	0.62248057	0.00508614
Bola2	orange	0.79423212	1.67E-05	0.64628766	0.00081273
Samd9l	orange	1.64397797	2.26E-30	1.59419142	1.69E-28
Fbxo40	skyblue	-1.5132189	1.54E-06	-1.1465284	0.00046545
Rnf150	red	-0.9187061	1.22E-05	-0.7077941	0.00117642
Zc3hav1l	red	-0.9114313	5.93E-06	-0.7791051	0.00021709
Utf1	yellow	4.91441962	0.0001125	2.15129113	0.02381139
Lix1	skyblue	-1.9085459	0.00049214	-1.4464067	0.01263326
Sned1	red	-1.0079376	0.00028773	-0.6524271	0.02535315
Gjb1	skyblue	-1.705749	0.00758204	-1.3262687	0.04919595
Cd300lf	black	4.61908725	2.29E-32	4.44202754	5.41E-30
Ccdc88b	yellow	2.90019864	2.64E-23	3.01537824	5.47E-25
Cdca4	orange	0.96384666	8.49E-10	0.86892309	7.25E-08
Diras2	skyblue	-2.7262076	2.77E-16	-1.4991711	1.28E-05
Bex4	red	-1.0370684	0.0043305	-0.8198635	0.03059368
Stx19	red	-0.7536577	0.00888401	-0.7160077	0.01802762
Ccr4	orange	1.07147251	0.11549192	2.44724389	0.00071436
Marcksl1	orange	1.57239031	1.18E-14	1.71577941	5.50E-17
Kcna3	yellow	1.74377819	8.32E-06	2.48125125	7.63E-10
Stbd1	red	-1.0977135	1.69E-09	-0.6357669	0.00090364
Kcna1	red	-1.0759941	0.00033119	-0.1126979	0.76218079
Ino80c	orange	0.70075854	3.22E-05	0.59246939	0.00066941
Prrg1	skyblue	-1.452131	9.55E-07	-1.2802058	2.69E-05
Timm8a1	orange	1.09010808	0.00026389	0.76983872	0.01447743
Zfp473	orange	2.03968292	9.39E-05	1.83655659	0.00076036
Rgmb	red	-0.8989998	4.03E-08	-0.6826552	5.51E-05
Eno4	skyblue	-2.5289295	2.56E-15	-1.2165131	0.00018884

Fcrl5	yellow	2.7983237	3.76E-06	2.98321897	5.74E-06
4932418E24I	skyblue	-1.7303109	2.30E-10	-1.1070666	9.16E-05
Arxes2	skyblue	-1.440559	0.00100506	-1.3386002	0.00304265
Ldlrad3	red	-0.6236859	3.51E-05	-0.6125909	7.42E-05
Cyb5r2	red	-1.4386091	0.00548566	-0.9155698	0.10770939
Lmod1	skyblue	-1.557061	5.25E-15	-1.0476547	3.39E-07
Entpd1	orange	0.60035353	1.75E-05	0.41292018	0.00465794
Nat8l	skyblue	-2.3906609	1.91E-07	-2.2363177	1.75E-06
Selplg	yellow	2.82158401	1.84E-39	2.57260736	8.33E-33
Bend7	red	-0.7615099	3.06E-05	-0.639016	0.00068023
Cracr2b	red	-1.3087571	3.81E-07	-1.0933038	3.75E-05
A630023P12I	orange	0.68461416	0.14942775	1.2470738	0.00947555
Gpr85	orange	1.05829597	0.11463023	2.17350791	0.00146004
Amigo2	red	-0.6444991	0.00042252	-0.5963539	0.00151757
Fbxo10	red	-0.9782613	2.08E-07	-0.7524649	0.00010256
Rnf149	orange	2.07342396	5.86E-11	1.77333861	4.32E-08
Gng7	red	-1.0551202	1.38E-05	-1.2304113	3.95E-07
Bcl11b	orange	1.26342532	0.03249164	2.32534436	7.07E-05
Syngr2	orange	0.76005828	1.39E-05	0.57928356	0.00141634
Frmd6	red	-0.781361	6.75E-05	-0.6038768	0.00297639
Ckap2l	yellow	2.64382219	2.07E-18	2.72628903	8.89E-19
Ric3	red	-1.4344434	7.17E-05	-0.9278759	0.01117103
Lhfp	red	-0.6627485	1.03E-06	-0.4469317	0.00157477
Omd	red	-0.6032633	0.25475363	-1.6397681	0.00119713
Pdp2	red	-0.7163323	0.00113016	-0.8187056	0.00028708
Scrt1	yellow	1.67316701	0.08576287	4.48911088	0.0008215
Osr1	orange	0.57561182	0.0008966	0.89946715	2.19E-07
Fam171b	red	-0.8786039	0.00191592	-0.198213	0.56492847
Gli2	red	-0.5817251	0.01168153	-0.9820351	2.42E-05
Mlf1	skyblue	-2.0504711	4.22E-14	-1.5327821	4.00E-08
Smim5	skyblue	-1.5449991	2.98E-08	-1.0654689	0.00020654
Sprr1b	skyblue	-2.3355198	0.00153315	-1.1605718	0.14541102
Fam212b	red	-1.0726741	2.53E-12	-0.5512004	0.00061876
Mypop	red	-0.6511083	0.00767669	-0.8005214	0.00109097
Bdnf	red	-0.9475133	0.0001962	-0.5211815	0.05444982
Fitm2	red	-0.716798	1.14E-05	-0.7401693	8.76E-06
Fbxl13	skyblue	-2.7670612	4.97E-13	-1.1620231	0.00125303
Cxcr6	yellow	4.20160403	2.15E-64	3.47754566	9.30E-44
Amica1	yellow	3.81266368	2.50E-31	2.66564384	7.92E-16
Phldb1	red	-0.7738871	1.56E-07	-0.6186394	4.76E-05
Tmem252	orange	0.71981714	0.00438405	0.25533088	0.36764959
Gm6377	yellow	3.47274008	1.79E-12	1.97002224	2.58E-05
Ctxn1	skyblue	-2.0189844	1.33E-23	-1.1942254	8.07E-09

Tpcn2	orange	1.16757037	3.36E-08	1.17835057	4.08E-08
Lurap1l	red	-1.3867985	3.02E-28	-0.8564279	3.68E-11
Tprn	orange	0.81471422	4.67E-05	0.40041129	0.0622766
Fndc9	orange	1.03387225	0.00059751	0.48233581	0.13829776
Zfp454	skyblue	-2.0583814	0.0021178	-1.346794	0.04904493
Ggnbp1	red	-0.7985412	1.39E-06	-0.9253204	5.87E-08
Klhl11	orange	0.66571798	0.0445292	1.19170421	0.00033184
Rpl29	orange	0.62984752	1.67E-08	0.56844176	6.35E-07
Tmem53	red	-0.6392322	0.00493842	-0.3620547	0.1347641
P2ry6	yellow	2.1163198	1.01E-08	1.67516218	1.09E-05
Ccdc37	skyblue	-1.8188396	1.76E-06	-1.3547765	0.00054944
Ifnb1	black	6.9757004	3.20E-06	6.92852553	6.19E-06
Lonrf2	skyblue	-2.7589536	7.42E-07	-2.7758928	2.47E-07
Dact2	red	-1.1089693	0.00211361	-0.6262507	0.10512577
Pkd1l3	orange	0.93320932	0.0004633	0.16301289	0.60061945
Vstm2a	skyblue	-2.9867295	0.00235915	-1.5091637	0.0933323
Gm12185	yellow	3.2029912	1.78E-48	3.10344617	8.01E-48
Arhgap30	yellow	2.49553228	8.16E-26	2.57246983	3.03E-27
Cdk5r1	orange	1.37211223	1.83E-06	0.9588087	0.00127013
4930539E08I	yellow	2.75482077	1.82E-05	3.82576935	3.66E-07
Cdca2	yellow	3.31190818	1.12E-36	3.01818606	7.00E-31
Prex2	red	-1.352691	1.49E-07	-1.0957188	3.60E-05
Yjefn3	red	-1.1803163	5.70E-05	-0.9376216	0.00162709
Elfn1	skyblue	-1.4585491	7.87E-08	-1.1733318	2.67E-05
Clec4a1	yellow	3.07635729	3.60E-18	2.65619018	1.37E-13
Ccr2	yellow	2.42449115	9.86E-26	2.61396709	1.85E-29
Ntf3	skyblue	-1.8533587	4.71E-06	-1.2363443	0.00357209
Themis	yellow	2.90101295	1.05E-09	4.6396881	3.92E-21
Agtr1a	red	-1.0187663	0.00087243	-0.7737471	0.01473273
Gm8186	orange	0.6868353	9.50E-05	0.52898248	0.00364693
Ivl	orange	2.26523355	0.00347628	1.14318275	0.1370462
C5ar1	yellow	2.68260783	1.88E-06	2.37305791	4.05E-05
Nrap	red	-1.4246159	4.37E-08	-0.8392497	0.0021429
Fam183b	skyblue	-2.0138207	1.13E-12	-1.1912849	3.93E-05
Myoz3	skyblue	-2.326843	2.92E-07	-1.5632823	0.00116975
Purg	red	-1.062671	5.31E-07	-0.9169305	2.91E-05
Hcar1	skyblue	-3.2675162	1.16E-07	-2.346315	0.00011927
Kcnk3	red	-1.0171304	2.32E-05	-1.0118104	3.89E-05
Scn3b	red	-0.8680773	3.00E-05	-1.0734293	3.41E-07
Syt12	orange	1.73800028	2.02E-07	1.82687684	5.71E-08
Fut4	orange	1.53198199	1.43E-09	1.07717719	3.57E-05
Zfp2	skyblue	-1.5929218	0.00175657	-1.2824219	0.01494929
Krt8	orange	1.50752228	1.37E-06	1.58976581	5.37E-07

Ogfr	orange	1.94659293	5.62E-26	1.81633525	1.26E-22
Zfp683	yellow	3.5927593	8.27E-13	3.55109901	6.70E-11
Tmem200a	skyblue	-1.7258001	1.16E-19	-1.3659779	9.65E-13
Chchd10	red	-1.3359249	2.27E-07	-1.0030918	0.00017944
Ctu2	orange	0.7740726	1.36E-06	0.75457364	4.27E-06
Slc36a3	black	4.83118866	0.00209699	2.87738168	0.02483603
Dtx3l	yellow	2.26363423	3.00E-46	2.19659493	1.19E-43
Htr1b	skyblue	-2.6838399	6.69E-12	-1.6521828	3.80E-06
Rps23	orange	0.58569735	3.28E-10	0.41760282	1.53E-05
Cdc42ep1	red	-0.9489085	4.65E-13	-0.8169318	9.51E-10
Sall2	red	-1.2274369	2.19E-14	-0.9474548	8.34E-09
Tceal1	skyblue	-1.5400434	3.90E-13	-1.2159968	2.48E-08
Tmie	red	-0.9522582	0.00840928	-0.7420002	0.0492554
Lingo1	skyblue	-1.1010905	0.00425443	-2.0249567	2.89E-07
Ap5b1	orange	0.87696953	1.15E-05	0.83010911	5.34E-05
Cfap46	skyblue	-2.0867584	5.11E-17	-1.1574633	4.31E-06
Ccdc69	orange	0.71342553	7.82E-05	1.10069059	6.84E-09
Lce1h	skyblue	-1.6823581	2.61E-05	-0.6896141	0.11040243
Gpr55	yellow	4.06901008	1.09E-22	3.68823733	4.22E-18
Slc17a5	red	-0.7470332	0.00079183	-0.7946261	0.00047163
Tifab	yellow	2.26189324	4.83E-09	2.12873242	6.48E-08
Zbtb14	red	-0.8808251	8.93E-06	-0.7124575	0.00048805
Nckap5	red	-0.9187556	1.33E-11	-0.9150717	2.75E-11
Nlrp10	red	-0.9015037	0.00735249	-0.8612587	0.01226435
Prss46	black	6.35339685	1.58E-05	6.80841606	6.06E-06
Gal3st1	red	-1.1076182	0.00015527	-0.7398162	0.01513619
Mmp12	orange	1.60573047	0.00195791	1.06030965	0.05300583
Zfp668	orange	0.84589956	1.92E-09	0.36581006	0.01467513
Arhgap15	orange	1.98448698	5.09E-13	1.843129	4.81E-11
Rpl36al	orange	2.10187126	5.06E-08	1.07613705	0.00469432
Tmsb4x	orange	0.73514367	2.22E-10	0.44626719	0.00022907
Armcx4	orange	0.63476724	0.00015913	0.72831566	2.09E-05
Arhgap23	red	-0.6110377	5.43E-08	-0.4317498	0.00021398
Fam161a	skyblue	-2.2605433	1.12E-18	-1.5618507	2.15E-09
Arl4c	orange	0.81253123	5.74E-07	0.93927994	1.06E-08
Nlrc3	yellow	2.66339854	2.72E-22	2.4921321	2.36E-19
2810025M15	red	-0.5310483	0.0023952	-0.7798521	9.76E-06
Vcpkmt	orange	1.01908094	1.87E-05	0.92482252	0.00017669
Rasd1	orange	1.15598986	0.00030561	0.59992812	0.07836533
Rasl11b	orange	0.69481096	2.49E-05	0.70635683	2.78E-05
2610318N02	yellow	3.13703948	7.19E-06	4.48779472	2.06E-09
H2afx	orange	1.40085477	3.17E-11	1.21965445	1.81E-08
Lrrc4	yellow	2.53441954	1.99E-06	2.62626144	2.93E-06

Mrps16	orange	0.69619257	7.12E-06	0.46310402	0.00471852
Ankrd55	yellow	2.45560522	6.92E-05	3.70742891	4.62E-07
Lrrc25	yellow	4.38463558	5.06E-36	3.95970683	8.95E-30
Idnk	orange	0.94873062	6.18E-34	0.85227175	1.43E-27
Apol10b	yellow	3.72396786	9.02E-52	3.08982649	1.93E-36
Rap2c	orange	1.00474185	6.76E-09	0.87053093	9.70E-07
Tdrp	red	-0.8982482	0.00105813	-0.7102735	0.01276048
Zfp697	orange	0.64712072	0.00142141	0.49551493	0.01911313
Grem2	skyblue	-2.3348963	4.85E-24	-1.6261517	5.32E-12
Gpr171	yellow	4.23463061	4.86E-80	4.28676641	1.51E-67
Ces2b	skyblue	-5.0684993	8.15E-15	-2.9624894	5.12E-09
Agmo	skyblue	-1.6738169	6.60E-05	-1.457259	0.00074802
Grrp1	orange	0.67135711	5.05E-06	0.51138134	0.00092915
Tmc8	orange	1.10696727	9.77E-08	1.22050483	6.87E-09
Gsg2	yellow	2.58601816	1.24E-14	2.88181349	1.21E-16
Vwa3b	skyblue	-1.7016276	1.59E-07	-0.9661222	0.00364483
Kcnk12	skyblue	-2.1569815	3.31E-07	-0.8799198	0.04249518
F2rl3	orange	2.00312067	1.77E-06	1.43910471	0.00108436
Gm867	red	-1.0971802	1.03E-07	-0.5886897	0.00588016
Lgr4	red	-0.6186474	0.00023558	-0.7734352	5.67E-06
Eva1b	red	-0.7108736	2.76E-06	-0.5705948	0.0002831
Il17d	skyblue	-1.7748309	6.47E-10	-0.8738843	0.0042424
Cxcr3	yellow	4.49193828	1.88E-49	3.93307487	9.77E-39
Klre1	orange	1.6278577	6.63E-06	1.39146963	0.00024518
Heatr1	orange	0.95172359	1.14E-08	0.81361188	2.03E-06
Evc2	red	-0.9934984	1.76E-13	-0.6989778	4.25E-07
Tmem220	red	-1.0026908	0.00023606	-0.9195704	0.00087688
Fzd2	red	-1.2759182	7.76E-12	-0.7039041	0.00032749
Gm9843	orange	1.15640511	0.000108	0.19352966	0.59452712
Synpo2	red	-1.1822186	1.99E-18	-0.762666	4.81E-08
Lgals3	orange	1.84474124	7.20E-10	1.69085937	2.97E-08
Gpr18	yellow	2.39372847	6.95E-24	2.14681671	1.77E-19
Rltpr	orange	1.57477485	4.32E-06	2.30947534	1.61E-11
Ch25h	black	4.69067944	1.13E-44	4.38303783	4.77E-39
Snx21	red	-0.5899024	0.00408082	-0.7848311	0.00015306
Sept6	orange	1.43410674	5.64E-30	1.48028403	6.94E-31
Tcf19	orange	1.62831293	1.59E-21	1.43687172	7.43E-17
Enthd1	black	4.79771693	0.00150667	3.64349465	0.00370354
Hamp	skyblue	-1.8417462	9.43E-06	-1.4232885	0.00080239
Lypd6	skyblue	-2.2028757	0.0003482	-1.0675733	0.09509652
Krt78	red	-1.0272448	0.00360034	-0.7161237	0.05452806
Slc35d3	skyblue	-2.5424473	7.68E-05	-0.9613297	0.14110948
Fbxl22	red	-1.3057936	1.43E-05	-0.8193441	0.00956962

Cldn8	skyblue	-2.8357	4.84E-17	-2.116452	3.13E-11
Fam171a1	red	-1.1160176	8.70E-09	-0.9748645	9.12E-07
5730508B09I	orange	0.64429503	0.0092285	0.65699353	0.0100529
Hyls1	orange	1.13200467	1.77E-11	0.8858359	2.78E-07
Kcnb1	red	-0.8848447	8.83E-07	-0.4434431	0.02109278
Tor1aip2	orange	0.86909386	1.20E-08	0.84298109	5.57E-08
Mmp13	yellow	3.66291551	8.80E-11	2.71238342	1.25E-06
Lrrc4c	skyblue	-1.0936654	0.03803569	-1.9814576	0.00016881
Fam78a	orange	1.58764769	1.53E-20	1.58008118	5.18E-20
Zfp831	orange	1.40961441	1.37E-06	1.3912585	4.12E-06
Ccdc121	skyblue	-2.5082348	5.36E-16	-1.2242698	3.07E-05
Gpd1l	red	-0.758102	2.12E-08	-0.647917	3.07E-06
Tmem150c	red	-0.6831305	0.00228947	-0.6234536	0.00714047
Ccdc96	skyblue	-1.6421815	2.58E-11	-0.9859743	7.24E-05
Ftl1	orange	0.72790576	0.0011147	0.43181784	0.06962969
Plekho2	orange	1.73272423	5.51E-11	1.6545519	6.52E-10
Arhgap42	red	-1.0843746	2.30E-05	-0.747397	0.00510901
Ptges	orange	1.30773698	9.80E-06	1.31488485	1.34E-05
Trim15	orange	1.21059328	0.00605317	1.70963314	0.0001401
Gm5084	skyblue	-2.5165189	5.86E-05	-0.844231	0.14929956
Tmem37	orange	1.57381168	1.64E-18	1.42587798	3.13E-15
Fam131a	red	-0.9537518	1.55E-07	-0.8986898	1.35E-06
Rtn4rl2	yellow	2.81800583	2.90E-15	2.59813802	6.17E-13
Tvp23a	yellow	2.14244618	3.31E-12	2.31300821	3.78E-13
Cdr2l	red	-0.8648057	2.03E-06	-0.4252627	0.02844283
Ankrd37	orange	1.41242128	2.65E-07	0.50170182	0.10758068
P2ry10	yellow	2.05167698	2.25E-13	2.56931279	1.89E-19
Sgms2	orange	0.67303671	0.09070555	1.14919846	0.00384941
Zfp438	red	-0.7989115	0.00112043	-0.4814359	0.06338336
Amigo1	red	-1.1759541	3.22E-11	-0.9355339	2.99E-07
Zfp169	orange	0.73462413	0.00224884	1.20272081	9.35E-07
Insl6	yellow	2.56644032	2.06E-07	1.20740895	0.01461758
Creg2	yellow	2.56795743	1.12E-05	2.78772528	3.61E-05
Adgb	skyblue	-1.9202762	1.12E-09	-1.0760717	0.00087628
Hs3st1	red	-1.3283363	5.65E-05	-0.7571683	0.02999816
Zfp11	red	-0.7196611	0.00218847	-0.4690746	0.05779656
Olfml1	red	-0.7654514	1.80E-05	-0.6361408	0.00056966
Gprc5c	red	-1.2562549	6.29E-12	-0.5429603	0.00453905
P4ha3	yellow	1.87991776	0.00084658	2.15353953	0.00019387
Lingo3	skyblue	-2.5337424	0.00428409	-2.9516826	0.01396406
Vtcn1	red	-1.114102	0.00679186	-0.1280242	0.79229502
Mblac2	red	-1.0026006	6.05E-06	-1.0116343	1.29E-05
Sv2c	orange	1.68007508	3.73E-06	1.05260059	0.0065167

Gimap9	orange	1.30490175	2.21E-18	1.32859327	4.08E-18
Camk2n2	orange	0.89688257	0.00397082	0.5373572	0.1116838
Nat2	orange	0.66834101	0.00012365	0.29228833	0.12894527
Adnp	orange	0.62984341	0.0700545	0.95259039	0.00646008
Plcb1	red	-0.8127408	0.00035963	-0.9659983	3.15E-05
Gpr183	orange	1.51512964	1.95E-07	1.17868332	0.00010145
Ercc6l	orange	2.04654334	1.26E-08	1.91177303	3.66E-07
Gen1	yellow	2.61282397	1.52E-11	2.16554711	9.34E-08
Cml3	skyblue	-2.1831127	7.63E-08	-1.8385598	9.17E-06
Zgrf1	orange	1.35755152	3.94E-06	1.49919827	8.74E-07
Ffar2	yellow	1.97279571	2.48E-09	2.3515914	1.39E-12
2900026A02I	red	-0.6925137	3.59E-13	-0.54476	2.42E-08
Zfp52	orange	0.78823791	0.00016975	1.08560983	4.29E-07
Samd3	yellow	2.07495952	7.47E-07	2.40709313	9.72E-08
Ncald	red	-0.7913833	5.83E-06	-0.5812478	0.00138981
Six1	red	-1.3426445	2.82E-05	-0.6036668	0.08020208
Ppapdc3	red	-1.3696572	7.08E-10	-0.9175405	0.00014864
Pcdh1	red	-1.1459358	1.45E-14	-0.8870532	6.12E-09
Kif18b	yellow	3.37624757	6.14E-29	3.85836778	6.25E-34
Gm45902	orange	1.21871847	2.96E-06	0.70482503	0.01164619
Tacstd2	orange	1.12884287	7.87E-12	1.25818982	2.62E-14
Plagl2	orange	0.61412823	0.00032478	0.56073719	0.00139081
Ccdc157	red	-0.9358713	1.09E-08	-0.6358493	0.00017787
Fhad1	red	-1.4496291	1.19E-15	-0.8836824	1.84E-06
Cd14	orange	1.00750456	6.25E-05	1.27383182	5.22E-07
Spn	yellow	2.86175075	4.02E-59	2.62820553	1.22E-49
Cbr1	red	-1.0799952	4.83E-08	-0.7512825	0.00025768
Tlr6	yellow	2.46989049	1.30E-09	2.00191213	1.36E-06
Siglech	orange	0.49119086	0.3608591	1.52937626	0.002425
Wdfy4	orange	0.8323446	0.00070863	0.77825928	0.00210403
Fam181b	skyblue	-1.9883379	0.00077136	-1.4755027	0.01175941
Rps19bp1	orange	0.81738112	0.00010276	0.6540217	0.00310896
Zfp579	red	-0.5501697	0.00045626	-0.6298348	8.20E-05
Map3k19	skyblue	-2.1715202	1.92E-11	-1.1710104	0.00030525
Otop1	skyblue	-4.2051852	0.00176225	-3.1415839	0.02497844
2010001K21I	skyblue	-2.1250978	0.00017442	-1.8920479	0.00097718
Rap2a	red	-0.7071452	1.49E-07	-0.4783222	0.00066834
Hist1h1e	red	-1.6745889	0.00318185	-0.4133813	0.52896906
B3gnt2	orange	0.78743419	0.02053634	0.92801658	0.00776292
AU021092	red	-1.2686161	1.10E-07	-1.0634946	1.47E-05
Dcun1d4	red	-0.7790664	5.10E-05	-0.765015	9.47E-05
Trim32	red	-0.711842	2.46E-09	-0.4938627	6.69E-05
Apon	yellow	2.33694848	0.00267178	2.56402159	0.00292525

Rinl	orange	2.01229794	9.87E-67	1.95646134	1.71E-61
Ttn	red	-1.1071645	0.00023457	-1.0259226	0.00092409
Nlgn2	orange	0.65112355	0.00112776	0.55756996	0.00707791
Cox6b2	red	-1.0290755	7.76E-09	-0.8798192	1.85E-06
Mest	red	-0.7722808	0.00042859	-0.8214694	0.00027593
Klhdc1	red	-1.1142461	0.00017709	-1.061741	0.00052673
Cd209f	skyblue	-3.2356413	1.05E-09	-2.20296	5.54E-05
Sox6	red	-1.2408948	5.00E-09	-0.9150943	3.56E-05
Sec31b	red	-0.835492	0.00168292	-0.587092	0.03489359
Lax1	yellow	2.95525219	5.05E-32	2.6256569	5.46E-25
Btla	orange	1.43822911	6.47E-15	1.50932078	8.30E-16
Slc6a7	skyblue	-2.0557878	0.00056196	-1.892869	0.00537694
Pfdn4	orange	0.61274938	0.00441766	0.37107044	0.11371487
Dock8	orange	0.60675772	4.85E-05	0.48954076	0.00157139
Rgs14	yellow	1.82857451	1.89E-13	2.09579222	6.12E-17
Gnpda1	orange	0.88560271	7.11E-11	0.81748197	4.65E-09
Mtcl1	red	-1.4895911	5.21E-08	-1.0136364	0.00036733
D630039A03	red	-1.5041963	2.93E-07	-0.6947195	0.024587
F730043M19	orange	1.75312687	5.05E-13	1.85135785	3.64E-14
Akr1b7	skyblue	-1.4020273	0.07489506	-2.4797932	0.00256066
Foxo6	skyblue	-1.508955	1.80E-06	-0.9001015	0.00714922
Bre	orange	0.61297831	2.00E-05	0.45890551	0.00223711
Rasal3	yellow	2.23822346	1.87E-32	2.37166774	5.80E-36
Gm9869	orange	1.48130595	0.00106169	1.84740996	0.0001387
Pld4	yellow	2.70271858	2.14E-14	2.57359463	6.04E-13
Cd177	orange	1.79437451	2.54E-12	1.82009969	6.54E-13
Ppp1r36	skyblue	-2.1166694	7.58E-09	-1.1245333	0.00293805
Zeb2os	orange	1.65943463	0.00735596	1.40852796	0.03368535
Fpr2	yellow	2.63928936	1.04E-07	2.16782142	2.12E-05
Dnah3	skyblue	-1.8097987	1.33E-06	-0.9261794	0.01867899
Tbc1d30	skyblue	-1.9988784	1.31E-11	-1.5337546	4.15E-07
Hbb-bs	red	-0.4203381	0.28026506	-1.0410674	0.00518063
Lrrc15	skyblue	-2.4037431	0.00273387	-2.2505541	0.00776963
Cx3cr1	orange	0.90258699	0.00840595	0.67466935	0.06376742
Cemip	orange	0.34333821	0.58014076	1.99128155	0.00045706
Tmem106c	red	-0.69127	1.30E-06	-0.5049474	0.00073285
Actn2	skyblue	-1.7294333	1.08E-06	-1.4281042	9.52E-05
Nrros	orange	1.45367981	3.12E-12	1.57749097	4.94E-14
Ccdc171	red	-0.8664713	0.00404172	-0.475654	0.14187557
Tchh	red	-0.8808918	0.0008857	-0.5159686	0.06406441
Bmpr1b	skyblue	-2.009215	4.75E-09	-0.8974353	0.01164445
Cebpe	black	2.60736898	0.00189317	5.0252932	4.63E-05
C130026I21F	orange	1.56503287	0.0175577	1.97900813	0.00217517

Tmem171	yellow	3.20166611	1.17E-13	3.23909586	2.17E-13
Nav2	red	-0.60433	0.00050169	-0.3583064	0.05234431
Robo2	skyblue	-1.6227072	7.28E-11	-1.4247454	2.20E-08
Pbx1	red	-0.597702	7.76E-13	-0.5305078	4.27E-10
Magi3	red	-1.0169068	2.16E-06	-0.5427113	0.01707498
D930048N14	red	-0.7836045	2.49E-07	-0.6560799	2.59E-05
Zmat1	red	-0.7324896	0.00395061	-0.9873541	0.00012353
Rap1b	orange	0.73375695	4.91E-06	0.63838943	0.00011335
Rab7b	orange	1.53627366	4.72E-08	1.17659337	5.34E-05
Tln2	red	-1.2701764	1.43E-08	-1.1189363	1.07E-06
Zfp608	red	-0.5966655	0.00027505	-0.2732108	0.12448022
Gm9888	yellow	1.72287964	0.01324872	2.21819386	0.00907409
Map1b	red	-0.8913376	2.87E-10	-0.6007155	4.18E-05
Klrc2	yellow	2.47742609	5.78E-13	1.76533867	4.09E-07
Swt1	red	-0.3875794	0.03388336	-0.6040454	0.00097882
Trim30b	yellow	3.0091351	6.27E-19	3.67572105	4.92E-27
Gpr25	black	4.57650898	0.00042203	5.4678027	0.00024669
A630001G21	orange	1.96168295	3.42E-16	1.75944861	6.88E-13
Gm9889	orange	1.91883478	9.87E-05	1.46419111	0.00280232
Oas1a	yellow	4.37674319	1.47E-56	3.91980046	1.13E-45
Grk4	red	-0.7579674	0.00333894	-0.5470616	0.04506024
1700030K09I	red	-0.648123	0.00161839	-0.4747901	0.02700274
Nup107	orange	0.70822278	2.54E-09	0.61287307	5.88E-07
Cysltr1	orange	0.90681204	0.00474148	0.0594542	0.88556426
Gm9892	orange	1.93766804	0.00138115	1.24568589	0.06792232
Junb	orange	1.73683658	4.61E-27	1.62197424	1.06E-23
Dnah6	skyblue	-2.616004	6.41E-13	-1.6524593	1.07E-05
Prkcb	yellow	1.95857899	2.58E-20	2.10727015	2.34E-22
Lamb2	red	-1.0155005	1.27E-10	-0.762154	2.85E-06
Cyp2j6	red	-0.7565559	3.61E-11	-0.4341732	0.00032919
Prkg1	red	-1.01334	0.00458736	-0.4759503	0.22102697
Arhgef15	red	-1.1402274	1.82E-11	-0.9904556	1.04E-08
Rnaseh2a	orange	0.60536172	1.38E-05	0.41262104	0.0049842
Glis3	red	-0.8217035	5.90E-06	-0.6359439	0.00056393
Rnf157	orange	0.81654375	0.00018206	0.89433003	5.56E-05
C130021I20F	skyblue	-3.1774043	0.00159377	-1.7186037	0.05251035
Gas1	skyblue	-1.2791752	4.38E-07	-1.2368503	1.94E-06
Cyp2f2	skyblue	-2.7154508	2.21E-08	-1.7222624	0.00067943
Creb5	orange	1.42258276	5.78E-08	1.14553537	2.36E-05
Gm6180	orange	1.27345433	7.92E-05	0.87047598	0.01145892
Cd8b1	yellow	3.0664243	2.31E-06	4.07262997	4.99E-10
Gm15413	yellow	4.56718757	0.00286332	1.60039775	0.23826564
Clec12a	yellow	3.35246752	1.01E-14	2.7712292	4.07E-10

9230110C19	red	-1.1843369	5.23E-05	-0.5600758	0.06895051
2700081O15	red	-1.2768523	1.16E-13	-0.9132832	2.63E-07
Myh7	skyblue	-2.2797102	2.50E-07	-1.5558538	0.00071058
Gpr141	yellow	3.85283327	1.42E-18	3.44116984	3.56E-15
Yap1	red	-0.6954008	1.33E-09	-0.4620165	0.00010662
Fank1	red	-1.3752883	0.00152509	-0.6864366	0.14447516
Socs3	yellow	2.95872179	1.11E-42	3.12391357	1.79E-47
Spag16	skyblue	-3.3032545	3.24E-18	-1.7692245	3.98E-06
Fes	orange	1.4793281	1.47E-10	1.35056889	8.95E-09
Daw1	skyblue	-1.9529148	1.07E-07	-1.2955531	0.00034103
Gpr21	skyblue	-2.8318498	0.00022162	-2.1412515	0.00490485
Cdh22	skyblue	-1.035835	0.00648859	-1.5508351	0.0001065
Bcl3	yellow	2.2213804	3.86E-37	2.35135793	2.56E-41
Arhgap20	red	-1.0066519	0.00111198	-0.6509976	0.04656733
Gm9899	skyblue	-1.8158505	0.00150535	-1.9521556	0.00080752
Dand5	red	-0.7216501	0.00150535	-0.6815928	0.00379678
Aldh1a1	skyblue	-1.9119987	4.00E-08	-1.4753989	4.07E-05
Rab4b	orange	0.67162986	0.00831255	0.63355898	0.01350566
AI854703	red	-1.3002527	0.0092285	-0.5577656	0.2980491
Sec61b	orange	0.76649107	2.52E-07	0.65052487	2.30E-05
Slamf8	black	4.76183887	2.59E-40	4.12691662	5.27E-31
D10Jhu81e	red	-0.8283241	1.15E-05	-0.6430308	0.00103415
Gas5	orange	0.65509537	8.42E-12	0.65195385	2.06E-11
Tarm1	black	7.59837197	2.68E-31	7.37036926	2.03E-27
Phgdh	orange	1.60942322	9.86E-26	1.38530784	4.40E-19
Cbx7	red	-1.0043436	8.68E-06	-0.7359965	0.00169349
Hunk	red	-0.9466119	2.22E-05	-0.7267856	0.00163557
Ggcx	red	-1.1726673	6.81E-08	-0.9204615	3.96E-05
Tg	yellow	2.28710824	4.17E-10	1.54898125	2.02E-05
Lgals7	red	-1.1328781	3.43E-05	-0.6679824	0.02095695
Shisa7	red	-1.4102975	0.00047731	-0.8600494	0.03943999
Ebf4	red	-0.9127064	0.00377115	-0.8027258	0.01342924
3110082I17R	orange	0.76721083	0.00132543	0.59436014	0.0171444
Zfand2a	orange	0.57940157	0.00123757	0.64118821	0.00045706
Gm9913	yellow	1.11238503	0.25408558	5.58535916	0.00036095
Sh3pxd2a	red	-0.8882178	3.61E-09	-0.5615973	0.0003479
Tll1	red	-0.7373825	5.07E-05	-0.9294373	1.40E-06
Dennd4a	orange	1.28715919	1.24E-17	0.99985678	8.12E-11
Aldh7a1	red	-0.7739877	1.93E-10	-0.5457772	1.53E-05
Plxnb1	red	-0.8795639	8.00E-06	-0.5410289	0.00890491
Dpep2	yellow	3.07399093	3.26E-09	1.36272987	0.0081782
Nebl	red	-0.9845488	0.00070435	-0.574602	0.06358522
Ptrh1	orange	0.6136728	0.00784263	0.61576338	0.00934421

1700016K19I	red	-0.9402278	0.00075503	-0.8284324	0.00405753
Grwd1	orange	0.9781743	0.00015185	0.9008554	0.00071927
Camk2d	orange	0.67987456	8.99E-07	0.66366348	2.68E-06
H2-T24	orange	1.64014452	9.01E-30	1.57350044	2.30E-27
Sh2d4a	red	-1.240328	1.54E-13	-0.7099962	5.13E-05
Cyhr1	red	-0.5845103	2.12E-08	-0.4529514	2.60E-05
6330403A02I	red	-1.420103	6.16E-06	-0.8320707	0.00979358
Pde5a	skyblue	-1.9800375	4.67E-17	-1.5578041	9.82E-11
Cd8a	yellow	3.12862238	2.55E-05	4.57682599	7.55E-10
Zfp14	red	-1.3046948	0.00073263	-1.1307664	0.00467031
Tusc1	orange	0.84112529	0.00056369	0.56430968	0.03265677
Ndst1	red	-1.0089642	4.61E-09	-0.5645847	0.00178816
Tmem179	skyblue	-2.4724835	0.00056911	-1.9484368	0.00554033
Sirt5	red	-1.0060186	2.81E-09	-0.8506414	1.33E-06
A930004D18	red	-0.8177099	1.58E-06	-0.732524	4.36E-05
Iigp1	black	4.65996271	1.43E-103	4.38089156	1.07E-91
Skida1	skyblue	-1.6770846	1.48E-06	-1.6207197	5.69E-06
Utp18	orange	0.68261115	4.40E-05	0.61799878	0.00035399
Syne3	orange	1.49599823	2.89E-12	1.71169982	1.98E-15
Fam83e	red	-1.4904602	1.15E-06	-0.6300941	0.05154671
Spock3	skyblue	-2.7094679	0.00207047	-0.7497698	0.40790775
Cthrc1	skyblue	-1.6697651	0.00522518	-1.6373211	0.00867396
Ifi205	black	4.97757774	1.44E-48	4.3151539	2.99E-37
Fgfr3	red	-0.7179799	0.0021677	-0.7152744	0.00287927
Lifr	red	-0.9162339	1.05E-06	-0.787987	4.44E-05
A630033H20	orange	2.25114812	2.95E-06	1.0564822	0.03841051
Lrrc36	skyblue	-2.0368506	2.83E-14	-1.1539851	2.26E-05
Taf4b	orange	1.16423469	5.44E-05	1.0077208	0.0006446
Lce3a	red	-1.1330183	0.00044539	-0.5231953	0.13654275
Kcnn4	yellow	2.91942023	5.20E-19	2.76524601	6.58E-17
Pnma1	red	-1.1682642	0.00016125	-1.0734515	0.00086617
Cklf	orange	1.11345083	4.29E-07	1.05927621	2.75E-06
Slfn5	orange	1.1952114	4.11E-17	1.04109735	5.02E-13
Fabp1	skyblue	-4.9923064	4.66E-08	-4.0239282	7.00E-07
Tmem120b	red	-0.8434118	0.00042228	-0.4491135	0.07882358
Gimap4	orange	1.19260911	2.42E-10	1.04751219	5.34E-08
Vsnl1	skyblue	-1.7203913	3.07E-07	-1.4597649	2.41E-05
Kcnn2	skyblue	-2.8895638	2.49E-08	-2.2114893	1.59E-05
Trim65	red	-0.6961576	1.25E-07	-0.6103912	6.19E-06
Zfp867	red	-0.5079568	0.00851878	-0.6006916	0.00195449
Sh3bp2	yellow	2.52170509	1.35E-20	2.36706407	3.65E-18
Pla2r1	red	-1.2268784	6.59E-09	-0.8887847	4.69E-05
Pabpc1l	yellow	2.11180353	9.30E-05	1.88141603	0.00071314

9130230L23F	skyblue	-2.1193677	5.69E-08	-1.4744105	0.00021492
Mettl7a1	skyblue	-2.5851041	7.41E-06	-1.9729568	0.00097127
Mmrn1	red	-0.6024774	0.00524376	-0.1861994	0.44635124
Pm20d2	red	-1.3798745	0.00286902	-1.0155078	0.03361479
5830411N06	orange	1.11132848	0.13940399	2.02878219	0.0063878
1600014C10	orange	1.53528239	2.54E-10	1.43956055	5.47E-09
Emcn	red	-0.6504428	1.82E-05	-0.7824853	3.90E-07
Ap1s3	orange	0.88112679	5.97E-07	0.59455702	0.00137116
Ankrd24	red	-1.006596	3.54E-09	-0.6987224	6.93E-05
Zfp771	orange	0.32379189	0.1187489	0.68973001	0.00073827
Hmgb2	yellow	2.25675716	6.66E-34	1.87986928	1.43E-23
Vmac	red	-0.8448577	3.87E-07	-0.8777246	2.11E-07
Phactr1	red	-0.7674259	6.34E-05	-0.6705907	0.00069676
Msra	red	-0.6697243	0.0001787	-0.5354666	0.00386162
Fsd1l	skyblue	-1.4925726	3.92E-09	-0.8952629	0.0006849
Set	orange	0.70169024	0.0003193	0.62845001	0.00176618
Cadm4	red	-1.1644704	7.16E-17	-0.765456	1.19E-07
Usp46	red	-1.0678965	2.21E-09	-0.9054197	7.58E-07
Whsc1l1	orange	0.6196177	2.77E-07	0.56532632	4.63E-06
Atrnl1	red	-0.6433339	2.82E-06	-0.4916332	0.00055383
Smim10l2a	skyblue	-1.8202634	0.00010625	-0.8010153	0.08624365
Rnd1	orange	1.41802602	6.63E-08	1.57117868	3.13E-09
Fam19a5	red	-1.4078193	0.00104494	-0.988768	0.02849423
Tmem158	red	-0.7085854	0.00329521	-0.2523968	0.35815108
Dsp	red	-0.7522523	0.0036011	-0.7470848	0.00482563
Txk	yellow	2.10900454	1.19E-10	2.70094645	1.02E-15
Zfp667	red	-1.0546028	0.00239717	-0.9245874	0.01044713
Atp5s	red	-0.6533468	0.00128878	-0.5831471	0.0071974
Zkscan4	skyblue	-1.3720769	1.00E-05	-1.5404156	1.49E-06
Afp	yellow	2.99051289	4.61E-06	2.22686945	0.00054188
Zfp174	red	-0.8492593	0.00280757	-0.7857573	0.00779394
Ifltd1	skyblue	-1.8149203	0.00285356	-2.4246219	8.54E-05
Zfp647	red	-0.8337514	0.0027308	-0.848671	0.00284833
Kbtbd13	skyblue	-2.1212909	4.92E-08	-1.4756719	0.00035693
Sec14l3	skyblue	-2.1282271	2.27E-07	-1.3934635	0.00115959
Agap1	red	-0.890389	3.02E-08	-0.8371058	3.23E-07
Cntn1	skyblue	-2.0566022	0.00064088	-1.9561557	0.00125378
Smyd1	skyblue	-1.5330489	3.43E-09	-1.4541674	4.15E-08
Nfic	red	-0.6351491	2.98E-16	-0.5651473	6.66E-13
A630012P03I	yellow	4.23537484	0.00079246	1.86373177	0.11144424
Arntl	orange	1.67915759	1.10E-08	2.4028926	5.98E-16
Cgrrf1	red	-0.5963993	0.00016692	-0.5622909	0.00055477
Sugct	red	-1.0664244	0.00435798	-0.2475605	0.56829091

Ifng	black	7.54189677	7.57E-38	5.48750246	5.97E-50
C1ra	orange	1.22388096	4.21E-26	1.14546461	8.00E-23
Sertad3	orange	0.67472894	8.38E-05	0.44292547	0.01416842
Zfp811	skyblue	-1.08157	0.04472366	-1.603489	0.00856179
Ankrd17	orange	0.63408052	4.81E-10	0.47979247	5.05E-06
Ntrk2	red	-0.0391632	0.90801939	-0.8155746	0.00454644
Tmem245	red	-0.8848743	8.07E-08	-0.5779071	0.00078131
Zfp93	red	-1.2282641	3.90E-08	-0.7975291	0.00053719
Pgbd1	skyblue	-1.4652672	0.00246743	-1.239815	0.0126856
Gm9967	red	-1.2926763	1.99E-05	-1.1153644	0.00068158
Fat2	red	-1.0399689	8.69E-06	-0.719082	0.00321952
Slc6a2	red	-0.9757784	1.39E-05	-0.7306899	0.00170394
Fut9	skyblue	-3.8745905	0.00121733	-1.1450553	0.26558578
Fbxo6	orange	0.68003287	4.85E-08	0.62269636	1.06E-06
Nell1	skyblue	-3.5399203	0.00141305	-2.6942464	0.00631746
H2-Q5	orange	2.01978825	9.51E-22	1.92591447	9.09E-20
Nap1l5	skyblue	-2.053949	0.00062149	-1.7525832	0.00415976
Maf	orange	0.80559552	9.51E-06	0.54960973	0.00385057
Cd47	orange	0.77759222	1.88E-09	0.47655283	0.00042882
Ano5	red	-1.1393086	0.00358925	-0.9901663	0.01710316
Pprc1	orange	1.12948126	1.39E-06	1.18389636	6.45E-07
Epm2a	skyblue	-1.5805855	0.00010515	-1.3913427	0.0009187
Lair1	yellow	3.93748802	5.98E-27	3.75907556	1.64E-24
B4galnt4	black	5.17885595	2.54E-41	4.96742931	2.78E-37
Hmcn2	skyblue	-1.9113282	1.68E-14	-1.1076006	2.19E-05
Dach1	red	-0.730523	0.00049966	-0.547335	0.01239773
Gpc3	red	-1.2338968	1.14E-10	-0.9406447	1.86E-06
Kbtbd11	orange	1.16577522	3.93E-09	0.75393465	0.0002565
Gm5069	skyblue	-1.6676344	0.00336907	-1.3365928	0.02097285
Klhl26	red	-0.6971169	2.35E-07	-0.4434026	0.00152213
Slain1	orange	1.20336539	9.40E-08	1.18140648	3.70E-07
Nap1l3	skyblue	-2.9061344	1.90E-09	-1.8325472	0.00011548
Ghr	red	-0.4791668	0.00054093	-0.5944465	2.28E-05
Ldoc1l	red	-0.6875427	2.58E-06	-0.447758	0.00366493
Gemin6	orange	0.85742036	6.00E-05	0.70779105	0.00175987
Abcd2	red	-0.7529987	8.60E-06	-0.6823833	8.36E-05
Tcf7l1	red	-0.6063894	4.18E-10	-0.2158552	0.03769348
Fmnl1	yellow	2.19261354	2.43E-24	2.1474274	2.85E-23
Mta3	orange	0.88267894	7.96E-09	0.70044642	9.07E-06
Zfp1	orange	0.87030439	6.65E-08	0.53950439	0.00144965
Tceb2	orange	0.7368553	9.09E-05	0.51740003	0.00865467
Izumo4	red	-0.9491838	8.04E-05	-0.6527564	0.00967845
Fam19a3	orange	0.39342624	0.26628784	1.05096247	0.00378968

Per2	red	-0.8294616	0.00035707	-0.8888616	0.00017527
Fancm	orange	0.93515874	1.40E-07	0.5103102	0.00605938
Tmem150a	red	-0.7315952	0.00014992	-0.6848454	0.00055362
Aasdh	red	-0.6024483	3.19E-05	-0.4969687	0.00102773
Fto	red	-0.6473403	3.96E-12	-0.4127099	2.13E-05
Prr18	skyblue	-2.2097609	1.60E-14	-1.1526843	0.00011081
Cldn23	red	-1.033919	8.68E-05	-0.7548612	0.00697197
Irs1	skyblue	-1.9309843	4.42E-05	-1.0693347	0.0324293
Nod2	orange	1.42019105	3.25E-07	1.59354356	1.62E-08
A430033K04I	red	-0.8283271	0.00011233	-0.517429	0.02130096
Clca3a1	black	4.67285332	1.67E-80	3.64001212	6.02E-51
S100a8	yellow	2.7145452	2.88E-09	2.06507843	8.61E-06
Fam105a	orange	1.42059765	6.58E-24	1.01415876	1.79E-12
S100a9	yellow	2.44496454	9.21E-09	2.08423116	1.35E-06
Grik2	skyblue	-1.3826581	0.00400022	-1.9527167	0.0002723
Eif3b	orange	0.53882742	0.00023991	0.6029191	5.41E-05
H2-T22	yellow	2.73674849	1.51E-79	2.43467698	4.48E-63
Trim34a	orange	1.15556555	5.52E-17	1.19691595	6.51E-18
AI504432	orange	1.27632203	1.27E-17	1.55247782	7.58E-25
Col8a2	skyblue	-2.2292335	2.11E-13	-1.1962591	0.00013283
Cfl1	orange	1.33224586	2.31E-09	1.22378241	7.30E-08
Pgpep1	red	-0.7502375	3.04E-13	-0.6391939	1.21E-09
Npm3	orange	0.95976539	4.90E-09	0.80632536	2.00E-06
Pard6g	red	-0.5835074	3.04E-05	-0.2977646	0.04610472
Lrguk	skyblue	-2.2603059	1.06E-13	-1.317793	1.23E-05
Cep70	red	-0.6162926	0.00188125	-0.4962899	0.0155742
Dennd1b	orange	1.00317451	1.36E-11	0.92925416	7.37E-10
Ms4a4b	yellow	4.33613292	1.52E-49	3.83525876	1.26E-38
1810011O10	red	-0.9054883	1.03E-09	-1.212255	2.04E-16
Sftpb	red	-0.9650912	0.00194604	-0.4902141	0.14613751
Lig1	orange	1.93362534	1.12E-21	1.55994263	3.28E-14
Adap1	yellow	2.26109177	2.18E-21	2.05139816	1.39E-17
Hoxaas2	red	-0.7376915	0.01470783	-0.9429652	0.00228206
Zbtb25	orange	0.70815969	0.00090397	0.50678329	0.02342727
Med12l	orange	0.95593391	0.00672326	1.52918386	1.82E-05
Chn1	red	-0.7174334	0.00018975	-0.8292051	2.18E-05
Mettl7a2	skyblue	-2.5557775	0.00541145	-1.7157881	0.06270857
Cngb3	black	7.05226258	2.58E-07	3.86598959	1.80E-06
Tmem154	orange	0.58261196	0.00280574	0.46621809	0.02163518
Cebpb	orange	1.21863035	4.20E-05	1.42999377	2.22E-06
Ptafr	yellow	2.7819547	3.92E-12	2.75566921	9.34E-12
Ccdc18	orange	1.61617344	0.00661394	2.13917737	0.00057228
Pign	orange	0.89202673	4.18E-06	0.74817776	0.00018328

Mpz	skyblue	-1.9749539	0.00096383	-0.8733074	0.18194684
Zfp658	red	-1.1859219	2.51E-05	-0.9453678	0.00102239
Trnp1	red	-0.775895	0.00417775	-0.6578607	0.01942078
Lrrc48	skyblue	-1.9519317	2.76E-11	-1.1116841	0.00026473
Fry	red	-0.5076818	0.00038204	-0.7241957	4.48E-07
Ppp1r14b	orange	0.56929912	0.00064997	0.7453543	1.05E-05
Apol8	black	4.77574003	2.93E-06	4.32815184	5.12E-06
Retsat	red	-0.6651878	0.00034213	-0.4712159	0.01552295
Ier5	orange	0.82450197	0.0004677	0.85823273	0.0003675
Nbeal2	orange	0.88702325	4.72E-14	0.86432962	3.10E-13
Capg	orange	1.89963584	1.04E-10	1.67225904	2.51E-08
Nfil3	yellow	3.04380501	3.66E-21	2.69468727	7.36E-17
Dnah9	skyblue	-2.6110799	6.89E-15	-1.3689196	7.88E-05
Hmga2	yellow	4.03456755	0.00897154	3.55464553	0.01933886
Zfp663	skyblue	-3.9090195	0.00010674	-2.3677978	0.00715891
Ttc26	red	-1.2404398	1.02E-07	-0.8465282	0.00047127
Gulp1	red	-1.2026897	8.08E-07	-1.1885016	1.85E-06
Glipr1	orange	1.82074791	2.90E-10	1.19256896	7.80E-05
Immp2l	red	-1.0547361	0.00613438	-0.7583777	0.06191501
Usp13	red	-1.4720208	7.98E-06	-1.0390375	0.00256339
Acbd4	red	-0.8895817	1.76E-05	-0.69342	0.00122317
Tatdn2	orange	0.9211726	4.50E-09	0.90199475	1.54E-08
Ces1d	skyblue	-1.9054646	4.86E-08	-1.3420763	0.00021492
Myh4	skyblue	-2.0523573	0.00033411	-0.6857838	0.28410633
Gm7536	orange	0.60272504	0.00661479	0.42047752	0.08225667
Skap1	yellow	2.81795119	1.79E-16	3.04001612	2.73E-18
Slc35f3	skyblue	-2.8944315	6.71E-07	-1.4690907	0.00882692
Fam47e	skyblue	-2.0686295	5.75E-12	-1.4359846	3.13E-06
Ces1g	skyblue	-2.9967491	1.39E-10	-3.1883695	1.42E-11
C030039L03I	red	-1.2083634	0.00085505	-1.1410042	0.00213149
Ebf1	red	-0.8861635	7.18E-09	-0.7069888	7.98E-06
Cntrl	orange	0.92444171	3.48E-11	1.01167824	5.30E-13
Npm1	orange	0.93677111	1.76E-09	0.6486159	5.97E-05
Gja5	red	-1.2122782	1.46E-05	-0.9956092	0.00058571
Scimp	yellow	4.17893633	3.67E-30	3.8839408	1.62E-25
Tmem140	orange	1.09458153	4.71E-14	1.27061684	1.51E-18
Trim12c	orange	1.34521508	1.10E-37	1.38566989	1.52E-39
Gm166	red	-0.8336537	0.0002183	-0.5513176	0.01815576
Scn3a	skyblue	-2.4124754	5.88E-12	-1.3273051	0.00028568
AB124611	yellow	3.23846146	4.97E-30	3.12373297	1.17E-27
Armc7	orange	1.25120961	3.88E-09	1.33056296	7.18E-10
BC051142	orange	1.16563498	0.00038277	0.74595224	0.0233718
Ccdc176	skyblue	-1.7862888	3.16E-06	-1.2947108	0.00102341

St6galnac2	red	-1.1227037	0.00039646	-0.9662015	0.00313139
Rpl38	orange	0.66123219	3.52E-06	0.6050389	3.64E-05
Cep170	orange	0.61344733	6.64E-06	0.58889451	2.49E-05
Chst3	red	-1.122864	1.84E-05	-0.6386365	0.02137784
Apol9a	black	5.26608437	1.06E-75	5.11790653	3.33E-74
Uxs1	orange	0.62176852	0.0027478	0.52779896	0.01445085
Birc2	orange	0.64573847	1.10E-08	0.40936694	0.00053239
Ryr3	skyblue	-1.3172185	0.00080978	-1.2123613	0.00329615
Zfp759	red	-0.9475656	0.00135397	-0.917721	0.00307281
Whsc1	orange	1.42881031	8.09E-14	1.16699147	2.25E-09
Zfp53	orange	0.61931254	0.00155527	0.50487122	0.01399719
Mpp7	red	-1.1116452	3.16E-10	-0.6507029	0.00042637
Phex	red	-1.178997	8.54E-07	-1.1174087	6.84E-06
Saa2	black	5.2057052	2.61E-05	3.90352845	1.07E-05
Bloc1s2	orange	0.71666455	0.00038578	0.61201218	0.00349346
Dtnbp1	orange	0.79364619	0.00047673	0.71104284	0.00245067
Lgals8	orange	0.64915032	8.83E-07	0.66551066	7.50E-07
Eif1a	orange	0.9855768	2.32E-07	0.87140786	8.36E-06
Trim30d	yellow	3.07390053	7.24E-52	2.6828	7.47E-40
Colq	skyblue	-3.3065538	3.21E-05	-1.3891608	0.10737081
Lce1a1	red	-1.1541695	0.00021057	-0.7846429	0.01663218
Gnai1	red	-0.6524575	4.23E-08	-0.5786381	2.14E-06
Gapdh	orange	0.65723485	0.00012423	0.64493646	0.00024043
Mex3b	red	-1.0303311	8.95E-06	-0.8550328	0.00041205
A830018L16I	skyblue	-3.1912603	1.81E-07	-1.008242	0.07335411
Tmem178b	skyblue	-2.4013683	0.0005509	-1.7332996	0.01405519
Sh3rf2	skyblue	-1.4728744	1.46E-14	-0.8393428	2.62E-05
Lepr	red	-1.0678494	1.36E-05	-0.9561989	0.00015057
Prtn3	orange	0.42466421	0.39665542	1.3727244	0.00371368
Megf6	skyblue	-1.7167327	2.14E-08	-1.3112737	3.43E-05
Cyb5d2	red	-0.6046347	0.00551045	-0.8046685	0.0002946
Ddx49	orange	0.64780016	2.50E-05	0.58948059	0.0001936
Bak1	orange	1.89362321	8.99E-14	1.7436156	1.24E-11
1700007G11	skyblue	-2.2380728	1.04E-12	-1.6195318	1.45E-07
Rpl32	orange	0.89797531	5.80E-12	0.71823885	8.04E-08
Gm10032	skyblue	-2.9036781	0.0015205	-1.2661773	0.15066306
Cacnb2	skyblue	-1.343359	7.85E-05	-1.2224576	0.00044919
Gsta2	skyblue	-1.6356046	0.00019289	-0.8804325	0.05554745
Unc13d	orange	0.65749389	0.00019079	0.74837618	2.97E-05
Fgf18	red	-1.3976021	7.44E-05	-0.6363309	0.0973092
Sema3b	red	-0.9312172	1.23E-05	-0.3783725	0.10273263
Mdn1	orange	0.53690159	0.02264313	0.62358326	0.00921886
Adtrp	red	-1.4485231	0.00300522	-1.0119399	0.04996651

Palld	red	-0.7577293	9.40E-06	-0.6724586	0.00013399
Mettl7a3	skyblue	-2.2729888	0.00158401	-1.8118484	0.01056405
Eml1	red	-1.2339013	1.04E-22	-0.960127	5.66E-14
Nfam1	orange	1.2424166	1.49E-05	1.24341479	2.27E-05
Tpm3-rs7	orange	1.30831246	1.04E-07	1.04998674	5.29E-05
Gstm1	skyblue	-1.712035	1.59E-06	-1.2537959	0.00071414
Chsy3	orange	0.89105691	0.05285676	1.50472264	0.00093153
T2	skyblue	-2.1282291	2.88E-06	-1.3888551	0.00153522
Gm5431	yellow	4.02681504	2.08E-34	4.11678381	3.41E-35
Gm5148	skyblue	-1.5878096	0.00074678	-1.187897	0.01169121
Zfp846	red	-0.8185226	0.00064111	-0.6413154	0.0105918
BC021614	orange	1.97399083	2.97E-14	1.66279567	1.07E-09
Arhgap35	red	-0.5803668	1.04E-05	-0.3172462	0.02299474
Tspan7	red	-1.0833615	2.40E-11	-0.9206765	2.78E-08
Serpina9	skyblue	-2.1465031	9.64E-06	-1.8065185	0.00027618
Espl1	yellow	2.7519599	4.24E-31	2.81801225	1.04E-31
Spock2	red	-0.6655329	0.00038935	-0.2208099	0.29313145
Mcm9	orange	0.79045572	1.58E-07	0.73582097	1.97E-06
Rrp1b	orange	0.68751353	3.38E-06	0.5037689	0.00105698
Gpr182	red	-0.6713607	0.00039351	-0.4485799	0.02422335
Zfp420	red	-0.9510196	5.23E-05	-0.618861	0.01125055
Syt17	skyblue	-1.9543803	1.81E-07	-1.8369526	1.01E-06
Cxcl2	black	6.8084163	1.70E-17	5.44733173	5.10E-25
Gm8369	yellow	2.96750908	7.96E-27	2.69576296	8.14E-22
Rnf207	skyblue	-1.3982424	0.17110216	-2.6871113	0.00737751
Gpc6	skyblue	-2.4717953	3.43E-09	-1.2360933	0.00297323
Gda	orange	2.02190091	2.39E-10	1.91765676	3.26E-09
Samd12	skyblue	-1.792066	5.08E-20	-1.7936017	8.39E-20
Tubb2a	orange	0.67653076	5.89E-05	0.71885061	2.88E-05
Memo1	orange	0.68705083	3.06E-05	0.53630742	0.00176846
Fcer1g	yellow	3.87858795	2.81E-35	3.38526169	6.01E-27
Lin9	orange	0.71768722	0.00246458	0.48494621	0.05250404
Kcnt1	black	5.37634923	4.66E-21	3.75050544	8.93E-15
Osm	yellow	3.59207214	7.62E-07	2.80700845	0.00015011
Thra	red	-0.8067878	6.17E-06	-0.5056219	0.00694634
Rnf169	orange	0.67762442	5.17E-06	0.52176728	0.00073045
Hist1h1b	yellow	2.12785542	0.06342852	4.74187068	0.0032624
Nfe2	orange	0.65912658	0.0081526	0.59164084	0.0229209
0610039K10I	yellow	3.50534238	1.28E-07	1.54968036	0.00404866
Pirb	yellow	3.28289702	4.27E-25	3.16703588	2.32E-23
C1qtnf3	skyblue	-3.2148915	3.10E-07	-1.4442326	0.02181117
Slc10a5	skyblue	-3.3543606	8.60E-10	-1.2192768	0.01505384
Gm10052	orange	0.65401261	0.00823149	0.49225224	0.06187594

Vwa8	red	-0.6502763	1.03E-05	-0.5949691	8.45E-05
Hnrnpa3	orange	0.7729625	1.97E-06	0.6967237	2.98E-05
Frem1	red	-0.6293891	0.00270774	-0.4810406	0.02943806
Rpl18	orange	0.79724269	2.51E-06	0.55922243	0.00152349
Fcgr4	black	6.16121938	1.24E-60	5.26579317	1.04E-45
Ifitm6	yellow	2.26037381	1.36E-05	3.1189551	8.28E-09
Ntrk3	skyblue	-2.1453223	1.05E-11	-1.0278415	0.00163456
Mtfmt	orange	0.6067103	1.27E-07	0.38060912	0.00156484
Fam19a1	skyblue	-2.3858803	8.10E-05	-1.6789485	0.00685876
Gm12715	orange	0.80903584	0.00955881	0.76396207	0.01769469
Lep	skyblue	-1.8078355	0.04006734	-2.6329559	0.00176231
Sept9	orange	0.83111716	2.70E-05	0.62518954	0.00237671
R74862	red	-1.0604182	0.00962428	-0.6401024	0.13502905
Rpl11	orange	0.71825311	2.59E-08	0.48693387	0.00028871
Tonsl	orange	1.22564025	2.52E-09	1.11643668	1.26E-07
Hopx	red	-0.7396052	0.00323696	-0.4917737	0.06442714
Csf2ra	yellow	2.22858525	6.39E-11	2.14429617	5.59E-10
Slc14a1	orange	1.59630252	0.0011563	1.58758698	0.00198253
Fxn	orange	0.52355659	0.01924618	0.74247572	0.00114513
Mamld1	red	-0.5910939	0.00224114	-0.6150827	0.00163904
Fxyd2	skyblue	-2.1780728	0.00027146	0.71689553	0.22598838
Actg2	red	-1.059682	1.58E-06	-0.0900532	0.74534483
Bcas3	red	-0.4503377	0.00417432	-0.5825715	0.00025189
Hadhb	red	-0.6094015	0.00013246	-0.5905637	0.0003166
Ptk2b	yellow	2.1888967	4.41E-20	1.94251166	7.64E-16
B3gnt8	orange	0.82693206	0.00751121	0.44615941	0.18464314
Fcgr3	yellow	3.25014274	6.83E-21	2.69290152	1.60E-14
Znhit1	orange	0.66015534	3.69E-05	0.21670667	0.22670195
Ccdc154	skyblue	-2.0853264	1.34E-11	-2.0966891	2.17E-11
Nsmce2	orange	0.64361287	1.04E-05	0.44557977	0.00343857
Calcrl	red	-1.2284647	1.59E-07	-1.0208317	2.32E-05
Stfa2l1	black	5.55557938	5.57E-05	3.54578724	0.00043052
Cdh24	orange	0.50802714	0.04076138	1.14244133	3.32E-06
Zfp637	red	-0.6440316	1.72E-06	-0.3822457	0.00676611
Myl3	red	-1.2906703	0.00026905	-0.8141222	0.02971329
Slx1b	red	-0.7609134	1.45E-06	-0.705804	1.39E-05
Nrm	orange	1.04719462	1.04E-09	0.84248955	2.03E-06
Eif4a1	orange	0.92887419	7.84E-09	0.91326524	2.30E-08
Rgs3	red	-1.0119701	2.40E-22	-0.9806206	6.81E-21
Dbp	skyblue	-3.2953509	1.80E-17	-2.4267468	9.62E-10
Zfp874b	red	-0.9050095	7.65E-05	-0.7360957	0.00167943
Hydin	skyblue	-3.005779	2.68E-19	-1.351143	6.82E-05
Tnip2	orange	0.74971756	8.79E-08	0.60861253	2.48E-05

Irak4	orange	0.84913377	1.18E-09	0.67538297	2.84E-06
Ptp4a3	red	-0.5828124	0.00192686	-0.6267457	0.0010895
Dsc3	red	-0.8608399	0.09048628	-1.3563409	0.00759392
Adamts14	orange	1.73703726	6.94E-20	1.19133229	8.32E-10
Grb2	orange	0.68167459	0.00015228	0.58837535	0.00154658
Hspa2	red	-0.8795763	8.67E-08	-0.8136927	1.29E-06
Chpt1	red	-1.241715	2.32E-08	-1.1268963	7.10E-07
Alox5ap	orange	1.84705118	1.04E-06	1.66824696	1.73E-05
Psma3	orange	0.80836485	2.84E-08	0.63069876	2.75E-05
Atp8b4	yellow	3.76334193	9.37E-28	2.69304265	1.08E-14
Kif27	skyblue	-2.1191592	1.63E-11	-0.8609572	0.00844742
Slc35e3	red	-1.0937254	3.64E-10	-0.8422738	2.65E-06
Cxcl11	black	7.10711772	7.19E-36	11.0296358	5.08E-18
Lrrc10	skyblue	-2.4002192	0.0182309	-3.6149181	0.00044798
Arrb2	yellow	2.41707337	3.95E-18	2.03461128	6.22E-13
Casc4	red	-0.9331476	3.26E-07	-0.7565162	6.11E-05
Gtf2i	red	-0.9468754	4.62E-10	-0.8381647	6.51E-08
Gm1661	skyblue	-2.2414032	5.81E-10	-0.9227294	0.0078272
Ppih	orange	1.19784082	2.34E-08	1.05092683	2.11E-06
Bckdha	red	-0.9651532	0.00179062	-0.8212019	0.01030756
Zfp128	red	-0.597235	0.06099452	-0.8244551	0.00939572
Chst8	skyblue	-2.4264358	2.47E-23	-1.322739	6.25E-08
Sntb1	red	-0.9527311	0.00013493	-0.6952805	0.00749588
Trim5	orange	1.00404593	5.82E-08	0.88787409	3.56E-06
Adgrg3	orange	1.11244196	3.73E-06	1.10495568	6.41E-06
Irak2	orange	0.70146965	2.76E-05	0.76259917	7.57E-06
Xcr1	orange	0.98746712	0.02001931	1.19352445	0.00765841
Zfp266	red	-0.844718	5.46E-06	-0.6241302	0.0012289
0610040J01F	red	-0.6685806	0.00157199	-0.0840337	0.74803037
Tor3a	orange	1.10527259	2.75E-19	0.88414059	1.57E-12
Tmem219	orange	1.11521699	2.52E-07	0.90326984	5.38E-05
Tnfrsf19	red	-1.3189935	8.23E-16	-1.1370785	8.60E-12
H2-Q9	yellow	2.33152836	4.63E-35	2.02443009	1.51E-26
Mfap2	red	-0.6644024	0.00381938	-0.5913049	0.0127308
H2-Eb1	orange	1.4655747	2.90E-21	1.11223461	1.92E-12
Ifitm2	orange	0.79222983	0.00123382	0.48853352	0.06102192
Layn	red	-1.0187379	4.85E-08	-1.0581655	2.85E-08
Eno3	skyblue	-1.5269684	2.50E-07	-1.1606054	0.00015395
Nkpd1	red	-1.0572154	0.00769026	-0.9199095	0.02508251
Hist1h4i	orange	1.9921715	2.39E-08	1.44829209	0.00010875
Atp8b2	orange	0.74055636	2.60E-08	0.70343896	2.16E-07
Pla2g16	orange	0.7962025	6.82E-05	0.55322732	0.0081782
Slc9a6	red	-0.8319887	2.21E-11	-0.8353892	3.31E-11

Plekhh1	red	-0.9909489	2.98E-05	-0.7436884	0.00236997
Nsa2	orange	0.85379949	4.64E-07	0.58044999	0.00104115
Tsga10	red	-1.0570985	1.24E-05	-1.2197184	6.47E-07
Gmfg	orange	1.24048129	2.14E-10	1.16806988	4.60E-09
Intu	red	-1.0919233	0.0008979	-0.1887283	0.59594054
B2m	yellow	2.1224356	6.03E-35	1.8171023	9.72E-26
Ube2s	orange	0.79695435	2.66E-09	0.836449	7.87E-10
Kcnd2	skyblue	-2.4724546	0.00754156	-0.8610173	0.38931766
Trim55	skyblue	-1.5651085	0.00030947	-2.024154	6.45E-06
Acyp2	red	-0.6361981	0.00761572	-0.3212668	0.21942719
Trmt61a	orange	1.21863175	4.23E-05	0.99470456	0.00127863
Slc4a4	red	-1.2134127	6.56E-10	-0.6098157	0.00300698
Hist1h4h	skyblue	-1.6391151	0.00050181	-1.3957316	0.00516824
Rrs1	orange	0.77294515	0.00030947	0.84776326	0.00010854
Olfr920	orange	1.4349365	1.75E-06	0.84712013	0.0050985
Cdh3	orange	0.31101385	0.29405691	0.91343186	0.00166143
Mcpt4	skyblue	-3.0148682	1.04E-08	-1.6245898	0.00077515
Lsamp	skyblue	-1.9836504	2.36E-06	-1.7207087	6.27E-05
Retnla	yellow	3.71497589	1.76E-47	3.98465882	1.85E-54
Prpf40a	orange	0.71963739	5.33E-09	0.40319216	0.0019182
Slc38a11	skyblue	-1.1376972	0.08431463	-1.9397469	0.00368655
Fnip2	orange	0.82676088	0.0001149	0.85506688	9.37E-05
Stk19	orange	0.94589731	1.38E-19	0.85670879	4.76E-16
H2-K1	yellow	2.15300382	1.73E-39	1.86684298	8.48E-30
Mmgt1	red	-0.6150325	2.53E-07	-0.6591831	5.90E-08
Dnaic1	skyblue	-2.8129908	6.71E-14	-1.7798085	2.71E-06
Hipk2	orange	0.96506996	6.42E-09	0.91963267	6.22E-08
Nol10	orange	0.93949018	2.78E-06	0.83670696	5.37E-05
Obscn	red	-0.7182117	0.00280628	-0.9099217	0.00018792
Snrpa	orange	0.58664606	5.65E-05	0.45070869	0.0029951
Krt5	red	-1.0441023	0.00031442	-1.093508	0.0002239
C1qtnf7	skyblue	-1.606313	9.04E-16	-1.300669	1.59E-10
Orm2	yellow	3.18420178	0.00036803	2.4833366	0.00365429
Zfp229	red	-0.7776462	4.19E-05	-0.7527039	9.34E-05
Dpp6	skyblue	-1.974505	0.0002884	-2.3757932	9.91E-06
Adgrg5	yellow	3.05144222	4.43E-21	2.58819012	2.38E-15
Dot1l	orange	0.70365415	9.51E-05	0.74201497	5.45E-05
Pclo	skyblue	-2.524526	7.89E-09	-1.2569626	0.00340429
Akap6	skyblue	-1.8796335	1.95E-05	-1.6928186	0.00019847
U2af1	orange	0.74172494	3.53E-12	0.57091628	2.04E-07
Gdpd1	red	-0.8728976	0.00012908	-0.7881253	0.00076295
Ppp1r1b	red	-1.1672898	0.00255177	-0.6918277	0.09288096
Tnnt3	red	-1.3594241	0.00019508	-0.8899226	0.02049839

Cyp2d22	red	-0.9052559	0.00176157	-0.7807072	0.00905827
Kalrn	orange	0.36457296	0.18665087	0.82323034	0.00198628
Cfd	skyblue	-2.6495823	0.00415584	-0.9448003	0.36162571
Armc4	skyblue	-2.1136939	5.93E-10	-1.178448	0.00053272
Myl1	skyblue	-2.1153784	0.00039439	-1.2400853	0.05107575
Inadl	red	-0.8890769	0.00142225	-0.5525724	0.06177605
Sphk1	orange	1.0242907	5.29E-09	0.74407747	4.14E-05
Ccdc62	red	-0.5005652	0.1136592	-1.1185277	0.00027003
Ces1e	skyblue	-2.6495752	0.00090125	-1.5832877	0.06086648
Olfr99	orange	1.766216	0.00447047	1.87364525	0.00615406
Rpl34	orange	0.59226243	1.68E-06	0.43801415	0.00065804
Hsh2d	yellow	3.2966961	4.25E-27	2.94639149	7.28E-22
Zfp13	red	-0.6402083	4.68E-05	-0.3931325	0.01625994
Lmtk3	orange	1.48951768	0.00046289	1.7965186	3.98E-05
Pgk1	orange	0.78026689	1.52E-06	0.61744472	0.00023764
Lmnb2	orange	0.52335231	0.00031915	0.60525945	4.40E-05
Trim54	red	-1.1128556	8.13E-05	-1.0597926	0.00028881
Cd200r4	yellow	2.50278422	1.01E-07	1.82892187	0.00017085
Btbd3	red	-1.3216074	8.55E-07	-0.9852926	0.00039429
Ear6	black	5.42330653	0.00758429	4.03222902	0.06130152
Hs6st2	skyblue	-2.4677368	5.48E-17	-1.7829975	8.10E-11
Erbb4	skyblue	-0.6721033	0.46629474	-2.389736	0.00723924
Tnfaip8	orange	0.89770828	5.73E-07	0.77763381	2.61E-05
Cks2	yellow	2.23780546	4.53E-10	2.36917153	1.16E-10
Opcml	skyblue	-2.15113	4.91E-05	-2.0644915	0.00021455
Pvrl2	orange	0.57155321	0.00206871	0.58156401	0.00220992
Rpp25	skyblue	-0.8694746	0.18135773	-1.9922202	0.00265786
Gm10115	skyblue	-0.5907572	0.4747596	-2.6935956	0.00231307
Rpl17	orange	0.59772429	2.89E-06	0.38048879	0.00448878
Cytl1	skyblue	-1.64198	0.00011209	-1.8019537	3.24E-05
Itgb1bp1	orange	0.60400322	1.79E-05	0.22827892	0.13943543
Tubb3	yellow	1.80632927	1.29E-16	2.03364366	7.49E-20
Arf2	orange	0.62331925	0.00128506	0.56523634	0.00467293
Fbxl12os	red	-1.5092706	0.00084952	-0.6265255	0.20941628
Ifit3b	yellow	3.14871876	3.68E-70	3.21082101	1.06E-72
Nsl1	yellow	2.00328061	2.16E-09	2.10182914	1.33E-09
Fabp4	skyblue	-2.3508309	0.00133051	-1.4641832	0.05984284
Ncr1	orange	0.8012035	0.03446374	1.83367291	1.57E-06
Cys1	skyblue	-2.2261053	2.48E-09	-1.8270628	1.85E-06
Tubb4a	skyblue	-1.6006588	6.88E-10	-0.9519683	0.00043241
Rpl7a	orange	0.70342858	4.81E-07	0.59181259	3.91E-05
Ncs1	red	-0.9399448	3.79E-05	-0.6219852	0.00904897
Cav3	red	-0.9507723	0.0005573	-0.1564018	0.64140601

Sim2	red	-1.0080941	0.00061303	-0.5289054	0.09623452
AI413582	orange	1.02928103	3.55E-11	0.93519847	5.82E-09
1810041L15F	skyblue	-2.1218204	1.48E-07	-1.2290095	0.00314116
Chia1	red	-0.8520013	0.01022134	-0.9598653	0.00437002
Csprs	black	5.83772344	0.00018032	1.69304502	0.12732344
Kcnc3	red	-0.934094	0.00054948	-0.4714874	0.10608427
Zfp599	orange	0.72873798	0.07412258	1.12061976	0.00696018
Actg1	orange	0.8973618	5.45E-05	0.91871893	5.23E-05
Tcp11	skyblue	-1.5969726	1.28E-14	-1.079383	2.53E-07
Phactr2	red	-0.989843	7.12E-08	-0.7123866	0.00018528
Rpl31-ps11	orange	1.0529478	0.00713124	-0.0234819	0.96273917
Klhl24	red	-1.2067429	5.07E-05	-0.9932202	0.00125425
Hdac10	red	-0.8229181	1.74E-07	-0.5002108	0.00237678
Acadm	red	-1.1645201	9.68E-08	-1.026644	4.61E-06
Stat4	yellow	3.43732107	1.40E-38	3.39636002	6.91E-34
Kdr	red	-1.4890549	1.87E-19	-1.0612685	3.74E-10
Cped1	skyblue	-1.9188074	4.10E-07	-1.299831	0.00098183
Rpl35	orange	0.77957903	2.64E-05	0.47402192	0.01538985
Msln	orange	1.33648183	0.00011148	1.25465932	0.0004156
Sox7	skyblue	-1.5808535	3.98E-45	-1.4050793	9.68E-36
Mapk3	red	-0.6574415	3.85E-12	-0.6629054	4.09E-12
Calml3	red	-0.9618875	0.00225869	-0.7918767	0.01562405
Kcnma1	red	-1.4146659	0.00097913	-0.8780568	0.04485596
Clip2	orange	0.64540521	0.00386635	0.61760071	0.00717486
Hspb11	red	-0.7707903	2.05E-05	-0.1385919	0.51696141
Cd300lb	orange	1.18072337	0.00053085	0.97083338	0.00593783
Ldha	orange	0.70675315	2.75E-05	0.57482699	0.00097969
Gpr84	black	8.31360228	2.63E-09	7.08710103	7.67E-07
Grm4	skyblue	-2.8968337	5.72E-05	-1.9766093	0.00298476
Zfp993	yellow	2.35530788	0.00022186	1.56616192	0.02049127
Parp10	yellow	2.30860815	9.79E-106	2.24382142	2.88E-100
Naa15	orange	0.74292991	6.26E-07	0.43254762	0.00598871
Gm8995	yellow	2.81665576	1.75E-65	2.68427341	3.91E-59
Tmem117	red	-1.1600966	0.00205049	-0.4754297	0.24777384
Rpl27	orange	0.92032193	2.16E-09	0.71517665	6.40E-06
Slc39a4	red	-0.6834066	2.66E-05	-0.4627631	0.00647964
BC023105	black	5.0951919	7.04E-10	5.19892408	4.50E-10
Tmed5	orange	0.77087941	2.08E-06	0.49612441	0.00362599
Lrrc43	skyblue	-1.7097027	1.28E-06	-0.8017605	0.02912478
Gm10131	orange	2.06371598	0.00944699	1.12031896	0.18286968
Ddo	skyblue	-2.0629745	1.41E-09	-1.6112602	3.96E-06
Wscd2	red	-0.7963098	1.69E-05	-0.1720354	0.40885664
B9d2	orange	0.63182161	0.00023254	0.63283082	0.00034625

Nmral1	orange	1.67589102	1.06E-10	1.89633216	8.61E-13
Syne2	red	-1.160494	8.54E-08	-0.8803331	8.54E-05
D630045J12I	red	-1.084945	1.83E-05	-0.9591973	0.00021758
Zkscan7	red	-0.9309931	0.00049695	-0.8063474	0.00335989
Eno1	orange	1.09222418	2.26E-07	0.82901375	0.00014713
Stmnd1	skyblue	-3.244975	5.20E-20	-1.9992514	8.19E-09
Sema3e	skyblue	-1.4936342	9.15E-08	-0.8198236	0.0053692
Aox1	red	-1.1641473	8.46E-07	-0.6394246	0.01035898
C230062I16I	red	-1.3531536	8.05E-05	-1.0763785	0.00278912
Unc5d	skyblue	-1.2274542	0.06215305	-2.5329808	0.00051003
Sox11	skyblue	-2.3920218	3.56E-10	-1.4004416	0.00043611
Jakmip1	orange	1.58546378	1.65E-07	2.29658516	6.67E-14
Brwd3	orange	0.70619552	9.99E-05	0.34943859	0.07045369
Pcdhb5	red	-1.1436867	0.00481451	-0.0777708	0.87967941
Sfxn4	red	-0.9798125	4.58E-06	-1.2513822	9.29E-09
Mapk15	red	-0.829145	4.19E-06	-0.4961651	0.00840888
Chadl	red	-1.1481797	0.00053076	-1.1366001	0.00091609
Chil4	yellow	1.75219603	0.03206387	3.24957934	2.01E-05
Utp14a	orange	0.78915235	3.65E-07	0.62751384	0.00011277
Prpf38a	orange	0.8187525	1.08E-06	0.57803084	0.00096427
Dupd1	skyblue	-3.7126917	0.00022996	-3.2701008	0.0046715
Slc24a3	red	-1.381711	9.95E-11	-0.8096481	0.00026314
Crem	orange	0.88036604	2.79E-07	0.91144817	1.94E-07
Dhrsx	orange	0.72826419	2.51E-05	0.80991061	4.33E-06
Dpp3	orange	0.65435471	3.43E-07	0.61258851	3.17E-06
Slco1a5	skyblue	-1.7947794	8.37E-05	-0.6679244	0.15031901
Wibg	orange	1.11447795	8.15E-14	0.96056815	3.21E-10
Trerf1	orange	0.60753248	0.00499014	0.27080035	0.25456719
Scgb3a1	skyblue	-3.4577809	5.12E-09	-2.2256549	0.00030219
Ipcef1	yellow	2.91352764	5.43E-26	2.96638922	2.21E-26
Fbln2	red	-1.398191	1.50E-19	-0.7845837	1.04E-06
Vrk2	orange	1.08546954	2.93E-05	0.84357502	0.00189268
Cnnm2	red	-0.7797587	1.14E-07	-0.4468919	0.00378968
Hcst	yellow	2.37765577	2.28E-15	2.24143069	2.59E-13
Cadm2	skyblue	-1.8625537	6.29E-05	-1.6977097	0.00027366
Prr36	red	-1.0855809	2.19E-05	-1.0595913	3.18E-05
Cenpj	orange	0.47406458	0.00313996	0.63439425	9.74E-05
Rab44	red	-1.1141118	0.00021831	-0.4816693	0.13871355
Gm4735	orange	1.44601168	0.00792178	1.20670746	0.03908509
4430402I18I	skyblue	-1.4280587	3.84E-07	-1.4140654	5.34E-07
Ifi27	red	-0.9795739	0.00013064	-0.522375	0.05663301
Paqr9	skyblue	-1.8464539	0.00145322	-2.5140748	2.05E-05
Hvcn1	orange	1.37439349	6.67E-05	0.885608	0.01516447

Ccdc146	skyblue	-1.7694037	1.71E-12	-1.0399189	3.61E-05
Tank	orange	0.84575111	1.62E-10	0.71212621	1.39E-07
Cntn4	skyblue	-2.2548833	0.00394647	-1.678622	0.04059136
Aox3	skyblue	-2.4081503	0.00056119	-1.152215	0.12806409
Lrrc51	skyblue	-1.6623321	1.20E-13	-0.9690996	1.85E-05
Hhip	red	-1.1401287	4.06E-12	-0.9893778	3.73E-09
mt-Rnr1	red	-0.6581435	0.00978093	-0.4592345	0.08833306
mt-Rnr2	red	-0.8078053	7.68E-05	-0.6599851	0.00179358
ND1	red	-0.6609651	0.00036811	-0.7661511	4.80E-05
ND2	skyblue	-1.388137	5.86E-08	-1.2832541	9.29E-07
COX1	red	-0.6635491	2.71E-07	-0.6247223	2.15E-06
ND4	red	-1.050643	7.78E-07	-1.1354079	1.39E-07
ND5	red	-0.9771964	3.42E-06	-0.8089293	0.00019577
ND6	red	-0.9342604	1.94E-05	-0.9778291	1.17E-05
CYTB	red	-1.1281528	5.66E-09	-1.1315972	8.16E-09
Sepp1	red	-0.9169387	0.00053149	-0.8035904	0.00326331
Gm25131	yellow	2.66498475	0.00190625	0.91331691	0.42836019
Snord104	orange	1.43361973	4.04E-05	1.01544964	0.00551077
Snora31	orange	1.19718637	0.00134504	1.26838033	0.00103986
Mir142b	yellow	1.61180972	0.0003014	2.8203024	2.44E-07
Gm24494	orange	0.82009113	0.08907373	2.18726173	1.65E-05
Cd209b	skyblue	-3.8248569	0.00042959	-3.820855	0.0007162
2610305D13	red	-0.9183084	0.0066034	-0.8259088	0.01757659
Dhrs3	red	-0.8676962	1.96E-07	-0.6253887	0.00030916
Gm1976	orange	1.13101053	0.00475999	0.98106421	0.01625415
Muc5b	skyblue	-2.7380102	1.60E-06	-0.7442486	0.24552592
Adamtsl1	red	-0.9431039	5.01E-05	-0.4331849	0.08445006
Kndc1	skyblue	-2.3202389	4.26E-11	-1.1947443	0.00102849
Slc31a2	orange	0.97388311	1.94E-06	0.73550975	0.00053751
E230001N04	orange	1.71812903	2.14E-06	0.74304977	0.05803515
Anks6	red	-0.6824058	0.00027028	-0.2847799	0.15831998
Spag8	skyblue	-1.5776017	7.92E-08	-0.788206	0.00717665
Pomgnt2	skyblue	-1.5630891	4.82E-10	-1.255951	1.08E-06
Gm10156	yellow	3.11861008	1.94E-05	2.02019148	0.00102887
Trim12a	orange	1.82214775	9.02E-94	1.74230096	6.74E-85
Vps37b	orange	0.63550924	0.00164355	0.67770986	0.00102768
Rtp3	red	-1.0409199	2.78E-06	-1.1572891	4.24E-07
Wdr6	red	-0.8328715	2.02E-10	-0.4821783	0.00043744
Serpina3c	orange	1.30468236	0.00150535	1.32630097	0.00153604
Serpina3f	black	6.53564448	9.32E-115	5.19437234	6.03E-79
Plekhd1	skyblue	-1.6206241	1.57E-06	-1.0290245	0.00351064
Mthfs	orange	1.09742262	0.00178627	1.00833379	0.0048087
Gm6254	orange	0.76151535	0.00929266	0.74491654	0.01765359

Gm6969	skyblue	-1.6886491	0.0121681	-2.0867233	0.00243907
Gm10167	orange	0.99711537	0.000475	1.01302301	0.00055877
6030419C18	red	-0.7236471	0.00621779	-0.3923091	0.16709286
Wdr35	red	-1.092698	2.66E-29	-0.7726387	2.22E-15
Gm5113	red	-0.7957708	0.0008976	-0.8128475	0.0009567
Pilrb2	orange	1.45058354	0.0004201	1.77782582	3.06E-05
Pilrb1	orange	0.83317555	0.00149919	1.82588667	1.93E-11
Tnfsf18	yellow	1.58990455	0.24261809	4.17068699	0.00374167
Zfp772	red	-0.7051395	1.67E-05	-0.2172549	0.24673888
Hmcn1	skyblue	-1.6366846	2.25E-23	-1.4336488	5.18E-18
Oas1g	black	5.42017044	5.61E-84	5.06789564	1.37E-75
Vsig10	red	-0.6271522	1.86E-12	-0.2958521	0.00172769
Myo1h	red	-1.2373383	0.00018749	-0.6179015	0.07085237
Cryba4	yellow	2.29571581	0.00187032	2.68615228	0.00085121
Serpinb5	red	-0.6339476	0.08286202	-1.008117	0.00598779
Gm3608	orange	1.67738036	0.0001978	0.3368399	0.51810875
Unc93a	yellow	4.3746277	5.84E-05	3.12021429	0.00167768
H2-K2	orange	0.63773702	1.13E-06	0.58452563	1.43E-05
H2-T23	yellow	2.64742812	5.35E-73	2.36382614	3.18E-58
Rplp0	orange	0.71162934	3.93E-11	0.57254522	2.23E-07
Ppp1r3c	red	-0.590035	0.01486382	-0.677455	0.00620016
Ifit1bl2	orange	0.82449247	0.00929136	0.59789643	0.07367882
Bmpr2	red	-0.818828	5.60E-05	-0.7495426	0.00033547
Tmem128	orange	0.93579302	2.11E-16	0.71160297	1.22E-09
Lyar	orange	0.88360208	1.41E-06	0.67564353	0.00039464
B3galt4	orange	0.68236938	0.00096788	0.32455623	0.14508816
Tspan6	red	-0.8037269	7.08E-05	-0.5668866	0.00714566
Zfp763	red	-0.7906528	0.00236213	-0.8347582	0.00170485
A430093F15I	yellow	4.15794811	1.68E-30	4.09339123	7.46E-29
Klra3	yellow	1.87784841	0.00323834	2.41204795	0.00045547
Klra7	yellow	2.03161268	9.94E-10	3.74270896	2.44E-18
Klk11	red	-0.8775615	0.0059854	-0.4342166	0.21281835
Syngap1	red	-1.0836189	3.59E-10	-0.6869625	0.00012057
Lpar5	orange	1.19694733	2.54E-05	0.37266865	0.24854838
Gm10222	red	-0.6049433	0.23238353	-1.674002	0.00114699
Khdc1a	yellow	2.07883829	0.05147081	3.94968486	0.0021429
Xlr4b	yellow	2.48396555	1.83E-10	2.09111401	2.13E-07
Myl9	red	-1.1289396	1.14E-10	-0.7812586	1.66E-05
Pex26	red	-0.8519893	8.27E-07	-0.6255473	0.00050277
Ccdc87	red	-1.1537737	0.00414391	-0.3927027	0.3495062
Gm13139	yellow	2.0563656	4.98E-07	2.1598976	1.23E-06
Zfp760	red	-1.1376048	5.14E-08	-0.879218	4.28E-05
Gtf2f2	orange	1.24289938	1.11E-07	0.81417744	0.00085223

Tcf15	red	-1.3763241	0.00431559	-0.4487922	0.43203311
1500009C09	yellow	2.79694651	0.01654294	4.4424631	0.00035175
Cenpm	yellow	3.14974746	1.18E-12	3.3398767	7.30E-13
Cst7	yellow	4.11234139	2.30E-54	3.56329393	1.81E-43
Col8a1	red	-1.0922651	1.23E-06	-1.2433082	7.96E-08
Macrod2	red	-0.6433251	0.01333153	-1.0540209	9.06E-05
Lgals1	orange	1.83254533	3.68E-09	1.48873337	3.15E-06
Il2rb	yellow	4.47747276	1.27E-87	4.21464468	2.18E-77
Gm11808	orange	0.75275385	0.00390314	0.25427464	0.39400475
Phf11d	yellow	2.9876	1.35E-37	2.95823042	9.19E-37
Apol9b	black	4.60960857	2.66E-41	4.42815731	2.20E-38
Efcc1	skyblue	-2.838174	1.40E-17	-1.9934188	3.16E-09
Shroom4	red	-0.853059	5.74E-07	-0.4606494	0.01068352
Slc4a5	skyblue	-2.5596867	1.06E-05	-1.4623533	0.01699715
Aup1	orange	0.60608866	5.13E-07	0.36418689	0.00400079
Dok1	orange	1.47250799	3.50E-13	1.2938359	3.54E-10
Cep152	orange	0.91124692	1.64E-09	0.82898869	8.88E-08
Pnp2	yellow	2.54135158	3.55E-13	2.00657954	2.52E-08
Gmnc	skyblue	-1.6405477	0.00193446	-0.8150822	0.12429299
Duox2	skyblue	-2.2724338	0.00011233	-0.8829114	0.15385019
Aard	skyblue	-2.4876422	1.94E-11	-1.9133186	4.64E-07
Zfp467	red	-0.6756218	9.87E-07	-0.5850183	3.51E-05
Gm4841	black	6.35044088	5.92E-62	7.38296937	4.87E-66
Actc1	skyblue	-2.4104755	5.10E-06	-1.8594751	0.00068934
Efcab1	skyblue	-2.3454481	2.44E-12	-1.9764794	3.89E-09
Gpr88	orange	1.08061286	0.008897	1.92525327	4.20E-06
Flnc	red	-1.2949491	7.16E-07	-0.5465154	0.0528145
Trp53i11	orange	1.55828074	1.61E-12	1.82998435	9.87E-17
Sars	orange	0.5915235	1.66E-06	0.43388087	0.00072368
Celsr2	red	-0.798175	5.71E-05	-0.4038863	0.05723876
Cry2	red	-0.9375552	3.23E-05	-0.8005722	0.00058017
Psrc1	orange	2.03548551	1.49E-09	1.88044893	4.17E-08
Mybphl	skyblue	-1.9622614	0.01442407	-2.4484802	0.00253933
Sort1	skyblue	-1.6719625	1.11E-07	-1.3568987	2.90E-05
Ptprz1	red	-1.101258	1.51E-06	-0.5039149	0.03370497
Psma5	orange	1.34239546	3.71E-14	1.20591131	2.13E-11
Il3ra	orange	1.50108034	4.51E-11	1.27890434	4.11E-08
Gstm6	red	-1.1904183	0.00383479	-0.8737886	0.04630157
Hist2h2be	red	-1.1723303	2.32E-09	-0.8050718	7.59E-05
Sf3b4	orange	0.65028127	9.82E-05	0.39175372	0.02684043
Ssb	orange	0.63633908	3.37E-08	0.37355179	0.00208483
Lce3f	red	-0.9232622	0.00274053	-0.5121615	0.12308523
Lce1j	skyblue	-2.0355385	0.00033006	-1.0027588	0.09745122

Lce1i	skyblue	-1.5708163	0.00312366	-1.2868494	0.01990274
Lce1e	skyblue	-2.0277919	6.30E-06	-1.3418246	0.00415688
Lce1a2	red	-1.2553487	0.00030451	-0.7152147	0.05402131
Msto1	orange	0.68157514	0.00081877	0.62172541	0.00313918
Gm5641	orange	1.66363849	0.00922454	1.15562586	0.12412865
Cdk7	orange	0.6302054	0.0034485	0.48027351	0.03351039
Adgrv1	skyblue	-2.1265977	0.00098155	-1.4547046	0.03097478
Nr2f1	skyblue	-1.3207864	5.00E-07	-1.393494	2.85E-07
Zfp72	red	-1.0889672	0.00066658	-1.0351595	0.00212752
Zfp874a	red	-0.5355757	0.04948761	-0.869674	0.00153027
Hist1h2bj	yellow	2.601034	0.03092256	4.39135527	0.00498065
Hist1h2ag	black	5.89395413	0.0001641	3.47419515	0.00817008
Hist1h3c	yellow	3.24504661	0.0001786	4.45274244	0.00056198
Ctxn3	red	-1.148679	0.00304457	-0.9134828	0.02507013
Prdm6	red	-1.1578021	1.46E-07	-0.8884489	9.34E-05
Lyz1	skyblue	-2.1688946	4.89E-05	-1.5616597	0.00500819
Lyz2	red	-1.4623657	5.26E-06	-1.0223627	0.00224451
Ank3	red	-0.7416005	0.00057026	-0.4521968	0.04808581
Gm10275	orange	0.86480927	0.002099	0.4405298	0.15081111
Gm5595	red	-0.8879873	0.00052537	-1.1349521	1.10E-05
Psmb3	orange	1.08275814	9.87E-12	0.90065024	3.22E-08
Tmem100	skyblue	-1.9755868	1.55E-10	-1.3406276	2.83E-05
Msi2	red	-0.6123168	0.00046648	-0.5334301	0.003137
Wfdc17	yellow	3.38693605	1.72E-16	3.49857036	3.13E-17
Slfn9	yellow	3.74551791	1.39E-39	3.58010987	5.04E-36
Cacng7	skyblue	-1.6539016	9.53E-08	-0.9161871	0.00304371
Nlrp1a	yellow	2.39489144	0.00267196	3.16540274	0.0005688
Ahnak	red	-0.5909832	9.29E-07	-0.477926	0.00012162
Sco1	orange	1.12752941	6.27E-07	0.40261976	0.10307839
Pabpn1l	skyblue	-2.9218999	0.01098233	-4.1223689	0.00472952
4930438A08I	black	5.80624979	9.71E-05	3.77697633	0.00017774
Irgm2	yellow	2.27363339	3.90E-29	2.32478932	2.16E-30
9930111J21F	orange	1.58462806	9.35E-18	1.34070842	9.60E-13
9930111J21F	yellow	2.08397068	2.47E-08	2.20071377	6.87E-09
Gm12166	orange	0.72139765	0.00144949	0.40078577	0.10710552
Spdl1	yellow	2.95938535	9.09E-23	2.80167293	2.05E-20
Hba-a2	red	-0.5882068	0.13929435	-1.1286427	0.00370932
Hba-a1	red	-0.5052548	0.16699056	-1.1685124	0.00093947
Fcho1	yellow	2.4059204	9.45E-35	2.56390419	1.06E-38
Ssbp4	orange	0.77182307	0.00013188	0.75363706	0.00027592
Sp140	yellow	2.29726179	8.63E-27	2.32812915	3.64E-27
Sp110	yellow	2.50332942	5.64E-48	2.46047296	3.91E-46
Fam149a	red	-0.9417498	3.57E-08	-0.592255	0.00084197

Fat1	red	-0.666716	1.52E-06	-0.315858	0.03321981
Gmppb	orange	1.07542659	2.99E-12	1.05397576	1.32E-11
Mpzl3	orange	0.80506695	0.00077125	0.36034384	0.16611578
Ccdc153	skyblue	-2.246412	1.91E-18	-1.4033525	1.07E-07
Rnf213	yellow	2.76579754	2.33E-68	2.72512616	2.27E-66
Prss36	skyblue	-1.6443081	2.74E-10	-1.1898664	9.98E-06
Capza1	orange	1.05429522	6.33E-09	0.8698902	3.16E-06
Ampd1	skyblue	-1.8553858	1.78E-05	-1.0155894	0.0282268
Fbxo39	yellow	3.22161866	1.01E-10	2.32623438	2.34E-08
Nlrp1b	yellow	3.70465283	9.16E-32	2.72295625	9.77E-20
Art5	red	-1.4324979	0.00390137	-0.8245626	0.12430572
Il18bp	yellow	4.20132378	1.77E-63	3.69300568	6.36E-50
Adamtsl3	red	-1.051871	4.48E-06	-0.8678837	0.00024297
BC094916	yellow	4.04911016	1.71E-20	3.66382735	5.33E-16
Fcrl6	black	5.05399821	0.00082672	4.48934197	0.00451562
Rgma	skyblue	-1.5072681	9.61E-09	-1.1006126	4.95E-05
Fcrlb	black	5.43565449	7.26E-27	5.77555386	1.85E-25
Wfdc10	skyblue	-6.5319191	2.13E-05	-0.2116878	0.86868079
Wfdc16	skyblue	-6.6323734	3.84E-05	-6.0524766	0.00024365
Rasal2	red	-0.7009034	0.00550358	-0.8851603	0.00052673
Slc17a7	skyblue	-1.7862901	0.12223247	-2.9838995	0.00919326
Mn1	red	-1.0842243	5.24E-07	-0.8505971	0.00014589
Gm572	skyblue	-1.7082872	0.00050022	-0.7706627	0.15138163
Fv1	red	-0.9246796	0.00518769	-0.6728994	0.05011165
Gm13251	orange	2.06671254	0.00208473	1.48151162	0.03404559
Sox13	red	-0.9704746	4.34E-08	-0.7705104	2.44E-05
Rnf186	skyblue	-2.0349359	7.24E-14	-1.4628294	1.60E-07
Runx3	yellow	3.96013265	1.90E-31	3.57914481	9.40E-26
Sars2	orange	0.7741406	1.57E-05	0.69910829	0.00016364
1700049G17	red	-0.7475532	0.00062003	-0.7641349	0.00062058
Gm10282	orange	0.66487352	7.09E-05	0.63327349	0.00020734
Tmem200b	skyblue	-1.6834443	1.85E-12	-1.2073968	8.47E-07
Rmdn3	orange	0.79789339	2.49E-10	0.73523177	1.06E-08
Gm12942	red	-0.7199931	9.15E-05	-0.7746358	4.56E-05
Rbm47	orange	0.32509024	0.04092757	0.61125937	1.00E-04
Pnmal2	skyblue	-1.7760368	6.61E-12	-1.8462026	1.66E-12
Zmynd12	skyblue	-1.7688746	3.24E-06	-1.139108	0.00219308
6330408A02I	red	-0.9631453	3.06E-09	-1.0270408	6.08E-10
Trabd2b	red	-0.6922333	0.00295939	-0.8024925	0.00072664
Lilra5	red	-1.4813183	0.00031635	-0.8761161	0.04532625
Ldlrad1	skyblue	-2.667737	2.99E-14	-1.5106995	1.75E-05
Rabepk	orange	0.89226397	0.00017054	0.76224211	0.00194449
Rasgrp3	red	-1.1941597	1.00E-12	-0.9490135	3.74E-08

Treml2	yellow	2.03927738	9.82E-10	2.0743069	3.25E-10
Nr2c2ap	orange	0.8779066	0.00971113	1.16061858	0.00167472
1700029J07F	skyblue	-1.6294652	5.79E-08	-1.0699025	0.00052524
Arhgef10	red	-0.9641228	3.65E-10	-0.6748684	2.24E-05
Ccdc78	red	-1.2418989	2.65E-07	-0.6796722	0.00683194
Naip5	yellow	2.11097486	6.17E-17	1.95953021	1.18E-14
Npw	skyblue	-2.0863799	1.63E-10	-1.1338256	0.00079414
Gpr31b	black	7.39554331	4.85E-07	8.06991734	6.21E-08
Bves	skyblue	-1.7976705	1.43E-08	-1.6562566	3.38E-07
Armc2	skyblue	-1.7770502	3.21E-09	-1.3654649	7.16E-06
G630090E17	skyblue	-2.2377853	0.00837382	-1.6666665	0.0578368
C1qtnf9	skyblue	-1.48675	0.00543481	-1.4819083	0.00745583
Setdb2	yellow	2.08615114	1.30E-35	1.96619103	5.10E-31
Ect2l	skyblue	-2.1431724	1.46E-08	-1.1382182	0.00251877
2410004P03I	skyblue	-1.8006709	3.39E-20	-1.1112633	2.14E-08
LOC1008624	orange	0.58869981	2.71E-05	0.43382631	0.00297323
Psmg4	orange	1.41594036	1.47E-13	1.13082583	8.39E-09
1110002L01F	red	-0.7944777	0.0092433	-0.5847192	0.06604376
Tmem139	red	-0.7034747	0.02411417	-0.927124	0.00242336
Klrg2	red	-0.6416424	0.00547361	-0.1673733	0.54315754
3425401B19I	skyblue	-1.9390607	8.70E-09	-1.5031741	1.81E-05
Cfap44	red	-1.5156325	1.14E-06	-0.6884128	0.03731244
Akr1c19	red	-1.2073754	4.39E-05	-0.554642	0.08420677
Tigit	black	7.48778557	1.36E-62	5.78554522	4.10E-63
Fam189a2	red	-1.0669796	8.13E-05	-0.7643174	0.00683346
Cebpd	orange	1.60314618	1.41E-11	1.03186801	2.82E-05
Mta2	orange	0.58141971	5.71E-17	0.47322449	2.51E-11
Rom1	orange	0.64933054	4.24E-05	0.6966291	2.42E-05
Ints5	orange	0.63884197	3.58E-06	0.28128311	0.059463
Lrrn4cl	red	-1.4438288	1.86E-06	-1.0966228	0.00059081
Ttc9c	orange	0.72357392	7.26E-07	0.60768201	5.41E-05
Mpst	red	-0.7540962	9.89E-06	-0.4953547	0.00550025
Csf2rb	yellow	2.27081888	9.02E-14	2.32442754	3.13E-14
Csf2rb2	yellow	2.71174983	9.04E-14	2.83316993	8.63E-15
Ncf4	yellow	3.09207267	2.01E-23	3.07219128	6.12E-23
Apol7e	yellow	3.44462721	3.48E-23	3.82042227	9.72E-25
Tmem28	skyblue	-1.6993658	4.33E-09	-0.9846994	0.00094847
Smpd5	orange	1.21856783	0.00081828	1.12702211	0.00272189
Zhx2	red	-0.9512685	5.46E-10	-0.8836271	1.47E-08
Apcdd1	red	-1.1940298	2.95E-15	-1.0423194	6.75E-12
Mcc	red	-1.1656665	1.24E-08	-0.8720869	3.86E-05
Gm94	red	-0.9172433	0.00829747	-0.7192051	0.04833562
Fndc1	skyblue	-2.0917991	4.33E-33	-1.4361113	4.76E-16

Ccnf	yellow	2.7279135	2.99E-34	3.22392103	1.04E-42
A530040E14I	yellow	3.01805184	3.84E-10	3.37270339	1.57E-11
Ang	skyblue	-1.2602346	4.85E-13	-1.2760662	9.90E-13
Trim6	orange	1.69242577	3.93E-08	1.64032507	1.14E-07
Klf12	skyblue	-1.2594642	9.47E-06	-1.2322725	2.41E-05
1700024G13	skyblue	-3.9713637	3.05E-12	-2.4265127	1.21E-05
Ppp1r3e	red	-0.5902657	0.00030296	-0.4375579	0.01049771
Fam84b	red	-1.4117871	3.88E-10	-1.0727733	3.91E-06
Slc39a2	orange	1.26167462	0.00267297	0.62495473	0.17111879
Gm10369	orange	1.81898053	0.00417432	1.21096937	0.05492083
5930412G12	skyblue	-2.5406749	4.42E-05	-0.8392255	0.19106467
Slfn2	yellow	3.83747826	4.90E-44	3.76319781	8.65E-42
Slfn10-ps	black	4.32856762	8.18E-06	7.39511299	5.70E-07
Lyrm9	red	-0.6621198	0.00100818	-0.7884964	0.00013498
Adam1a	red	-0.6694319	0.06145344	-1.1271127	0.00150424
Spef2	red	-1.3486936	6.31E-06	-0.7970464	0.00996804
Smim10l1	red	-0.8856757	2.10E-08	-0.8444778	1.66E-07
Myo18b	red	-0.6367824	0.00299205	-0.5289578	0.01822136
Klra14-ps	orange	1.10314595	0.06450949	1.61235299	0.00968954
Gm10419	skyblue	-1.7863498	0.00186814	-0.9186326	0.12923552
Acrbp	red	-1.048371	1.55E-05	-1.1442635	3.95E-06
G530011O06	yellow	3.74973428	8.84E-06	3.46963213	4.83E-05
Gpr27	skyblue	-1.6791875	1.02E-06	-1.194102	0.00097098
4933439C10	red	-0.6356239	0.00366057	-0.2584482	0.2838747
Gm10435	skyblue	-2.4545671	0.0058843	-3.1546565	0.00064214
Gm12258	red	-0.8842665	0.00019132	-0.3661057	0.15681121
Sod3	red	-0.6594869	1.09E-05	-0.3705641	0.01959683
Nup62cl	skyblue	-1.8510908	0.00318319	-1.2430074	0.05079321
Ptgr2	red	-0.7500519	5.89E-06	-0.4431449	0.01111574
Acot1	red	-1.1639691	4.28E-05	-0.4321234	0.16633178
Gprasp2	skyblue	-3.0817491	2.76E-06	-1.6193244	0.01688374
Oip5	yellow	3.23799677	7.63E-08	1.74473927	0.00359644
Vamp5	red	-0.5883994	2.37E-05	-0.4466869	0.0022436
Gpr174	yellow	2.29068743	6.35E-14	2.41242137	8.17E-14
Drc1	skyblue	-1.9889167	4.79E-19	-1.4460976	1.40E-10
4930599N23	yellow	1.86085574	7.85E-06	2.06614123	1.17E-06
Gm12000	yellow	2.67675201	0.00399587	3.95361719	0.0035577
9330158H04	red	-0.623139	0.02691901	-0.9237615	0.00137116
2500004C02	skyblue	-1.358532	0.00021492	-1.2247842	0.00131792
Nudt10	skyblue	-1.5952226	0.00070804	-1.2346672	0.00848137
Nudt11	skyblue	-1.8490462	1.44E-05	-1.290136	0.00311294
C920025E04	yellow	2.55246101	2.75E-23	2.47256734	4.29E-21
Gm6034	yellow	3.44630846	1.11E-08	2.20816052	8.78E-05

H2-Q6	yellow	2.19315427	3.72E-30	2.03863195	4.43E-26
H2-D1	orange	1.75993329	9.54E-31	1.49637198	2.42E-22
Lst1	yellow	3.11672731	8.99E-23	2.99737614	1.13E-19
Ly6g6d	skyblue	-1.4321929	1.13E-06	-1.3068118	3.05E-05
C4b	orange	1.06556931	7.48E-09	1.2138045	5.70E-11
H2-Ab1	orange	1.71742306	5.95E-20	1.31662759	6.54E-12
Cyp4f15	skyblue	-2.7233558	3.76E-10	-1.4331653	0.00129432
Gm10505	yellow	1.6916666	0.05968684	2.72632101	0.00448869
Arhgdig	red	-1.3584901	1.40E-08	-0.826306	0.00089842
Sft2d1	orange	0.70290704	0.00035869	0.59315226	0.00365169
Rsph3a	red	-0.756887	2.04E-06	-0.5399559	0.00124368
Ifi204	black	6.02132166	1.45E-55	5.22197265	1.21E-42
AI607873	yellow	4.1503327	1.59E-32	3.43542126	1.17E-22
Pydc4	yellow	2.74457156	1.49E-39	2.52952413	1.31E-33
Gm10521	yellow	2.28356659	3.44E-07	3.25042944	2.29E-12
Gm4951	yellow	3.9963364	4.02E-30	3.75785195	9.71E-27
Ppp1r12b	red	-0.8669168	9.46E-12	-0.4972908	0.00018954
3222401L13F	skyblue	-2.0800887	1.16E-06	-1.4561598	0.00092602
Pcdhb22	red	-0.8791334	6.34E-05	-0.6572812	0.00498608
1700066B19I	yellow	2.51486745	1.81E-05	1.59390882	0.00470344
Prob1	skyblue	-1.6904387	5.57E-11	-1.4289783	6.04E-08
D2hgdh	red	-0.6272871	0.0005251	-0.6908553	0.00018957
Fbxo36	red	-0.9307225	0.00119455	-0.6868308	0.02190939
Catip	skyblue	-1.7758451	2.24E-17	-0.9303246	1.43E-05
Nbeal1	red	-0.8374537	1.47E-05	-0.5996441	0.00287227
Klhl21	red	-0.9170462	6.26E-10	-0.7115522	3.15E-06
Rpl31	orange	0.59553241	0.00021546	0.37705654	0.02645714
Apitd1	yellow	2.41606851	1.44E-12	1.70522341	2.96E-06
Gm13241	yellow	2.67835143	0.00703054	1.8084504	0.04249949
5730409E04I	red	-0.7763373	4.95E-06	-0.6788943	0.00011834
Btbd19	orange	1.29522524	5.68E-09	1.02313206	7.62E-06
Gm10575	red	-0.7104591	0.00094363	-0.4954382	0.02420595
Alg6	red	-0.6618221	0.00552464	-0.2237415	0.41356167
Cdkn2b	orange	0.65572026	2.08E-06	0.44008838	0.0023161
Fam196a	red	-0.5305739	0.07915151	-1.0993324	0.00052411
Iqck	red	-0.7836396	0.0020085	-0.4269162	0.11403528
Ccl27a	red	-0.8737089	0.00011157	-1.012329	1.34E-05
Il11ra1	red	-0.6780681	7.41E-06	-0.652733	2.59E-05
Gm1966	yellow	2.81292408	1.78E-21	3.08565882	2.61E-25
Mob3b	red	-0.9419289	1.85E-10	-0.4965737	0.00140937
Hbb-bt	red	-0.8892137	0.03128833	-1.1841994	0.0043499
Olfr550	skyblue	-1.1242767	0.03455225	-1.5131716	0.00600624
Gm9574	yellow	1.77578782	1.04E-07	2.44909942	1.28E-12

Rhog	orange	1.35514223	6.96E-11	1.1123978	1.79E-07
Ttpa	red	-0.9191602	0.00521211	-0.637396	0.0593869
Klhl40	skyblue	-2.1087588	4.30E-08	-0.8173287	0.04991458
Omp	orange	2.04538764	0.00020864	1.70566724	0.00177073
Fam169b	orange	1.22252319	1.43E-07	1.97101928	4.72E-17
Snrnp40	orange	0.88203931	1.57E-07	0.74031118	2.02E-05
Svip	skyblue	-1.7918303	2.33E-08	-1.2608138	0.00014346
Saa1	black	9.15344908	4.32E-12	7.17760219	1.96E-09
Rpl13a	orange	0.58287815	0.00051627	0.50634379	0.0039954
Nlrc5	yellow	3.29760062	2.10E-69	3.41489981	2.40E-74
Ces1h	red	-1.3831654	0.00094064	-0.9484252	0.03089536
Zfp788	red	-0.5776711	0.00259384	-0.5901595	0.00250323
Zfp791	red	-0.9576227	0.00372483	-0.552878	0.10997279
Adh1	skyblue	-1.2663579	1.14E-18	-1.2538183	4.46E-18
2210011C24	skyblue	-1.9937487	2.27E-05	-1.3512234	0.00592155
Cox7a1	skyblue	-2.2259714	1.19E-06	-1.9599816	3.57E-05
Zfp382	orange	1.53178056	0.00084029	1.56583003	0.00085121
Dda1	orange	0.58423095	5.16E-05	0.551819	0.00019895
Gm10654	yellow	2.05903139	0.00729852	4.6332301	1.02E-06
Cyp2a4	skyblue	-3.6143163	0.00142849	-2.5897283	0.01398194
Amy1	red	-1.4315539	3.86E-05	-0.9986547	0.00559589
Zfp94	red	-0.8806196	0.00239388	-0.8853778	0.00282437
I830077J02R	yellow	2.89233959	4.40E-19	2.76792523	1.85E-17
C5ar2	orange	1.53583002	2.59E-05	1.44265784	0.00012445
Ehd2	red	-0.6671096	1.99E-08	-0.433711	0.00046858
AI429214	red	-0.7509426	0.02343843	-0.9741232	0.00372491
Pira11	yellow	3.84151892	2.18E-08	2.67297545	5.93E-05
Gm15448	yellow	3.74537829	2.37E-08	3.24325013	7.88E-07
Zfp872	orange	1.67992857	0.00111951	1.00379556	0.06959608
Spc24	yellow	2.15234009	3.04E-12	2.31369223	2.11E-13
Mex3a	orange	0.09281356	0.79942726	1.0944106	0.00053892
Clec4g	skyblue	-2.7479324	0.00352942	-2.8514837	0.00286501
A430078G23	orange	1.28057335	5.78E-07	0.96338567	0.00027736
Fat3	skyblue	-1.504627	1.08E-12	-1.5726953	3.85E-13
Zfas1	orange	1.59526429	3.18E-20	1.46740219	5.72E-17
4931440P22I	orange	0.83221561	0.00806886	0.79717731	0.01625456
Ankub1	skyblue	-1.7829129	0.00021957	-1.0881857	0.01344824
Mafb	yellow	2.92601367	1.40E-18	2.64309135	3.60E-15
Tldc2	yellow	1.33070443	0.0590225	3.17591111	0.00169637
Gm7120	red	-0.173013	0.73879024	-1.5596849	0.00075336
Sox2	red	-1.2746551	3.52E-09	-0.6975786	0.00189795
Mcidas	yellow	1.88005772	0.00538244	2.61717184	8.56E-05
Eif2s2	orange	1.24392535	2.13E-07	0.97838326	8.03E-05

Ttll9	red	-1.052925	0.00295334	-0.8411428	0.01889423
Foxs1	orange	1.20657231	0.0199216	1.42188206	0.00756585
LOC1000389	yellow	4.18837556	6.96E-09	4.20467398	1.06E-08
Defb23	skyblue	-3.9797472	0.00019508	-0.693228	0.38149283
Zfp950	red	-0.6196007	0.00445169	-0.6777856	0.0023558
Thbd	skyblue	-1.4631616	4.08E-10	-1.2290427	2.95E-07
Kiz	red	-0.9596054	2.23E-06	-0.760775	0.00027736
Gm5535	yellow	3.00601569	0.00164216	3.54286844	0.0010191
Ankef1	skyblue	-2.426814	0.00166133	-1.5031225	0.05815933
Ube2n	orange	0.61546945	1.10E-05	0.38527229	0.00885995
4833422C13	red	-1.4050759	0.00023967	-1.0692454	0.0064653
Plxnc1	orange	0.59250999	0.00023086	0.48825272	0.00355846
Arrdc3	red	-1.2774733	8.75E-05	-1.0275294	0.00231374
Slc4a11	orange	1.72566056	2.52E-06	1.66870864	6.81E-06
Itpa	orange	0.54405729	0.00131806	0.59870305	0.00052729
Gas2l3	orange	1.6054283	1.01E-05	1.87734309	6.58E-07
Gm14005	orange	1.0665129	0.0002764	1.15112517	0.00012932
Pdzd7	red	-0.7142167	0.00194916	-0.9249574	6.67E-05
Hpse2	skyblue	-2.1298826	0.00017917	-1.4257296	0.01136891
Ctla2b	orange	0.74375828	0.04473289	1.02358731	0.00752773
Grk6	orange	1.10088266	3.74E-12	1.05618403	4.53E-11
Ifit3	yellow	3.6383125	4.44E-132	3.52236213	6.08E-124
Gm14207	skyblue	-1.4053854	2.61E-07	-1.3433382	1.02E-06
Pak6	orange	1.4747955	2.00E-28	1.0146678	2.38E-14
Fibin	skyblue	-1.6931627	0.00140266	-1.3168171	0.01707498
Dcdc5	skyblue	-3.0769743	0.00219145	-0.9092416	0.39098227
Qser1	red	-0.6345465	0.00037085	-0.4269303	0.02268116
AW112010	yellow	3.39628903	8.93E-58	3.03482563	5.94E-46
Fjx1	orange	0.45514419	0.01762743	0.63897708	0.00101554
Yae1d1	red	-0.9069528	1.30E-10	-0.6244098	2.20E-05
Cd80	yellow	3.18269967	1.03E-14	2.09169161	4.69E-07
Ccdc162	skyblue	-1.9853191	1.52E-14	-0.9707578	0.00028402
Heg1	red	-0.8580136	2.77E-09	-0.5433768	0.00030803
Cenpw	yellow	2.2059763	1.53E-07	2.81710583	6.21E-10
Ttc30a1	red	-0.538668	0.04228785	-0.791697	0.00360677
Ttc30b	red	-1.4581599	6.54E-09	-1.0338452	6.27E-05
Wipf1	orange	0.78747923	2.27E-06	0.56349487	0.0011509
Aldh3b2	red	-0.9408339	0.0030665	-0.7710736	0.02003912
Erich2	skyblue	-1.5663355	2.99E-10	-1.2970293	3.00E-07
Sp5	red	-1.1217348	0.00339616	-0.6985613	0.0918659
Klhl41	red	-1.0166326	0.00013989	-0.7942049	0.00429444
Scn2a1	skyblue	-1.5146192	0.00702696	-0.8361675	0.13673299
Fign	skyblue	-1.4755009	4.33E-09	-1.3541239	1.03E-07

Rprm	red	-1.1743681	0.00765642	-0.9848899	0.03138556
2810410L24F	red	-1.3200439	1.60E-10	-0.9323185	1.06E-05
Hoxc4	skyblue	-1.5052693	7.10E-05	-1.1420624	0.00371064
Smim6	red	-1.090428	0.00016583	-0.645143	0.03546331
Gm13375	red	-0.8591454	0.00112444	-0.8494156	0.00178237
Rsph10b	skyblue	-1.8459088	7.89E-09	-0.9638194	0.00362316
6330403L08F	red	-1.0074078	1.48E-14	-0.7940613	3.49E-09
Hoxb2	red	-0.8153532	0.00309785	-0.2651865	0.38342417
Nynrin	red	-0.940571	1.88E-08	-0.760938	7.72E-06
Zfp652	red	-0.6369638	0.00014706	-0.5351497	0.00203295
Ly6a	yellow	2.18972903	4.46E-49	1.93749953	1.64E-38
Msrb1	orange	1.18017533	1.82E-07	0.97663789	2.78E-05
Sox4	red	-1.467146	1.96E-16	-0.6435943	0.00070558
Wfdc3	skyblue	-1.567679	0.00124225	-0.946216	0.05743205
Acsf2	red	-0.6350379	5.70E-05	-0.4845429	0.00302034
2700094K13I	orange	0.75019962	3.43E-08	0.54675917	0.00011404
Ass1	orange	1.44406369	2.38E-13	0.95864758	2.75E-06
Trbv1	yellow	2.95082263	1.35E-07	3.35204854	4.96E-09
Trbv2	yellow	1.77357579	0.00055415	3.37916028	2.04E-08
Trbv3	yellow	3.22276127	4.48E-08	3.71945976	1.50E-09
Trbv13-1	black	2.66029025	9.12E-06	4.94175983	1.25E-11
Trbv13-2	yellow	0.89134767	0.14973655	3.2215148	1.81E-07
Trbv13-3	yellow	2.32298164	7.02E-05	3.98546174	2.26E-07
Trbv14	black	4.41519891	2.42E-11	4.85356772	1.25E-11
Trbv15	yellow	2.24509667	0.00673768	4.03898373	1.60E-05
Trbv16	yellow	2.78347476	3.43E-08	4.07389388	3.34E-12
Trbv17	yellow	3.15687167	0.00035394	3.88106294	4.32E-05
Trbv19	yellow	3.83286729	4.17E-12	3.92520559	2.04E-10
Trbv20	yellow	3.23253268	8.81E-09	3.97663414	1.12E-10
Trbv26	yellow	2.73753078	0.00406969	1.85135791	0.06534277
Trbv29	black	3.33483232	2.11E-07	5.45613747	7.30E-11
Trbj1-3	yellow	1.37391249	0.02348951	3.37631336	1.21E-06
Trbj1-4	yellow	1.8177358	0.00505536	2.41197722	0.00035175
Trbj1-5	orange	1.28274169	0.019666	2.44055675	3.48E-05
Trbj1-7	yellow	1.88548329	0.04005106	3.43858914	0.00352952
Trbc1	yellow	1.93425298	0.00225598	3.42106913	6.33E-08
Trbj2-1	yellow	0.9254043	0.19924751	3.56840425	3.18E-05
Trbj2-2	yellow	1.76936345	0.02496277	3.86611822	0.00065939
Trbj2-3	yellow	1.56371512	0.08556506	3.37833565	0.00216434
Trbj2-5	yellow	2.30791277	0.02671942	3.36800117	0.0059693
Trbj2-7	yellow	2.15822433	0.00011603	3.20191175	8.99E-08
Trbc2	yellow	2.30037326	0.00172858	3.43302093	3.28E-06
Trbv31	yellow	2.25559512	0.00106864	4.52041285	9.62E-07

Igkv2-112	skyblue	-2.5303225	0.00741257	-1.2697705	0.28681287
Igkv4-50	yellow	4.002866	0.00978195	2.80775256	0.0885085
Igkv5-48	yellow	3.0894726	1.94E-06	3.61701961	7.47E-08
Ighg2c	yellow	4.29122673	5.50E-05	1.667971	0.14967572
Ighg2b	yellow	2.99729162	0.00108346	3.12142834	0.00090028
Ighg3	yellow	3.59738183	0.00696646	2.07884256	0.14596563
Ighj1	yellow	3.12972948	0.00453379	1.50379757	0.13785546
Tcrg-C2	yellow	2.45190854	4.42E-08	2.12596883	5.90E-06
Trav3-1	yellow	1.18894123	0.47043227	6.04740263	0.00151007
Trav8d-2	black	6.55548958	0.00010515	2.07064503	0.1606116
Trav16d-dv11	black	6.22521331	0.0035154	3.72961046	0.08989549
Trav16n	black	4.73462539	4.89E-07	5.71700072	1.20E-05
Trav14n-3	black	3.95610859	0.00244704	5.05801671	0.00066918
Trav13-1	black	3.63278159	0.00095582	5.391157	0.000684
Trav16	black	5.0468954	1.06E-06	5.61121666	7.44E-05
Traj16	black	4.37815172	0.00018512	5.92808593	0.00011181
Trac	yellow	3.56359511	2.42E-64	3.55053044	2.06E-61
Snora17	orange	1.59385409	0.00030205	0.39039827	0.42866346
Snord89	orange	1.91100936	0.00054101	1.89415804	0.00074131
Snord83b	orange	1.85164107	0.00283975	1.75086614	0.00808935
Mir208b	skyblue	-3.2040951	0.00069322	-3.105674	0.00096254
F630028O10	orange	1.17273649	0.00084736	0.65653555	0.07837707
Ankrd63	skyblue	-4.2088014	2.47E-07	-2.6289037	0.00193387
Capns2	red	-0.8801928	0.00449607	-0.8140902	0.01145084
Erich3	skyblue	-2.0325936	5.88E-22	-0.5801153	0.00794399
Dnm3os	orange	0.78792812	0.00622034	0.73074673	0.01490247
Gm2000	orange	1.43237732	0.00177199	0.19873895	0.72574913
Nrarp	red	-0.9509811	0.00030486	-0.6173818	0.02473091
Klhdc7a	skyblue	-1.5950533	9.95E-11	-0.7103561	0.00597746
Krtap17-1	skyblue	-1.4334065	0.00072832	-2.0764563	5.13E-06
AI593442	skyblue	-0.4031235	0.7535279	-3.608852	0.00752909
AW011738	orange	1.01262086	1.64E-07	1.12425638	7.51E-09
Smim1	red	-0.777641	0.00746508	-0.4507367	0.14773482
Gm4294	orange	1.71046871	0.00581619	0.67622216	0.34827974
Ppil6	skyblue	-2.1792473	1.82E-08	-1.2023682	0.00234686
Raet1a	yellow	3.18175043	0.00520582	4.6218015	0.00512302
Abracl	orange	1.43341346	4.15E-18	1.04149666	9.76E-10
Ankrd65	skyblue	-2.2905327	0.00014787	-1.7995849	0.00337839
Cfap74	red	-1.440904	6.75E-06	-0.9191766	0.00551077
Gm13152	red	-1.446711	0.0023209	-0.8261885	0.09307604
Gm13212	yellow	2.45696324	4.83E-18	1.41114198	9.39E-07
Aunip	yellow	4.08270665	6.86E-16	2.93084272	7.99E-10
Nkain1	skyblue	-1.5730189	1.14E-05	-1.4810016	6.53E-05

Dcdc2b	skyblue	-1.8125551	1.04E-08	-1.1421934	0.00041761
Bnip3	red	-0.3546753	0.12307606	-0.6239523	0.00596699
Cyp4a12b	skyblue	-5.6609396	1.15E-11	-4.165506	3.24E-10
Gm4070	yellow	2.46333622	2.40E-37	2.27325882	1.20E-32
1810010H24	skyblue	-1.8954083	1.20E-08	-1.1584015	0.00081627
Trim30c	black	4.33223604	9.64E-16	7.19299891	1.59E-13
Gm12695	skyblue	-2.9660305	5.33E-05	-1.8925462	0.00856385
Gm11627	skyblue	-1.980429	2.37E-06	-1.2315454	0.00674585
Aoc2	orange	0.54305518	0.01727317	0.70076613	0.00233396
Fam174b	skyblue	-1.470575	1.54E-13	-1.1733619	8.84E-09
Tmem8b	red	-1.268394	8.33E-08	-0.9431394	0.00011694
Slfn1	black	5.44564733	5.47E-21	5.4954827	7.69E-21
Evi2a	yellow	2.63416132	3.59E-16	2.35426075	7.69E-13
Rad54b	yellow	2.19204607	4.46E-12	2.83427195	4.52E-16
9530053A07l	skyblue	-1.8801769	1.26E-10	-1.3582615	4.81E-06
Gm5150	yellow	3.36292494	2.15E-08	2.63862801	6.89E-06
Gm9733	yellow	2.9453899	1.62E-06	4.27864004	9.68E-10
Dact3	red	-0.7638837	0.00035013	-0.3962735	0.08559359
Eif5a	orange	1.08270324	1.51E-07	0.98509149	3.05E-06
Hist3h2a	skyblue	-1.3065461	1.73E-05	-1.270758	5.13E-05
Igtp	yellow	4.08078597	2.48E-68	4.00681014	5.54E-66
Zfp931	red	-1.5124457	0.00012091	-0.7623128	0.06801689
Gm14322	red	-0.8830291	0.00738014	-1.0045675	0.00231722
Gm14420	red	-0.8221183	0.00052341	-0.7228367	0.00318648
Gm14418	skyblue	-1.8902776	2.99E-05	-1.1885686	0.00701185
Gm14305	red	-1.2142598	0.0011979	-0.5207426	0.19566152
Ifi47	yellow	3.85284026	2.41E-34	3.29617708	7.96E-26
Tgtp2	yellow	3.32652498	4.28E-73	3.19128259	1.41E-73
Tgtp1	black	5.07803318	2.55E-62	5.12003124	2.04E-63
Cdc20b	yellow	1.84421087	0.00010843	3.76018558	1.93E-11
Naip6	yellow	2.1388459	1.15E-09	1.96100819	4.09E-08
Naip2	yellow	2.2312572	6.15E-09	2.09733316	8.27E-08
Arhgap8	orange	0.88229128	8.07E-10	1.02264434	1.27E-12
Hsbp1l1	skyblue	-0.5263829	0.41385285	-1.9502872	0.00224125
Ces1b	red	-1.1808238	0.00528786	-1.0060506	0.02218714
Sec61g	orange	0.81882065	4.42E-05	0.81824557	6.70E-05
Zfp429	orange	1.11347973	1.74E-06	0.69653207	0.00452887
Zfp456	orange	1.42781461	0.00328776	0.76965824	0.15183825
Serpina3m	black	5.0615289	2.36E-10	4.84055759	2.07E-09
Serpina3i	black	4.95895008	8.00E-34	4.63393091	1.17E-32
Ifi27l2a	yellow	2.70162387	2.32E-40	2.81332593	1.63E-43
Ly6c1	yellow	2.47788084	4.74E-81	2.11210822	4.88E-59
Slc45a4	red	-0.5952172	2.15E-05	-0.6244817	1.15E-05

Nrp	orange	0.71627315	4.77E-05	0.61517325	0.00071645
Gm14010	black	2.90358422	0.0003692	5.72188557	7.84E-05
Slc8a3	skyblue	-2.1246067	1.17E-06	-1.7391039	0.00013659
Capn3	red	-1.1531409	0.00282901	-0.2421315	0.59792707
Atxn7l1os1	yellow	2.83429948	0.00078984	1.51410863	0.06959608
Cd209g	skyblue	-2.9282437	0.00090834	-3.1997099	0.00159781
Zan	red	-1.3126658	0.00174315	0.14909297	0.79041872
Gzmc	black	8.92288893	3.42E-12	7.92758571	1.33E-09
Psme2	yellow	2.21530149	7.57E-24	1.99364094	2.40E-19
Zfp664	red	-0.7091103	2.00E-08	-0.5203749	6.93E-05
Gm9531	orange	1.57200957	0.00879543	0.69095212	0.30542529
Ccr5	black	5.09257404	2.59E-43	4.92371857	2.34E-40
Ccdc13	skyblue	-1.9567844	3.18E-11	-0.8158601	0.00553648
Xirp1	red	-1.387131	4.11E-07	-0.8241095	0.00422925
Gm5930	skyblue	-4.398783	0.00059627	-2.6039532	0.01510943
Tmem233	skyblue	-2.9491867	1.39E-09	-1.9394111	0.00014994
Clec7a	yellow	2.22503598	6.96E-08	1.98732236	2.52E-06
Klrb1b	orange	1.26567089	1.71E-05	0.79544499	0.0106863
Gm20661	red	-1.075104	0.00020629	-0.0172045	0.96177047
Ifit1bl1	yellow	3.88218993	3.99E-40	3.49267837	4.44E-33
Ackr4	skyblue	-3.8259719	3.61E-12	-2.2657375	5.65E-05
Gm43302	black	4.36957892	0.0004326	4.56663227	0.00031775
Gbp4	yellow	3.53970084	2.47E-53	3.44409607	1.60E-50
Gm11110	orange	1.31013617	0.0064974	1.57718523	0.00204486
Ms4a6c	yellow	4.30588994	2.97E-23	4.05810464	1.46E-20
Arpc4	orange	0.9930092	4.72E-08	0.75465754	6.02E-05
Neu2	skyblue	-3.4214999	8.35E-14	-1.9878606	2.10E-05
Kcnj13	skyblue	-2.1802132	0.00017283	-1.3797583	0.01520635
St6galnac4	orange	0.62993476	0.00041375	0.74047181	4.38E-05
Cldn34c1	skyblue	-1.3565953	0.00173129	-1.2637969	0.004739
Gm7609	yellow	2.52568609	0.00278801	2.49543267	0.00125425
Pin4	orange	0.80577082	1.11E-05	0.35040935	0.07946515
Phyhd1	orange	1.06044529	0.00066998	0.47973853	0.15033939
H2-T10	yellow	2.40826981	3.63E-42	2.31452674	9.03E-39
1700101E01R	skyblue	-2.4098951	2.45E-20	-1.3015475	3.60E-07
Gm11131	orange	1.01290796	1.17E-07	0.5161756	0.01220442
Apoo	red	-0.5998255	0.00218258	-0.3747728	0.0754232
Tmsb10	orange	1.85877902	2.24E-17	1.6767836	3.84E-14
Gm5640	skyblue	-2.6758111	0.00415584	-3.1245451	0.00777011
H2-DMb1	orange	1.7575314	4.81E-16	1.50322214	9.07E-12
Kifc1	yellow	3.02025938	4.49E-26	3.37759205	6.05E-31
Haus3	orange	0.92821571	1.26E-05	0.95030597	1.29E-05
Gm684	red	-1.1164376	0.0004885	-0.3964662	0.26039204

Pglyrp2	yellow	2.6658106	3.92E-24	2.65275944	4.70E-23
Tmem182	skyblue	-1.2137131	0.00027071	-1.4631455	1.74E-05
Gm5483	yellow	4.10996895	0.00013175	3.37476444	0.00106998
Muc4	orange	0.63666848	0.04496183	1.30238021	2.65E-05
Tm4sf19	black	4.88392485	0.00022124	5.342237	0.00032114
Gm17193	orange	1.73196597	0.00275606	0.6393053	0.2854249
Gm17396	red	-0.7005851	0.02266184	-0.8277486	0.00858253
2610203C22	red	-1.5321276	1.90E-05	-0.3904344	0.30702039
Ulbp1	orange	1.62743516	0.00947739	1.74623717	0.01258099
LOC1026380	yellow	1.41261622	0.04907082	2.92180494	0.00020686
Xlr4a	yellow	2.42033573	4.35E-10	2.52057078	3.90E-10
Klra18	yellow	2.24727062	1.03E-08	2.60060768	2.41E-10
Klra1	yellow	3.3779297	0.00053076	1.64128951	0.0679176
Gm15846	orange	1.24791653	0.11299382	1.96926439	0.00959342
Brms1	orange	0.79949727	1.10E-08	0.57622308	7.20E-05
B230307C23	orange	0.86270967	0.0071463	1.266909	0.00014097
C920021L13I	red	-1.2866181	1.21E-09	-0.9396065	1.46E-05
Rpl38-ps2	orange	0.95354033	0.00172819	0.71715198	0.02823287
Zfp36l1-ps	orange	2.66444598	0.00416551	0.08077954	0.93022563
Hist2h3c2	skyblue	-3.7111107	2.40E-06	-1.0573387	0.15094647
Gm11400	orange	1.24504505	0.0002721	1.29008826	0.00016175
Bambi-ps1	yellow	2.23426543	4.11E-07	2.88387197	3.85E-09
Gm14760	yellow	0.80010035	0.38760603	3.46332412	0.00367316
LOC1052442	orange	1.25918691	6.39E-06	0.19372445	0.56379583
Gm15821	orange	1.68421082	7.50E-09	1.74692258	1.07E-09
Slc48a1	red	-0.6783269	2.16E-10	-0.5158788	2.83E-06
Pira1	yellow	3.4375982	1.96E-11	4.29008596	3.53E-15
Fzd10	red	-0.3602581	0.35537382	-1.1716589	0.00284006
Pira6	orange	1.72995419	0.00124072	1.90999234	0.00051781
Gm12216	orange	0.34576138	0.04394768	0.60384215	0.00039464
Snrpert	orange	1.28100053	1.72E-05	1.03394821	0.00084908
Gm11585	orange	1.07832184	6.40E-05	0.79645842	0.00520319
Dnmt3c	orange	1.86192457	0.01212084	2.00859834	0.00937673
Gm13655	skyblue	-1.7717681	0.0097492	-1.4133652	0.05030923
Gm16464	yellow	2.40675867	0.0002377	3.18772651	6.45E-06
Gm14276	orange	1.42225634	0.00373879	0.90419004	0.08420677
Gm12250	black	5.31687783	1.34E-69	5.39716724	1.23E-71
Gm8822	orange	1.44472521	9.53E-05	1.61725998	6.38E-05
Gm11425	black	6.53988327	2.67E-05	5.62489892	0.00055125
Gm13204	orange	0.68860972	0.14332411	1.36232279	0.00287927
Gm15056	black	7.5893047	3.50E-08	6.48398053	4.64E-06
Gm15382	skyblue	-2.0927211	4.88E-05	-1.1784208	0.02434402
Gm11427	yellow	2.26088923	4.06E-09	2.73209048	1.28E-11

Gm15575	skyblue	-1.2894519	0.15006605	-3.0929761	0.00542791
Ctsf	skyblue	-1.5201427	1.21E-05	-1.5577953	1.12E-05
Gm13688	skyblue	-3.4666248	0.00413776	0.37343302	0.74028924
Gm16016	red	-0.4781158	0.17464819	-1.0935375	0.00151223
Gm13340	red	-0.6854112	0.00136761	-0.5362828	0.01519941
Gm11771	skyblue	-2.2995078	0.00226069	-1.190463	0.13502123
Zfp133-ps	skyblue	-0.9592628	0.18800945	-2.615513	0.00359195
Gm13436	orange	0.88507235	0.00012291	0.54399946	0.0302225
Ccnb2-ps	red	-1.3427415	0.00203644	-0.7946404	0.08304693
Hmgb1-ps5	black	4.71061114	1.17E-10	3.74253312	4.47E-08
Gm15712	skyblue	-2.4232599	0.00069666	-2.1394877	0.00329615
Gm15696	orange	2.3981115	0.00043279	1.25547823	0.06516309
Gm11478	orange	1.15096297	0.00087454	0.61927329	0.10024249
Rpl3-ps1	orange	0.61763966	0.00062523	0.23000535	0.25899394
Znf41-ps	orange	1.3230285	0.00127569	1.17576128	0.0067863
Gm22107	orange	1.8558781	0.00675846	0.62620783	0.37511766
Gm15472	black	5.71687578	0.00055933	5.69304759	0.00084908
Gm2568	skyblue	-0.5095887	0.48733441	-2.0836247	0.00357935
Mir142hg	yellow	2.05553386	1.06E-21	2.89485403	3.41E-35
Gm14321	orange	1.47664158	1.62E-05	0.63138997	0.06041801
Gm15232	yellow	2.91243336	0.00014762	2.13765803	0.01827974
AI847159	yellow	1.5522543	0.02320974	2.91273629	0.00074802
Gm14097	red	-1.7158235	0.0070713	-0.3015791	0.70846165
A730011C13	orange	1.14178682	0.00062588	0.88439683	0.0100529
Ccdc85c	red	-0.6321533	4.81E-07	-0.324602	0.0145661
Gm281	skyblue	-2.8981604	2.24E-11	-1.3918455	0.00125378
Foxo6os	skyblue	-4.6071346	1.48E-09	-3.5378749	2.84E-07
Gm830	skyblue	-2.1551367	0.0002249	-2.9913823	3.88E-06
B430010I23F	skyblue	-2.8612398	1.60E-10	-2.2542877	8.18E-07
Gm15503	yellow	3.06189254	0.00119399	1.89474349	0.04920528
Gm16267	yellow	2.0192045	0.01235483	3.93467585	0.00014448
Gm14011	orange	1.92004951	0.00619737	-1.2613127	0.11841076
Gm13111	skyblue	-3.1090905	5.66E-09	-1.822524	0.0007893
Rptoros	skyblue	-1.4109391	0.00027078	-1.401953	0.0003913
4931413K12I	orange	1.09977175	0.00073263	0.92974981	0.00798778
Mir22hg	orange	1.17284187	1.78E-15	0.94670299	3.06E-10
1110018N20	red	-1.7326061	0.00588421	-0.3905737	0.59206404
Snhg15	orange	1.54263376	1.05E-06	1.58688471	1.02E-06
Gm10785	orange	1.11719358	0.00753898	0.41389644	0.36866398
D830026I12F	skyblue	-1.2304535	7.01E-05	-1.4000802	9.00E-06
AI838599	skyblue	-1.7541343	8.50E-06	-1.6076856	7.07E-05
Gm12576	skyblue	-1.9395206	0.00027252	-0.6123689	0.28597331
Gm16151	skyblue	-2.6053405	1.85E-05	-1.3361665	0.02257463

Gm15663	skyblue	-1.6084238	1.01E-06	-1.0745517	0.00215956
4930430E12I	black	7.77118346	4.78E-14	6.44338355	9.15E-17
Gm15964	orange	1.71065756	0.00358449	0.44766261	0.4954307
Gm16188	skyblue	-3.8190577	0.00050313	-1.3617078	0.07925375
Snhg17	orange	0.59774069	0.00296139	0.60429274	0.00337699
Gm16150	skyblue	-1.2732713	0.01327516	-1.4915995	0.00454262
Gm13056	red	-1.1533814	0.0033239	-0.6715223	0.12497183
Foxd2os	orange	1.199822	3.93E-05	1.59577612	6.70E-08
Gm14319	yellow	2.18718602	0.00810932	1.56614556	0.06130152
Gm11734	skyblue	-2.4107637	5.56E-05	-1.1975927	0.03050578
1110046J04F	red	-0.9713561	0.00047811	-0.8502055	0.00376368
Gm15347	orange	1.62957536	0.00260161	1.61893222	0.00257944
4933423P22I	orange	1.7454326	0.00789379	0.89771434	0.19035268
Gm11772	orange	1.25115006	0.00025657	1.06820797	0.00314116
Gm11738	black	2.19347397	0.08078437	5.3566846	0.00116075
Bcas3os2	skyblue	-2.797159	1.31E-05	-1.7621853	0.00335032
Gm12963	red	-1.2272046	0.00376526	-1.0141197	0.0229979
Gm13166	yellow	3.18042521	0.00123761	0.86151025	0.4781091
Slc36a3os	black	4.72403803	8.37E-08	4.28677664	3.65E-06
4930412C18	skyblue	-2.0231465	1.04E-05	-1.65426	0.00037152
Gm15721	skyblue	-4.4483875	0.00420018	-2.8447087	0.01706197
A730017L22F	red	-0.7883604	0.00166387	-0.3900891	0.14098687
Dhx58os	yellow	1.77948519	8.26E-05	4.37585634	2.09E-11
Gm15410	skyblue	-3.5946859	0.00128285	-2.0888707	0.03143013
4930469K13I	yellow	4.06464393	0.00372862	2.65406236	0.01664094
Mecomos	skyblue	-0.7353561	0.23216186	-1.792853	0.00355323
Gm16046	red	-0.9959343	0.00051144	-0.6011684	0.05005661
D630024D03	skyblue	-1.6446434	4.52E-07	-0.7938878	0.02001059
Gm15987	orange	0.91227035	0.06233224	1.91847049	0.00015121
Lin52	red	-0.5804758	0.00560596	-0.6943409	0.00112768
Gm15912	skyblue	-1.360045	0.0001164	-1.2297197	0.00087947
2610507I01F	skyblue	-1.7355474	2.37E-25	-1.4504667	7.70E-18
Arhgap27os3	yellow	3.0839583	8.95E-23	3.3778626	2.88E-25
Gm15832	yellow	3.00924281	1.42E-21	1.96713904	1.37E-10
Gm15601	orange	1.20846633	5.34E-05	1.34014081	7.01E-06
2610307P16I	red	-1.5868514	4.06E-08	-0.4244122	0.16757126
Cd63-ps	orange	0.77777236	0.02455327	1.11165544	0.00155714
2310014F06I	red	-1.3128599	0.00025377	-1.1045166	0.00368398
4930481B07I	red	0.04193767	0.95229299	-1.6289722	0.00990294
Syna	red	-1.0953685	0.00577922	-0.1657865	0.72675792
Gm15904	skyblue	-0.7809691	0.35856729	-2.4922226	0.00276692
Gm15411	red	-1.3922756	0.00046662	-0.7281441	0.07396877
Gm13816	skyblue	-2.6431169	6.72E-05	-2.2303066	0.00064558

Gm5970	black	5.7576616	1.69E-38	6.78449009	3.78E-41
Gm16731	yellow	3.68427121	0.00401995	1.76005882	0.11576336
B230206L02I	skyblue	-2.0624104	0.00046901	-0.0859462	0.90327775
Gm13293	orange	1.85477033	1.18E-05	0.72000249	0.09990326
Wipf3	red	-0.3713062	0.06312143	-0.6274287	0.00139968
Gm12473	red	-0.8525851	0.00109432	-0.7224597	0.00775989
Unc45bos	red	-1.102576	0.00098873	-0.2981845	0.42587748
Gm11636	skyblue	-3.1044928	0.00074187	-1.0793148	0.23481863
Gm15326	orange	1.05843922	0.07947797	1.81241212	0.00227861
Gm2415	red	-1.291158	5.42E-08	-0.7981488	0.00124285
9030622O22	skyblue	-1.6990145	7.65E-07	-1.0065724	0.00263627
Bach2os	yellow	2.88446702	0.00040781	2.57418406	0.00283329
Ccpg1os	skyblue	-1.630807	2.73E-12	-1.058208	9.80E-06
A330040F15I	yellow	2.38802773	7.10E-06	2.00127887	0.00017631
Gm7846	orange	1.80222188	0.00743852	0.97940647	0.16833706
Tmcc3os	skyblue	-2.6115673	0.00327965	-0.6750335	0.49982382
2810433D01	skyblue	-2.2381549	0.0001698	-0.5443186	0.42090669
Snhg12	orange	1.07241673	3.58E-08	0.87466089	1.30E-05
Gm12840	skyblue	-2.9491291	0.00188125	-1.0735186	0.23991752
Gm15998	skyblue	-1.6648579	0.00799745	-0.6643796	0.32723344
Gm16144	orange	0.79370365	0.15229502	1.5174568	0.00674059
Prkag2os1	red	-0.6562008	0.11089864	-1.1263528	0.00953426
Gm11707	yellow	2.78346355	0.00042643	1.98206919	0.01519938
Adap2os	orange	1.2861541	0.00040656	1.05519329	0.00527732
9130208D14	yellow	3.24960877	9.46E-07	2.4201025	7.59E-05
Erich2os	skyblue	-2.3144606	0.0046003	-1.4251639	0.08823704
Gm15856	orange	2.17788403	0.0058461	1.02875275	0.20892996
Cd101	orange	1.57126972	0.00046055	1.71946885	0.00019522
Gm2830	red	-0.7109507	0.10471332	-1.4777537	0.00061146
Gm15500	orange	0.72530558	0.00023377	0.44265098	0.0351455
Gm12002	skyblue	-3.5273134	0.00520687	-2.1057046	0.13092236
Dnah2os	orange	1.74406975	0.00166573	0.64167749	0.2944033
Susd5	skyblue	-1.8757796	7.50E-06	-0.9284523	0.02800387
Gm15609	orange	0.69781152	0.02110268	1.27177871	2.77E-05
Gm14029	yellow	3.48446407	1.82E-11	4.5780951	1.95E-10
Gm15222	red	-0.8764985	0.00049873	-0.8191309	0.00168849
Gm11827	orange	0.96171632	0.00901244	-0.6037916	0.10171977
5330413P13I	skyblue	-2.7385333	2.89E-07	-1.2393175	0.01232653
Gm15675	red	-0.8637253	0.00047433	-0.3633932	0.1820233
2410006H16	orange	1.02848976	5.34E-09	0.72382511	8.46E-05
E030013I19R	skyblue	-2.9651089	1.79E-13	-2.4631526	4.01E-10
A430108G06	skyblue	-3.6589124	2.10E-08	-0.910953	0.0959083
4930512H18	yellow	3.29747283	0.00016974	2.05308481	0.00333115

Gm15883	skyblue	-1.9372662	5.83E-06	-0.9560108	0.03349515
A230072C01	red	-1.2883335	4.17E-07	-1.0247697	0.00010373
4930429F24I	red	-1.3167089	5.20E-07	-0.746028	0.00769887
Gm13835	orange	1.08321586	0.00154217	0.62321618	0.09449884
4930481A15I	skyblue	-1.5310286	2.30E-07	-1.1813085	7.08E-05
Gm12596	yellow	3.07534622	0.04125803	4.34022497	0.00909523
4933431E20I	skyblue	-1.8359459	0.00144293	-1.0505176	0.08717408
4930443O20	skyblue	-0.9541861	0.31779786	-4.2202203	0.00162753
Gm15475	skyblue	-2.3442749	2.22E-08	-1.480783	0.00029471
Gm13889	orange	1.91223618	2.82E-09	1.69066395	2.70E-07
2610027K06I	red	-0.8799791	0.00022267	-0.9937755	3.80E-05
Gm13387	orange	0.62203914	0.07402882	1.45757671	2.09E-05
Gm11611	skyblue	-1.800262	3.81E-05	-0.5950144	0.2071815
Gm8093	yellow	2.90798235	0.0014231	3.11573513	0.00295861
Eldr	orange	2.01257572	3.86E-05	1.21502512	0.01944938
AI662270	yellow	2.9904211	3.08E-14	2.75218214	3.80E-11
Gm11714	yellow	2.75215198	0.000194	2.29428521	0.00490076
Gm16316	red	-0.4942163	0.44215165	-1.4749065	0.00774184
Plcxd2	orange	1.61254473	2.07E-15	1.34511405	8.96E-11
A830082K12I	skyblue	-1.7596787	1.15E-06	-1.8480639	2.52E-07
2010001A14I	red	-0.7299584	0.00891796	-1.1558468	6.89E-05
Gm15133	yellow	2.64252548	2.48E-07	3.3521009	4.57E-08
D230022J07I	skyblue	-2.2100399	0.00111521	-0.0318115	0.97213252
A230056P14I	skyblue	-1.8851037	6.71E-06	-1.2869036	0.00278619
2810408I11R	yellow	3.04125699	0.00735867	2.19519174	0.01402834
Gm11377	skyblue	-1.0380155	0.00356081	-1.4263663	0.00043567
E230016M11	skyblue	-1.9902888	0.00101436	-0.6079319	0.36343285
Gm15990	yellow	2.37593283	0.00025075	3.08592	6.46E-05
2610035D17	red	-1.2796219	0.00013201	-0.9274149	0.00789192
Gm15726	yellow	1.60714564	0.01718787	2.67702759	3.86E-05
Gm16015	orange	0.90860494	0.00820335	1.58189804	2.17E-05
4933424M12	orange	2.46542802	0.00372694	0.32872813	0.75594527
Gm13556	yellow	2.78828494	0.00327624	1.79728517	0.06070727
C430049B03	skyblue	-1.6260547	3.36E-09	-0.9849194	0.00084672
BC065397	red	-1.1333619	0.00665796	-1.2583363	0.00353478
Kantr	red	-0.6044049	0.00177329	-0.7806672	7.46E-05
Cers1	skyblue	-1.4287312	0.00250198	-1.6914246	0.00069667
2310065F04I	skyblue	-3.0251501	2.51E-05	-1.6845686	0.02800387
4930577N17	orange	1.75167288	0.00379644	-0.0691076	0.92171684
Ppp1r18os	orange	1.85972555	0.00019371	2.01911	8.37E-05
Gm14286	orange	0.56262519	0.02967973	0.86845275	0.00095206
Gm13822	black	5.64519459	5.03E-05	3.83685937	3.30E-05
2900089D17	red	-1.0128393	0.00215234	-0.8837255	0.00993985

Gm16091	orange	1.41996917	7.11E-09	1.09315765	9.89E-06
9830144P21I	orange	1.31177347	4.33E-07	0.9223887	0.00028589
Zmiz1os1	orange	0.92912372	0.00771333	0.13239767	0.76820904
Gm16576	red	-0.6948549	0.0033358	-0.6004568	0.01501586
Hectd2os	skyblue	-3.109504	0.00189863	-1.3925641	0.17198126
Epb41l4aos	orange	0.73570771	0.00214322	0.20038514	0.46994219
Gm16174	yellow	2.34565724	4.47E-06	2.16889007	1.59E-05
Zfp111	red	-0.7016471	2.48E-05	-0.4693384	0.00739246
4930458D05	red	-0.5559448	0.39053593	-1.5706156	0.00936527
1500009L16F	orange	1.25754731	0.00021808	1.0415103	0.00322899
Hotairm1	red	-0.9745769	0.0030076	-1.0455984	0.00178994
1200007C13	yellow	3.27955048	4.00E-05	3.39831991	1.36E-05
Gm15283	yellow	1.82441352	0.04988321	2.95539561	0.00130839
Gm26280	orange	0.1774961	0.80981358	1.57684062	0.00940046
Gm22079	yellow	2.60139885	0.00049247	2.39465049	0.00062587
1700058P15I	orange	0.84700815	0.01186374	1.22493352	0.00026475
Tnfsf13	red	-1.0186733	4.01E-05	-0.7579228	0.00323536
Gm4477	skyblue	-1.6673878	6.23E-05	-1.8027894	3.34E-05
Cbx6	red	-0.7233445	1.98E-06	-0.7266813	2.83E-06
Mir17hg	orange	0.98041849	6.61E-07	0.6210083	0.00307834
Klra8	orange	1.79736762	7.64E-06	1.56550306	0.00016462
Tgfbr3l	red	-1.0620875	0.00012069	-0.4012536	0.17424625
Ier5l	orange	0.98212841	0.00177008	0.85901362	0.00823899
Slc5a3	red	-1.2313266	1.87E-12	-0.9683649	1.45E-07
Gm15684	yellow	2.13168607	0.0069049	1.98409313	0.01490247
Gm16136	red	-0.88923	0.00067608	-0.8103153	0.00265129
Shkbp1	orange	1.05879363	1.68E-12	0.77263862	6.09E-07
Zfp882	skyblue	-1.6026645	1.57E-06	-1.2803534	0.00019111
9230117E06I	skyblue	-4.9044018	4.13E-08	-2.9155979	1.75E-05
Etohd2	orange	0.8025171	1.43E-05	0.46731422	0.01670614
Gm16184	orange	1.8125506	0.00141301	1.45396073	0.01096232
Bcl2a1b	yellow	3.56841476	1.81E-14	3.0012903	3.90E-10
Gm8459	black	5.65624123	9.45E-05	5.03543057	0.00083838
Pira2	yellow	2.50448421	0.00019112	2.89773131	2.55E-05
Gm15446	yellow	0.00643434	0.98671399	5.80891792	1.21E-21
Gm16578	black	3.30731876	0.0324857	5.04166163	0.00267716
A430072P03I	yellow	1.78178557	0.0453821	3.02528116	0.00363331
Galnt6os	yellow	2.26585158	0.01128229	3.07321275	0.00076635
LOC1026343	skyblue	-1.8635986	5.12E-06	-1.2196555	0.0045069
Srpx	red	-1.0604784	6.94E-08	-0.5506616	0.00728294
AI480526	red	-0.8161793	1.97E-07	-0.6256794	0.00010815
Alms1-ps2	skyblue	-3.0990125	0.00020996	-2.192246	0.01672506
Ttbk2	red	-0.624286	0.00318259	-0.6305342	0.00366278

Nhlrc4	skyblue	-2.5829074	1.82E-10	-1.3003198	0.00122584
Ugt1a7c	orange	1.19752899	6.08E-05	0.7896647	0.01379254
Pou3f1	yellow	2.0298679	0.001272	2.02340158	0.00206965
Uba52	orange	1.24154495	0.00104497	1.78303706	3.69E-06
Gm614	yellow	2.9579276	2.94E-05	1.96749625	0.00323895
Ugt1a6b	skyblue	-1.1332607	0.13046927	-2.1939451	0.00436579
Acad11	red	-1.3527059	4.05E-09	-1.0732921	5.46E-06
Fbxw10	yellow	1.80787808	1.28E-05	2.22544679	2.66E-07
Gm16556	black	5.92796915	0.00021628	2.90435654	0.02491181
Tepp	skyblue	-2.0675943	0.00026392	-2.6276945	5.86E-06
Itga10	red	-0.8959111	0.00023821	-0.8807356	0.00041434
Tmem189	orange	0.86588838	9.97E-09	0.65870318	2.46E-05
Trim34b	orange	1.97120719	0.00048614	1.08943186	0.05994608
Cfb	yellow	2.79412326	2.53E-09	2.82901492	3.68E-09
Car15	skyblue	-1.5232212	0.00944699	-1.2662098	0.02992078
Gm17017	yellow	2.17924394	4.55E-09	2.24740828	8.22E-09
Alms1-ps1	skyblue	-3.1876925	5.28E-05	-1.7161234	0.01972635
Gm4524	skyblue	-2.7419871	2.93E-05	-2.3088509	0.00060538
Mndal	orange	1.93924921	8.72E-39	1.84620849	7.44E-35
Gm17296	red	-0.4465901	0.01493726	-0.6336954	0.00057041
Lrrc10b	skyblue	-2.1381429	5.12E-13	-1.0821902	0.00040669
1700071M16	yellow	4.0430522	3.75E-06	3.86230829	2.75E-05
2310050C09	skyblue	-2.746961	0.00051079	-0.9680077	0.25564871
4932443I19R	skyblue	-2.2720932	1.23E-06	-1.180444	0.01639787
Gm8229	skyblue	-4.3040544	0.00137125	-2.4171954	0.02641095
Gm38399	orange	1.10533418	0.0006805	1.17317872	0.00050004
Sfta2	red	-1.3257443	0.00010139	-0.7896545	0.02828334
Rps11-ps1	orange	0.41420879	0.58925082	2.0210186	0.00936307
Snrpe	orange	1.09056167	5.79E-12	0.89013299	5.54E-08
Olfr753-ps1	black	4.97695272	0.00051875	4.30209127	0.00114443
A430057M04	yellow	0.8649555	0.28263289	2.60706374	0.00245148
Tex9	orange	0.41936888	0.05624976	0.67387541	0.00182277
Olfr456	skyblue	-2.4761347	0.00058896	-2.3939731	0.00154658
Zfp712	red	-0.7347748	0.02807011	-0.8840976	0.00977621
Gm17182	skyblue	-2.1753252	0.00213894	-0.3292747	0.68630782
Gad1-ps	orange	2.290435	0.00218176	1.29741169	0.09735954
Gm340	orange	2.091754	2.05E-10	1.4090071	2.44E-05
Gm17082	orange	0.50016463	0.09996348	0.93667788	0.00396443
Apold1	orange	1.3332619	3.28E-07	0.90133905	0.0007018
Gm17173	yellow	3.17508611	0.0236428	4.19457744	0.00748543
Gm17201	orange	0.59097503	0.00130936	0.67183982	0.00045134
Klhl33	skyblue	-2.1366729	9.41E-06	-1.5732697	0.00176878
Trbj2-6	yellow	1.58330474	0.01865955	3.38628677	6.49E-06

Rps13	orange	0.75396214	1.07E-10	0.54061102	7.83E-06
Gm17112	red	-0.8013758	0.00718933	-0.1745864	0.6158187
Hspa1b	skyblue	-2.8052625	1.28E-06	-1.8599668	0.00206965
Gm6904	yellow	4.19126654	6.50E-09	3.25325743	3.67E-06
Pabpc4l	skyblue	-1.5459221	0.00792178	-2.8926878	5.30E-05
F830016B08I	black	4.57461353	3.08E-37	5.67009975	4.57E-39
Ccdc71l	orange	0.58809881	0.00247309	0.38233008	0.06444527
Phf11c	yellow	3.09241693	2.99E-34	2.8076818	8.12E-29
Gm17334	orange	2.0730583	3.80E-15	1.72345806	2.44E-11
Smim13	red	-0.6261104	1.20E-05	-0.3983287	0.00829384
Col6a5	skyblue	-2.160279	2.38E-05	-1.1043287	0.03771612
Ak9	skyblue	-1.8914699	2.09E-07	-1.4975733	5.81E-05
Vmn2r97	yellow	2.76302568	5.89E-11	2.15690497	3.35E-07
Mpc1-ps	red	-0.32626	0.18157118	-0.7007148	0.00341687
Zfp422-ps	red	-1.2408265	8.98E-05	-1.1166767	0.00150002
Tma7	orange	0.68719101	3.31E-07	0.58337362	2.62E-05
Gm6548	orange	1.46080418	5.68E-23	1.44687843	1.88E-22
2010016I18R	yellow	1.9759169	6.99E-10	2.04073663	3.00E-10
Lsm5	orange	0.96511834	1.82E-06	0.62693042	0.00368655
Phf11b	black	4.52113562	2.42E-47	4.37502389	6.01E-44
Apol11a	black	3.48039755	0.01010945	6.06123796	0.00038787
Vmn2r96	yellow	3.74424124	0.0029843	3.37271587	0.00610392
Apol11b	orange	2.20065737	2.40E-06	1.28768574	0.00857129
H2-Q2	orange	0.36230294	0.29093112	1.39064774	1.35E-05
Sec14l5	skyblue	-2.43659	6.23E-07	-1.8701399	0.00020385
Gm17098	skyblue	-2.2913971	0.00022412	-2.5819998	3.00E-05
Zfp964	skyblue	-1.4339805	1.48E-06	-1.9367644	2.32E-10
Tnnc1	skyblue	-0.9581105	0.18894541	-1.8728697	0.0085729
Gm9844	orange	1.8041248	9.50E-07	1.38680465	0.00042921
Rps2-ps10	orange	1.31882085	7.60E-05	1.46491733	3.66E-05
Hspa1a	skyblue	-2.3429103	6.42E-05	-1.6801991	0.00590621
BC049352	yellow	2.18457755	0.03754549	3.60179721	0.0041242
Gbp11	black	5.2950623	6.53E-117	5.33194265	1.88E-121
Dynlt1a	orange	1.32724553	0.00022555	1.19542289	0.00094253
Tnxa	skyblue	-2.4155091	0.00086543	-1.2751833	0.09058412
1110038B12I	orange	1.69009313	5.67E-14	1.50296047	3.67E-11
Gm19345	black	4.6456369	3.24E-07	3.80362897	3.66E-07
Gm20519	yellow	0.66328824	0.60629976	3.46845616	0.00736785
Neat1	red	-0.6173848	1.10E-05	-0.3224738	0.0310342
Gm19684	orange	2.02920347	3.39E-06	1.7003005	0.00015541
BB365896	skyblue	-2.3764963	0.00055555	-2.4742269	0.0004034
Platr17	yellow	2.44457665	0.00685358	2.73752434	0.00535967
C130080G10	skyblue	-2.8068054	0.00041713	-1.8007221	0.02570487

Vhl-ps1	black	5.44395709	0.00015864	5.26973666	0.000491
2610524H06	orange	1.68232274	1.53E-07	1.2836338	0.00012494
Gm20394	yellow	2.26870977	2.56E-05	2.42292072	2.85E-05
Art2a-ps	yellow	2.80338587	0.00022348	3.10698892	0.00050854
Nlrp1c-ps	yellow	3.29079694	1.09E-07	4.10854456	8.96E-10
Gm20496	yellow	3.14072519	1.08E-06	2.7815003	4.05E-07
Btnl6	black	5.94486497	0.00030608	5.84589134	0.00057319
Gm22858	yellow	0.97655874	0.27976058	2.64216439	0.00771822
Mir5107	orange	0.96743735	0.00438902	1.56772988	6.23E-06
6330562C20	orange	1.67250045	0.00478633	1.0561527	0.09775694
Lrch4	orange	1.56652512	5.41E-07	0.82713199	0.01142688
Gm20703	yellow	2.33109805	0.02053594	3.94041748	0.00686211
Rpl41	orange	0.83617053	6.03E-07	0.50978244	0.00371482
Gm20717	yellow	3.16053043	2.17E-05	1.97039341	0.00228114
Gm20667	yellow	2.66340863	0.00446509	2.74162477	0.00995652
4931403E22I	skyblue	-1.791349	2.37E-06	-0.8942932	0.0203576
Trav4-3	yellow	2.90083604	0.00233038	3.58822211	0.00108671
Gm2237	orange	2.45256005	8.95E-07	0.51659799	0.30798247
Ighv1-74	black	6.44857104	0.00327746	1.39232929	0.544538
Hist1h2ao	yellow	3.83189091	5.44E-13	3.73389314	1.45E-08
Ccdc42b	skyblue	-2.507536	1.04E-05	-1.2196358	0.0297479
Gm3788	yellow	2.17420485	8.13E-05	1.8612529	0.0020003
Zfp955a	red	-0.8325129	0.00064381	-0.4401578	0.09177737
Trbv12-2	yellow	2.84766172	2.36E-05	4.50997333	1.18E-08
Trbd2	yellow	4.00652231	6.89E-06	3.30701544	4.49E-05
Cecr6	skyblue	-2.6008042	1.74E-07	-1.1864361	0.0204239
Gal3st2	orange	1.24588451	0.02053817	1.41729134	0.00708575
Trav7-4	black	4.01898102	0.00376874	4.72421915	0.00084927
Hist1h2ap	yellow	4.03181066	2.77E-06	3.34465421	0.00025018
Gm14403	skyblue	-1.9347363	2.49E-07	-1.2256843	0.00151286
BC147527	yellow	4.02587904	4.94E-29	3.82497955	4.18E-26
Trav3-3	black	4.58599377	1.65E-09	6.01182546	5.63E-07
D430019H16	skyblue	-1.3511816	1.41E-10	-1.1572419	9.52E-08
Trav9-2	black	3.06078863	0.00527813	5.36700931	0.00057921
Sirpb1b	yellow	3.14574868	8.04E-12	3.38248033	1.80E-12
AC149090.1	red	-0.8970999	9.57E-08	-0.4826537	0.00677079
Ccdc85b	red	-0.8951043	3.93E-05	-0.7454043	0.00094093
Mid1-ps1	orange	1.09826503	0.0069146	1.8540414	4.14E-06
Zfp799	red	-0.7329147	0.00464624	-0.7293753	0.00601646
Gm10130	orange	1.13422506	0.00564503	0.98036331	0.01885444
Noc2l	orange	0.87537023	1.86E-05	0.73955514	0.00046388
Ighv5-17	orange	2.45079195	0.00937356	-0.2393924	0.83795987
Trbv12-1	yellow	1.90341545	0.01137755	3.25178693	0.00013253

Fmo6	skyblue	-1.9179565	0.00062113	-0.5388411	0.35604832
Trav5-4	black	4.67058491	0.00064268	3.01904948	0.02156507
Gm21188	black	4.63803278	6.52E-18	4.37565308	1.10E-15
2010005H15	orange	1.91155439	0.00156153	0.60548389	0.39137822
Gm8979	yellow	2.87077573	0.0021359	3.67999241	0.00042273
Trbd1	yellow	3.72222938	1.95E-05	2.49196675	0.00074331
Rnaset2a	yellow	3.0821374	0.02230301	3.98533943	0.00983266
Trav14-2	yellow	3.5758347	0.00752777	3.08106212	0.00326226
Igkv4-53	yellow	4.69935478	0.00226435	1.12251032	0.52206968
Sirpb1a	yellow	4.10180039	4.02E-17	3.73329263	1.51E-14
Nupr1l	skyblue	-1.8010653	8.01E-05	-1.3789434	0.0032935
Igkv6-17	yellow	3.24930356	9.35E-10	1.96088753	0.00029507
Lce1k	red	-1.5225375	0.00010066	-0.7090091	0.09702734
Syne1	red	-0.6262413	2.84E-05	-0.6843745	6.74E-06
Trav4-4-dv10	yellow	3.47848282	0.00063448	4.24265264	0.0002455
Gm16505	skyblue	-2.6584628	0.00015283	-2.0966885	0.00201122
Ankrd66	red	-0.8273319	0.00057269	-0.9202136	0.00013044
Dnah7a	skyblue	-2.048143	2.67E-07	-1.0340543	0.00831777
Vkorc1	red	-1.1219552	0.0006144	-0.3025906	0.42393416
Kcnj11	skyblue	-1.1382934	0.01001815	-1.7985192	5.37E-05
Cmtm4	red	-0.9376798	1.64E-07	-0.6252356	0.00080168
Smim22	red	-0.7881758	0.00094166	-0.6569803	0.00940042
Ighv1-78	black	5.52568545	2.22E-07	1.71677947	0.08763667
Samd11	yellow	2.12773313	0.01412345	4.45352754	0.00019317
Trav17	black	6.10284181	0.00094566	6.01428946	0.00154658
Igkv12-44	orange	-0.0379774	0.96452357	1.93090703	0.0073327
Cdkn2d	orange	0.9526098	3.21E-09	0.87258734	1.10E-07
Trav10	black	5.4626051	0.00037902	4.94637613	0.00194824
Trav12-2	black	3.48068569	9.87E-05	7.11139772	1.20E-06
Ighv1-63	black	8.78675358	0.00561858	1.81953827	0.59653978
Trav9-4	black	5.24547215	0.00018674	4.3466221	0.0008286
Psmb9	yellow	2.46383585	1.95E-71	2.28347054	2.37E-61
Gm28373	yellow	2.76905423	2.81E-05	2.84653906	2.52E-05
Shisa8	orange	1.96701589	1.38E-08	1.86966138	1.42E-07
Trav9-1	black	5.40926836	1.71E-05	4.40896033	8.92E-05
2500002B13I	red	-0.2858466	0.47930732	-1.5483355	8.80E-05
Gm9530	orange	1.04119022	0.00654249	0.39916461	0.37653884
Gdap10	yellow	2.66817243	2.20E-10	2.43541944	8.00E-09
E230013L22I	orange	0.80240717	0.00129055	0.94709795	0.00022498
A230028O05	black	4.97062324	8.08E-09	5.49276272	4.17E-09
4930556M19	red	-1.3206714	8.52E-10	-0.766217	0.0006881
Gm26880	orange	2.26603862	0.00953071	0.00063495	0.9993873
Gm17435	orange	1.50896656	0.00997901	0.61809892	0.34795021

4731419I09F	orange	1.89854281	0.00378717	1.12729401	0.07836533
9530082P21I	orange	0.85476331	3.45E-08	0.91159043	5.92E-09
Pvt1	orange	1.41617504	0.00012504	1.59402831	3.55E-05
Gm4262	red	-1.0642109	0.00330517	-0.5000787	0.18729407
Gm26760	skyblue	-1.780946	0.03125441	-2.3450564	0.00839052
Cdiptos	red	-0.9963071	0.00011379	-1.2456774	4.43E-06
Gm10425	orange	0.47089323	0.29322657	1.17732491	0.00545456
Foxl1	skyblue	-1.9743282	4.37E-06	-0.7902237	0.0768043
Gm9917	red	-0.8702664	1.32E-06	-0.7467153	5.42E-05
1810034E14I	red	-0.7201852	0.00802666	-0.1973748	0.52506375
Gm2885	skyblue	-1.6134004	0.00556512	-0.6827724	0.26529393
Gm26580	skyblue	-1.8706932	0.00938508	-0.8335962	0.2832304
Gm19705	yellow	2.18745156	6.82E-09	1.92958034	1.74E-07
B230354K17I	red	-0.5396904	0.00568015	-0.7185321	0.0003155
A530020G20	red	-0.9349212	0.00755766	-0.6806421	0.06406441
D230017M19	skyblue	-1.3365075	0.00041812	-1.3868354	0.00044543
Gm26628	yellow	1.87915962	0.02258834	2.43460211	0.00358074
1110002J07F	yellow	2.58777248	0.00861337	1.66432263	0.11736503
Gm26510	orange	1.27672426	6.32E-05	1.14707513	0.00048391
2310002F09I	skyblue	-1.4712696	0.00626803	-1.8086094	0.00376777
2700038G22	orange	1.99392666	6.92E-08	1.65530258	1.04E-05
4632428C04	red	-1.0100244	0.00273254	-0.6724622	0.06130152
9330175E14I	yellow	3.18049304	6.18E-85	3.34711948	1.37E-91
Snhg5	orange	0.64951186	8.07E-06	0.64978688	1.43E-05
1010001N08	red	-0.4500365	0.27578783	-1.0985862	0.00594364
Gm16907	orange	1.1373409	4.65E-06	0.68153382	0.00981071
Gm26767	red	-0.7032027	0.00697174	-0.1669603	0.57184442
Gm26684	skyblue	-2.0557429	0.00418257	-1.5789122	0.0322404
2010300F17I	yellow	4.13242744	4.57E-08	3.23612315	8.04E-06
Hmgb1-ps8	yellow	2.41907816	3.14E-08	1.57119642	0.0005533
Gm17249	red	-0.3741863	0.29573491	-1.5650691	2.32E-05
Gm10516	red	-0.9567916	0.00063283	-0.9695003	0.00090016
2700012I20F	skyblue	-2.4576129	0.00025347	-0.6498122	0.27372082
Fendrr	red	-1.0519929	5.94E-05	-0.7164462	0.00894341
Gm26671	red	-1.0640163	0.00045759	-0.2573041	0.4295167
C920009B18	yellow	3.56307547	6.75E-13	2.715986	1.38E-08
C030034L19I	skyblue	-1.5057689	2.86E-05	-1.3534815	0.00025721
Gm26876	yellow	3.82254395	0.00744994	3.34254931	0.01163122
Gm26816	orange	1.27228394	0.00596062	0.88522913	0.07881701
Gm26814	skyblue	-2.2762642	0.00126373	-1.7431258	0.01594547
9230116N13	orange	1.0917284	0.00770208	0.87784587	0.04123156
AU020206	orange	1.21005845	1.50E-17	0.89006798	9.83E-10
Mir155hg	yellow	3.82704043	1.52E-13	3.95437897	3.70E-13

Gm26522	orange	1.78144546	7.30E-11	1.57408227	2.42E-07
Gm10544	skyblue	-1.8352698	0.00899939	-0.7738982	0.29257386
Gm26781	black	4.60160595	1.99E-11	4.68431538	1.43E-10
Gm16754	orange	0.6281902	0.03754443	0.85794074	0.00498625
Rian	skyblue	-2.0277183	1.55E-11	-1.3423953	1.62E-05
2310031A07I	yellow	2.13553861	0.00057185	1.78889439	0.0070147
9530026P05I	red	-1.439703	0.00347465	-1.0704155	0.03755352
D430036J16I	skyblue	-2.305565	1.86E-09	-1.9021577	5.11E-07
5830432E09I	orange	1.38861455	7.44E-05	1.75433734	2.48E-06
Gm26586	orange	1.33791774	0.00204385	1.30572928	0.00454729
9930014A18I	red	-1.021249	2.46E-05	-0.5231523	0.03858894
4933406C10	skyblue	-1.5847139	0.00220558	-1.5990619	0.00253544
4930516B21	skyblue	-1.8502972	6.78E-08	-1.4571935	3.41E-05
Gm16675	orange	1.66667704	6.81E-09	1.70945998	2.49E-09
2610037D02	orange	1.09899435	0.00477833	0.56844836	0.16827121
2610020C07	orange	0.84274581	0.12170409	1.65307912	0.00130175
Gm26902	yellow	3.10156819	1.23E-07	2.06890797	0.00028975
D430018E03	yellow	1.22480277	0.07286705	2.72437814	0.00020654
Gm26637	yellow	2.40178944	1.45E-06	2.52947038	1.83E-06
Gm26797	orange	0.97584347	0.0091564	0.8371326	0.02892434
Gm26799	yellow	1.93120057	0.00021931	2.14731208	4.91E-05
E230029C05	yellow	2.44702214	1.74E-13	2.6772309	1.18E-13
Gm26660	red	-1.1654167	4.21E-06	-0.887025	0.0011287
Gm17322	black	3.73237134	0.00085476	6.76417812	9.51E-06
A930012L18I	skyblue	-2.1346394	0.00055307	-2.0918823	0.00060172
Gm17597	skyblue	-5.0445948	0.00069806	-1.8590297	0.04282471
1110019D14	skyblue	-1.5174245	0.000154	-1.2873913	0.0020939
Mirt1	yellow	1.9793524	1.19E-15	2.43268787	7.12E-23
A130051J06I	orange	0.79845166	0.07464139	1.4901113	0.00093709
Mhrt	skyblue	-2.4120992	0.06141783	-3.6929856	0.00470638
Gm26764	orange	1.56896175	0.00626279	1.09362783	0.07958948
Gm26740	orange	1.72647865	8.79E-07	1.80714717	1.94E-06
2810429I04I	yellow	1.73389344	0.02911464	3.66607877	0.00012942
9530036O11	skyblue	-2.1763719	0.00026162	-0.824518	0.19953934
Gm26530	orange	0.56259336	0.03443613	1.15585791	1.33E-05
E030044B06I	skyblue	-2.8121261	0.00133798	-3.7391042	0.00036614
2010110K18I	black	3.02400187	0.00012391	6.76854074	1.11E-06
A730056A06I	skyblue	-4.9549543	0.0015894	-2.397447	0.04811746
Snhg4	orange	1.67432693	7.23E-07	1.61508794	2.71E-06
4833407H14	yellow	2.23856817	2.72E-12	2.43006513	1.06E-13
9330136K24I	orange	1.47379985	1.30E-05	0.5617539	0.11253107
Gm2115	skyblue	-3.0782733	9.96E-09	-1.9500561	0.00029191
Gm17259	red	-0.7256343	0.18546626	-1.3929843	0.00915621

Gm16685	yellow	2.95811972	4.65E-12	2.5693488	7.60E-09
Panct2	skyblue	-2.7250112	1.48E-08	-1.4135854	0.00313842
Gm26809	orange	0.87682421	0.00055395	0.84810241	0.00113239
Gm26813	orange	1.74315456	0.00103367	0.95101209	0.07988939
E530011L22F	red	-0.7916101	0.00178022	-0.9587442	0.0003155
Gm16701	red	-0.4356474	0.17620909	-1.1166894	0.00044375
Gm807	black	6.22271954	0.00057269	4.21901272	0.0078887
A930007I19F	orange	1.79157272	3.14E-07	1.16058594	0.0016403
Gm26603	yellow	2.13636971	0.00297617	2.31281983	0.00120538
Gm26703	orange	1.73173968	0.00012728	1.61951035	0.00047536
Gm26682	orange	1.9866879	0.00501423	1.07451233	0.13359605
Gm26541	orange	1.26851922	0.00012131	1.49788877	1.49E-05
4930547M16	skyblue	-1.9977466	0.00988938	-0.8678772	0.25771685
Gm26711	orange	1.52832994	0.04503573	2.4185947	0.00262399
A730020E08I	skyblue	-1.4568942	0.00920152	-1.1766438	0.04304778
Gm26917	orange	1.69299741	3.47E-06	1.26667046	0.0008225
Ptprv	orange	1.55968941	1.41E-05	2.21816637	4.22E-09
A930001A20I	skyblue	-3.6247028	0.00673768	-1.8098277	0.11590336
Gm26947	yellow	2.66562591	1.48E-06	2.28888673	3.87E-05
Gm6981	red	-1.4185821	0.00657305	-0.3177506	0.57184442
2700099C18	yellow	1.99649821	7.99E-08	2.25993797	9.52E-09
Bvht	red	-0.6512627	0.00010785	-0.6062409	0.00046335
Bin2	yellow	2.73307863	8.64E-25	2.61705936	1.05E-22
Rassf10	skyblue	-1.9149848	4.14E-11	-1.4521757	9.94E-07
Gm26973	skyblue	-1.7365486	1.78E-08	-0.9018229	0.00451932
Gm27010	orange	1.12609781	4.06E-08	0.80839277	0.00021774
Snhg6	orange	1.18030662	1.57E-06	1.37231606	3.72E-08
Gm27514	orange	1.59509981	0.06063266	2.12260559	0.00939572
Lockd	yellow	2.0296336	1.51E-06	2.91597246	1.74E-08
5830468F06I	yellow	1.95391144	0.05229819	2.90535574	0.00497059
Gm28043	orange	0.2526991	0.34048253	0.6580147	0.00970662
Mir6377	skyblue	-1.4126252	0.00758429	-1.5243801	0.01026243
Gm27202	skyblue	-3.0407682	0.00176022	-2.3544466	0.01254695
C1rb	orange	1.7791979	0.00065662	1.92527142	0.00039645
1500015A07I	orange	0.88166493	7.25E-06	0.64489784	0.00195526
Gm27252	orange	1.50719082	2.29E-05	0.183353	0.65492889
Gm28053	yellow	2.5321044	0.01176363	2.56772723	0.00640326
Gm18853	yellow	3.43370825	4.21E-06	2.71762673	0.00019566
Gm18852	yellow	3.48447123	9.35E-09	3.94130337	7.20E-10
2810030D12	skyblue	-1.5333992	0.00036409	-1.0602	0.01899367
Ms4a14	yellow	3.80657692	3.99E-13	3.96197629	9.23E-13
Gm17767	orange	1.19313974	0.06740411	1.72147412	0.00629144
1700097N02	black	3.05731292	0.0038024	4.93205967	0.00076689

Gm29538	skyblue	-2.0989112	5.98E-05	-1.6053155	0.00209352
Gm28729	skyblue	-2.4354297	3.10E-18	-1.4542712	3.24E-08
Gm18301	black	2.46783008	0.02137824	5.38067966	0.00025916
2310034G01	red	-0.9466745	0.00011802	-0.69794	0.00567311
Zfp383	red	-0.9716798	7.51E-11	-1.1421238	4.18E-14
BE692007	yellow	2.96618897	5.57E-23	2.82055131	1.87E-20
Gm28884	yellow	4.46538787	0.00224114	2.04792064	0.08583137
Gm5960	black	7.22940411	2.09E-06	6.93794066	8.82E-06
Gm7160	orange	0.89158237	1.17E-05	0.92783949	6.42E-06
Gm6652	skyblue	-2.9268562	1.72E-06	-1.8284113	0.00080067
Rbm3-ps	yellow	2.82847281	2.44E-11	2.4897863	5.94E-09
2810013P06I	red	-0.5822856	0.00011605	-0.43845	0.00550859
Bcl2a1d	yellow	3.287741	1.34E-17	2.62104736	2.75E-11
B130024G19	red	-0.9100946	2.37E-07	-0.9100711	6.22E-07
1700025N23	yellow	3.72762258	5.80E-08	4.03331807	1.02E-07
Gm28439	red	-0.8771905	0.05657707	-1.267518	0.00774529
Gm19585	yellow	3.91003302	3.77E-18	4.06368916	1.28E-15
2610306M01	red	-0.6709821	0.0038982	-0.5027081	0.04483809
1810006J02F	yellow	2.62525551	0.00584318	1.70854102	0.0316208
Gm28557	skyblue	-2.3115849	2.03E-06	-0.460684	0.35985836
4930422M22	skyblue	-1.792468	0.00695641	-1.1976885	0.06327165
2310008N11	skyblue	-2.5425674	1.51E-05	-2.2775872	0.00023114
Gm28935	yellow	2.08803569	0.06720707	4.98696307	0.00068918
2310020H05	skyblue	-1.7963298	0.00241959	-1.17163	0.09763628
C530043A13	skyblue	-3.3978588	1.62E-07	-0.7070267	0.17718228
Gm19261	black	3.80415779	0.00697519	5.52720948	0.00043023
Gm29585	yellow	2.37960404	2.74E-11	1.97134121	2.96E-09
Gm5511	yellow	3.72681075	0.00111807	1.08255777	0.30194464
F730311O21	orange	0.83655334	0.05951345	1.39643912	0.00172214
Gm28523	skyblue	-1.0233979	0.00771528	-1.499037	0.00019847
Gm28874	orange	1.7673554	0.00908482	0.55153045	0.47830553
Lhb	skyblue	-2.2131663	4.69E-17	-1.0890099	2.38E-05
Gm28437	red	-0.435669	0.09285364	-0.7563767	0.00381158
Eif4a-ps4	orange	1.15070145	5.21E-05	0.82678548	0.00566942
Gm28720	skyblue	-2.7566501	4.14E-06	-2.2273524	0.0002648
4930568A12I	yellow	2.91410037	1.03E-06	2.57676716	3.52E-05
Dnah7c	skyblue	-1.9832141	1.72E-11	-1.8157541	8.49E-11
Ms4a4a	yellow	4.45081868	8.03E-35	3.77934853	5.72E-26
Ace3	red	-1.1490255	2.66E-05	-1.1995393	2.10E-05
Kcnq1ot1	red	-0.5446583	0.02952908	-0.7661976	0.00241651
2310040G24	red	-1.1019251	0.00875554	-0.5819078	0.22036921
Gm19461	red	-1.2446653	0.00351905	-0.9543893	0.0303791
Gm29237	red	-1.2961568	0.00013676	-0.8303021	0.02005918

9130401M01	orange	1.15826641	0.00024875	0.55749322	0.09883937
Gm28438	red	-0.905983	0.00579719	-0.8387359	0.01348904
Bcl2a1a	yellow	2.81083339	1.49E-09	2.22782146	3.26E-06
I830127L07R	black	5.26794994	8.51E-15	5.12090897	1.64E-14
Gm20257	orange	0.93739874	9.70E-07	0.57324826	0.00376744
Gm37472	orange	0.82768865	0.00506705	0.47558229	0.14421519
Gm37468	black	5.0340901	0.00063809	4.83259308	0.00038314
Gm33280	skyblue	-2.5686163	7.09E-05	-1.4723656	0.02433686
Gm37718	yellow	2.48642241	0.00640934	2.08036431	0.06488985
Snrpn	skyblue	-1.1044539	5.19E-05	-1.6095449	1.04E-08
Gm7357	skyblue	-2.0673733	0.00076723	-1.8877632	0.00347849
Gm37626	skyblue	-0.8401043	0.19106327	-2.5574043	0.00016329
Gm19721	orange	0.57148386	0.3472391	2.03917111	0.00071118
Sh2d1b1	orange	0.77481335	0.03938638	1.01695019	0.00873157
Gm37464	skyblue	-2.7073623	8.21E-07	-1.2248362	0.02383018
Pcdhga12	red	-1.1052934	1.29E-06	-0.6768151	0.00472462
Pcdhga9	red	-1.5378545	2.07E-07	-0.2984359	0.33926395
Gm38197	skyblue	-1.8717307	3.97E-06	-1.8083556	2.11E-05
A930004J17F	orange	1.48527822	4.41E-09	1.03227594	0.00011409
Gm37289	yellow	2.74597351	0.00809747	0.97647824	0.33576029
Pcdhac2	skyblue	-2.4264737	1.13E-08	-1.7901757	3.73E-05
A630081D01	yellow	2.52543087	0.00149796	1.34615013	0.11318525
Gm7694	red	-0.6492668	0.00313457	-0.6767079	0.00260761
Naaladl2	red	-1.1141506	0.00117256	-1.2762983	0.00037344
Gm38253	yellow	1.81391041	0.03655723	2.93662215	0.00065701
Gm37795	orange	1.19726051	8.89E-07	1.10233246	9.16E-06
Pcdhgc3	red	-0.9970994	1.10E-07	-0.6053641	0.00201002
9430034N14	orange	1.32277005	0.0069977	1.65943119	0.00138757
Pcdhgb6	red	-1.008495	0.00025532	-0.7263396	0.01125906
Gm32200	skyblue	-1.61181	1.74E-06	-1.3824399	7.68E-05
Gm37387	yellow	2.34995713	5.67E-05	2.19373751	0.00022108
Gm37303	yellow	0.6050093	0.50878569	2.92031585	0.00139347
Gm37169	yellow	2.67269533	4.05E-05	2.59316863	6.02E-05
Gm37090	red	-0.6796081	0.00049566	-0.5925512	0.00372595
Gm37728	skyblue	-1.4259908	0.00251074	-1.3402771	0.00838495
Lce1d	red	-1.293508	8.26E-05	-0.8121514	0.01945891
Pcdhac1	skyblue	-3.6628274	0.00573158	-3.636747	0.00052424
BC037039	red	-1.420517	0.00306091	-0.259915	0.65344149
Gm38157	orange	1.09574133	0.00056186	0.26422058	0.5028267
Gm38158	yellow	2.59471773	8.53E-11	2.02724043	5.32E-07
Gm37581	orange	0.4002334	0.32502602	1.10364403	0.0030223
Lsmem2	red	-1.254998	0.00256393	-0.9627125	0.0341215
Gm37666	yellow	2.44916965	5.77E-11	2.25944835	4.62E-09

Pcdhga5	red	-1.0312835	9.59E-05	-0.3787337	0.16751091
Gm37352	orange	0.57013815	0.06863092	1.09394849	0.00033397
Gm37652	skyblue	-3.6029524	0.0004517	-0.9713923	0.36426265
Gm37859	skyblue	-1.4751334	0.0001516	-1.3373351	0.00081747
Gm36932	orange	1.63976488	0.00780243	1.56176072	0.0187285
Gm20056	orange	2.16544616	0.00164639	1.22826883	0.08758316
Gm37249	skyblue	-2.5947037	5.59E-05	-0.7234216	0.29251374
Gm37010	black	3.40368986	0.00051741	4.908753	0.00138173
Gm37499	red	-1.4182678	0.00835008	-0.3940497	0.51044903
Pcdhgb7	red	-1.3580991	1.83E-07	-0.7997872	0.00303939
Gm38275	black	6.57318819	2.99E-06	3.79852789	0.00445008
Gm38103	skyblue	-2.0114701	0.00713599	-2.1842699	0.00998019
Gm38102	red	-0.8749609	0.00038137	-0.5141364	0.04865699
A130071D04	yellow	2.51645287	4.54E-08	2.70625674	4.35E-08
Gm37689	yellow	4.13400184	0.00333518	2.82529241	0.00664505
Gm37691	orange	1.07852889	0.00056499	0.9708793	0.00340972
Gm38244	orange	1.21059598	0.01686171	1.38617885	0.00865688
Gm37516	red	-1.4525424	0.00318319	-1.1115609	0.03008553
Gm37033	orange	0.69081621	0.00124305	0.2665997	0.28295859
Gm37829	red	-0.5697886	0.00429387	-0.8493312	3.81E-05
A430027H14	skyblue	-1.8620196	0.01660499	-2.2088157	0.00788246
Gm33994	yellow	3.84999904	0.00042388	1.5889245	0.1334288
Gm4610	yellow	2.63726958	4.62E-06	3.24549764	4.60E-08
Gbp6	black	4.6987312	1.87E-109	4.86287289	1.03E-116
Gm43162	orange	1.25582076	0.00054146	0.54501807	0.1984893
Gm42462	yellow	3.74997623	1.77E-06	3.55244605	6.82E-06
Gm42984	skyblue	-2.1621422	0.00549979	-0.9857297	0.23940501
Gm5547	orange	1.45379188	5.64E-05	1.23159566	0.00128075
Trdc	orange	-0.0661768	0.93990005	2.42966333	0.00091315
Gm6560	orange	1.63525577	3.46E-07	1.49381357	7.07E-06
4833413G10	red	-0.9973828	0.00016614	-1.0818687	4.56E-05
Gm43437	skyblue	-1.9836933	4.70E-07	-1.6655487	2.08E-05
Gm43423	red	-0.3368652	0.49822769	-1.5568449	0.00098955
Gbp10	black	6.03886169	1.28E-72	6.26011372	3.54E-79
Gm43667	skyblue	-1.7513217	1.00E-06	-1.5904494	2.55E-05
Snord43	yellow	2.79017685	0.00360839	1.873651	0.03622773
Gm43668	red	-1.1490196	0.00018765	-0.7163373	0.0338654
Sox2ot	skyblue	-3.7955305	2.58E-06	-0.7963362	0.21861804
Gm43071	orange	1.1386897	0.00067256	0.59084018	0.0989326
Gm43719	skyblue	-1.5175351	3.15E-07	-1.5282426	8.74E-07
Gm42515	orange	1.98403145	0.00097826	-0.0128001	0.98874703
Gm43618	skyblue	-3.4279349	7.31E-06	-4.5364492	1.98E-06
Gm43205	red	-1.5954964	0.00527813	-0.3711441	0.57001983

Gbp5	yellow	4.51219819	2.46E-127	4.21302919	7.00E-112
Gm43714	orange	1.85553465	0.00723641	1.56165565	0.0232083
Igkv2-109	orange	0.03604912	0.97148153	2.14394989	0.00774526
Gm43272	yellow	2.31577474	0.00059146	1.86076576	0.0052487
Gm42659	orange	1.50502474	5.80E-05	1.16440003	0.00322277
Gm43328	orange	0.77640415	0.00049897	0.96855396	2.07E-05
Gm43305	yellow	2.57743276	7.93E-14	2.04497568	6.80E-09
Gm42748	orange	1.022565	0.06915051	1.58521958	0.00418479
Gm43595	skyblue	-2.2105175	3.66E-07	-1.5076821	0.00069945
Gm43351	black	5.63843062	0.00019048	6.13160804	6.72E-05
Gm43006	orange	0.91847427	0.00392937	0.41174052	0.28929003
Gm42141	yellow	2.92947738	0.0010223	2.32071576	0.01262595
Gm19439	skyblue	-1.890992	0.00021524	-1.3997137	0.00836336
Gm43508	red	-1.3592769	0.00021984	-0.8675322	0.02368544
Gm42517	skyblue	-2.1775385	1.39E-05	-0.9971751	0.05407283
4932422M17	red	-0.9086298	0.00018273	-0.5771612	0.02379969
Gm42780	skyblue	-2.812696	0.00362357	-0.9459885	0.39268573
AI506816	orange	1.75988537	3.86E-12	1.7634608	5.47E-12
Gm8493	orange	1.47669053	0.02599525	2.05192581	0.00255831
Gm4332	orange	1.74289696	0.00062115	0.02615654	0.96837975
Gm42892	skyblue	-2.2298609	6.42E-05	-1.0940425	0.07085237
Gm30648	skyblue	-1.9384013	0.00058447	-0.8270661	0.16587993
4930555A03I	skyblue	-1.9299658	0.00435306	-1.0129913	0.20547553
Gm42918	orange	1.62312058	0.00436669	1.08993255	0.06001162
Gm43464	yellow	2.07838166	0.0341869	3.36418441	0.00198095
Gm42658	orange	1.13415776	0.00044807	1.29451896	0.00014193
Gm8539	skyblue	-1.8928508	0.00051548	-0.40958	0.4645502
Gm20755	skyblue	-3.3136013	0.00020529	-2.7698308	0.00066261
C130083M11	red	-0.8531543	0.00769049	-0.3329676	0.34082196
Gm40348	skyblue	-4.0111493	0.00807526	-0.3347805	0.80390948
Gm36551	yellow	4.53733673	0.00119455	3.7215462	0.00511558
Gm9403	skyblue	-2.4677834	0.0065878	-0.5928653	0.54326182
Gm43181	yellow	3.84744773	0.00027239	3.49587789	0.00013152
Gm36266	red	-0.7860956	0.00495398	-0.4768721	0.11164513
Trav7-5	black	4.28245978	0.00511882	6.31728127	3.23E-05
Gm20559	orange	1.51371124	5.06E-31	1.50562879	1.60E-30
Gm43050	red	-0.3937177	0.07737312	-0.6332041	0.00441273
Gm10441	skyblue	-4.4087806	3.49E-05	-2.7203139	0.00246707
Peg13	skyblue	-1.3939806	1.11E-07	-1.2134153	6.59E-06
Gm43621	orange	1.06049286	0.00432193	0.62360272	0.10710292
5930430L01F	red	-1.0715103	0.00164019	-0.8555822	0.01615963
Gm43633	yellow	2.06071318	0.00201549	2.94783285	6.60E-05
Gm45495	skyblue	-1.3467004	6.33E-05	-1.1737173	0.00091766

Gm43068	black	6.66654841	3.73E-06	6.33190991	1.91E-05
Gm42726	orange	1.57199064	0.0022542	0.67148035	0.23289652
Gm15997	skyblue	-3.538033	0.00454728	-1.5787388	0.12345487
Gm42528	skyblue	-1.7307407	0.00088239	-1.1922981	0.0287851
Gm43496	skyblue	-2.3580751	0.00190625	-1.6525979	0.04161501
Gm43197	orange	1.59737465	6.47E-05	1.60199433	9.97E-05
Gm43769	yellow	1.55372327	0.10517331	2.96309447	0.00433244
Gm10461	red	-1.0387531	0.00973469	-0.5725188	0.2022919
Mpv17	red	-0.7677262	0.00068017	-1.0541083	3.86E-06
Gm43775	orange	1.67548861	0.00148228	1.72009052	0.00197977
Gm19719	yellow	2.19664875	0.00010757	2.07405411	0.0002903
Gm42549	orange	0.93558375	0.12329318	1.7350552	0.00559896
AI839979	orange	1.3333106	3.28E-05	1.03993288	0.00149767
Gstm2-ps1	skyblue	-1.7907033	0.00365024	-1.5022097	0.01524725
Gm43588	skyblue	-2.2185721	0.0032514	-1.0024319	0.24498004
Gm43323	orange	1.48744873	6.48E-05	0.88920967	0.03055499
Zfp862-ps	red	-1.1224608	4.62E-05	-0.9869866	0.00048127
Gm44165	black	6.52247733	7.35E-07	4.87756516	3.90E-08
Gm20560	red	-1.4353833	1.76E-05	-0.7530148	0.02508863
Gm38910	red	-0.9724603	0.00029	-1.044138	0.00015737
Gm44321	orange	0.91668137	0.09322223	1.91728026	0.00152571
Gm44013	yellow	3.03340393	0.00128765	0.67855422	0.51674024
Gm44401	orange	2.25300416	0.00223398	1.31137971	0.06014035
Gm43914	skyblue	-1.3157558	0.00158822	-1.2310549	0.00504859
Gm44000	skyblue	-2.1172499	4.16E-08	-1.3658749	0.00010169
Gm45769	red	-0.832537	0.00882709	-0.6664829	0.04387047
Gm43936	skyblue	-2.4629524	0.00564994	-2.9371261	0.0096533
Gm44250	red	-1.3605401	5.76E-08	-1.0768737	3.14E-05
Gm10388	yellow	2.19740299	1.86E-07	2.11525927	1.23E-06
Gm6375	orange	1.36673709	0.00319159	1.12560233	0.01948904
Gm44080	yellow	3.9999946	0.00043061	-0.1071035	0.94031162
Gm38708	red	-0.982983	3.58E-05	-0.8523315	0.00039554
B130021K23I	orange	0.6617335	0.35469316	2.32668742	0.00631557
Gm43890	yellow	3.81672531	1.10E-05	3.88979785	5.45E-05
Gm44423	orange	1.4683161	3.35E-05	1.95001725	5.44E-08
Gm43953	yellow	0.82848297	0.38215113	2.81854526	0.00783685
Gm44210	skyblue	-2.7869468	6.16E-06	-2.997447	5.01E-06
Gm6637	yellow	3.79706183	2.92E-09	2.86327066	3.50E-06
Gm45738	yellow	3.04476696	0.00532174	0.64429235	0.54580245
9930120I10R	orange	1.15016741	0.11293555	2.18624299	0.00310431
4930417O13	yellow	2.60515124	0.00010454	3.3353675	2.11E-05
Gm44175	yellow	1.70124582	0.07555434	5.42110399	6.46E-06
Gm44020	skyblue	-0.8874021	0.1511174	-2.0459983	0.00508591

1810059H22	skyblue	-1.958297	0.00322693	0.11657226	0.88956611
Gm43982	yellow	1.60450338	0.13942736	3.38926067	0.00934267
Olfr1372-ps1	orange	0.86474306	5.61E-05	0.7131999	0.00136588
Gm43984	orange	1.09982158	0.00857903	1.87453173	7.08E-06
Gm44187	orange	2.07793456	0.0052167	1.4295056	0.05181368
Gm36640	skyblue	-3.1256651	0.00165608	-1.0395458	0.28410633
Gm44224	skyblue	-3.9351975	0.00623599	-1.9884424	0.07014422
Gm44174	yellow	4.00013293	6.40E-09	3.47734992	3.87E-07
Gm44292	yellow	2.43679987	1.36E-10	2.40039988	5.59E-10
Gm44663	orange	2.03931225	0.00415584	1.24951973	0.12098249
5430431A17I	red	-0.8348965	0.00238374	-0.6897642	0.01753805
Gm42372	yellow	2.71504527	5.95E-09	1.88537024	2.85E-05
Gm44509	skyblue	-2.1511105	4.27E-06	-1.7700251	0.00026077
Gm32633	orange	0.76278173	0.00882694	1.18504761	5.52E-05
Snhg1	orange	1.12618258	6.53E-12	1.04938052	3.37E-10
Gm30928	skyblue	-2.5649308	1.63E-09	-1.8850896	1.18E-05
Gm44851	yellow	2.62833916	0.00021681	1.35918444	0.06878107
4930413G21	orange	0.22795486	0.3453769	0.80121481	0.00058329
4732496C06	red	-1.0805392	0.0001081	-0.968234	0.00075368
Gm33248	skyblue	-2.0371929	0.00196223	-1.1531256	0.09294098
Gm39094	skyblue	-4.1863806	0.0023209	-0.6106727	0.66914185
Gm33989	red	-0.8866789	0.00026368	-0.8902901	0.00034103
Gm34121	skyblue	-2.5550897	0.00789379	-1.3093933	0.17816636
Gm44758	red	-1.2421156	0.00330517	-0.8358284	0.06507124
Gm45226	skyblue	-1.9920317	0.00372021	-0.8839551	0.17682465
2310043P16I	orange	2.03753226	2.95E-10	1.23884716	0.0001901
Gm36028	skyblue	-2.3720535	0.00667811	-0.893392	0.27799673
Gm626	skyblue	-2.6235702	1.93E-10	-1.0749265	0.01140684
Gm44861	black	2.48381647	0.00033699	5.69338467	7.74E-06
9130015G15	skyblue	-2.2314383	1.07E-06	-1.6978025	0.00015335
Gm45221	orange	1.10457853	0.05651485	2.41957039	5.14E-05
2210406H18	yellow	2.29507143	0.04948761	3.82697498	0.00533133
4833411C07	red	-0.5662446	0.3331839	-1.6531097	0.00478162
Gm29683	skyblue	-3.9112275	7.22E-07	-1.4634307	0.02701306
Gm32031	orange	0.08579031	0.85239732	1.11928988	0.00655159
Gm44829	orange	1.61626529	0.00335263	0.7067598	0.25160536
Gm35339	skyblue	-1.3232854	4.06E-05	-1.2193283	0.00023091
Gm44659	black	4.91359672	0.00075347	3.71477812	0.00111086
Fam81b	skyblue	-2.5012716	8.21E-16	-1.4431312	1.00E-06
Gm44860	yellow	3.01564761	0.000762	3.57925437	0.00759381
Gm45220	yellow	1.90863465	0.00223941	2.44997609	0.0002925
Dcst2	orange	1.25971757	0.0063231	0.3433018	0.53059805
1810010D01	red	-1.1432688	0.00345901	-1.2679627	0.00160852

A330076H08	red	-0.9115895	0.05230055	-1.4605503	0.00242398
Prmt1	orange	0.86716287	3.81E-06	0.79066227	4.14E-05
Gm49396	orange	0.6557073	0.00077467	0.34485075	0.1030173
Gm5737	skyblue	-2.2814653	0.00055501	-2.1735604	0.00143926
A230057D06	red	-1.2158206	0.00080514	-0.8804705	0.02049839
A930037H05	orange	2.00989284	2.60E-07	1.86255797	2.09E-06
Gm45223	yellow	2.24180999	2.20E-05	1.72051763	0.00125425
Gm44710	red	-0.7763367	0.00143756	-1.1536177	4.66E-06
Gm45222	yellow	1.83196864	4.76E-09	2.10247792	9.13E-12
Nup62	orange	0.91609703	1.19E-12	0.76659752	6.19E-09
Gm44667	orange	1.47771482	0.00810446	1.75043263	0.00288339
Muc16	skyblue	-1.078832	0.00258539	-1.3149246	0.00032933
Cfap99	skyblue	-2.0201598	1.62E-05	-1.0685609	0.02360609
Gm31105	skyblue	-1.370315	0.00213794	-1.2355096	0.00571051
Gm45470	skyblue	-2.8508735	5.46E-06	-2.5607946	7.13E-05
Gm36869	yellow	2.57557495	0.00140705	4.01264261	0.0003428
Gm45606	orange	1.29793461	1.44E-05	1.20962359	0.00011383
Gm45418	yellow	2.8685285	0.00070667	2.33028992	0.00655162
Gm45640	orange	1.58171452	0.00023257	1.27373784	0.00477111
Gm45605	skyblue	-1.4942959	1.53E-07	-1.2366969	2.29E-05
Eid3	orange	0.88382106	0.03011283	1.37939803	0.00085408
Hspa14	orange	0.65847882	5.91E-11	0.52609645	4.26E-07
Gm45819	skyblue	-2.2663951	3.76E-09	-1.595464	4.60E-05
Gm45663	orange	1.6052821	0.00396248	0.22696201	0.74037112
Gm20071	red	-0.6539339	0.00268536	-0.32224	0.15372868
5430430B14I	red	-0.7428141	0.01832994	-1.0568388	0.00059779
Gm45353	skyblue	-2.2317914	0.01250806	-3.0727194	0.0055993
5430437J10F	yellow	2.72382645	1.27E-09	1.94768668	3.88E-05
Gm45471	black	4.16155302	8.63E-05	4.89999214	0.00011715
Gm45564	orange	0.64396921	0.31336196	2.20892397	0.00059237
5830408C22	orange	1.21959591	7.58E-05	0.30609378	0.36921797
Igip	red	-1.166909	8.86E-11	-1.0402046	1.57E-08
Flt3l	red	-1.1597032	4.17E-06	-0.8634347	0.00096801
Gm20219	red	-0.9154911	0.00012908	-0.6000539	0.01628575
Gm20100	yellow	3.51803025	1.94E-05	2.63510879	0.00134443
E330018M18	skyblue	-2.6399343	0.00267726	-1.8606511	0.02070819
Gm45552	orange	0.8151143	0.01061233	0.88311806	0.00633521
4930412F12I	orange	2.21167287	0.00808725	1.12527493	0.1979188
Gm19935	red	-1.0157633	0.00010255	-0.3763088	0.18998851
Gm45716	orange	1.44063572	5.81E-07	0.90123104	0.00226055
Gm33543	skyblue	-2.3162263	0.04712835	-3.2878886	0.00640911
Gm45332	skyblue	-1.9514929	0.0006537	-1.2397057	0.04230157
Gm42031	yellow	3.07047317	1.48E-14	3.08061097	1.74E-14

Gm45869	skyblue	-2.9092788	1.02E-05	-1.3429482	0.02468113
4933406B17	skyblue	-2.4904573	2.85E-07	-1.4007149	0.00297141
Gm45752	yellow	2.09255787	0.00079181	2.51522141	0.00012316
Gm45712	black	4.93737733	0.00114109	3.89255341	0.00159019
Gm4316	yellow	1.81597225	0.00012093	2.52961469	1.11E-06
Gm45774	yellow	2.59649572	0.00068537	3.50088232	4.84E-06
Gm45854	yellow	1.63650938	0.03593266	2.94959548	0.00074427
Gm39822	orange	1.0017291	6.69E-05	0.75267774	0.00387027
Gm42047	yellow	2.12761999	3.07E-07	3.49745703	1.68E-17
A530010L16	yellow	2.09938166	1.62E-07	2.05594233	8.08E-07
Gm31718	yellow	3.90808771	3.73E-15	2.39447166	1.27E-07
Gm45767	red	-0.9602192	0.00758845	-0.8658616	0.01715237
Gm33104	yellow	2.50736397	1.97E-06	1.12682672	0.03160294
D030034A15	skyblue	-2.158877	0.0039659	-1.8203403	0.01487096
Gm35028	black	2.38531517	0.0266447	6.05786012	0.00012544
Gm20300	red	-1.0990336	3.96E-07	-0.8062899	0.00040719
Gm48065	yellow	4.33210197	0.00086649	2.28176038	0.00967129
Gm6545	black	5.41804889	1.62E-27	4.97184154	1.99E-23
AC160562.1	yellow	1.8583942	0.00797633	2.27687933	0.00274953
Gm39323	yellow	3.8574184	2.74E-13	4.52419455	5.06E-15
Gm48717	skyblue	-1.4632099	0.00794271	-1.148171	0.05094527
E030022I16	orange	1.58975066	0.00501983	1.79831889	0.0027491
Gm47445	orange	1.84001462	0.00404751	0.50828946	0.5008398
AC160637.1	orange	1.77455025	7.48E-12	1.60562199	1.04E-09
Gm48478	yellow	0.8936144	0.32900856	3.11227607	0.00417494
Gm47050	yellow	3.87567504	3.22E-11	2.25543045	3.67E-05
1700019L13	black	6.35937137	1.14E-09	5.41311271	1.21E-10
Gm47640	red	-0.9813011	0.0011283	-0.6489341	0.03265177
Gm30373	yellow	3.93877652	9.13E-06	3.7292023	1.82E-05
Gm33858	yellow	2.75902675	0.00267196	3.48926497	0.00126591
Gm47950	orange	1.18090166	0.00072494	1.08166328	0.00212371
1190001M18	yellow	2.4937375	0.00070729	2.69880601	0.00093941
Gm6477	orange	0.90521537	0.00992148	0.81697494	0.03254139
AC153498.1	black	7.3821923	3.51E-07	5.99895871	6.45E-05
Gm36660	skyblue	-1.31977	0.0005278	-1.2863242	0.00113394
Gm47218	orange	1.4917721	0.00040513	1.70763337	2.77E-05
Lilr4b	black	5.02689019	8.49E-56	5.12231838	7.20E-58
A130077B15	black	4.93527651	0.00075305	3.17348792	0.00127092
C230072F16	skyblue	-2.0411058	9.35E-06	-0.6086631	0.20767152
Lilrb4a	yellow	3.77196945	1.58E-27	4.04140139	2.13E-31
Gm19990	red	-0.9542786	0.00080904	-0.4036258	0.1945056
Ifngas1	yellow	2.82251778	0.00047537	4.1584999	3.25E-05
4930466K18	skyblue	-1.5077149	9.86E-05	-1.4442838	0.00041457

Sfta3-ps	red	-1.4597417	6.12E-05	-1.0026668	0.00845476
Gm48768	red	-0.8183413	0.00677807	-1.061223	0.00047163
Gm47720	skyblue	-1.8083234	0.00055555	-1.6277915	0.00272674
Gm29684	yellow	2.36925025	0.0007684	2.97844872	0.00025874
Gm33091	yellow	3.80164389	9.17E-08	2.92530808	3.30E-05
Gm35696	skyblue	-2.6565004	6.98E-05	-0.7692597	0.23774188
Gm36283	skyblue	-1.3459341	3.25E-05	-1.1690254	0.00056109
Gm47461	skyblue	-5.7982979	0.00016003	-3.3852684	0.00461519
Gm48882	skyblue	-1.806205	0.00562701	-1.6474023	0.01740642
Gm48835	skyblue	-2.9959546	1.55E-06	-2.0666848	0.0004246
4933412E12I	orange	1.03095246	1.02E-11	0.83808275	6.69E-08
Gm4798	skyblue	-3.47023	0.00579537	-1.0178637	0.36074186
Gm47644	red	-0.5339171	0.36350721	-1.7771639	0.00250795
Gm36172	yellow	3.42052329	0.00757858	1.07747156	0.33259946
Gm33677	skyblue	-3.1369004	0.00202496	-4.3406724	0.00137808
Gm38407	yellow	2.10656581	0.00025178	1.78048807	0.00398995
Gm4739	yellow	2.38897493	4.10E-05	2.15217106	0.00173123
Gm46224	black	4.37080425	1.77E-14	4.67711466	1.14E-12
Gm47567	black	5.84032927	0.00023725	2.26928366	0.08212207
Gm47015	orange	1.37852112	2.46E-05	1.42657582	2.16E-05
D430020J02I	yellow	1.81606957	2.23E-06	2.10421227	8.93E-08
Gm33424	skyblue	-1.9204816	0.0096079	-1.55796	0.06048236
A930040O22	skyblue	-2.8669748	0.00350096	0.04797765	0.96368171
Gm47139	skyblue	-1.9045343	0.00019285	-0.538702	0.30947578
Gm19951	yellow	4.28033155	5.39E-21	3.82479285	9.10E-17
Gm7511	skyblue	0.3247071	0.71114819	-3.6811837	0.00646963
AC125351.1	red	-1.090083	4.54E-05	-0.7363885	0.00784559
Gm46430	red	-0.6778895	0.00181271	-0.7822626	0.00037517
Gm32219	skyblue	-1.9053523	0.00038499	-2.0825866	0.00059518
Gm47260	orange	0.89699722	0.00263174	0.70456267	0.0220235
Gm49329	skyblue	-0.3360595	0.64156089	-2.159474	0.00304524
AC163354.1	yellow	2.17366777	5.67E-07	1.66612789	0.00022383
5033406O09	orange	1.35468058	0.00578915	0.4277492	0.43541492
AC121965.1	orange	0.65757642	0.00019629	0.55775321	0.00235012
Gm6566	orange	1.56414097	0.00036751	0.53105903	0.27072114
Gm40909	yellow	1.48418763	0.0316604	2.75071043	5.63E-05
Gm47155	skyblue	-1.3555022	0.00407154	-1.1854482	0.01870128
Gm47705	yellow	3.71108629	7.69E-07	4.02015322	3.84E-06
4930549C15	skyblue	-1.020442	0.18283021	-2.9119023	0.00126509
Gm48662	skyblue	-2.5627526	2.72E-13	-2.4397828	1.51E-11
Gm47662	yellow	3.35353997	0.00125591	2.94629136	0.00493009
Gm35279	yellow	3.32457579	0.0001426	3.23335127	0.00108436
Gm38655	skyblue	-3.4144914	2.09E-06	-1.966137	0.00590871

Gm48600	red	-1.2141706	0.00613197	-1.0155638	0.02962795
Gm49027	skyblue	-1.9294472	4.92E-06	-0.3104483	0.49904645
Gm47773	orange	1.9273813	0.00703369	-0.5213523	0.52097398
Gm31544	black	5.90382423	3.70E-05	4.63853153	0.00057046
F630042J09F	skyblue	-1.0647579	0.00378483	-1.3899179	0.00044855
Gm48500	skyblue	-2.0615672	0.00169244	-1.3799356	0.03633584
Gm47732	skyblue	-2.7808424	0.00041578	-1.2997689	0.10448706
Gm36161	yellow	4.31334215	3.36E-15	4.12929118	4.81E-13
Gm47242	black	6.05854811	7.43E-14	6.56854656	3.08E-11
Gm47754	yellow	2.92352375	0.00075421	2.48125529	0.00365013
Gm21370	black	3.61747483	0.00029434	5.24837875	0.00011108
4930579J19F	skyblue	-3.0216992	1.07E-05	-2.6507871	5.52E-05
Gm49284	red	-1.2548104	1.37E-11	-1.0055666	4.82E-08
G930009F23	orange	0.02789552	0.95533971	1.37143381	0.00169162
Gm49041	orange	1.93798453	5.84E-07	1.90316739	3.67E-06
Gm34934	red	-1.3715394	9.96E-07	-1.038268	0.00030681
Eef1akmt4	orange	1.71818152	1.87E-06	1.32364021	0.00030081
AC174678.1	orange	0.83985996	0.00528908	0.29349637	0.38702305
AU022793	yellow	2.70773917	1.19E-06	1.71631554	0.00179829
9930017N22	orange	0.9756187	0.00846337	0.12625005	0.78534677
Gm32618	skyblue	-1.6827222	1.04E-09	-1.7462057	1.65E-09
Gm35019	yellow	3.29753783	0.00031595	1.16467435	0.19648641
Pnp	yellow	2.13943279	7.24E-27	1.81194477	2.25E-19
Eppk1	orange	0.55441606	0.01101541	0.61750816	0.00551244
Gm19510	yellow	3.65235619	0.00620704	3.98658012	0.00997863
Gm36107	black	4.59821256	0.00730069	2.7930739	0.06690972
Gm32857	skyblue	-3.3832442	0.00024425	-3.0173992	0.00042109
Gm2682	orange	1.62717473	7.72E-06	1.89801507	5.29E-07
Gm48972	black	5.36338208	0.00034084	3.83740991	0.00719402
AC160336.1	skyblue	-3.6506971	1.33E-05	-2.4358546	0.00476411
Gm49092	skyblue	-2.7267305	0.00892564	-1.1737613	0.17492024
AC113595.1	yellow	2.38102927	1.41E-15	2.45553026	5.32E-16
AC110211.1	yellow	2.1868477	0.00599264	1.60656402	0.03715913
AC119264.1	yellow	3.05984496	1.86E-09	3.38756999	1.00E-08
AC102334.1	skyblue	-2.1121876	0.00044043	-1.9652785	0.00083408
AL592187.2	black	5.95534189	4.53E-05	4.99499198	0.00098223
AC157583.2	red	-1.3608246	0.00106093	-0.8588569	0.04838148
AC132863.2	orange	1.90739016	0.00041111	0.96105432	0.11552136
AC123724.2	red	-1.2039947	0.0001516	-1.0597591	0.00125356
AC147041.1	orange	2.29485656	0.00010624	1.2706041	0.03105285
AC154311.1	skyblue	-2.1202439	0.00025621	-1.165666	0.04497613
AC139671.1	orange	1.30594081	5.76E-05	0.92333568	0.00509047
AC163018.2	orange	2.53300986	0.00233617	-0.0593201	0.95154216

AL592187.7	yellow	3.83653883	0.00097221	4.00342217	0.00378464
AC138177.4	skyblue	-2.7995145	0.00911745	-2.6738957	0.02318518
AC123724.3	yellow	2.46795076	1.13E-13	1.9099381	2.50E-07
Gm2808	yellow	3.76976661	4.10E-07	2.32952518	0.00025999
AC126280.1	skyblue	-1.1277784	0.06595928	-1.7980013	0.004754
AC129574.2	yellow	4.04171405	1.47E-05	1.62342673	0.04749579
AC131339.2	red	-0.8706361	0.03991216	-1.5276986	0.00037207
AC127341.3	yellow	2.40912697	5.41E-05	3.00149841	2.62E-06
AC154478.1	black	3.02407417	0.01290094	5.77647434	0.00056179
AC125141.3	orange	2.04806676	0.00819024	1.92079534	0.03903846
AC161607.1	skyblue	-1.5647635	1.87E-05	-1.3256148	0.00033469
AC117241.2	black	6.98196009	2.30E-05	6.40927253	0.0001693
AC130815.3	orange	1.26724043	5.43E-07	0.73765151	0.00526903
